# Enhancement of the Performance of Large Language Models in Diabetes Education through Retrieval-Augmented Generation: Comparative Study

**DOI:** 10.2196/58041

**Published:** 2024-11-08

**Authors:** Dingqiao Wang, Jiangbo Liang, Jinguo Ye, Jingni Li, Jingpeng Li, Qikai Zhang, Qiuling Hu, Caineng Pan, Dongliang Wang, Zhong Liu, Wen Shi, Danli Shi, Fei Li, Bo Qu, Yingfeng Zheng

**Affiliations:** 1 State Key Laboratory of Ophthalmology, Zhongshan Ophthalmic Center, Sun Yat-sen University Guangdong Provincial Key Laboratory of Ophthalmology and Visual Science Guangdong Provincial Clinical Research Center for Ocular Diseases GuangZhou China; 2 Research Centre for SHARP Vision The Hong Kong Polytechnic University Hong Kong China; 3 Peking University Third Hospital Beijing China

**Keywords:** large language models, LLMs, retrieval-augmented generation, RAG, GPT-4.0, Claude-2, Google Bard, diabetes education

## Abstract

**Background:**

Large language models (LLMs) demonstrated advanced performance in processing clinical information. However, commercially available LLMs lack specialized medical knowledge and remain susceptible to generating inaccurate information. Given the need for self-management in diabetes, patients commonly seek information online. We introduce the Retrieval-augmented Information System for Enhancement (RISE) framework and evaluate its performance in enhancing LLMs to provide accurate responses to diabetes-related inquiries.

**Objective:**

This study aimed to evaluate the potential of the RISE framework, an information retrieval and augmentation tool, to improve the LLM’s performance to accurately and safely respond to diabetes-related inquiries.

**Methods:**

The RISE, an innovative retrieval augmentation framework, comprises 4 steps: rewriting query, information retrieval, summarization, and execution. Using a set of 43 common diabetes-related questions, we evaluated 3 base LLMs (GPT-4, Anthropic Claude 2, Google Bard) and their RISE-enhanced versions respectively. Assessments were conducted by clinicians for accuracy and comprehensiveness and by patients for understandability.

**Results:**

The integration of RISE significantly improved the accuracy and comprehensiveness of responses from all 3 base LLMs. On average, the percentage of accurate responses increased by 12% (15/129) with RISE. Specifically, the rates of accurate responses increased by 7% (3/43) for GPT-4, 19% (8/43) for Claude 2, and 9% (4/43) for Google Bard. The framework also enhanced response comprehensiveness, with mean scores improving by 0.44 (SD 0.10). Understandability was also enhanced by 0.19 (SD 0.13) on average. Data collection was conducted from September 30, 2023 to February 5, 2024.

**Conclusions:**

The RISE significantly improves LLMs’ performance in responding to diabetes-related inquiries, enhancing accuracy, comprehensiveness, and understandability. These improvements have crucial implications for RISE’s future role in patient education and chronic illness self-management, which contributes to relieving medical resource pressures and raising public awareness of medical knowledge.

## Introduction

### Background

Diabetes mellitus is a chronic long-term illness that requires continual health education and assistance to improve patient outcomes [1,2]. The shortage of diabetes counselors and the limitations of traditional education methods make it challenging to address the unique requirements of each diabetic patient [3]. Large language models (LLMs), such as ChatGPT, hold significant promise in diabetes self-management and information assessment [3-8]. However, concerns exist around the accuracy and reliability of these models, mainly stemming from the variable credibility of their training data which is sourced from a wide variety of internet text and self-supervised learning [9-11]. Furthermore, LLMs may lack domain-specific knowledge, risking the production of potentially inaccurate responses [12-15].

Recent studies have primarily assessed the capabilities of LLMs in responding to diabetes-related questions, revealing limitations in their expertise in medical specialties, which remain unresolved. For example, Meo et al [16] indicated both ChatGPT and Google Bard scored below 60% in endocrinology and diabetes. They concluded that while these artificial intelligence tools show potential in academic medical education, they require more updated information in these specific medical fields. Goodman Rachel et al [17] also highlighted the precision of chatbots in medical queries and underlined the need for further research and model development for enhanced accuracy and validation in clinical practice. Hulman et al [18] showed that ChatGPT-generated responses could be distinguished from expert responses by 59.5%, suggesting a gap compared with expert human performance. Therefore, addressing these gaps by augmenting LLMs with more specialized knowledge and updated information is crucial for improving their role in patient understanding and management of diabetes.

In response to these unresolved challenges, our study introduces “RISE” (Retrieval-augmented Information System for Enhancement), an independent workflow designed to enhance the performance of LLMs in the medical domain by automatically retrieving real-time external knowledge. We used LLMs with and without RISE to answer diabetes-related inquiries from patients, assessing the improvements that RISE brings to the original LLMs in terms of accuracy, comprehensiveness, and understandability. Our RISE aims to bridge the knowledge gaps identified in LLMs, providing a more robust and reliable tool for addressing patient concerns about diabetes management and understanding.

The main contributions of our work are (1) we introduce RISE, an innovative framework based on the retrieval-augmented generation (RAG) algorithm that enhances LLMs with real-time, domain-specific knowledge to provide accurate and comprehensive responses to diabetes-related inquiries, improving patient self-management and outcomes. (2) We reduce the risk of inaccurate or irrelevant responses from LLMs by integrating local and external real-time information retrieval, enhancing model transparency by identifying source information. (3) We incorporate an additional module for accuracy and safety checks before responding, ensuring that the provided information is reliable and free from harmful content. (4) We validate the RISE framework through assessments by clinicians and patients to demonstrate the feasibility of adopting RISE-enhanced LLMs in diabetes management and education.

### Related Works

#### Large Language Models

LLMs, such as GPT-3 [19], GPT-4 [20], and PaLM [21], have garnered significant attention due to their exceptional language understanding and generation capabilities [22,23]. However, when applied to domain-specific tasks, particularly in the medical field, their performance may be limited by a lack of specialized knowledge and vocabulary in the training data [24-26]. Adapting LLMs for biomedical applications presents several challenges, including insufficient domain knowledge and high computational costs. As a result, only a few LLMs have been fine-tuned for medical consultation using open-source models with 6.5-13 billion parameters, such as ChatDoctor [27] and MedAlpaca [28]. However, this approach of fine-tuning open-source models has its limitations. Medical domain-specific models often use relatively smaller-scale LLMs (eg, LLaMA [27] with 7B parameters), which may result in lower accuracy and robustness, compared with GPT-4 [29]. Moreover, fine-tuning even these smaller LLMs is computationally intensive and costly [27]. The introduction of new knowledge requires complete retraining of the model, placing additional burdens on developers. Furthermore, LLMs are generally prone to hallucination, which is a challenge that fine-tuning struggles to address [17,30-33].

#### Retrieval-Augmented Generation in Medical Questions and Answers

Recent studies have explored the application of RAG [34,35] in the medical domain to enhance the performance of LLMs in question-answering tasks. These approaches enable LLMs to achieve improved performance without needing time-intensive and costly fine-tuning while facilitating timely updates without retraining the entire model.

In specialized medical domains, LLMs have been augmented with limited medical corpora to address specific areas such as liver diseases (LiVersa) [36], diffuse large B-cell lymphoma [32], and nephrology [37]. Simultaneously, in the general medical context, frameworks such as Almanac [38] and RECTIFIER [39] have been proposed to integrate LLMs with medical guidelines and treatment recommendations.

Despite their potential, these approaches also present several limitations. The effectiveness of RAG-based models largely depends on the quality and currency of the used data sources. The previous studies typically rely on fixed and related smaller knowledge bases, such as Wikipedia or guidance documents, thereby limiting their effectiveness in specialized medical domains [40,41]. Outdated or incorrect information can result in inaccurate or misleading outputs. Furthermore, retrieval errors or the inclusion of biased and unsafe content inherent in the LLMs, without further filtering, may lead to inaccuracies in the generated output, potentially misleading patients.

Our RISE framework addresses these limitations by comminating with local and internet-based knowledge sources, curated from over 200 reputable academic websites, ensuring access to a wide range of up-to-date clinical evidence. Moreover, we incorporate additional fact-checking and safety check modules before responding. By prioritizing the accuracy and safety of the retrieved information, our framework offers a more reliable and secure pathway for answering clinical questions, significantly reducing the risk of misleading patients.

## Methods

### Framework of Retrieval-Augmented Information System for Enhancement

**Figure 1:**
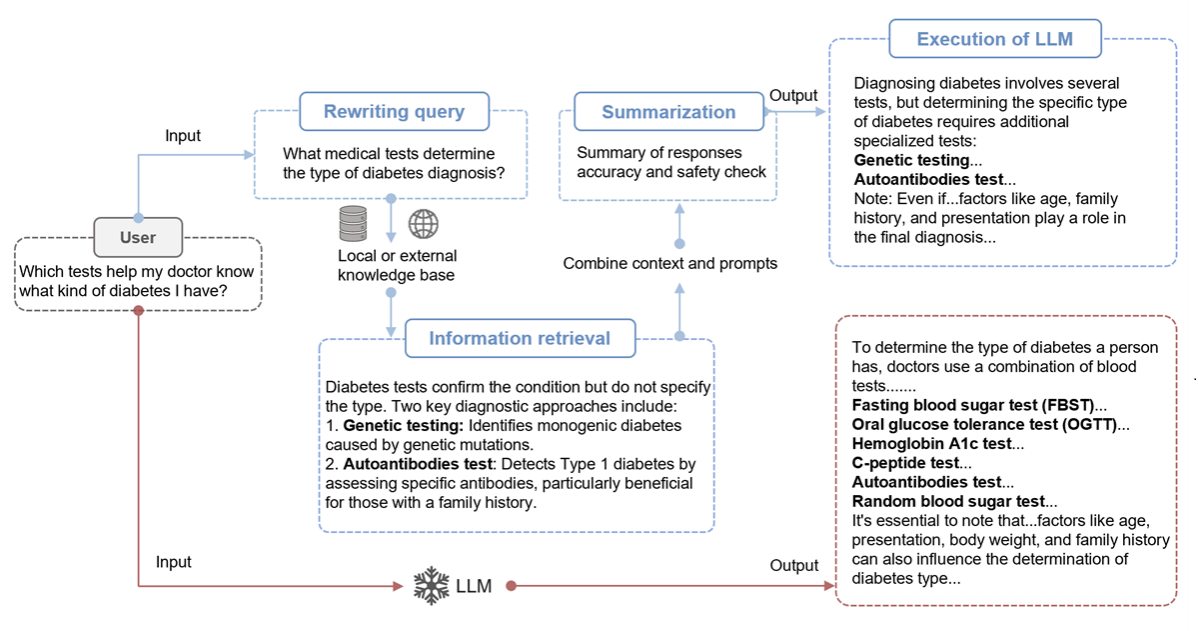
Comparing responses of LLMs before and after “RISE” integration. Red bars: response from base LLMs without RISE framework. Blue bars: overview of RISE framework and query response after integration with RISE. The framework of RISE: (1) Rewriting query: Improve query accuracy and relevance using large language models. (2) Information retrieval: Search for relevant information using the revised query from the local data set and external knowledge base. (3) Summarization: Summarize retrieved information into concise key points, combined with fact-checking and safety checks. (4) Execution: LLMs take action based on summarized information (for implementation details, refer to Multimedia Appendix 1). LLMs: large language models; RISE: Retrieval-augmented Information System for Enhancement.

Our study introduced the RISE framework, an innovative approach designed to improve the performance of medical question answering of LLMs. Our novel algorithm derives from RAG [34,42,43], which retrieves pertinent information from local databases or external knowledge from academic websites. Our RISE is a standalone framework comprising four steps (Figure 1 and Multimedia Appendix 1).

### Rewriting Query

The first step in the RISE framework involves rewriting the original query using advanced LLMs, including GPT-4, Claude 2, and Google Bard (Alphabet Inc; subsequently rebranded as Gemini). This process aims to enhance the query by correcting spelling errors, expanding abbreviations, and incorporating synonyms, thereby broadening the scope of potential results.

### Information Retrieval

Relevant information is retrieved from a local vectorized database and external knowledge sources. The rewritten query is embedded in the same vector space as the database, and the Facebook AI Similarity Search (FAISS) algorithm is used for similarity search to find the top 5 most pertinent documents (retriever=vectorstore.as_retriever (search_type=“similarity,” search_kwargs={“k:” 5}), results = retriever.invoke [query]). If no results are found locally, external knowledge is sourced from academic websites (over 200), ensuring that all information adheres to stringent academic and research standards.

### Summarization

The third step involves summarizing the retrieved information into a concise and understandable format by prompt. This step also includes fact-checking and safety checks to ensure accuracy and reduce harmful content.

Fact-checking is performed in 2 steps. First, the retrieved raw text and the question are input, and the retrieved text is broken down into multiple claims. Second, these claims and the question are input, allowing the model to self-check which claims are confirmed using external knowledge sources. The model then returns the verified claims as the final summarization text.

The safety check process uses a set of 24 rules to restrict and filter the content, ensuring the generated responses are safe and appropriate. The model is prompted with the instruction, that is “Your answer must adhere to the following rules: {rules}.”

### Execution

The final step involves presenting the summarized information and prompts to the LLMs to generate the final answer for the user. The prompt instructs “Use the following pieces of context to answer the question at the end. Note that your response should be as brief as possible and no more than 300 words. If you don’t know the answer, just say that you don’t know, don’t try to make up an answer.”

### Local Database

A local database of diabetes-related information was created to provide domain-specific knowledge for the RISE framework. PubMed Central [44] was used to acquire a corpus of scientific papers and clinical practice guidelines relevant to diabetes. The database covers various aspects of diabetes, including pathophysiology, diagnosis, treatment, management, and patient education, rather than answering specific questions used in the evaluation. The retrieved documents comprise over 600 full-text articles.

The retrieved documents were then preprocessed to remove potentially unstructured or noisy information, such as figures, tables, references, and author disclosures. After cleaning each document, the CharacterTextSplitter function from Langchain was used to divide the documents into smaller fragments. We then used the OpenAI model Text-Embedding-ADA-002 as an embedding function to generate embeddings for each fragment in FAISS using the function “db=FAISS.from_documents (docs, embeddings),” where “docs” refers to the document fragments and “embeddings” refers to the Text-Embedding-ADA-002 model. The resulting index was saved locally for continuous access and retrieval using the function “db.save_local (“faiss_index”).”

When a user submitted a question, the rewriting query was transformed into an embedding vector and compared with the database embeddings using cosine similarity. The top k=5 document segments with the highest similarity scores were retrieved and used as the knowledge context for the user’s query. A sample of the data set and related code are provided in Multimedia Appendices 1 and 2.

### Study Design

#### Overview

This study was conducted at Sun Yat-sen University from September 25, 2023, to February 30, 2024. The 43 diabetes-related questions were selected from the National Institute of Diabetes and Digestive and Kidney Diseases website [45] across the following 5 domains, that are concepts of diabetes, symptoms and causes, diabetes tests and diagnosis, managing diabetes, and prevention. The questions aimed to cover topics commonly asked by the public and patients regarding diabetes care.

#### Respond Generation

We prepared a set of 43 diabetes-related inquiries to be posed to 3 base language models - GPT-4, Claude 2, and Google Bard - as well as their respective versions enhanced by RISE. In total, there were 6 models involved, with an enhanced version corresponding to each base model. From September 30, 2023, to February 5, 2024, we independently fed the entire set of 43 inquiries into each of the 6 models, treating each question as a separate input and resetting the conversation between queries to minimize bias.

**Figure 2:**
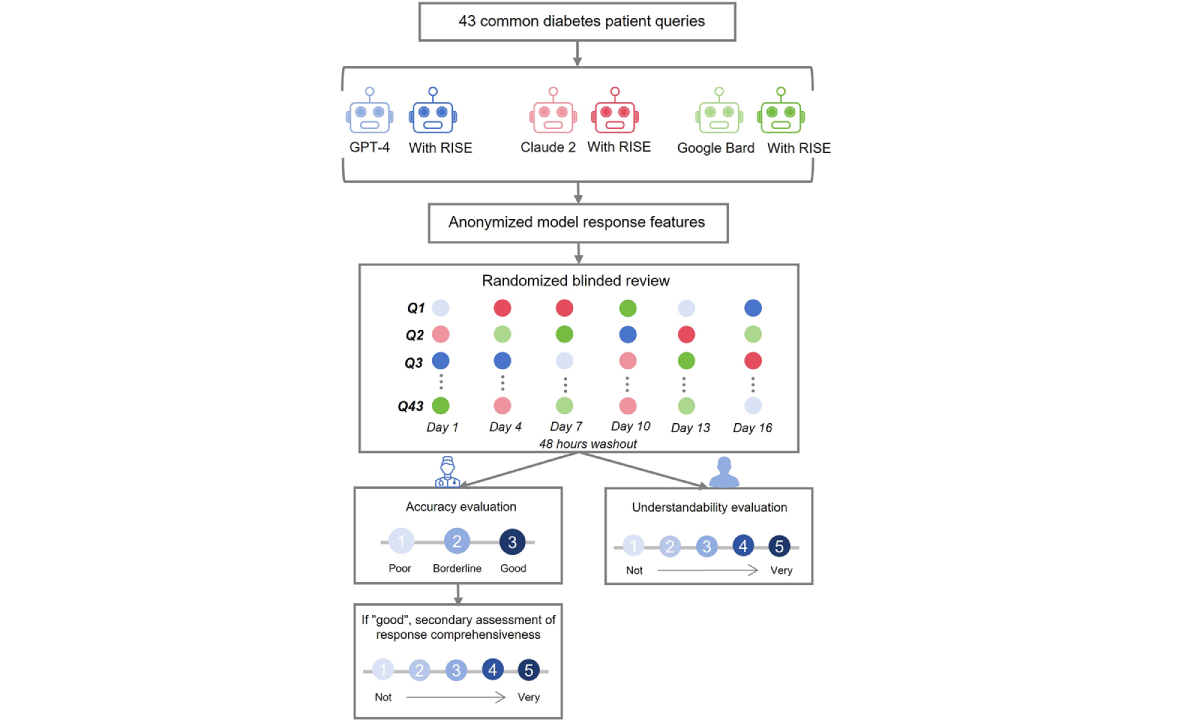
Flowchart of overall study design. The study evaluates the performance of 3 publicly available large language models and their RISE-enhanced versions in addressing common diabetes-related inquiries. The evaluation is conducted from the perspectives of both the clinicians and diabetic patients. Clinicians evaluate the accuracy and comprehensiveness of responses. Patients assess the understandability.

Model responses were evaluated in a blinded, randomized manner through 2 aspects - first by clinician assessment focusing on accuracy and comprehensiveness, and then by patient evaluation of understandability. The evaluation process involved clinicians with over 5 years of experience in general medicine. The responses from all 6 models were shuffled randomly into 6 different rounds. To remove potential model indicators from responses, they were transformed into plain text before being distributed to 3 clinicians and 3 diabetes patients. They all analyzed responses across 6 rounds spaced 48 hours apart to eliminate confounding (Figure 2). Responses for each model and raw scores for evaluation are provided in Multimedia Appendix 3.

### Accuracy Evaluation

We conducted an accuracy evaluation for each response by assigning scores and ratings. A “Poor” rating received 1 point, “Borderline” received 2 points, and “Good” received 3 points. Each response underwent assessment by 3 clinicians. For scoring, the score for each question is the sum of the score assigned by the 3 graders, with a maximum possible score of 9. For rating, we used a majority consensus method among the 3 clinicians. A response was considered “Good” only if more than 2 clinicians rated it as such. In cases where the 3 clinicians provided differing ratings, we implemented a stringent strategy by giving the response the lowest mark (ie, “poor”). The accuracy rate is defined as the proportion of responses with a final rating of “Good.”

The accuracy scoring criteria include (1) “Poor” indicating replies containing mistakes that might considerably mislead patients and potentially result in damage, (2) “Borderline” assigned to answers with potential inaccuracies but unlikely to misguide or damage patients, and (3) “Good” reserved for replies without errors.

### Comprehensiveness Evaluation

For responses that obtained a “good” rating by majority consensus, the clinicians further evaluated the comprehensiveness of each response. A 5-point scale was used (1) “not comprehensive” for reactions critically missing information (1 point), (2) “slightly comprehensive” for replies with limited but primary details (2 points), (3) “moderately comprehensive” for reactions providing more than half of the essential information (3 points), (4) “comprehensive” for reactions covering most critical points (4 points), and (5) “very comprehensive” for reactions giving comprehensive information (5 points). For each response, the average score was calculated by the mean of the scores assigned by the 3 clinicians.

### Understandability Evaluation

A total of 3 diabetic patients evaluated response understandability. A 5-point scale different from comprehensiveness evaluation was used; (1) “very poor” for responses difficult to understand or completely irrelevant (1 point), (2) “poor” for responses somewhat difficult to understand or partially irrelevant (2 points), (3) “average” for responses generally understandable but requiring some effort or having minor ambiguities (3 points), (4) “good” for responses most of which are easily understandable with very few unclear parts (4 points), and (5) “Excellent” for responses very clear and easy to understand, fully meeting the reader’s needs (5 points). For each response, the average score for understandability was calculated based on the score given by each patient.

### Statistical Analysis

Statistical analyses were used SPSS (version 22.0; IBM Corp). Normal distribution was assessed with Kolmogorov–Smirnov test. Our data were found not to follow a normal distribution, *P*<.001. Group comparisons used the Wilcoxon signed-rank test for accuracy, comprehensiveness, and understandability scores with and without RISE. For the comparison of the proportions of “good,” “borderline,” and “poor” ratings across the models, the chi-square test was used. *P* values <.05 were regarded as significant.

### Ethical Considerations

This study involved publicly available data without collecting human or animal samples and data. The study has been approved by the ethics committee of Zhongshan Ophthalmic Center (ZOC, Guangzhou, China; approval number 2024KYPJ124).

## Results

### Accuracy Evaluation

**Figure 3:**
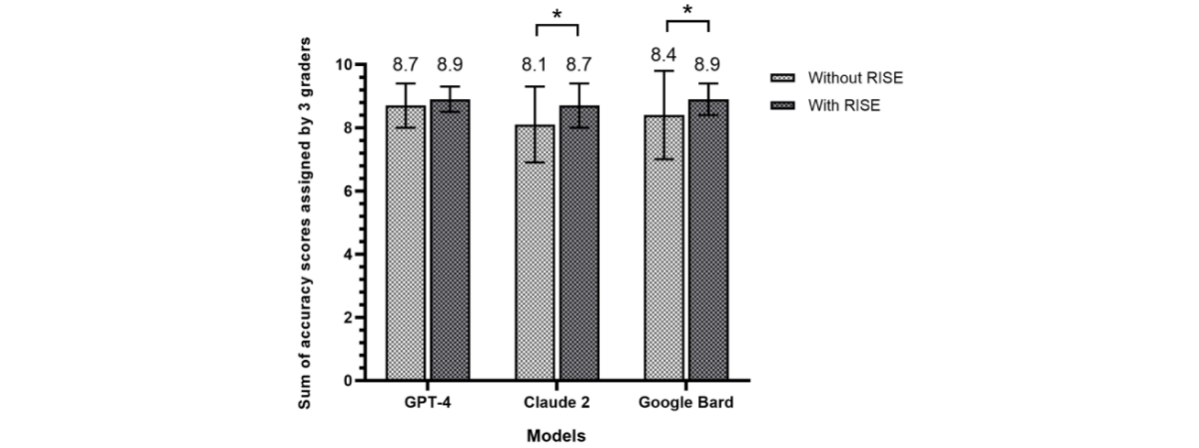
Average scores of responses from large language models. Answers from each model were scored 1-3 points by 3 clinicians. The maximum score for each response is 9 points. An asterisk (*) denotes statistical significance at *P*<.05. Model call dates: September 30, 2023 to February 5, 2024. RISE: Retrieval-augmented Information System for Enhancement.

We evaluated 3 LLMs and their RISE-enhanced versions for answering diabetes-related questions. As shown in Figure 3, the average accuracy scores of all 3 original models increased substantially with the RISE enhancement. Specifically, the accuracy scores improved from 8.72 (SD 0.70) to 8.91 (SD 0.37; *P*=.09) for GPT-4 after applying RISE, 8.09 (SD 1.23) to 8.65 (SD 0.65; *P*=.001) for Claude, and 8.37 (SD 1.36) to 8.86 (SD 0.47; *P*=.01) for Bard (maximum score per response is 9 points).

**Figure 4:**
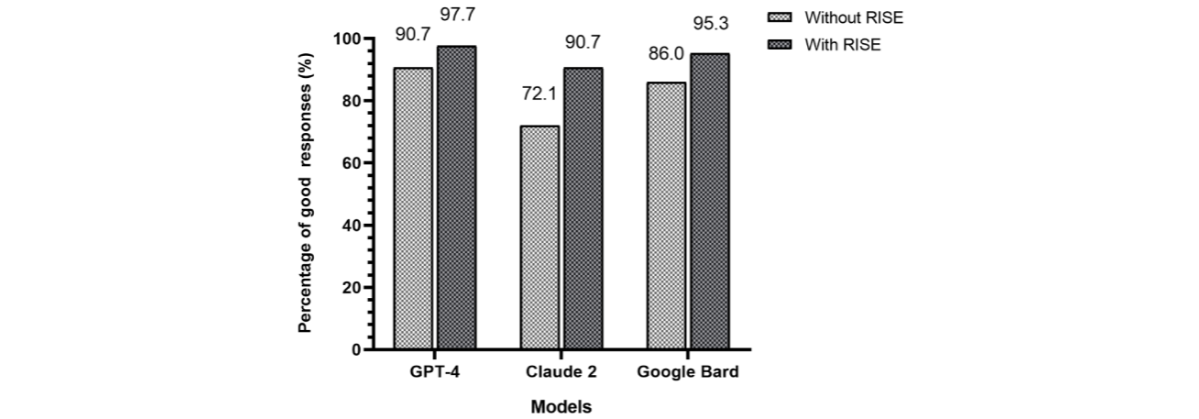
Accuracy rates (proportion of “Good” responses) of large language models. Model call dates: September 30, 2023 to February 5, 2024. RISE: Retrieval-augmented Information System for Enhancement.

We further evaluated the percentage rated as “Good”, representing accuracy rates, of the LLMs with and without RISE (Figure 4). The results showed increased accuracy rates after incorporating RISE across all original models. Specifically, after the incorporation of RISE, the proportion of accuracy responses for GPT-4 increased from 91% (39/43) to 98% (42/43), for Claude from 72% (31/43) to 91% (39/43), and for Bard from 86% (37/43) to 95% (41/43). Furthermore, GPT-4 enhanced by RISE exhibited the highest accuracy rates, reaching 98% (42/43). In addition, Table 1 presents the accuracy of the models across 5 domains. All 6 models achieved the highest accuracy, reaching 100% (16/16), in the “Preventing Diabetes Problems” domain. However, in the “Concepts of Diabetes” and “Symptoms & Causes” domains, the models exhibited relatively lower average accuracy rates.

**Table 1:** Accuracy of model response across 5 diabetes educational domains.

Domain	Responses, n	GPT-4, n (%)		Claude 2, n (%)			Google Bard, n (%)		
		Without RISE^a^	With RISE	Without RISE	With RISE	Without RISE		With RISE	
Concepts of diabetes	4	2 (50)	4 (100)	2 (50)	2 (50)	2 (50)		4 (100)	
Symptoms and causes	4	3 (75)	3 (75)	1 (25)	3 (75)	2 (50)		4 (100)	
Diabetes tests and diagnosis	4	4 (100)	4 (100)	3 (75)	4 (100)	3 (75)		3 (75)	
Managing diabetes	15	14 (93)	15 (100)	9 (60)	14 (93)	14 (93)		14 (93)	
Preventing diabetes problems	16	16 (100)	16 (100)	16 (100)	16 (100)	16 (100)		16 (100)	
Total	43	39 (91)	42 (98)	31 (72)	39 (91)	37 (86)		41 (95)	

^a^RISE: Retrieval-augmented Information System for Enhancement.

Accuracy rates indicate the percentage rated as “Good” in accuracy evaluation.

### Comprehensiveness Evaluation

The study also assessed the comprehensiveness of model responses through a 1- to 5-point rating scale by 3 clinicians for the responses rated as “good” (Table 2). The results revealed that the integration of RISE led to a decrease in the number of responses with scores lower than 3 and an increase in the number of responses with higher scores of (4,5]. For instance, after incorporation of RISE, the number of responses scoring (1, 2] and (2, 3] reduced from 3 to 0 for GPT-4, from 6 to 3 for Claude, and from 8 to 2 for Bard. In addition, the number of responses scoring (4, 5] increased from 19 to 38 for GPT-4, from 9 to 24 for Claude, and from 13 to 18 for Bard.

**Table 2:** Comprehensiveness evaluation for responses of large language models with and without RISE.

Score range	GPT-4		Claude 2		Google Bard	
	Without RISE^a^ (n=39)	With RISE (n=42)	Without RISE (n=31)	With RISE (n=39)	Without RISE (n=37)	With RISE (n=41)
(1, 2], n (%)	2 (5)	0 (0)	2 (7)	0 (0)	2 (5)	0 (0)
(2, 3], n (%)	1 (3)	0 (0)	4 (13)	3 (8)	6 (16)	2 (5)
(3, 4], n (%)	17 (44)	4 (10)	16 (52)	12 (31)	16 (43)	21 (51)
(4, 5], n (%)	19 (49)	38 (91)	9 (29)	24 (62)	13 (35)	18 (44)
Score, mean (SD)^b^	4.14 (0.72)	4.69 (0.39)	3.79 (0.78)	4.20 (0.60)	3.73 (0.80)	4.10 (0.62)

^a^RISE: Retrieval-augmented Information System for Enhancement.

^b^SD: Standard deviation. For responses rated as “good” by most graders, comprehensiveness was further evaluated.

Furthermore, the average scores for comprehensiveness also improved significantly after integrating RISE. GPT-4’s average score increased from 4.14 (SD 0.72) to 4.69 (SD 0.39; *P*<.001), Claude increased from 3.79 (SD 0.78) to 4.2 (SD 0.60; *P*=.002), and Bard increased from 3.73 (SD 0.80) to 4.10 (SD 0.62; *P*=.001). Among the 3 models, GPT-4 consistently achieved the highest scores for comprehensiveness both before and after the integration of RISE, with scores of 4.14 (SD 0.72) and 4.69 (SD 0.39), respectively.

### Understandability Evaluation

In addition to assessing the accuracy and comprehensiveness of model responses by clinicians, this study also evaluated the public’s understanding of responses (Table 3). A total of 3 diabetes patients rated the understandability on a scale of 1 to 5. The results indicated the integration of RISE led to a decrease in the number of responses with scores lower than 4 and an increase in the number of responses with scores of (4, 5]. Specifically, after incorporation of RISE, the number of responses scoring lower than 4 was reduced from 15 to 4 for GPT-4, 27 to 24 for Claude, and 31 to 21 for Bard. In addition, the number of responses with higher scores of (4, 5] increased from 18 to 39 for GPT-4, 16 to 19 for Claude, and 12 to 22 for Bard after the incorporation of RISE.

**Table 3:** Evaluation of public understandability in responses from large language models with and without RISE.

Score range	GPT-4		Claude 2			Google Bard	
	Without RISE^a^	With RISE	Without RISE	With RISE	Without RISE		With RISE
(1, 2], n (%)	0 (0)	0 (0)	0 (0)	0 (0)	0 (0)		0 (0)
(2, 3], n (%)	0 (0)	0 (0)	1 (2)	2 (5)	1 (2)		1 (2)
(3, 4], n (%)	15 (35)	4 (9)	26 (61)	22 (51)	30 (70)		20 (47)
(4, 5], n (%)	18 (65)	39 (91)	16 (37)	19 (44)	12 (28)		22 (51)
Score, mean (SD)^b^	4.32 (0.61)	4.64 (0.51)	4.01 (0.73)	4.07 (0.74)	3.96 (0.86)		4.16 (0.82)

^a^RISE: Retrieval-augmented Information System for Enhancement.

^b^SD: Standard deviation. An understandability evaluation was conducted for all responses.

Furthermore, the average scores for understandability also improved after RISE integration. GPT-4’s average score increased from 4.32 (SD 0.61) to 4.64 (SD 0.51; *P*<.001), Claude improved from 4.01 (SD 0.73) to 4.07 (SD 0.74; *P*=.31), and Bard elevated from 3.96 (SD 0.86) to 4.16 (SD 0.82; *P*=.002). Among the 3 models, GPT-4 consistently exhibited the highest understandability scores both before and after RISE integration, with scores of 4.32 (SD 0.61) and 4.64 (SD 0.51), respectively.

## Discussion

### Principal Findings

Considering the prevalence of type 2 diabetes mellitus as a major public health concern, particularly in light of the widespread dependence of patients on online resources for health-related information, this study introduces RISE workflow models to enhance the performance of LLMs as timely and relevant diabetes education tools [42,46,47]. Our findings demonstrate that RISE significantly improves the accuracy and comprehensiveness of LLM responses to patient queries about diabetes management and care. On average, the percentage of accurate responses increased by 12% (15/129) with RISE, with rates increasing by 7% (3/43) for GPT-4, 19% (8/43) for Claude 2, and 9% (4/43) for Google Bard. The framework also enhanced response comprehensiveness and understandability, improving mean scores by 0.44 (SD 0.10) and 0.19 (SD 0.13), respectively.

### Comparison to Previous Work

Previous studies have also applied LLMs in diabetes management and education. A study by Sun et al [7] found that 74.5% (149/200) of GPT-4’s answers accurately responded to 200 frequently asked questions on diabetes management education. Hernandez et al [48] showed ChatGPT could correctly answer 98.5% (69/70) of patient questions about type 2 diabetes, and the 1.5% (1/70) inappropriate response needs to be improved. These findings were consistent with our results before integrating RISE, showing 91% (39/43) accuracy for base GPT-4 in responding to diabetes questions. Although most information provided by advanced LLMs may be correct, it is essential to realize that even small mistakes can potentially cause significant problems, especially with medical scenarios. Even minimal misinformation can lead to misconceptions, which might inadvertently delay treatment. Thus, minimizing potential errors and improving accuracy and validation are required before considering LLMs integration into patient diabetes care.

RAG has shown promise in enhancing LLM performance [49,50], however, most current RAG approaches rely on fixed, smaller, static knowledge bases. Our results showed model responses were more specific and accurate than those generated by general LLMs after incorporating specific knowledge of the RISE framework, which is consistent with previous studies. Previous studies have applied RAG in other clinical specialties, such as general medicine, hepatology, and lymphoma [32,38,43,51]. These studies’ knowledge bases were mainly medical texts, research papers, and disease guidelines, limiting their flexibility and generalizability. In contrast, the RISE framework used a local medical knowledge base from NIH (National Institutes of Health) and the dynamic, real-time retrieval of external knowledge through the latest medical guidelines, academic research papers, and reputable health websites.

### Future Directions

The RISE framework demonstrates the potential of RAG in enhancing the performance of LLMs for diabetes education, and there are several promising directions for future research and development. These include creating large specialized medical knowledge bases tailored for diabetes education, integrating multimodal data such as medical images and electronic health records, and developing domain-specific retrieval and ranking algorithms for evidence-based information [52,53]. Furthermore, exploring the bilingual or multilingual potential of these chatbots, such as investigating the performance of the RISE framework when questions are asked in languages like Chinese, could expand their use in real-world clinical practice outside the English-speaking world. Another promising direction is exploring personalized RAG systems that adapt to individual preferences of patients and contexts. Ensuring RAG systems’ interpretability, transparency, privacy, and security is crucial in the medical domain.

### Strengths and Limitations

This study has several strengths. First, novel RAG algorithms that effectively use local databases and external academic knowledge markedly improve the precision and real-time performance of responses to diabetes-related inquiries. Second, the RISE framework incorporates rigorous factual and safety checks for the generated outputs, ensuring reliable and secure responses.

There are some limitations in the study. The RISE framework was developed and evaluated exclusively within the domain of diabetes. The generalizability of RISE to other medical domains remains uncertain. Future investigations should extend the application of the RISE framework to diverse medical specialties. Moreover, the scope of our evaluation was limited to these predetermined queries. Future research should conduct clinical trials to assess the RISE’s ability to effectively address inquiries of patients and enhance the efficiency of diabetes management in real-world clinical scenarios.

### Conclusions

In conclusion, the RISE framework shows promise as a safer and more reliable option for generating responses to common queries from diabetes patients. RISE significantly enhances the accuracy and comprehension of original LLM responses by retrieval of external knowledge from reliable sources. This framework can potentially be a supplementary tool to improve patient understanding and disease outcomes.

## Appendix

Multimedia Appendix 1 Code for the Retrieval-augmented Information System for Enhancement framework.

Multimedia Appendix 2 Sample of the Retrieval-augmented Information System for Enhancement Diabetes question and answer data set (n=50).

Multimedia Appendix 3 Responses for each model and raw scores for evaluation (clean).

## Abbreviations

FAISS: Facebook AI Similarity Search
LLMs: large language models
NIH: National Institutes of Health
RAG: Retrieval-augmented Generation
RISE: Retrieval-augmented Information System for Enhancement

## Data Availability

The complete data sets generated and analyzed during this study are available from the corresponding author on reasonable request.
