# Supplementary material for: Enhancement of the Performance of Large Language Models in Diabetes Education through Retrieval-Augmented Generation: Comparative Study
Source: J Med Internet Res. 2024 Nov 8;26:e58041. doi: 10.2196/58041 (PMC11584532; doi:10.2196/58041)
Supplement: Multimedia Appendix 3 [file jmir_v26i1e58041_app3.pdf]

Responses for each model and raw scores for evaluation

Contents

Response of Large Language Models

Supplementary Table 1a: GPT-4 Response to Diabetes-Related Questions 2

Supplementary Table 1b: GPT-4+RISE Response to Diabetes-Related Questions 45

Supplementary Table 1c: Claude Response to Diabetes-Related Questions 86

Supplementary Table 1d: Claude+RISE Response to Diabetes-Related Questions 119

Supplementary Table 1e: Bard Response to Diabetes-Related Questions 149

Supplementary Table 1f: Bard+RISE Response to Diabetes-Related Questions 193

Raw Score and Rating for Accuracy and Comprehensiveness of each Large Language Models

Supplementary Table 2a: Raw Score and Rating of each Base Large Language Models, Attained for Each Question 240

Supplementary Table 2b: Raw Score and Rating of each RISE-enhanced Large Language Models, Attained for Each Question 243

Raw Score for Understandability of each Large Language Models, Attained for Each Question

Supplementary Table 3: Raw Score for Understandability of each Large Language Models, Attained for Each Question 245

Supplementary Table 1a: GPT-4 Response to Diabetes-Related Questions

Question	GPT-4 Response
**Topic: Concepts of Diabetes**	
**1. What Is Diabetes?**	What Is Diabetes?<br><br>Diabetes is a chronic medical condition affecting the body's ability to regulate blood sugar (glucose) due to issues with the hormone insulin. Insulin, produced by the pancreas, helps cells absorb glucose from the bloodstream. When there's insufficient insulin or the body doesn't use it effectively, blood sugar levels rise.<br><br>Types of Diabetes:<br><br>Type 1: An autoimmune condition where the body attacks insulin-producing cells. Often diagnosed in youth.<br>Type 2: The most common form, where the body doesn't produce enough insulin or resists it. Typically seen in adults but can occur in youth.<br>Gestational: Arises during pregnancy due to increased insulin needs. Usually temporary but increases the risk of type 2 diabetes later.<br>Symptoms:<br>Include frequent urination, excessive thirst, weight loss, fatigue, blurred vision, and slow-healing sores.<br><br>Complications:<br>Unmanaged diabetes can lead to heart disease, stroke, kidney issues, eye problems, and nerve damage.<br><br>Management:<br>While incurable, diabetes can be controlled with medications, diet, exercise, and regular blood sugar monitoring.<br><br>Awareness of diabetes symptoms and risk factors is crucial for early detection and management, reducing potential complications.
**2. How can I determine which type of diabetes I have?**	Determining the Type of Diabetes You Have

	Diabetes is a chronic condition characterized by elevated blood sugar levels. There are primarily two main types: Type 1 and Type 2 diabetes. Determining the type you have is crucial for appropriate management.<br><br>Age of Onset:<br><br>Type 1 Diabetes often appears in childhood or adolescence, but it can occur at any age.<br>Type 2 Diabetes is more common in adults, although it's increasingly seen in younger individuals due to lifestyle factors.<br>Symptoms:<br><br>Type 1 Diabetes: Rapid onset of symptoms like excessive thirst, frequent urination, unexplained weight loss, and fatigue.<br>Type 2 Diabetes: Symptoms may be milder and develop over time. Some individuals might not even notice any symptoms initially.<br>C-Peptide Test: Measures the amount of C-peptide in your blood, which can indicate how much insulin your pancreas is producing. Low levels suggest Type 1, while normal or high levels typically indicate Type 2.<br><br>Autoantibody Testing: In Type 1 diabetes, the immune system mistakenly attacks insulin-producing cells. Testing for these autoantibodies can help confirm a Type 1 diagnosis.<br><br>Risk Factors:<br><br>Type 1: Family history, presence of other autoimmune conditions.<br>Type 2: Obesity, age, family history, sedentary lifestyle, history of gestational diabetes.<br>Management:<br><br>Type 1: Requires insulin injections or an insulin pump.<br>Type 2: Managed with lifestyle changes, oral medications, and sometimes insulin.

	It's essential to consult with a healthcare professional for a definitive diagnosis. They will consider your symptoms, medical history, and may order specific tests to determine the type of diabetes you have.
**3. Who is more likely to develop type 1 diabetes?**	Type 1 diabetes is an autoimmune condition where the body attacks its insulin-producing cells. Factors increasing the likelihood of developing type 1 diabetes include:<br><br>Genetics: A family history or certain genes can elevate the risk.<br>Age: It's more commonly diagnosed in children and young adults.<br>Geography: Higher incidence is observed farther from the equator, like in Northern Europe.<br>Viral Exposure: Viruses like enteroviruses and mumps might trigger the condition.<br>Early Diet: Introducing cow's milk or cereals before 4 months might elevate risk, though evidence isn't conclusive.<br>Vitamin D Deficiency: Essential for immune function, its deficiency might be linked to increased risk.<br>Autoantibodies: Individuals testing positive for diabetes-related autoantibodies are at higher risk.<br>While these factors heighten the risk, they don't guarantee onset. Conversely, absence of these factors doesn't ensure immunity. Regular check-ups and symptom awareness aid early detection and management. Immediate medical attention is advised if symptoms are observed.
**4. Who is more likely to develop type 2 diabetes?**	Type 2 diabetes is a complex metabolic disorder influenced by a combination of genetic, environmental, and lifestyle factors. While anyone can develop type 2 diabetes, certain groups and individuals with specific characteristics are at a higher risk:<br><br>Age: Older adults, typically those over 45, are more likely to develop type 2 diabetes. However, in recent years, there's been a concerning rise in younger populations, including children, adolescents, and young adults.<br><br>Family History: Individuals with a parent or sibling with type 2 diabetes have a higher likelihood of developing the condition.<br><br>Ethnicity: Certain ethnic groups, including African Americans, Hispanic/Latino Americans, American Indians, and some Asian Americans and Pacific Islanders, are at a greater risk.

	Obesity: Being overweight, especially with central obesity (excess belly fat), significantly increases the risk. Fat distribution and the way the body stores fat play a role in insulin resistance.<br><br>Physical Inactivity: Sedentary lifestyles contribute to weight gain and insulin resistance, elevating the risk of type 2 diabetes.<br><br>Poor Diet: Diets high in processed foods, sugars, and unhealthy fats, but low in fiber and whole grains, can increase the risk.<br><br>Gestational Diabetes: Women who had diabetes during pregnancy or delivered a baby weighing more than 9 pounds are at an increased risk.<br><br>Polycystic Ovary Syndrome (PCOS): Women with PCOS have a higher risk due to insulin resistance.<br><br>Other Medical Conditions: Conditions like high blood pressure and abnormal cholesterol levels can increase the risk. Having prediabetes, where blood sugar levels are higher than normal but not yet in the diabetic range, is a significant risk factor.<br><br>Smoking: Smokers are 30-40% more likely to develop type 2 diabetes than non-smokers.<br><br>It's essential to recognize that while these factors increase risk, they don't guarantee someone will develop diabetes. Regular check-ups, a balanced diet, physical activity, and maintaining a healthy weight can significantly reduce the risk.
**Topic: Symptoms & Causes**	
**1. What are the symptoms of diabetes?**	I'm not a human expert, but I can provide information based on the knowledge I've been trained on. Diabetes is a chronic condition that affects the body's ability to process sugar. The symptoms of diabetes can vary depending on the type of diabetes and the individual's specific circumstances. Here are some common symptoms associated with diabetes:<br><br>Frequent urination: People with diabetes may need to urinate more often than usual, especially at night.<br><br>Increased thirst: Along with frequent urination, there might be an unquenchable thirst.

	Extreme hunger: Despite eating regularly, there might be a persistent feeling of hunger.<br><br>Unexplained weight loss: Even though one might be eating more than usual, there can be a sudden and unexplained loss of weight.<br><br>Fatigue: Feeling tired and lethargic throughout the day is common.<br><br>Blurred vision: High blood sugar levels can lead to blurred vision.<br><br>Slow-healing sores or frequent infections: Diabetes can affect the body's ability to heal and resist infections.<br><br>Tingling, pain, or numbness in the hands or feet: This is often a sign of nerve damage, which can be caused by prolonged high blood sugar levels.<br><br>Darkened skin in certain areas: This is known as acanthosis nigricans and is often found in the armpits and neck.<br><br>Presence of ketones in the urine: This is a result of the body breaking down muscle and fat for energy when it doesn't have enough insulin.<br><br>It's important to note that Type 1 and Type 2 diabetes might have overlapping symptoms, but they can also have some distinct signs. For example, Type 1 diabetes symptoms can come on quickly and be more severe.<br><br>If someone suspects they have diabetes or is experiencing any of these symptoms, it's crucial to see a healthcare professional for a proper diagnosis and treatment. Regular monitoring and management can help prevent complications associated with diabetes.
**2. What causes type 1 diabetes?**	I'm not a human expert, but I can provide information based on existing knowledge. Type 1 diabetes is an autoimmune condition, and its exact cause is not fully understood. However, the following are commonly accepted explanations for the development of type 1 diabetes:

	Autoimmune Reaction: Type 1 diabetes is characterized by the body's immune system mistakenly attacking and destroying insulin-producing beta cells in the pancreas. This autoimmune reaction means the body can no longer produce sufficient insulin.<br><br>Genetic Factors: Certain genes can increase the risk of developing type 1 diabetes. However, having these genes doesn't guarantee the onset of the disease; it just increases susceptibility.<br><br>Environmental Factors: Some researchers believe that exposure to certain environmental factors, such as viruses, might trigger the autoimmune response in genetically susceptible individuals.<br><br>Viral Infections: Some viruses, like the coxsackievirus, mumps, and rubella, have been linked to the onset of type 1 diabetes. It's believed that these viruses might trigger the autoimmune destruction of beta cells or directly infect the beta cells, leading to the disease.<br><br>Other Factors: Dietary factors, early exposure to cow's milk, and a lack of vitamin D have also been studied as potential triggers, but the evidence is not conclusive.<br><br>It's important to note that type 1 diabetes is not caused by diet or lifestyle. It's a chronic condition that usually appears in childhood or adolescence, although it can develop at any age.<br><br>If you or someone you know is concerned about diabetes, it's essential to consult with a healthcare professional for a comprehensive understanding and diagnosis.
**3. What causes type 2 diabetes?**	What Causes Type 2 Diabetes?<br><br>Type 2 diabetes is a chronic condition that affects the way the body processes blood sugar (glucose). While the exact cause of type 2 diabetes is multifaceted and not completely understood, several factors are believed to contribute:<br><br>Insulin Resistance: In the early stages of type 2 diabetes, the body becomes resistant to the effects of insulin, a hormone that helps glucose enter cells. As a result, the body needs more insulin to help glucose enter cells. Over time, the pancreas cannot produce enough insulin to keep blood glucose levels normal.

	Genetics: Family history can play a role. If you have a parent or sibling with type 2 diabetes, you're at a higher risk of developing the condition.<br><br>Obesity: Being overweight is a primary risk factor. Fat, especially abdominal fat, releases chemicals that can destabilize the body's metabolic and cardiovascular systems, leading to insulin resistance.<br><br>Poor Diet: Consuming foods high in refined sugars, unhealthy fats, and calories can lead to obesity and insulin resistance. A diet low in fiber and high in processed foods can increase the risk.<br><br>Physical Inactivity: A sedentary lifestyle can lead to weight gain and insulin resistance. Regular physical activity helps maintain a healthy weight and makes cells more sensitive to insulin.<br><br>Age: The risk increases as you get older, especially after age 45. However, type 2 diabetes is also increasing among children and adolescents.<br><br>Hormonal Conditions: Conditions like polycystic ovary syndrome (PCOS) can increase the risk.<br><br>Gestational Diabetes: Women who had diabetes during pregnancy (gestational diabetes) or who delivered a baby weighing more than 9 pounds are at an increased risk.<br><br>Other Factors: High blood pressure, abnormal cholesterol levels, and a history of heart disease can also contribute.<br><br>It's essential to understand that while these factors increase the risk, they don't guarantee that one will develop type 2 diabetes. Lifestyle choices, such as a balanced diet and regular exercise, can play a significant role in prevention.
**4. What causes gestational diabetes?**	Gestational diabetes is a form of diabetes that occurs during pregnancy. While the exact cause is not fully understood, several factors contribute to its development:<br><br>Hormonal Changes: Pregnancy results in the production of various hormones that help the baby grow and develop. Some of these hormones can make cells more resistant to insulin, a hormone that regulates blood sugar. As the pregnancy progresses, the amount of these hormones increases, leading to higher insulin resistance.

	Pancreatic Limitations: The pancreas produces insulin, and in response to the increased insulin resistance during pregnancy, it needs to produce more insulin. If the pancreas can't produce enough insulin to overcome the resistance, blood sugar levels rise, leading to gestational diabetes.<br><br>Increased Sugar in the Body: During pregnancy, the body might not be able to break down and use sugar as efficiently. This can lead to sugar accumulating in the bloodstream.<br><br>Risk Factors: While any pregnant woman can develop gestational diabetes, certain risk factors increase the likelihood. These include being over the age of 25, having a family history of diabetes, being overweight, and having had gestational diabetes in a previous pregnancy. Additionally, women from certain ethnic groups, such as African American, Hispanic, Native American, and Asian American, are at a higher risk.<br><br>It's essential to understand that gestational diabetes doesn't mean a woman had diabetes before pregnancy or will have it afterward. However, it does increase the risk of developing type 2 diabetes later in life. Regular screening and maintaining a healthy lifestyle can help manage and prevent complications associated with gestational diabetes.
**Topic: Diabetes Tests & Diagnosis**	
**1. Who should be tested for diabetes?**	Who Should Be Tested for Diabetes?<br><br>Diabetes is a chronic condition that affects the body's ability to process sugar. Early detection is crucial to manage the disease and prevent complications. The following groups of people should consider getting tested for diabetes:<br><br>Age Factor: Adults aged 45 and older should be tested for diabetes, especially if they are overweight or obese. If the results are normal, retesting should be done at least every three years.<br><br>Overweight and Obesity: Individuals of any age who are overweight or obese and have one or more of the following risk factors should be tested:<br><br>Family history of diabetes.

	High blood pressure.<br>Low HDL cholesterol or high triglycerides.<br>History of gestational diabetes or giving birth to a baby weighing more than 9 pounds.<br>Polycystic ovary syndrome (PCOS).<br>Ethnicity: People of certain racial and ethnic groups, including African Americans, Hispanic/Latino Americans, Native Americans, Asian Americans, and Pacific Islanders, are at a higher risk.<br><br>History of Heart Disease: Individuals with a history of heart disease or stroke should be screened.<br><br>Physical Inactivity: Sedentary individuals are at an increased risk.<br><br>Signs and Symptoms: Anyone experiencing symptoms of diabetes, such as frequent urination, excessive thirst, unexplained weight loss, fatigue, blurred vision, or slow-healing sores, should get tested.<br><br>Gestational Diabetes: Women who were diagnosed with gestational diabetes during pregnancy should be tested every three years.<br><br>Other Conditions: Individuals with conditions like polycystic ovary syndrome or those showing signs of insulin resistance should be screened.<br><br>Early detection and management of diabetes can prevent complications and improve the quality of life. If you fall into any of the above categories or have concerns about your risk, consult with a healthcare professional about getting tested.
**2. What tests are used to diagnose diabetes?**	Tests Used to Diagnose Diabetes:<br><br>Diabetes is a chronic condition characterized by elevated levels of blood glucose (sugar). Early diagnosis is crucial to manage the disease and prevent complications. Several tests are used to diagnose diabetes:

	Fasting Blood Sugar Test (FBG): This test measures the blood sugar level after an overnight fast. A fasting blood sugar level of 126 milligrams per deciliter (mg/dL) or higher indicates diabetes.<br><br>Oral Glucose Tolerance Test (OGTT): After fasting, a person consumes a sugary solution, and blood sugar levels are tested at intervals. A reading of 200 mg/dL or higher two hours after drinking the solution indicates diabetes.<br><br>Hemoglobin A1c Test: This test provides an average of blood sugar levels over the past 2 to 3 months. An A1c level of 6.5% or higher is diagnostic for diabetes.<br><br>Random Blood Sugar Test: A blood sample is taken at a random time, regardless of when the individual last ate. A blood sugar level of 200 mg/dL or higher suggests diabetes.<br><br>C-Peptide Test: This test measures the amount of C-peptide in the blood, which can indicate how much insulin the pancreas is producing. It's used to differentiate between type 1 and type 2 diabetes.<br><br>Autoantibodies Test: This test checks for the presence of autoantibodies that are often present in type 1 diabetes. It helps distinguish between type 1 and type 2 diabetes.<br><br>It's essential to note that one test result alone is not enough for a diagnosis. Doctors often repeat tests or use multiple tests to confirm a diabetes diagnosis. If you suspect you have diabetes or are at risk, it's crucial to consult with a healthcare professional for appropriate testing and diagnosis.
**3. What test results tell me if I have diabetes or prediabetes?**	Diagnosing Diabetes and Prediabetes<br><br>To determine if an individual has diabetes or prediabetes, healthcare professionals rely on specific tests. The most common tests include:<br><br>Fasting Blood Sugar (FBS) Test: This test measures blood sugar levels after an overnight fast.<br><br>Normal: Below 100 mg/dL

	Prediabetes: 100-125 mg/dL<br>Diabetes: 126 mg/dL or higher<br>Oral Glucose Tolerance Test (OGTT): After fasting, a person consumes a drink containing glucose, and blood sugar levels are tested at intervals.<br><br>Normal: Below 140 mg/dL<br>Prediabetes: 140-199 mg/dL<br>Diabetes: 200 mg/dL or higher<br>Hemoglobin A1c Test: This test provides an average blood sugar level over the past 2-3 months.<br><br>Normal: Below 5.7%<br>Prediabetes: 5.7% to 6.4%<br>Diabetes: 6.5% or higher<br>Random Blood Sugar Test: A blood sample is taken at a random time, regardless of when the person last ate.<br><br>Diabetes: 200 mg/dL or higher (along with symptoms of diabetes)<br>It's essential to note that a single test result indicating high blood sugar does not confirm diabetes. Typically, the test is repeated on another day to verify the diagnosis. Additionally, other conditions and medications can affect blood sugar levels, so it's crucial to discuss any abnormal results with a healthcare professional.
**4. Which tests help my doctor know what kind of diabetes I have?**	Which tests help my doctor know what kind of diabetes I have?<br><br>To determine the type of diabetes a person has, doctors employ a combination of blood tests. These tests not only diagnose diabetes but also help distinguish between its various types:<br><br>Fasting Blood Sugar Test (FBST): Measures blood sugar after an overnight fast. A level of 126 milligrams per deciliter (mg/dL) or higher indicates diabetes.<br><br>Oral Glucose Tolerance Test (OGTT): After fasting, you'll drink a sugary solution, and your blood sugar levels will be tested at intervals. A 2-hour blood sugar level of 200 mg/dL or higher suggests diabetes.

	Hemoglobin A1c Test: Indicates average blood sugar levels over the past 2-3 months. An A1c level of 6.5% or higher confirms diabetes.<br><br>C-Peptide Test: Measures the amount of C-peptide, a byproduct of insulin production, in the blood. Lower levels might indicate type 1 diabetes, while higher or normal levels suggest type 2 diabetes.<br><br>Autoantibodies Test: People with type 1 diabetes often have specific autoantibodies in their blood, which are absent in those with type 2 diabetes. This test can be crucial in distinguishing between the two types.<br><br>Random Blood Sugar Test: A blood sample is taken at a random time, regardless of when you last ate. A blood sugar level of 200 mg/dL or higher suggests diabetes.<br><br>It's essential to note that while these tests provide valuable information, the diagnosis and classification of diabetes type are based on a comprehensive clinical assessment. Factors like age, presentation, body weight, and family history can also influence the determination of diabetes type. Always consult with a healthcare professional for an accurate diagnosis.
**Topic: Managing Diabetes**	
**1. How can I manage my diabetes?**	Managing Diabetes: A Brief Guide<br><br>Diabetes is a chronic condition that affects the body's ability to process sugar. Proper management is crucial to prevent complications and maintain a good quality of life. Here are some key steps to manage diabetes effectively:<br><br>Monitor Blood Sugar Levels: Regularly check your blood glucose levels using a home glucose monitor. This helps in understanding how different factors like food, activity, and stress affect your levels.<br><br>Healthy Diet: Focus on a balanced diet rich in whole grains, lean proteins, healthy fats, and plenty of fruits and vegetables. Limit the intake of sugary foods and drinks. Consider consulting a nutritionist to create a meal plan tailored to your needs.

	Regular Exercise: Engage in at least 150 minutes of moderate-intensity aerobic activity every week, such as walking or cycling. Exercise helps improve insulin sensitivity and control blood sugar levels.<br><br>Medication: If prescribed, take diabetes medications or insulin as directed. Never skip doses without consulting your healthcare provider.<br><br>Regular Check-ups: Visit your healthcare provider regularly for check-ups. This helps in early detection and management of potential complications.<br><br>Manage Stress: Chronic stress can elevate blood sugar levels. Techniques like meditation, deep breathing, and yoga can help manage stress.<br><br>Avoid Smoking and Limit Alcohol: Smoking can worsen diabetes-related complications, while excessive alcohol can lead to unpredictable changes in blood sugar.<br><br>Stay Educated: Stay updated about diabetes and its management. The more you know, the better equipped you'll be to handle challenges.<br><br>Seek Support: Join diabetes support groups or online communities. Sharing experiences and learning from others can be beneficial.<br><br>Remember, every individual is different, so what works for one person might not work for another. It's essential to work closely with your healthcare provider to develop a personalized plan that suits your needs.
**2. What are the recommended targets for blood glucose levels?**	Recommended Targets for Blood Glucose Levels in Diabetes Management:<br><br>Managing blood glucose levels is crucial for individuals with diabetes. The recommended targets can vary based on individual health conditions, age, and the presence of other risk factors. However, general guidelines are as follows:<br><br>Fasting Blood Glucose (FBG):<br><br>Target: 70-130 mg/dL (3.9-7.2 mmol/L)<br>This measures blood sugar after an overnight fast and before the first meal of the day.

	Postprandial Blood Glucose (PPBG):<br><br>Target: Less than 180 mg/dL (10 mmol/L)<br>This measures blood sugar 1-2 hours after meals.<br>Hemoglobin A1c (HbA1c):<br><br>Target for most adults: Less than 7%<br>HbA1c provides an average blood sugar level over the past 2-3 months. A lower percentage indicates better blood sugar control.<br>It's essential to note that these targets might be adjusted based on individual needs. For instance, older adults or those with certain medical conditions might have slightly relaxed targets. Conversely, tighter control might be recommended for younger individuals or those without complications.<br><br>Regular monitoring and consultation with healthcare professionals are vital. They can provide personalized guidance and adjust targets as necessary to ensure optimal health and prevent complications.
**3. What medicines might I take for diabetes?**	Medicines for Diabetes:<br><br>Diabetes is a chronic condition that affects the body's ability to regulate blood sugar levels. The type of medication prescribed for diabetes largely depends on the type of diabetes one has. Here are some common medications used to treat diabetes:<br><br>Insulin: This is a hormone that helps regulate blood sugar. There are various types of insulin, including rapid-acting, short-acting, intermediate-acting, and long-acting. It's often administered via injections or insulin pumps.<br><br>Metformin (Glucophage, Glumetza, others): This is a first-line medication for type 2 diabetes. It works by improving the sensitivity of your body tissues to insulin and reducing liver glucose production.<br><br>Sulfonylureas: These medications help your body secrete more insulin. Examples include glipizide (Glucotrol), glyburide (DiaBeta, Glynase), and glimepiride (Amaryl).

	DPP-4 inhibitors: These medications lower blood sugar levels by blocking the action of an enzyme called DPP-4. Examples are sitagliptin (Januvia) and linagliptin (Tradjenta).<br><br>GLP-1 receptor agonists: These are injectable medications that slow digestion and help lower blood sugar levels. Examples include exenatide (Byetta) and liraglutide (Victoza).<br><br>SGLT2 inhibitors: These drugs prevent the kidneys from reabsorbing sugar into the blood, allowing it to be excreted in the urine. Examples are canagliflozin (Invokana) and dapagliflozin (Farxiga).<br><br>Thiazolidinediones: Like metformin, these medications make the body's tissues more sensitive to insulin. Examples are rosiglitazone (Avandia) and pioglitazone (Actos).<br><br>It's essential to note that the choice of medication depends on individual health conditions, potential drug side effects, and other factors. Regular monitoring and consultation with a healthcare professional are crucial to ensure optimal diabetes management.
**4. What are the different types of insulin?**	Insulin is a hormone essential for regulating blood sugar levels. For individuals with diabetes, external insulin may be required to manage their condition. There are several types of insulin, each with its own onset, peak, and duration of action:<br><br>Rapid-Acting Insulin:<br><br>Examples: Lispro (Humalog), Aspart (NovoLog), and Glulisine (Apidra).<br>Onset: Begins working within 15 minutes.<br>Peak: 1-2 hours.<br>Duration: 3-5 hours.<br>Short-Acting Insulin:

	Example: Regular or R insulin (Humulin R, Novolin R).<br>Onset: Takes effect within 30 minutes.<br>Peak: 2-3 hours.<br>Duration: 6-8 hours.<br>Intermediate-Acting Insulin:<br><br>Example: NPH or Isophane insulin (Humulin N, Novolin N).<br>Onset: Starts working in 1-2 hours.<br>Peak: 4-12 hours.<br>Duration: 14-24 hours.<br>Long-Acting Insulin:<br><br>Examples: Glargine (Lantus, Toujeo), Detemir (Levemir), and Degludec (Tresiba).<br>Onset: Begins in 1-2 hours.<br>Peak: Minimal or no pronounced peak.<br>Duration: Up to 24 hours or longer.<br>Ultra-Long-Acting Insulin:<br><br>Example: Degludec (Tresiba).<br>Onset: 1 hour.<br>Peak: Almost no peak.<br>Duration: Up to 42 hours.<br>Premixed Insulin:<br><br>Combines specific proportions of intermediate-acting and short- or rapid-acting insulin.<br>Examples: NovoLog Mix 70/30, Humalog Mix 75/25.

	It's crucial for individuals with diabetes to work closely with their healthcare providers to determine the most appropriate insulin type and regimen for their specific needs.
**5. What are the different ways to take insulin?**	Insulin is a crucial hormone for regulating blood sugar levels, and for people with certain types of diabetes, external insulin administration becomes necessary. There are several ways to take insulin:<br><br>Insulin Injections: This is the most common method. Using a fine needle and syringe, insulin is injected into the fatty tissue just under the skin. The injection sites can be the abdomen, thighs, buttocks, or the back of the upper arms.<br><br>Insulin Pens: These are devices that look like regular pens but contain a cartridge of insulin. They are more convenient than syringes, especially for people on the go. The pen allows you to set the dose by turning a dial, making it easier to administer the correct amount.<br><br>Insulin Pumps: These are small devices worn outside the body and deliver a continuous supply of insulin through a tiny flexible tube that's inserted under the skin. They can be programmed to release varying amounts of insulin at different times, mimicking the body's natural release of the hormone.<br><br>Inhalable Insulin: A relatively newer method, this involves inhaling insulin powder through a device, much like an asthma inhaler. It gets absorbed into the bloodstream through the lungs.<br><br>Insulin Jet Injectors: These devices send a fine spray of insulin through the skin using high-pressure air instead of a needle.<br><br>Insulin Patches: Still in development, these would allow for the continuous release of insulin through the skin without the need for injections.<br><br>It's essential for individuals with diabetes to work closely with their healthcare providers to determine the best insulin delivery method for their needs. Regular monitoring and adjustments are crucial for optimal blood sugar control.
**6. What oral medicines treat type 2 diabetes?**	Oral Medications for Type 2 Diabetes:

	Type 2 diabetes is a chronic condition that affects the way the body processes blood sugar (glucose). Several oral medications are available to help manage blood sugar levels in people with type 2 diabetes. These include:<br><br>Biguanides (Metformin): The most commonly prescribed drug for type 2 diabetes. It works by decreasing the amount of glucose produced by the liver and improving the sensitivity of muscle cells to insulin.<br><br>Sulfonylureas: These drugs stimulate the pancreas to produce more insulin. Examples include glipizide, glyburide, and glimepiride.<br><br>Thiazolidinediones (TZDs): These make the body's tissues more sensitive to insulin. Examples are pioglitazone and rosiglitazone.<br><br>DPP-4 Inhibitors: These reduce blood sugar levels by affecting the breakdown of a naturally occurring compound in the body. Sitagliptin and linagliptin are examples.<br><br>SGLT2 Inhibitors: These drugs prevent the kidneys from reabsorbing glucose, leading to excretion of glucose in the urine. Examples include canagliflozin, dapagliflozin, and empagliflozin.<br><br>Alpha-Glucosidase Inhibitors: These slow the breakdown of starches in the gut, slowing the rise in blood sugar levels after meals. Acarbose is an example.<br><br>Bile Acid Sequestrants: Though primarily used to lower cholesterol, they also help reduce blood sugar levels. Colesevelam is an example.<br><br>It's essential for individuals to work closely with their healthcare provider to determine the best medication or combination of medications for their specific needs. Regular monitoring of blood sugar levels, a balanced diet, and physical activity are also crucial components of effective diabetes management.
**7. What should I know about side effects of diabetes medicines?**	Side Effects of Diabetes Medicines: What You Should Know<br><br>Diabetes medicines are crucial for managing blood sugar levels and preventing complications. However, like all medications, they can have side effects. It's essential to be aware of these potential side effects and discuss any concerns with your healthcare provider.

	Oral Medications:<br><br>Metformin: The most common side effects include stomach upset, diarrhea, and metallic taste. These usually decrease over time.<br>Sulfonylureas (e.g., glipizide, glyburide): They can cause low blood sugar (hypoglycemia), weight gain, and skin rash.<br>DPP-4 inhibitors (e.g., sitagliptin): They might lead to joint pain and a slight risk of pancreatitis.<br>Insulin:<br><br>Using insulin can sometimes result in hypoglycemia, especially if meals are skipped or there's increased physical activity. Weight gain, swelling, and injection site reactions are other potential side effects.<br>GLP-1 Receptor Agonists (e.g., liraglutide):<br><br>These can cause nausea, vomiting, and diarrhea. There's also a minimal risk of pancreatitis.<br>SGLT2 Inhibitors (e.g., canagliflozin):<br><br>They might lead to urinary tract infections, yeast infections, and increased urination. Rarely, they can cause diabetic ketoacidosis, a serious condition.<br>Thiazolidinediones (e.g., pioglitazone):<br><br>Potential side effects include weight gain, swelling, and an increased risk of heart failure.<br>Key Takeaways:<br><br>Always take medications as prescribed.<br>Monitor for signs of low blood sugar, such as dizziness, sweating, and confusion.<br>Report any unusual or severe side effects to your doctor.<br>Regularly review your medication regimen with your healthcare provider to ensure it's effective and safe.

**8. What foods can I eat if I have diabetes?**	If you have diabetes, it's essential to focus on foods that help regulate blood sugar levels and support overall health. Here are some recommended foods:<br><br>Whole Grains: Choose whole grains like quinoa, barley, brown rice, and oats. They have a lower glycemic index and provide steady energy.<br><br>Leafy Greens: Spinach, kale, and chard are low in calories and carbohydrates, making them excellent choices.<br><br>Fatty Fish: Salmon, mackerel, sardines, and trout are rich in omega-3 fatty acids, which can reduce inflammation and lower the risk of heart disease.<br><br>Nuts and Seeds: Almonds, walnuts, flaxseeds, and chia seeds are nutrient-dense and can help stabilize blood sugar.<br><br>Legumes: Beans, lentils, and chickpeas are high in fiber and protein, helping to maintain steady blood sugar levels.<br><br>Berries: Blueberries, strawberries, and raspberries are packed with antioxidants and are lower in sugar than many fruits.<br><br>Avocado: High in healthy fats and fiber, avocados can improve cholesterol levels and reduce the risk of heart disease.<br><br>Dairy or Dairy Alternatives: Greek yogurt and almond or soy milk can be good choices, but always opt for unsweetened versions.<br><br>Lean Proteins: Chicken, turkey, tofu, and eggs are excellent protein sources that won't spike your blood sugar.<br><br>Healthy Fats: Olive oil, avocados, and nuts contain fats that are beneficial for heart health.<br><br>Avoid or limit sugary foods and beverages, processed snacks, and fried foods. It's also essential to monitor portion sizes and consult with a nutritionist or healthcare provider to create a meal plan tailored to your needs.
**9. What foods and drinks should I limit if I have diabetes?**	If you have diabetes, it's essential to monitor your food and drink intake to manage blood sugar levels effectively. Here are some foods and drinks you should limit:

	1. Sugary Beverages: Avoid sodas, sweetened teas, fruit drinks with added sugars, and energy drinks. These can cause rapid spikes in blood sugar.<br><br>2. Trans Fats: Found in margarines, peanut butter, spreads, and some frozen dinners. They're linked to insulin resistance and heart disease.<br><br>3. White Bread, Rice, and Pasta: These refined carbohydrates can lead to rapid blood sugar spikes. Opt for whole grains instead.<br><br>4. Fruit-Flavored Yogurt: Often packed with sugar. Choose plain yogurt and add fresh fruit for flavor.<br><br>5. Sweetened Breakfast Cereals: Go for whole-grain, low-sugar varieties.<br><br>6. Flavored Coffee Drinks: Some of these beverages contain an alarming amount of sugar. A simple coffee with milk or a sugar substitute is a better choice.<br><br>7. Honey, Agave Nectar, and Maple Syrup: Even though they're natural, they can still raise blood sugar levels.<br><br>8. Dried Fruit: Contains concentrated sugar. Fresh or frozen fruit is preferable.<br><br>9. Packaged Snack Foods: Especially those high in sugar, unhealthy fats, and refined flour.<br><br>10. Alcohol: Can lead to blood sugar fluctuations. If consumed, do so in moderation and always with a meal.<br><br>11. Fried Foods: High in unhealthy fats and can promote inflammation.<br><br>12. High-Fat Dairy Products: Opt for low-fat versions to reduce saturated fat intake.<br><br>In summary, focus on whole, unprocessed foods and monitor carbohydrate intake. Always consult with a healthcare professional or nutritionist to create a meal plan tailored to your needs.

**10. When should I eat if I have diabetes?**	When Should I Eat If I Have Diabetes?<br><br>If you have diabetes, maintaining a consistent eating schedule is crucial for stabilizing blood sugar levels. Here are some general guidelines:<br><br>Regular Meal Times: Aim to eat meals at the same times every day. This helps your body predict and manage blood sugar fluctuations. For many, this means breakfast, lunch, and dinner spaced evenly throughout the day.<br><br>Avoid Skipping Meals: Skipping meals can lead to unpredictable blood sugar spikes or drops. If you're on medication or insulin, it's especially important to eat regularly to prevent hypoglycemia (low blood sugar).<br><br>Snacking: Depending on your blood sugar levels and medications, you might benefit from healthy snacks between meals. Snacks can prevent blood sugar levels from dropping too low and provide sustained energy. Opt for snacks that combine protein, healthy fats, and complex carbohydrates, like nuts or whole grain crackers with cheese.<br><br>Monitor Blood Sugar: Everyone's body is different. Use a blood glucose meter to monitor your levels. This will help you understand how different foods and meal timings affect you. Share this data with your healthcare provider to make any necessary adjustments.<br><br>Night-time Eating: If you take insulin or certain diabetes medications, be cautious about eating large amounts right before bed. This can increase the risk of nighttime hypoglycemia. A small, balanced snack might be recommended if you're prone to low blood sugar at night.<br><br>Consult Your Healthcare Provider: These are general guidelines, and individual needs can vary. It's essential to work closely with your doctor or a registered dietitian to create a meal plan tailored to your specific needs.
**11. How much can I eat if I have diabetes?**	How Much Can I Eat If I Have Diabetes?<br><br>If you have diabetes, it's essential to understand that it's not just about limiting sugar, but managing your overall diet to maintain stable blood sugar levels. Here's a general guideline:

	Carbohydrates: They have the most significant impact on blood sugar. Monitor your intake of foods like bread, rice, pasta, fruits, and sweets. Consider following the "plate method" – half of your plate should be non-starchy vegetables, a quarter should be lean protein, and the remaining quarter can be whole grains or starchy foods.<br><br>Proteins: Lean proteins like chicken, turkey, fish, tofu, and legumes are excellent choices. They have minimal impact on blood sugar when consumed in moderation.<br><br>Fats: Healthy fats from avocados, nuts, seeds, and olive oil can be beneficial. However, limit saturated and trans fats found in fried foods, baked goods, and certain meats.<br><br>Fiber: High-fiber foods like whole grains, legumes, fruits, and vegetables can help stabilize blood sugar levels.<br><br>Portion Control: This is crucial. Even healthy foods can raise blood sugar if consumed in large amounts. Use measuring cups or a food scale to ensure you're eating the recommended portion sizes.<br><br>Regular Meals: Eat regular meals and snacks throughout the day to prevent significant blood sugar fluctuations. Aim for consistency in your meal timings and portion sizes.<br><br>Monitor Blood Sugar: Everyone's body reacts differently to foods. Regularly checking your blood sugar levels can help you understand how different foods affect you.<br><br>Consultation: Always consult with a registered dietitian or diabetes educator to create a personalized meal plan tailored to your needs.<br><br>Remember, managing diabetes is about balance. It's not about depriving yourself but making informed choices to enjoy a diverse and nutritious diet while maintaining stable blood sugar levels.

**12. Will supplements and vitamins help my diabetes?**	Will Supplements and Vitamins Help My Diabetes?<br><br>Diabetes is a complex metabolic disorder that affects how the body processes glucose. The role of supplements and vitamins in diabetes management has been a topic of interest and research.<br><br>Vitamin D: Some studies suggest that people with low levels of vitamin D might be at a greater risk for type 2 diabetes. Supplementing with vitamin D may improve blood sugar control in some people, but the evidence is not consistent.<br><br>Chromium: Chromium is an essential trace element. Some research indicates that it might improve insulin sensitivity and blood sugar control. However, the results are mixed, and more research is needed.<br><br>Magnesium: Low magnesium levels are often seen in people with type 2 diabetes. Some studies suggest that magnesium supplementation might improve insulin function and lower blood sugar levels.<br><br>Alpha-lipoic acid: This antioxidant has been researched for its potential effects on improving insulin sensitivity and reducing symptoms of diabetic neuropathy. However, more studies are required to confirm its benefits.<br><br>Omega-3 fatty acids: While they don't directly impact blood sugar levels, omega-3s can reduce the risk of heart disease, a common complication of diabetes.<br><br>Cinnamon: Some small studies have found that cinnamon might help improve blood sugar levels, but the evidence is not strong enough to recommend it as a treatment.<br><br>Conclusion: While certain supplements and vitamins may offer benefits for people with diabetes, they should not replace standard diabetes treatments. It's essential to consult with a healthcare professional before starting any new supplement, as some can interact with medications or affect blood sugar levels. A balanced diet, regular exercise, and adherence to prescribed medications remain the cornerstone of diabetes management.

**13. Why should I be physically active if I have diabetes?**	Why Physical Activity is Essential for People with Diabetes:<br><br>Diabetes is a condition where the body either doesn't produce enough insulin or doesn't use it effectively, leading to elevated blood sugar levels. Regular physical activity offers multiple benefits for individuals with diabetes:<br><br>Blood Sugar Control: Exercise helps muscles use glucose more efficiently, reducing blood sugar levels. Over time, consistent physical activity can lead to a decrease in the overall need for insulin or oral diabetic medications.<br><br>Weight Management: Being overweight is a risk factor for type 2 diabetes. Physical activity helps burn calories and reduce body weight, which can improve insulin sensitivity.<br><br>Heart Health: Diabetes increases the risk of heart disease. Regular exercise strengthens the heart, lowers bad cholesterol, and raises good cholesterol, reducing the risk of cardiovascular complications.<br><br>Blood Pressure Regulation: High blood pressure is common among people with diabetes. Exercise helps lower blood pressure, reducing strain on the heart and the risk of stroke.<br><br>Improved Circulation: Exercise promotes better blood flow, reducing the risk of peripheral artery disease and neuropathy, common complications in diabetes.<br><br>Mood Enhancement: Physical activity releases endorphins, the body's natural mood elevators. This can help combat depression and anxiety, which are more prevalent in people with chronic conditions like diabetes.<br><br>Prevention of Complications: Regular exercise can prevent or delay diabetes-related complications such as kidney disease, eye problems, and nerve damage.<br><br>In conclusion, being physically active is not just about managing diabetes but also about enhancing overall well-being. It's essential to consult with a healthcare professional before starting any exercise regimen to ensure it's safe and tailored to individual needs.

**14. How can I be physically active safely if I have diabetes?**	Being Physically Active Safely with Diabetes<br><br>Physical activity is essential for everyone, including those with diabetes. It helps control blood sugar levels, reduces the risk of heart disease, and promotes overall well-being. However, if you have diabetes, there are some precautions to consider:<br><br>Consult Your Doctor: Before starting any exercise regimen, always consult with your healthcare provider. They can provide guidance tailored to your specific condition and needs.<br><br>Monitor Blood Sugar: Check your blood sugar levels before, during, and after exercise. This helps prevent hypoglycemia (low blood sugar) or hyperglycemia (high blood sugar). Carry a quick source of glucose, like glucose tablets or fruit juice, in case of low blood sugar.<br><br>Stay Hydrated: Drink plenty of water before, during, and after your workout to stay hydrated and help regulate blood sugar levels.<br><br>Wear Appropriate Footwear: Diabetic neuropathy can reduce sensation in the feet. Wear comfortable, well-fitting shoes to prevent blisters and injuries. Check your feet daily for any signs of cuts, blisters, or sores.<br><br>Warm Up and Cool Down: Start with a 5-10 minute warm-up, like walking or stretching, before moving on to more intense activities. After exercising, cool down for another 5-10 minutes to gradually bring your heart rate back to normal.<br><br>Avoid Extreme Temperatures: Extreme cold or heat can affect blood sugar levels. Try to exercise indoors during very hot or cold days.<br><br>Listen to Your Body: If you feel any discomfort, pain, dizziness, or shortness of breath, stop exercising immediately and seek medical attention if necessary.<br><br>Stay Consistent: Aim for at least 150 minutes of moderate-intensity aerobic activity per week, combined with strength training exercises at least twice a week.

	Remember, every individual's needs and conditions are different. It's essential to find an exercise routine that's safe and effective for you. Stay active, stay safe, and enjoy the benefits of physical activity!
**15. What physical activities should I do if I have diabetes?**	Physical Activities for Individuals with Diabetes<br><br>Physical activity is a crucial component in managing diabetes. Regular exercise can help lower blood sugar levels, improve insulin sensitivity, and reduce the risk of diabetes-related complications. If you have diabetes, consider the following physical activities:<br><br>Aerobic Exercise: Engage in moderate-intensity aerobic activities like brisk walking, cycling, swimming, or dancing for at least 150 minutes per week. These exercises help improve cardiovascular health and regulate blood sugar levels.<br><br>Strength Training: Incorporate resistance or strength training exercises at least twice a week. This can include lifting weights, using resistance bands, or bodyweight exercises like squats and push-ups. Strength training helps build muscle mass, which can increase your metabolic rate and assist in blood sugar control.<br><br>Flexibility and Balance: Activities like yoga and tai chi not only enhance flexibility but also improve balance and coordination. This is especially important for older adults with diabetes to prevent falls and injuries.<br><br>Stretching: Before and after any physical activity, take a few minutes to stretch. Stretching can help prevent muscle soreness and improve flexibility.<br><br>Stay Active Throughout the Day: In addition to structured exercises, aim to be active throughout the day. Simple activities like taking the stairs, gardening, or doing household chores can also benefit your blood sugar levels.<br><br>Note: Before starting any exercise regimen, it's essential to consult with your healthcare provider, especially if you have any diabetes-related complications or other health conditions. They can provide guidance tailored to your individual needs and ensure that you exercise safely.
**Topic: Preventing Diabetes Problems**	
**1. How can I lower my chances of developing type 2 diabetes?**	Lowering Your Chances of Developing Type 2 Diabetes:

	Type 2 diabetes is a chronic condition where the body doesn't use insulin properly, leading to elevated blood sugar levels. While certain factors like genetics and age can't be changed, there are several lifestyle modifications you can adopt to reduce your risk:<br><br>Maintain a Healthy Weight: Being overweight increases the risk of type 2 diabetes. Aim for a body mass index (BMI) within the recommended range. Even losing 5-10% of your current weight can make a difference.<br><br>Eat a Balanced Diet: Focus on whole foods like vegetables, fruits, lean proteins, and whole grains. Limit processed foods, sugary drinks, and excessive amounts of unhealthy fats.<br><br>Stay Active: Engage in at least 150 minutes of moderate-intensity aerobic activity, like brisk walking, every week. Muscle-strengthening activities on two or more days a week can also help.<br><br>Limit Alcohol and Quit Smoking: Excessive alcohol can lead to weight gain and affect insulin sensitivity. Smoking is also a risk factor for diabetes and many other health conditions.<br><br>Regular Check-ups: Monitor your blood sugar levels, blood pressure, and cholesterol. Early detection of any abnormalities can help in timely intervention.<br><br>Manage Stress: Chronic stress can affect blood sugar levels. Techniques like meditation, deep breathing exercises, and yoga can help manage stress.<br><br>Get Enough Sleep: Aim for 7-9 hours of sleep per night. Poor sleep can affect insulin sensitivity and appetite-regulating hormones.<br><br>Remember, while these steps can significantly reduce your risk, they don't eliminate it entirely. It's essential to stay informed, get regular check-ups, and work closely with healthcare professionals to monitor and maintain your health.
**2. What should I do if my health care professional told me I have prediabetes?**	If your health care professional has informed you that you have prediabetes, it means your blood sugar levels are higher than normal but not yet high enough to be classified as type 2 diabetes. However, prediabetes can progress to type 2 diabetes if not managed properly. Here's what you should do:

	Lifestyle Changes: Adopt a balanced diet rich in whole grains, lean proteins, and vegetables. Limit the intake of processed foods, sugary beverages, and excessive fats. Regular physical activity, such as brisk walking for 30 minutes a day, can also help lower your blood sugar levels.<br><br>Maintain a Healthy Weight: If you're overweight, losing even a small amount of weight (5-7% of your body weight) can significantly reduce your risk of developing type 2 diabetes.<br><br>Regular Monitoring: Schedule regular check-ups with your health care provider to monitor your blood sugar levels. This will help you track your progress and make necessary adjustments.<br><br>Medication: Some people with prediabetes may benefit from medications that help control blood sugar levels. Discuss with your doctor if this is a suitable option for you.<br><br>Stay Informed: Educate yourself about prediabetes. Understanding the condition will empower you to make informed decisions about your health.<br><br>Limit Alcohol and Quit Smoking: Both can increase your risk of type 2 diabetes. If you smoke, seek resources to help you quit, and if you drink alcohol, do so in moderation.<br><br>Manage Stress: Chronic stress can affect blood sugar levels. Find healthy ways to cope, such as meditation, deep breathing exercises, or physical activity.<br><br>Remember, prediabetes is a warning sign, but it's also an opportunity. With the right steps, you can prevent or delay the onset of type 2 diabetes and lead a healthy life.
**3. If I had gestational diabetes when I was pregnant, how can I lower my chances of developing type 2 diabetes?**	If you had gestational diabetes during pregnancy, you are at a higher risk of developing type 2 diabetes later in life. However, there are several steps you can take to reduce this risk:<br>Healthy Diet: Focus on a balanced diet rich in whole grains, lean proteins, healthy fats, and plenty of fruits and vegetables. Limit the intake of processed foods, sugary beverages, and excessive amounts of unhealthy fats.

	Regular Physical Activity: Aim for at least 150 minutes of moderate-intensity aerobic activity, like brisk walking, every week. Incorporate strength training exercises at least twice a week.<br><br>Maintain a Healthy Weight: If you're overweight, even a modest weight loss can help in reducing the risk. Work with a healthcare professional to determine a healthy weight range for you.<br><br>Regular Check-ups: Schedule regular check-ups with your healthcare provider. This will allow for early detection of any changes in your blood sugar levels.<br><br>Breastfeeding: If possible, breastfeed your baby. Breastfeeding can improve your blood sugar levels and has numerous benefits for your baby.<br><br>Avoid Smoking: If you smoke, consider quitting. Smoking increases the risk of various health complications, including type 2 diabetes.<br><br>Limit Alcohol: If you consume alcohol, do so in moderation. Excessive alcohol can lead to weight gain and may increase your risk of type 2 diabetes.<br><br>Stay Informed: Diabetes research and recommendations are continually evolving. Stay updated on the latest information and guidelines.<br><br>Remember, while these steps can significantly reduce your risk, they don't eliminate it entirely. Regular monitoring and a proactive approach to your health are crucial.
**4. What health problems can people with diabetes develop?**	Health Problems Associated with Diabetes:<br><br>People with diabetes are at an increased risk of developing a range of health complications, largely due to prolonged high blood sugar levels. Some of the most common complications include:<br><br>Cardiovascular Disease: Diabetes increases the risk of heart disease, stroke, and hypertension. High blood sugar levels can damage blood vessels and nerves that control the heart.

	Neuropathy: Elevated blood sugar can injure nerve fibers throughout the body, leading to tingling, pain, or numbness, most commonly in the hands and feet.<br><br>Nephropathy: Diabetes can damage the kidneys, impairing their ability to filter waste from the blood. Severe damage can lead to kidney failure.<br><br>Retinopathy: Diabetes can damage the blood vessels of the retina, potentially leading to blindness. It also increases the risk of other eye conditions like cataracts and glaucoma.<br><br>Foot Complications: Reduced blood flow and nerve damage in the feet can lead to ulcers, infections, and at worst, amputation.<br><br>Skin Conditions: Diabetes can make the skin more susceptible to bacterial and fungal infections.<br><br>Hearing Impairment: People with diabetes have a higher likelihood of experiencing hearing problems.<br><br>Oral Health Issues: Diabetes increases the risk of gum inflammation and infections.<br><br>Gastroparesis: Damage to the nerves controlling the stomach can slow the movement of food, leading to nausea and digestive problems.<br><br>Mental Health: Diabetes can increase the risk of depression and anxiety. Managing the disease can also be stressful, impacting overall mental well-being.<br><br>It's essential for individuals with diabetes to manage their condition through regular monitoring, medication, diet, and exercise to reduce the risk of these complications.
**5. How can I lower my chances of a heart attack or stroke if I have diabetes?**	If you have diabetes, your risk of heart attack or stroke increases. However, by taking proactive steps, you can significantly lower this risk:<br><br>Blood Sugar Control: Regularly monitor your blood glucose levels and maintain them within the target range set by your healthcare provider. Proper management can prevent or delay cardiovascular complications.

	Blood Pressure Management: High blood pressure can strain your heart, leading to heart disease. Regular check-ups and adhering to prescribed medications can help keep it in check.<br><br>Cholesterol Levels: High LDL (bad cholesterol) can clog arteries, increasing heart attack risk. Aim for lower LDL and higher HDL (good cholesterol) levels. A balanced diet, regular exercise, and medications can help achieve this.<br><br>Healthy Lifestyle Choices:<br><br>Diet: Opt for a balanced diet rich in whole grains, lean proteins, and healthy fats. Limit salt, sugar, and saturated fats.<br>Exercise: Engage in at least 150 minutes of moderate-intensity aerobic activity weekly, like brisk walking.<br>No Smoking: Smoking accelerates heart disease. If you smoke, seek help to quit.<br>Limit Alcohol: Consume in moderation or avoid altogether.<br>Regular Check-ups: Regularly visit your healthcare provider to monitor diabetes and assess cardiovascular risk. They can adjust treatments as necessary.<br><br>Medications: Adhere to prescribed medications, not just for diabetes, but also for blood pressure, cholesterol, and other conditions. Some diabetes medications also have cardiovascular benefits.<br><br>Manage Stress: Chronic stress can exacerbate diabetes and heart disease. Find healthy ways to cope, such as meditation, deep breathing exercises, or hobbies.<br><br>Maintain a Healthy Weight: Being overweight increases heart disease risk. Aim for a healthy weight through diet and exercise.<br><br>In summary, a combination of lifestyle changes, regular monitoring, and medication adherence can significantly reduce the risk of heart attack or stroke in individuals with diabetes.
**6. What are the warning signs of heart attack and stroke?**	Warning Signs of Heart Attack:

	Chest Discomfort: Most heart attacks involve discomfort in the center of the chest that lasts for more than a few minutes. It may feel like pressure, squeezing, fullness, or pain. It can also come and go.<br>Discomfort in Other Areas: Pain or discomfort can also be felt in the shoulders, arms, back, neck, jaw, or stomach.<br>Shortness of Breath: This can occur with or without chest discomfort.<br>Other Symptoms: Cold sweat, nausea, lightheadedness, or extreme fatigue.<br>Warning Signs of Stroke:<br>Remember the acronym F.A.S.T.:<br><br>Face: Ask the person to smile. Does one side of the face droop?<br>Arms: Ask the person to raise both arms. Does one arm drift downward?<br>Speech: Ask the person to repeat a simple phrase. Is their speech slurred or strange?<br>Time: If you observe any of these signs, call emergency services immediately.<br>Both heart attacks and strokes are medical emergencies. Recognizing these symptoms and seeking immediate medical attention can save a life. It's essential to act quickly, as timely treatment can reduce damage and improve outcomes.
**7. What causes low blood glucose in people with diabetes?**	What Causes Low Blood Glucose in People with Diabetes?<br><br>Low blood glucose, also known as hypoglycemia, is a condition where the level of glucose (sugar) in the blood drops below normal. For people with diabetes, several factors can lead to hypoglycemia:<br><br>Medications: The most common cause is the side effects of medications used to treat diabetes. Insulin and certain oral medications can lower blood sugar levels, and if not balanced with food intake or other factors, can lead to hypoglycemia.<br><br>Missed or Delayed Meals: Skipping meals or eating less than usual without adjusting medication can result in low blood sugar levels.<br><br>Increased Physical Activity: Exercise increases the uptake of glucose into cells, which can lower blood sugar levels. If a person with diabetes exercises more than usual without adjusting their food intake or medication, they may experience hypoglycemia.

	Alcohol Consumption: Alcohol can interfere with the liver's ability to release glucose into the bloodstream, leading to decreased blood sugar levels.<br><br>Other Medical Conditions: Some other conditions, such as kidney disorders or certain hormonal deficiencies, can affect glucose metabolism and lead to hypoglycemia.<br><br>Overcorrection of High Blood Sugar: Sometimes, in an attempt to lower high blood sugar levels, individuals might take more medication than needed, leading to a rapid drop in blood sugar.<br><br>It's essential for people with diabetes to recognize the symptoms of hypoglycemia, which can include shakiness, sweating, confusion, blurred vision, and dizziness. Immediate treatment typically involves consuming a source of fast-acting sugar, like glucose tablets or fruit juice, and monitoring blood sugar levels to ensure they return to normal.
**8. How can I prevent low blood glucose if I have diabetes?**	Preventing Low Blood Glucose (Hypoglycemia) in Diabetes<br><br>Low blood glucose, also known as hypoglycemia, can be a concerning complication for individuals with diabetes. Here are some steps to help prevent it:<br><br>Monitor Blood Glucose Regularly: Use a blood glucose meter or continuous glucose monitor (CGM) to check your levels frequently. This helps you understand how different activities and foods affect your glucose levels.<br><br>Adjust Medications: Always take medications as prescribed. If you experience frequent episodes of hypoglycemia, consult your healthcare provider. They might adjust the dosage or timing.<br><br>Eat Balanced Meals: Ensure you eat regular meals and snacks that combine carbohydrates, proteins, and fats. Avoid skipping meals, especially if you've taken insulin or oral diabetes medications.<br><br>Limit Alcohol: Alcohol can lower blood sugar levels. If you drink, do so in moderation and always with a meal.

	Exercise Safely: Physical activity can lower blood glucose. Eat a small snack before exercising if your blood sugar is on the lower side. Monitor your levels before, during, and after exercise.<br><br>Educate Yourself: Attend diabetes education programs to learn more about managing your condition and preventing complications.<br><br>Be Prepared: Always carry a source of fast-acting sugar (like glucose tablets or fruit juice) in case of hypoglycemia. Inform friends, family, and coworkers about the signs of low blood sugar and how they can help.<br><br>Night-time Checks: If you're concerned about nighttime hypoglycemia, consider checking your blood sugar levels before bed and during the night.<br><br>Consistent Routine: Try to keep a consistent routine in terms of meal timings, medication, and physical activity. Sudden changes can affect blood sugar levels.<br><br>Stay Informed: New research and technologies are constantly emerging. Stay updated and consult your healthcare provider about any new strategies or tools that might benefit you.<br><br>Remember, everyone's body reacts differently. It's essential to work closely with your healthcare team to develop a personalized plan that suits your needs.
**9. How can I keep my kidneys healthy if I have diabetes?**	Keeping Kidneys Healthy with Diabetes<br><br>Diabetes can increase the risk of kidney damage, but with proper care, you can protect your kidneys and maintain their health. Here are some steps to consider:<br><br>Blood Sugar Control: Regularly monitor your blood sugar levels and aim to keep them within the target range set by your doctor. Consistent high blood sugar can damage the tiny blood vessels in the kidneys.<br><br>Blood Pressure Management: High blood pressure can strain the kidneys. It's essential to keep your blood pressure within the recommended range. Regular check-ups, a balanced diet, and prescribed medications can help.

	Regular Check-ups: Visit your healthcare provider regularly to monitor kidney function. They may conduct urine tests to check for protein or other signs of kidney stress.<br><br>Healthy Diet: Limit salt and protein intake. Focus on fresh fruits, vegetables, whole grains, and lean proteins. Your doctor or a dietitian can provide specific recommendations.<br><br>Stay Active: Regular physical activity can help regulate blood sugar levels and blood pressure. Aim for at least 30 minutes of moderate exercise most days of the week.<br><br>Avoid NSAIDs: Over-the-counter pain relievers like ibuprofen can harm the kidneys if taken regularly. Consult your doctor before taking any medication.<br><br>Limit Alcohol and Tobacco: Both can raise blood pressure and harm the kidneys. If you drink, do so in moderation, and consider quitting smoking.<br><br>Stay Hydrated: Drink plenty of water throughout the day, but consult your doctor if you have any fluid restrictions.<br><br>Manage Stress: Chronic stress can elevate blood sugar and blood pressure. Find healthy ways to cope, such as meditation, deep breathing exercises, or hobbies.<br><br>Follow Medication Guidelines: Always take medications as prescribed and discuss any potential side effects with your healthcare provider.<br><br>Remember, early detection and proper management are key. By taking these steps, you can significantly reduce the risk of kidney complications related to diabetes.
**10. What can I do to keep my feet healthy?**	Keeping Your Feet Healthy with Diabetes<br><br>Diabetes can increase the risk of foot complications due to reduced blood flow and nerve damage (neuropathy). Therefore, maintaining foot health is crucial. Here are some essential tips:

	Regular Inspection: Check your feet daily for cuts, blisters, red spots, and swelling. Use a mirror or ask someone to help if you can't see the bottoms of your feet.<br><br>Wash with Care: Wash your feet in lukewarm water every day. Avoid soaking them, as this can dry out your skin. Gently dry, especially between the toes.<br><br>Moisturize: Apply a thin coat of lotion or petroleum jelly to the tops and bottoms of your feet. Avoid putting creams between your toes, as this can lead to fungal infections.<br><br>Trim Nails Carefully: Cut your toenails straight across and file the edges to avoid ingrown toenails. If you have concerns about your nails, consult a podiatrist.<br><br>Wear Proper Footwear: Always wear shoes and socks, even indoors. Choose well-fitting, comfortable shoes that protect your feet. Avoid tight or pointy shoes that can cause pressure points.<br><br>Protect from Extremes: In cold weather, wear warm socks. In hot weather, use sunscreen on the tops of your feet. Never walk barefoot on hot pavements or sand.<br><br>Promote Blood Flow: Elevate your feet when sitting. Wiggle your toes and move your ankles several times a day. Avoid crossing your legs for long periods.<br><br>Avoid Smoking: Smoking can reduce blood flow to the feet, increasing the risk of complications.<br><br>See a Specialist: Schedule regular check-ups with a podiatrist, especially if you notice any changes or issues with your feet.<br><br>Remember, early detection and intervention can prevent serious foot complications. Stay proactive in your foot care and consult your healthcare provider with any concerns.
**11. When should I see my health care provider about foot problems?**	When to See Your Health Care Provider About Foot Problems in Diabetes:<br><br>Diabetes can lead to a range of foot-related complications. Early detection and timely intervention are crucial to prevent serious outcomes. Here are signs that you should immediately consult your health care provider:

	Numbness or Tingling: A sensation of "pins and needles" or complete loss of feeling in the feet can indicate diabetic neuropathy, a condition where high blood sugar damages nerve fibers.<br><br>Changes in Foot Shape: Deformities like bunions, hammertoes, or any unusual changes in the shape of your feet can be concerning.<br><br>Sores or Ulcers: Any open sores, especially those that don't heal within a week, can lead to infections and require immediate attention.<br><br>Swelling or Redness: Swelling in one or both feet, or areas of persistent redness, can be signs of inflammation or infection.<br><br>Pain: Persistent or unusual pain, especially if it's not related to an injury, can be a sign of underlying issues.<br><br>Cold or Hot Feet: A significant temperature change in your feet can indicate poor circulation or nerve damage.<br><br>Ingrown Toenails or Fungal Infections: These can lead to more severe infections if not treated promptly.<br><br>Dry, Cracked Skin: Especially around the heels, can become entry points for infections.<br><br>Discoloration: Dark spots, blue or purple areas, or other unusual discolorations can be signs of circulation problems or other issues.<br><br>Odor: An unusual or persistent odor can be a sign of infection.<br><br>Remember, diabetes makes it harder for your body to heal and resist infections. Regular foot self-exams can help you spot problems early. If you notice any of the above symptoms or have concerns about your feet, it's essential to see your health care provider promptly. Early intervention can prevent complications and ensure your feet remain healthy.

**12. How common is diabetic eye disease?**	Diabetic eye disease is a group of eye conditions that can affect individuals with diabetes. These conditions include diabetic retinopathy, diabetic macular edema, cataract, and glaucoma. Among these, diabetic retinopathy is the most common and a leading cause of blindness in adults.<br><br>According to the Centers for Disease Control and Prevention (CDC), diabetic retinopathy has been diagnosed in approximately 4.1 million adults in the United States, and that number is expected to increase as the prevalence of diabetes rises. Globally, the World Health Organization (WHO) estimates that diabetic retinopathy is responsible for 2.6% of all cases of blindness.<br><br>The risk of developing diabetic eye disease increases with the duration of diabetes. Individuals who have had diabetes for more than 20 years are at a significantly higher risk. Other factors that can increase the risk include poor blood sugar control, high blood pressure, high cholesterol, pregnancy, and tobacco use.<br><br>Early detection and timely treatment are crucial in preventing vision loss from diabetic eye disease. Regular eye exams, at least once a year, are recommended for all individuals with diabetes, regardless of whether they have any symptoms. This is because, in its early stages, diabetic eye disease often has no noticeable symptoms. By the time vision problems become apparent, the disease may have already progressed to a more advanced stage.<br><br>In conclusion, diabetic eye disease is a significant public health concern, especially given the rising prevalence of diabetes worldwide. Regular screening and early intervention are essential in managing the disease and preventing vision loss.
**13. Who is more likely to develop diabetic eye disease?**	Diabetic eye disease encompasses a group of eye conditions that affect people with diabetes. These conditions include diabetic retinopathy, diabetic macular edema, cataract, and glaucoma. The risk of developing diabetic eye disease increases with the duration of diabetes and the level of blood sugar control.<br><br>Who is more likely to develop diabetic eye disease?<br><br>Duration of Diabetes: The longer a person has diabetes, the greater their risk of developing diabetic eye disease. For instance, individuals who have had diabetes for more than 20 years are at a higher risk compared to those diagnosed more recently.<br><br>Poor Blood Sugar Control: Consistently high blood sugar levels can damage the blood vessels in the retina, leading to diabetic retinopathy. Maintaining good blood sugar control can significantly reduce the risk.

	High Blood Pressure and Cholesterol: People with diabetes who also have high blood pressure or elevated cholesterol levels are at an increased risk. These conditions can exacerbate the damage to the retinal blood vessels.<br><br>Ethnicity: Some ethnic groups, such as African Americans, Hispanics, and Native Americans, have a higher prevalence of diabetes and are, therefore, at a greater risk of developing diabetic eye complications.<br><br>Pregnancy: Diabetic women who become pregnant may experience rapid onset or worsening of diabetic retinopathy.<br><br>Tobacco Use: Smoking or using other forms of tobacco can increase the risk of diabetic eye disease and its progression.<br><br>Regular eye check-ups are crucial for early detection and treatment of these conditions, which can prevent vision loss. It's essential for individuals with diabetes to be aware of these risk factors and manage their health accordingly to reduce the likelihood of developing diabetic eye disease.
**14. When should I see my health care provider about eye problems?**	When to See Your Health Care Provider About Eye Problems in Diabetes<br><br>Diabetes can have a significant impact on your eyes, leading to conditions like diabetic retinopathy, cataracts, and glaucoma. It's essential to be proactive about your eye health to prevent or manage these complications. Here's when you should consult your health care provider:<br><br>Regular Check-ups: Even if you don't notice any issues, it's crucial to have a comprehensive eye exam at least once a year. Early detection of diabetic eye diseases can lead to more effective treatments and prevent vision loss.<br><br>Vision Changes: If you experience sudden or gradual changes in your vision, such as blurriness, double vision, or dark spots, it's essential to see your provider promptly. These could be signs of diabetic retinopathy or other eye conditions.<br><br>Pain or Pressure: Any persistent pain or a feeling of pressure in your eyes should be addressed immediately. It could indicate conditions like glaucoma.

	Flashes or Floaters: Seeing flashes of light or floating spots can be a sign of retinal detachment, which is a medical emergency.<br><br>Difficulty in Night Vision: If you find it challenging to see at night or in low light, it might be an early sign of cataract development.<br><br>Redness or Swelling: Persistent redness, swelling, or any discharge from the eyes can indicate an infection or inflammation and should be addressed.<br><br>Physical Changes: If you notice any physical changes, like a cloudy lens, it could be a sign of cataracts.<br><br>Remember, diabetes increases the risk of various eye problems. Regular monitoring, maintaining blood sugar levels, and timely medical interventions can help preserve your vision. If you experience any of the above symptoms or have concerns about your eye health, don't hesitate to consult your health care provider.
**15. What can I do to protect my eyes?**	Protecting Your Eyes When You Have Diabetes<br><br>Diabetes can increase the risk of several eye conditions, including diabetic retinopathy, glaucoma, and cataracts. To protect your eyes and maintain good vision, follow these recommendations:<br><br>Regular Eye Exams: Schedule comprehensive eye exams at least once a year. Early detection of eye problems can prevent more severe complications.<br><br>Manage Blood Sugar Levels: Consistently high blood sugar can damage the tiny blood vessels in the retina. Monitor your blood sugar regularly and follow your doctor's advice on keeping it within the target range.<br><br>Control Blood Pressure and Cholesterol: High blood pressure and cholesterol can increase the risk of eye disease. Maintain a healthy diet, exercise regularly, and take prescribed medications as directed.<br><br>Wear Sunglasses: Protect your eyes from harmful UV rays by wearing sunglasses outdoors. Choose glasses that block at least 99% of UVA and UVB radiation.<br><br>Avoid Smoking: Smoking can accelerate the progression of diabetic retinopathy and other eye conditions. If you smoke, seek help to quit.

	Exercise Regularly: Physical activity can help regulate blood sugar levels and improve overall health, benefiting your eyes.<br><br>Know the Symptoms: Be aware of vision changes like blurry vision, floaters, flashing lights, or any vision loss. If you experience any of these, consult an ophthalmologist immediately.<br><br>Follow Medication Guidelines: If prescribed medications for diabetes or any other condition, take them as directed. Some medications can affect vision, so inform your doctor if you notice any changes.<br><br>In conclusion, proactive management of diabetes and a healthy lifestyle can significantly reduce the risk of eye complications. Regular check-ups and early intervention are key to preserving good vision.
**16. What if I already have some vision loss from diabetes?**	What if I already have some vision loss from diabetes?<br><br>Diabetes can lead to a range of eye complications, with diabetic retinopathy being the most common. If you've already experienced some vision loss due to diabetes, it's essential to understand the following:<br><br>Immediate Medical Attention: If you notice any sudden vision changes or loss, seek medical attention immediately. Early detection and treatment can prevent further deterioration and may even reverse some of the damage.<br><br>Regular Eye Exams: Ensure you have regular comprehensive eye exams, at least once a year, or as recommended by your ophthalmologist. These exams can detect early signs of retinopathy and other eye conditions before they become severe.<br><br>Manage Blood Sugar Levels: Keeping your blood sugar levels within the recommended range can slow the progression of retinopathy and reduce the risk of other diabetes-related eye complications.

	Treatment Options: There are several treatments available for diabetic retinopathy, including laser surgery, vitrectomy, and injections of medications into the eye. The type of treatment will depend on the severity of the retinopathy and the specific issues you're experiencing.<br><br>Lifestyle Choices: Adopting a healthy lifestyle can help manage diabetes and its complications. This includes a balanced diet, regular exercise, avoiding smoking, and controlling blood pressure and cholesterol levels.<br><br>Low Vision Aids: If you've experienced significant vision loss, low vision aids such as magnifying glasses, large-print books, and screen-reading software can help you maintain independence and quality of life.<br><br>In conclusion, while vision loss from diabetes can be concerning, with proactive management and medical care, further complications can often be prevented or minimized. Always consult with your healthcare provider about any concerns related to your vision or diabetes management.

Supplementary Table 1b: GPT-4+RISE Response to Diabetes-Related Questions

Question	GPT-4+RISE Response
**Topic: Concepts of Diabetes**	
**1. What Is Diabetes?**	Diabetes: An Overview<br><br>Diabetes is a metabolic disorder that affects the body's ability to process and utilize glucose, which is a primary source of energy for our cells. Glucose is derived from the foods we consume and is also produced by the liver. For glucose to be used by cells for energy, it needs the assistance of a hormone called insulin.<br><br>How Does Insulin Work?<br><br>Insulin is produced by the pancreas, a gland located behind the stomach. This hormone plays a crucial role in allowing glucose to enter the cells. Once inside the cells, glucose is used to produce energy. In essence, insulin acts as a key that unlocks the cell doors, permitting glucose to enter.<br><br>What Goes Wrong in Diabetes?<br><br>In individuals with diabetes, there are issues related to insulin production or function:<br><br>Type 1 Diabetes: The body's immune system mistakenly attacks and destroys the insulin-producing cells in the pancreas. As a result, little to no insulin is produced. This type of diabetes is usually diagnosed in children and young adults, which is why it's also referred to as juvenile diabetes.<br><br>Type 2 Diabetes: This is the most common form of diabetes. In type 2 diabetes, the body either resists the effects of insulin or doesn't produce enough insulin to maintain normal glucose levels. It's more common in adults, but can also be diagnosed in children and adolescents.<br><br>Gestational Diabetes: This type occurs in some women during pregnancy. While it usually resolves after giving birth, women who've had gestational diabetes have a higher risk of developing type 2 diabetes later in life.

	Complications of Diabetes<br><br>If not managed properly, high blood glucose levels can lead to a range of health complications, including:<br><br>Eye Damage: Diabetes can lead to retinopathy, cataracts, and glaucoma, increasing the risk of blindness.<br>Kidney Damage: High glucose levels can impair kidney function, leading to kidney disease or kidney failure.<br>Nerve Damage: Elevated blood sugar can damage the nerves, especially in the legs, leading to neuropathy, which can cause tingling, pain, or numbness.<br>Heart Disease: Diabetes increases the risk of heart disease, stroke, and high blood pressure.<br>Cancer: There's a link between diabetes and an increased risk of certain types of cancer.<br>Prevention and Management<br><br>Managing blood glucose levels is crucial for individuals with diabetes. This can be achieved through a combination of medication, diet, exercise, and regular monitoring. Early diagnosis and appropriate management can help prevent or delay the onset of complications.
**2. How can I determine which type of diabetes I have?**	Determining the Type of Diabetes You Have<br><br>Diabetes is a condition where the body cannot regulate blood sugar levels properly. There are three main types: Type 1, Type 2, and Gestational diabetes. Here's how you can differentiate between them:<br><br>Insulin Dependency:<br><br>Type 1 Diabetes: Your pancreas doesn't produce insulin. You must take insulin daily through injections, pens, pumps, or even an automated insulin delivery system (artificial pancreas).<br>Age and Lifestyle:

	Type 2 Diabetes: Often develops in adults but can occur at any age. Initially, it might be managed through lifestyle changes like a healthy diet, physical activity, and weight management. However, many need diabetes medicines, including insulin, especially as the disease progresses.<br>Pregnancy:<br><br>Gestational Diabetes: Only occurs during pregnancy. It can be managed with a healthy eating plan and moderate-intensity activities like brisk walking. If these aren't sufficient, insulin might be prescribed as it's safe during pregnancy.<br>Symptoms and Management:<br><br>Type 1: Rapid onset of symptoms like excessive thirst and frequent urination. Requires daily insulin.<br>Type 2: Symptoms can be milder and develop over time. Management might involve oral medications, and some may eventually need insulin.<br>Gestational: Managed with diet, exercise, and possibly insulin during pregnancy.<br>Medication and Treatment:<br><br>Regardless of the type, managing diabetes can sometimes feel burdensome due to daily medications. However, advancements in medicine and delivery systems have made it more manageable. It's essential to discuss with your doctor the best treatment options tailored to your needs.
**3. Who is more likely to develop type 1 diabetes?**	Type 1 diabetes is an autoimmune condition primarily diagnosed in children and young adults, but it can manifest at any age. Several factors influence the likelihood of developing type 1 diabetes:<br><br>Age: Predominantly, children and young adults are diagnosed, but it's not exclusive to this age group.<br>Family History: Individuals with a parent or sibling diagnosed with type 1 diabetes have a heightened risk.<br>Geographical Prevalence: In the U.S., approximately 5% of the diabetic population has type 1 diabetes.<br>Genetics: Certain genetic markers can elevate the risk.<br>Environmental Factors: Exposure to certain viruses might act as triggers.<br>Dietary Factors: Early introduction of certain foods might influence risk, though research is ongoing.<br>Awareness and early detection are crucial. If someone exhibits symptoms or has risk factors, seeking medical consultation is imperative.

**4. Who is more likely to develop type 2 diabetes?**	Type 2 diabetes is a metabolic disorder that can develop at any age, but it's more prevalent in middle-aged and older individuals. Several factors increase the likelihood of its onset:<br><br>Age: Those aged 45 or older are at a heightened risk. While it's more common in this age group, it's worth noting that even children can develop type 2 diabetes.<br><br>Ethnicity: The condition is more prevalent in certain ethnic groups. African Americans, Hispanic/Latino, American Indians, Asian Americans, and Pacific Islanders are particularly susceptible.<br><br>Family History: A family history of diabetes can predispose individuals to the condition. If a close family member, like a parent or sibling, has diabetes, the risk is higher.<br><br>Body Weight: Being overweight or obese significantly amplifies the risk. Excess weight, especially when concentrated around the abdomen, leads to increased insulin resistance.<br><br>Physical Inactivity: A sedentary lifestyle is a major risk factor. Regular physical activity helps manage weight and increases insulin sensitivity.<br><br>Other Health Conditions: High blood pressure and certain other health problems can increase the risk. Additionally, conditions that affect insulin utilization or production, such as prediabetes, elevate the likelihood of developing type 2 diabetes.<br><br>Gestational Diabetes: Women who developed diabetes during pregnancy or delivered a large baby (weighing more than 9 pounds) are at an increased risk.<br><br>It's crucial to understand that while these factors heighten the risk, they don't guarantee the onset of diabetes. Regular medical check-ups, maintaining a balanced diet, engaging in physical activity, and managing body weight are pivotal in reducing the risk of type 2 diabetes.
**Topic: Symptoms & Causes**	
**1. What are the symptoms of diabetes?**	Diabetes is a chronic condition where the body struggles with insulin production or usage, leading to elevated blood sugar levels. The symptoms of diabetes include:

	Increased thirst and urination: Due to the body's attempt to expel excess sugar.<br>Increased hunger: Cells can't get adequate sugar, causing persistent hunger.<br>Fatigue: High blood sugar can cause constant tiredness.<br>Blurred vision: Elevated sugar levels can make the eye's lens swell.<br>Numbness or tingling: Indicative of nerve damage from prolonged high sugar levels.<br>Sores that don't heal: Slow wound healing, especially on the feet.<br>Unexplained weight loss: More common in type 1 diabetes, even if eating regularly.<br>The onset of these symptoms varies:<br><br>Type 1 diabetes symptoms can appear quickly, often within weeks, and be severe.<br>Type 2 diabetes symptoms develop slowly over years and can be so mild they're unnoticed. Many with type 2 might have no symptoms and only discover their condition when facing diabetes-related complications.<br>Recognizing these symptoms and seeking medical attention is crucial for early diagnosis and management, reducing the risk of further complications.
**2. What causes type 1 diabetes?**	Type 1 diabetes is an autoimmune condition where the body's immune system mistakenly targets and destroys the insulin-producing beta cells in the pancreas. This destruction means the body is unable to produce the insulin it needs to regulate blood sugar levels effectively.<br><br>The exact cause of type 1 diabetes remains not fully understood, but it is believed to result from a combination of genetic and environmental factors. On the genetic front, certain genes are known to increase the risk of developing the condition. However, merely having these genes doesn't mean one will develop diabetes; it just heightens the susceptibility.<br><br>Environmental factors, particularly viruses, are also believed to play a role. Some research suggests that viruses like coxsackievirus, mumps, and rubella might trigger the autoimmune response in individuals who are genetically predisposed. These viruses could either initiate the autoimmune destruction of beta cells or directly infect and damage them.<br><br>Furthermore, ongoing studies, such as TrialNet, are delving deeper into the potential causes of type 1 diabetes. These studies aim to identify more precise triggers and explore possible interventions to prevent or slow down the progression of the disease.

	In summary, type 1 diabetes arises from an interplay of genetic predisposition and environmental triggers, with the body's immune system playing a central role in the onset of the disease.
**3. What causes type 2 diabetes?**	What Causes Type 2 Diabetes?<br><br>Type 2 diabetes, the most prevalent form of diabetes, arises from a combination of genetic and lifestyle factors. Here are the primary causes:<br><br>Overweight, Obesity, and Physical Inactivity: A sedentary lifestyle combined with being overweight or obese significantly increases the risk of type 2 diabetes. Notably, carrying extra weight, especially around the belly, is associated with insulin resistance, which is a precursor to type 2 diabetes. This relationship can be assessed using Body Mass Index (BMI) charts.<br><br>Insulin Resistance: This condition is characterized by the body's cells—muscle, liver, and fat cells—not utilizing insulin effectively. Initially, the pancreas compensates by producing more insulin. However, over time, it fails to meet the body's demands, leading to elevated blood glucose levels.<br><br>Genes and Family History: Genetics play a pivotal role in the onset of type 2 diabetes. If family members have the condition, the risk is higher. Additionally, certain racial and ethnic groups, including African Americans, Alaska Natives, American Indians, Asian Americans, Hispanics/Latinos, Native Hawaiians, and Pacific Islanders, are more susceptible due to genetic predispositions.<br><br>Lifestyle Factors: Consuming a diet high in processed foods and sugars, combined with a lack of physical activity, can lead to weight gain and insulin resistance, both of which are risk factors for type 2 diabetes.<br><br>In summary, while genetics can predispose an individual to type 2 diabetes, lifestyle choices, particularly those related to diet and physical activity, play a crucial role in its onset. Prevention strategies often focus on maintaining a healthy weight, staying active, and eating a balanced diet.
**4. What causes gestational diabetes?**	Gestational diabetes is a condition that arises during pregnancy when a woman's body cannot produce enough insulin to meet the increased needs, leading to elevated blood sugar levels. The exact cause of gestational diabetes is multifaceted, involving hormonal, genetic, and lifestyle factors:

	Hormonal Changes: Pregnancy triggers the production of various hormones essential for fetal growth. Hormones produced by the placenta contribute to insulin resistance, a common phenomenon in all women during late pregnancy. These hormonal changes can make the body's cells less responsive to insulin.<br><br>Genetic Factors and Family History: Genetics play a significant role in the development of gestational diabetes. Women with a family history of diabetes are at a higher risk. Additionally, certain ethnic groups, such as African Americans, American Indians, Asians, and Hispanics/Latinas, have a higher prevalence of gestational diabetes, suggesting a genetic predisposition.<br><br>Insulin Production: The pancreas is responsible for producing insulin. As insulin resistance increases during pregnancy, the pancreas needs to produce more insulin to keep blood sugar levels in check. Gestational diabetes develops when the pancreas cannot produce sufficient insulin to overcome this resistance.<br><br>Weight and Lifestyle Factors: Being overweight or having obesity before pregnancy can increase the risk of gestational diabetes as these conditions often come with pre-existing insulin resistance. Additionally, excessive weight gain during pregnancy can further exacerbate the risk. Lifestyle factors, including diet and physical activity, can also influence the development of this condition.<br><br>In summary, gestational diabetes is influenced by a combination of hormonal changes during pregnancy, genetic predispositions, and certain lifestyle factors. While it is a temporary condition that usually resolves post-pregnancy, it does increase the risk of future type 2 diabetes. Regular monitoring and a healthy lifestyle can help manage and mitigate the risks associated with gestational diabetes.
**Topic: Diabetes Tests & Diagnosis**	
**1. Who should be tested for diabetes?**	Who Should Be Tested for Diabetes?<br><br>Diabetes is a chronic condition affecting sugar metabolism. Early detection is paramount for effective management and prevention of complications. Here's who should consider testing:<br><br>Symptomatic Individuals: Anyone displaying diabetes symptoms, such as increased thirst, frequent urination, or unexplained weight loss, should be tested.<br><br>Type 1 Diabetes:

	Commonly diagnosed in children and young adults with evident symptoms.<br>Adults might exhibit less specific symptoms.<br>Relatives of type 1 diabetes patients can opt for free testing via TrialNet, even without symptoms.<br>Type 2 Diabetes:<br><br>Routine testing is advised for those with risk factors:<br>Aged 35 or older.<br>Belonging to high-risk ethnic groups: American Indian, Black or African American, Asian American, Hispanic/Latino, or Pacific Islander.<br>Overweight or obese with at least one other risk factor.<br>Women with a history of gestational diabetes.<br>Children and teens (10-18 years) who are overweight or obese and have additional risk factors should also be tested.<br>Normal test results warrant retesting every 3 years.<br>Prediabetes: Individuals with blood glucose levels higher than normal but not in the diabetes range are diagnosed with prediabetes. They should be tested for type 2 diabetes annually.<br><br>Pregnancy:<br><br>Women planning pregnancy, especially those with risk factors, should test for type 2 diabetes.<br>If already pregnant, testing for type 2 diabetes should occur at the first prenatal appointment or within the first 15 weeks.<br>All pregnant women without prior diabetes diagnosis should be tested for gestational diabetes between 24-28 weeks.<br>Post-delivery, women with gestational diabetes should test for type 2 diabetes within 12 weeks.<br>Insurance: Medicare may cover testing costs for those with specific risk factors. It's advisable to check with other insurance providers regarding coverage.<br><br>Early detection and intervention can significantly improve health outcomes. If you fall into any of the categories mentioned or have concerns, consult a healthcare professional.

**2. What tests are used to diagnose diabetes?**	Tests Used to Diagnose Diabetes:<br><br>Diabetes is diagnosed through various tests that measure blood glucose levels. The choice of test often depends on individual circumstances, such as symptoms, pregnancy status, and other health conditions:<br><br>Fasting Plasma Glucose Test (FPG): This test gauges blood glucose after an overnight fast of at least 8 hours. It provides a snapshot of your blood sugar level at a specific point in time.<br><br>A1C Test: Also known as hemoglobin A1C, HbA1C, glycated hemoglobin, or glycosylated hemoglobin test, this measures average blood glucose levels over the past 3 months. It doesn't require fasting. However, factors like pregnancy stage, certain anemias, and hemoglobin variants can affect its accuracy.<br><br>Random Plasma Glucose Test: Used when symptoms of diabetes are present, and there's no time to wait for a fasting test. This test can be done at any time, irrespective of when you last ate.<br><br>Glucose Challenge Test: Primarily for pregnant women, this test involves drinking a glucose-rich liquid, followed by a blood test an hour later. It screens for gestational diabetes.<br><br>Oral Glucose Tolerance Test (OGTT): This test can detect type 2 diabetes, prediabetes, and gestational diabetes. After fasting for 8 hours, an initial blood sample is taken. You then consume a sugary drink, and another blood sample is drawn 2 hours later. For pregnant women, multiple samples might be taken over 2 to 3 hours.<br><br>It's essential to understand that a single test result might not be conclusive. Often, doctors will repeat tests or use a combination to ensure an accurate diagnosis. If you suspect you have diabetes or are at risk, always consult with a healthcare professional.
**3. What test results tell me if I have diabetes or prediabetes?**	Understanding Diabetes and Prediabetes Test Results<br><br>To determine if you have diabetes or prediabetes, specific tests are used, each with its own measurement criteria. Here's a breakdown of what the test results mean:

	Hemoglobin A1c Test (A1C):<br><br>Normal: Below 5.7%<br>Prediabetes: 5.7% to 6.4%<br>Diabetes: 6.5% or above<br>Fasting Plasma Glucose Test (FPG):<br><br>Normal: 99 mg/dL or below<br>Prediabetes: 100 to 125 mg/dL<br>Diabetes: 126 mg/dL or above<br>Oral Glucose Tolerance Test (OGTT):<br><br>Normal: 139 mg/dL or below<br>Prediabetes: 140 to 199 mg/dL<br>Diabetes: 200 mg/dL or above<br>Random Plasma Glucose Test:<br><br>Normal: Not applicable<br>Prediabetes: Not applicable<br>Diabetes: 200 mg/dL or above<br>It's crucial to understand that a single test result indicating high blood sugar doesn't conclusively confirm diabetes. Typically, a second test is conducted to verify the diagnosis. If you are pregnant, some tests might have different cutoffs, so it's essential to consult with your doctor about your specific results.
**4. Which tests help my doctor know what kind of diabetes I have?**	Which tests help my doctor know what kind of diabetes I have?<br><br>Diagnosing diabetes involves several tests, but determining the specific type of diabetes requires additional specialized tests:

	Genetic Testing: This is primarily used to diagnose monogenic diabetes, a rare form caused by a gene mutation. Monogenic diabetes can be found in anyone but is more prevalent in infants who develop diabetes before 6 months of age, some teenagers, and some young adults. Genetic testing can identify most forms of monogenic diabetes by detecting specific gene mutations.<br><br>Autoantibodies Test: This test identifies proteins in the immune system that mistakenly attack healthy tissues and cells. If you have diabetes and these autoantibodies are present in your blood, it's an indication of type 1 diabetes. A healthcare professional will take a blood sample for this test.<br><br>Note: Even if you don't have diabetes but have a close family member with type 1 diabetes, your doctor might recommend this test. Detecting these autoantibodies can indicate a higher risk of developing type 1 diabetes in the future. In such cases, it's essential to be aware of diabetes symptoms and undergo follow-up testing as advised by your doctor.<br>While the tests mentioned above can help differentiate between the types of diabetes, it's crucial to understand that the exact type and treatment plan should be determined through a comprehensive clinical assessment. Factors like age, family history, and presentation play a role in the final diagnosis. Always consult with a healthcare professional for an accurate diagnosis and appropriate treatment.
**Topic: Managing Diabetes**	
**1. How can I manage my diabetes?**	Managing Diabetes: A Comprehensive Guide<br><br>Diabetes is a chronic condition that requires consistent care. To effectively manage diabetes, consider the following steps:<br><br>Understand Your Diabetes ABCs:<br><br>A for the A1C Test: Reflects your average blood glucose over the past 3 months. Aim for below 7 percent, but consult your health care team for your specific goal.<br>B for Blood Pressure: The target for most is below 140/90 mm Hg.<br>C for Cholesterol: Monitor both LDL ("bad") and HDL ("good") cholesterol. LDL can clog blood vessels, increasing risk of heart attack or stroke. HDL helps clear LDL from your system.<br>S for Stop Smoking: Smoking narrows blood vessels, exacerbating diabetes complications. Quitting reduces risks of heart attack, stroke, and other diabetes-related issues.

	Follow a Diabetes Meal Plan: Collaborate with your health care team to devise a meal plan. Opt for whole grains, lean proteins, and plenty of fruits and vegetables. Limit sugary and high-sodium foods.<br><br>Incorporate Physical Activity: Aim for at least 30 minutes of moderate activity, like brisk walking or swimming, most days. This aids in regulating glucose levels and overall health.<br><br>Take Prescribed Medications: Adherence to medication is pivotal. If on insulin or other diabetes medicines, ensure regular intake even if you feel well.<br><br>Monitor Blood Glucose: Regularly check blood glucose levels, especially if on insulin. Use a blood glucose meter to track daily levels, and maintain a record to discuss with your health care team.<br><br>Seek Support: Consider joining support groups or online communities. Sharing experiences can offer insights and emotional support.<br><br>Stay Educated and Communicate: Regularly consult with your health care team, discuss your goals, and make necessary adjustments to your care plan.<br><br>By focusing on these aspects, you can effectively manage diabetes, reduce potential complications, and lead a healthier life.
**2. What are the recommended targets for blood glucose levels?**	Recommended Targets for Blood Glucose Levels in Diabetes Management:<br><br>Blood glucose management is pivotal for individuals with diabetes to prevent complications and maintain overall health. The recommended targets for blood glucose levels, based on general guidelines and the provided reference, are:<br><br>Fasting Blood Glucose (Before a meal):<br><br>Target: 80-130 mg/dL<br>This level is measured after an overnight fast and before consuming the first meal of the day.<br>Postprandial Blood Glucose (Approximately 2 hours after a meal starts):

	Target: Less than 180 mg/dL<br>This level provides insight into how the body processes sugar from meals.<br>Hemoglobin A1c (HbA1c):<br><br>General Target for most adults: Less than 7%<br>HbA1c offers an average of blood sugar levels over the past 2-3 months. A percentage below 7% typically indicates good blood sugar control.<br>It's crucial to understand that these are general targets and individual needs can vary. Factors such as age, duration of diabetes, risk of hypoglycemia, and other health conditions can influence personalized targets. Therefore, it's essential to have regular consultations with a healthcare team. They can provide tailored guidance, ensuring that individualized targets align with one's health status and goals.<br><br>In conclusion, while the above targets serve as a general guideline, it's imperative to collaborate with healthcare professionals to determine the best range suitable for individual circumstances.
**3. What medicines might I take for diabetes?**	Medicines for Diabetes:<br><br>The medicine prescribed for diabetes is determined by the type of diabetes, its control over blood glucose levels, and other personal factors like other health conditions, medication costs, insurance coverage, access to care, and lifestyle.<br><br>For Type 1 Diabetes:<br><br>Insulin: This is the primary medication for Type 1 diabetes. There are multiple forms of insulin available, each with different properties and durations of action.<br>For Type 2 Diabetes:<br><br>Metformin: This is the most frequently prescribed oral medication for Type 2 diabetes. It aids in reducing blood sugar levels.<br>Sulfonylureas (e.g., glyburide, glipizide): These drugs stimulate the pancreas to produce more insulin, thereby lowering blood sugar.<br>DPP-4 inhibitors (e.g., sitagliptin, saxagliptin): They increase the levels of incretin hormones, promoting insulin release and subsequently reducing blood sugar.

	GLP-1 receptor agonists (e.g., liraglutide, semaglutide): These drugs enhance insulin secretion and decrease appetite, leading to lower blood sugar levels.<br>SGLT2 inhibitors (e.g., canagliflozin, dapagliflozin): They prevent the kidneys from reabsorbing glucose, resulting in increased glucose excretion through urine.<br>It's imperative to understand that the above list is not exhaustive. The choice of medication is influenced by individual needs, medical history, and other factors. Always consult a healthcare professional for tailored advice and treatment options.
**4. What are the different types of insulin?**	Insulin is a vital hormone that regulates blood sugar levels. For those with diabetes, external insulin may be needed. There are several types of insulin, each distinguished by its onset, peak, and duration:<br><br>Rapid-Acting Insulin:<br><br>Onset: Begins within 15 minutes.<br>Peak: 1 hour.<br>Duration: 2-4 hours.<br>Ultra Rapid-Acting Insulin:<br><br>Onset: Starts within 15 minutes.<br>Peak: 1 hour.<br>Duration: 5-7 hours.<br>Inhaled Rapid-Acting Insulin:<br><br>Onset: 10-15 minutes.<br>Peak: 30 minutes.<br>Duration: 3 hours.<br>Short-Acting (Regular) Insulin:<br><br>Onset: Begins in 30 minutes.<br>Peak: 2-3 hours.

	Duration: 3-6 hours.<br>Intermediate-Acting Insulin:<br><br>Onset: Takes 2-4 hours.<br>Peak: 4-12 hours.<br>Duration: 12-18 hours.<br>Long-Acting Insulin:<br><br>Onset: Starts in 2 hours.<br>Peak: None.<br>Duration: 24 hours.<br>Ultra Long-Acting Insulin:<br><br>Onset: Begins in 6 hours.<br>Peak: None.<br>Duration: Can last 36 hours or longer.<br>The table mentioned provides a comprehensive overview of the different insulin types, their onset, peak, and duration. It's essential for individuals with diabetes to collaborate with healthcare professionals to select the most suitable insulin type for their needs.
**5. What are the different ways to take insulin?**	Insulin administration is essential for many individuals with diabetes. The method chosen often depends on personal preferences, lifestyle, and insurance plans. Here are the different ways to take insulin:<br><br>Needle and Syringe: The traditional method where insulin is drawn from a vial and injected. While the abdomen is the fastest absorption site, rotating injection sites (thigh, buttocks, upper arm) is recommended to prevent tissue hardening. The frequency varies from multiple daily injections to just one.<br><br>Insulin Pen: Resembling a writing pen, it either comes pre-filled (disposable) or with replaceable cartridges. They offer convenience, with some advanced pens having memory functions or dose calculators. They're especially useful for those with difficulty using syringes.

	Insulin Pump: A device delivering continuous insulin doses. One type is worn externally, connected to a tube inserted under the skin. Another tubeless type adheres directly to the skin. Both types can be programmed for specific insulin needs, with additional doses administered at meal times.<br><br>Inhaler: This method involves inhaling powdered insulin, providing an alternative for those averse to needles. It's suitable for adults with type 1 or type 2 diabetes.<br><br>Jet Injector: This device administers insulin as a high-pressure spray through the skin, eliminating the need for a needle.<br><br>Artificial Pancreas: A sophisticated system comprising a continuous glucose monitor (CGM) and an insulin pump. The CGM tracks blood glucose levels, sending data to a program that calculates and adjusts insulin doses in real-time, mimicking a healthy pancreas's function.<br><br>It's crucial to discuss with a healthcare professional to determine the best insulin delivery method, considering individual needs and circumstances.
**6. What oral medicines treat type 2 diabetes?**	Oral Medications for Type 2 Diabetes:<br><br>Type 2 diabetes affects how the body processes blood sugar (glucose). To manage this condition, many individuals may need to take oral medicines, in conjunction with a healthy diet, physical activity, and other lifestyle measures.<br><br>Metformin: This is often the first medication prescribed for type 2 diabetes. Metformin works by reducing glucose production in the liver and enhancing the body's sensitivity to insulin. It is available in both pill and liquid forms. Notably, metformin may also assist in minor weight loss.<br><br>Sulfonylureas: Drugs like glipizide, glyburide, and glimepiride stimulate the pancreas to produce more insulin, aiding in lowering blood glucose levels.<br><br>Thiazolidinediones: Medications such as pioglitazone and rosiglitazone make the body's tissues more receptive to insulin.<br><br>DPP-4 Inhibitors: Drugs like sitagliptin and linagliptin influence the breakdown of a natural compound in the body, leading to reduced blood sugar levels.

	SGLT2 Inhibitors: Medications like canagliflozin, dapagliflozin, and empagliflozin prevent the kidneys from reabsorbing glucose, resulting in glucose excretion through urine.<br><br>Alpha-Glucosidase Inhibitors: Acarbose, for instance, decelerates the digestion of starches in the gut, moderating the post-meal rise in blood sugar.<br><br>Bile Acid Sequestrants: While primarily used for cholesterol management, drugs like colesevelam can also decrease blood sugar levels.<br><br>Combining two or more types of diabetes medicines can be more effective in controlling blood glucose than a single medication. It's pivotal for individuals to collaborate with their healthcare providers to identify the most suitable medication or combination for their specific needs.
**7. What should I know about side effects of diabetes medicines?**	Side Effects of Diabetes Medicines: What You Should Know<br><br>Diabetes medicines play a pivotal role in regulating blood sugar levels. However, they can come with potential side effects. It's crucial to understand these side effects and maintain open communication with your healthcare provider.<br><br>Understanding Side Effects:<br><br>Side effects are unintended reactions that can arise from taking a medication. They vary depending on the individual and the specific medicine.<br>Hypoglycemia:<br><br>Some diabetes medicines can lead to hypoglycemia, or low blood glucose. Symptoms include dizziness, sweating, and confusion. This can occur if the medicine isn't balanced with food intake and physical activity.<br>Oral Medications:<br><br>Metformin: Common side effects include upset stomach, diarrhea, and a metallic taste. These often diminish over time.<br>Sulfonylureas (e.g., glipizide, glyburide): These can cause weight gain, skin rash, and hypoglycemia.<br>DPP-4 inhibitors (e.g., sitagliptin): Potential side effects include joint pain and a slight risk of pancreatitis.

	Insulin:<br><br>Insulin can lead to hypoglycemia, especially if not coordinated with meals or if there's increased physical activity. Other side effects include weight gain and reactions at the injection site.<br>Other Side Effects:<br><br>Some diabetes medicines can cause an upset stomach, weight gain, and other digestive issues.<br>Key Recommendations:<br><br>Always adhere to the prescribed dosage and timing of your medications.<br>Be vigilant for signs of hypoglycemia and know how to treat it.<br>Discuss any unusual or persistent side effects with your doctor.<br>Regularly consult with your healthcare provider to ensure your medication regimen remains effective and safe.
**8. What foods can I eat if I have diabetes?**	Foods Suitable for Diabetics:<br><br>Vegetables:<br><br>Non-starchy: Broccoli, carrots, greens, peppers, tomatoes.<br>Starchy (in moderation): Potatoes, corn, green peas.<br>Fruits: Opt for oranges, melons, berries, apples, bananas, and grapes.<br><br>Grains: Prioritize whole grains like wheat, rice, oats, quinoa, and foods made from them, such as bread and pasta.<br><br>Protein: Choose lean meats, skinless poultry, fish, eggs, nuts, dried beans, peas, and tofu.<br><br>Dairy: Go for nonfat or low-fat options like milk, yogurt, and cheese.

	Heart-healthy fats: Use oils like canola and olive oil, nuts, seeds, fatty fish (e.g., salmon), and avocado. When cooking, prefer oils over butter or margarine.<br><br>Tips:<br><br>Balance is key. You can enjoy favorite foods in moderation.<br>Monitor portion sizes.<br>Opt for heart-healthy fats.<br>Consult with a healthcare provider for a personalized meal plan.<br>Remember, with diabetes, it's not just about avoiding certain foods but making overall healthier choices.
**9. What foods and drinks should I limit if I have diabetes?**	If you have diabetes, managing your dietary intake is crucial to control blood sugar levels. Here are foods and drinks you should limit:<br><br>1. Fried Foods: High in unhealthy fats, they can increase the risk of heart disease and inflammation.<br><br>2. Foods High in Saturated and Trans Fats: These include certain baked goods, margarines, and processed snacks. They can elevate cholesterol and contribute to heart disease.<br><br>3. Salty Foods: Excessive sodium can raise blood pressure. Limit processed foods, canned soups, and salty snacks.<br><br>4. Sweets: Baked goods, candy, ice cream, and other sugary treats can cause rapid spikes in blood sugar.<br><br>5. Beverages with Added Sugars: This includes juices, regular sodas, and sports or energy drinks. They can lead to weight gain and higher blood sugar levels.<br><br>6. Alcohol: If consumed, do so in moderation. For women, this means up to one drink a day, and for men, up to two. Alcohol can interfere with certain diabetes medications and insulin, leading to low blood sugar, especially if consumed on an empty stomach.

	Recommendations:<br><br>Opt for water or unsweetened beverages over sugary drinks. If you enjoy sweetened coffee or tea, consider using a sugar substitute.<br>When consuming alcohol, accompany it with some food to prevent a drastic drop in blood sugar.<br>In summary, a balanced diet focusing on whole, unprocessed foods with careful monitoring of carbohydrate and sugar intake is key. Always consult with a healthcare professional to tailor dietary choices to your individual needs.
**10. When should I eat if I have diabetes?**	When Should I Eat If I Have Diabetes?<br><br>Managing meal timings is essential for individuals with diabetes to maintain stable blood sugar levels. Here's what you need to know:<br><br>Consistent Meal Times: Some people with diabetes benefit from eating at consistent times daily. This predictability helps the body manage blood sugar fluctuations more effectively.<br><br>Flexibility in Meal Timing: Depending on the type of diabetes medicines or insulin you take, you might have some flexibility in meal timings. For instance, if you're on "mealtime" insulin, your eating schedule can be more adaptable. However, it's crucial to ensure you consume a consistent amount of carbohydrates if required by your medication regimen.<br><br>Risks of Skipping Meals: If you're on certain diabetes medicines or insulin, skipping or delaying meals can lead to a significant drop in blood glucose levels, known as hypoglycemia. It's essential to be aware of this risk and plan meals accordingly.<br><br>Eating Around Physical Activity: Physical activity can affect blood sugar levels. If you're planning to engage in exercise or any strenuous activity, consult your healthcare team. They might advise you to eat before and after the activity to prevent drastic blood sugar changes.<br><br>Consultation is Key: The information provided offers general guidelines. Individual needs can vary based on the type of diabetes, medication, lifestyle, and other factors. Always work closely with your healthcare provider or a registered dietitian to create a personalized meal plan.

**11. How much can I eat if I have diabetes?**	How Much Can I Eat If I Have Diabetes?<br><br>Managing diabetes involves monitoring food intake to maintain stable blood glucose levels. Here's a guideline:<br><br>Importance of Right Amount: Eating the correct amount helps manage blood glucose and weight. Your healthcare team can advise on daily food and calorie intake.<br><br>Weight Management: If overweight, create a weight-loss plan with your health team. The Body Weight Planner can help set calorie and activity goals. For weight loss, consume fewer calories, and opt for foods lower in calories, fat, and sugar.<br><br>Meal Planning Methods: Two popular methods are the plate method and carbohydrate counting:<br><br>Plate Method: This method emphasizes portion control without calorie counting. For lunch and dinner:<br>Use a 9-inch plate.<br>Half should be nonstarchy vegetables.<br>One-fourth should be protein (meat or alternatives).<br>One-fourth should be grains or starchy vegetables.<br>Optionally, include a small fruit bowl and a small milk glass.<br>Carbohydrate Counting: This involves tracking daily carbohydrate intake. Since carbohydrates become glucose, they significantly impact blood glucose. This method is especially useful for those on insulin. Learn which foods contain carbohydrates, read nutrition labels, and tally daily intake. Focus on healthy sources like fruits, vegetables, whole grains, beans, and low-fat milk.<br>Portion Sizes: Use everyday objects to gauge portion sizes:<br><br>Meat/Poultry: Palm of your hand or a deck of cards.<br>Fish: A checkbook.<br>Cheese: Six dice.<br>Cooked rice/pasta: A rounded handful or tennis ball.

	Pancake/Waffle: A DVD.<br>Peanut Butter: A ping-pong ball.<br>Healthy Carbohydrates: Prioritize carbohydrates from fruits, vegetables, whole grains, beans, and low-fat milk. Limit refined grains and added sugars.<br><br>Consultation: Consider medical nutrition therapy with a registered dietitian for a tailored meal plan.<br><br>Remember, individual needs vary. Always consult healthcare professionals for personalized advice.
**12. Will supplements and vitamins help my diabetes?**	Will Supplements and Vitamins Help My Diabetes?<br><br>Diabetes is a multifaceted metabolic condition that impacts the body's ability to regulate glucose. The potential of supplements and vitamins in diabetes care has been explored, but it's crucial to approach the topic with evidence in mind.<br><br>Evidence Base: There is no concrete evidence suggesting that taking dietary supplements, including vitamins, minerals, herbs, or spices, can effectively manage diabetes.1<br><br>Vitamin and Mineral Needs: While some individuals might benefit from supplements, it's typically when they can't obtain adequate vitamins and minerals from their diet. It's always preferable to meet nutritional needs through whole foods when possible.<br><br>Potential Risks: Supplements can have side effects and may interact with prescribed diabetes medications, potentially affecting their efficacy.2 It's essential to be cautious and informed.<br><br>Consultation is Key: Before considering any dietary supplement, it's imperative to consult with a healthcare provider. They can provide guidance on whether a supplement is appropriate, considering individual health status and any potential interactions with medications.2<br><br>Holistic Management: While the allure of supplements is understandable, the foundation of diabetes management remains a balanced diet, regular physical activity, and adherence to prescribed treatments. Supplements should not be viewed as a replacement for these core strategies.

	Conclusion: While the idea of using supplements and vitamins for diabetes might seem appealing, there's no robust proof of their efficacy in diabetes management.1 Always prioritize a holistic approach to diabetes care and consult with a healthcare professional before introducing any new supplements to your regimen.
**13. Why should I be physically active if I have diabetes?**	The Importance of Physical Activity for Individuals with Diabetes:<br><br>Diabetes is a condition characterized by elevated blood sugar levels. Managing these levels is crucial, and physical activity plays a pivotal role in this. Here's why being active is essential for those with diabetes:<br><br>Blood Glucose Regulation: Physical activity helps lower blood glucose levels. When you exercise, muscles utilize glucose for energy, which aids in reducing elevated blood sugar.<br><br>Cardiovascular Benefits: Exercise lowers blood pressure and improves blood circulation. This is vital as diabetes can increase the risk of heart-related issues.<br><br>Weight Management: Physical activity burns extra calories, assisting in weight control. Being overweight can exacerbate diabetes symptoms, so maintaining a healthy weight is beneficial. For those who are overweight, combining exercise with a calorie-reduced diet can lead to significant health improvements.<br><br>Mood and Mental Health: Engaging in physical activity releases endorphins, boosting mood. Additionally, it can prevent falls, enhance memory in older adults, and potentially improve sleep quality.<br><br>Prevention of Complications: Regular exercise can lead to long-term health benefits, such as better cholesterol levels, reduced sleep apnea, and increased mobility, as evidenced by the Look AHEAD study.<br><br>Recommendations: Even small increments of exercise can be beneficial. It's recommended to aim for at least 30 minutes of moderate to vigorous activity 5 days a week. For those seeking weight loss or maintenance, 60 minutes or more might be necessary.

	In summary, physical activity is not just about managing diabetes but encompasses a holistic approach to overall health. Always consult with a healthcare professional before embarking on a new exercise regimen to ensure it's appropriate for individual needs.
**14. How can I be physically active safely if I have diabetes?**	Safe Physical Activity for Individuals with Diabetes<br><br>Physical activity is beneficial for those with diabetes, but certain precautions are essential. Here's how you can exercise safely:<br><br>Consultation is Key: Before starting any exercise routine, discuss with your healthcare team, especially if you have other health issues. They can guide you on the best time to exercise, considering your meal plan and diabetes medications.<br><br>Hydration: Drink water before, during, and post-exercise to remain hydrated.<br><br>Avoid Hypoglycemia: Physical activity can lower blood glucose, so it's crucial to prevent hypoglycemia. If you're on insulin or specific diabetes medicines like sulfonylurea, you're at a higher risk. Always plan - consider taking less insulin, or have a carbohydrate-rich snack before or after intense activity. Monitoring your blood glucose levels before, during, and after exercise is advisable.<br><br>Foot Care: Due to potential nerve damage and poor blood flow from elevated blood glucose, foot care is vital. Wear comfortable, supportive shoes and inspect your feet regularly, especially after exercising.<br><br>Be Cautious with High Blood Glucose: For those with type 1 diabetes, avoid intense physical activity if you detect ketones in your blood or urine. Ketones can indicate hyperglycemia (high blood glucose) and low insulin levels. Exercising with ketones present can further elevate your blood glucose. It's essential to know the dangerous ketone levels for you and how to test for them. Note: Ketones are rare in type 2 diabetes.<br><br>In summary, while physical activity offers numerous benefits for individuals with diabetes, it's essential to take specific precautions. Regular consultations with your healthcare team, monitoring blood glucose, and proper foot care are crucial components of a safe exercise regimen.
**15. What physical activities should I do if I have diabetes?**	Physical Activities for Individuals with Diabetes

	Physical activity plays a pivotal role in diabetes management. It aids in regulating blood sugar levels, enhancing insulin sensitivity, and reducing diabetes-related complications. Based on the provided information, here are recommended activities:<br><br>Aerobic Exercise: Activities that increase your heart rate and breathing are beneficial. Aim for 30 minutes most days, which can be split throughout the day. Examples include:<br><br>Brisk walking or hiking<br>Swimming or water-aerobics<br>Dancing<br>Cycling<br>Playing sports like basketball or tennis<br>Strength Training: Building muscle is essential as it helps burn more calories and maintain weight. Engage in strength training 2-3 times weekly using:<br><br>Hand weights<br>Elastic bands<br>Weight machines<br>Start with light weights and gradually increase as you gain strength.<br>Stretching: Stretching enhances flexibility, reduces stress, and prevents muscle soreness. Yoga, including chair yoga for those with mobility issues, is an excellent way to stretch and relax.<br><br>Daily Activities: Incorporate physical activity into your daily routine:<br><br>Walk during phone calls or TV commercials.<br>Engage in chores like gardening, cleaning, or washing the car.<br>Opt for stairs over elevators.<br>Park further away when shopping.

	Plan active family outings, like biking or park walks.<br>If sitting for extended periods, engage in light activities every 30 minutes, such as leg lifts, arm stretches, or walking in place.<br>Safety First: It's crucial to consult your healthcare team before starting any exercise regimen, especially if you have complications like nerve damage or vision issues. They can provide guidance tailored to your needs, ensuring you exercise safely and effectively.
**Topic: Preventing Diabetes Problems**	
**1. How can I lower my chances of developing type 2 diabetes?**	To lower your chances of developing type 2 diabetes, consider the following evidence-based recommendations:<br><br>Weight Management: Shedding even a small amount of weight can make a significant difference. Aim to lose 5 to 7 percent of your starting weight. For example, if you're 200 pounds, a loss of 10 to 14 pounds can help in diabetes prevention.<br><br>Physical Activity: Engage in at least 30 minutes of exercise on most days of the week. If you're new to exercise, consult with a health professional to determine suitable activities and start gradually.<br><br>Dietary Choices: Emphasize a balanced diet with reduced calorie intake. Opt for smaller portions and foods lower in fat. Replace sugary drinks with water to further cut down on calories.<br><br>Consultation: Regularly consult with your health care provider. They can offer personalized advice and strategies to further reduce your risk.<br><br>Remember, the most effective approach to prevent type 2 diabetes involves long-term lifestyle changes. Initiatives like "Your Game Plan to Prevent Type 2 Diabetes" can provide structured guidance on this journey.
**2. What should I do if my health care professional told me I have prediabetes?**	If your health care professional has informed you that you have prediabetes, it's crucial to understand its implications. Prediabetes means your blood glucose levels are elevated but not yet at the level of type 2 diabetes. This condition is sometimes referred to as "borderline diabetes" and indicates a higher risk of developing type 2 diabetes in the future.<br><br>Here's what you should consider:

	Understand the Risk: About 1 in 3 Americans has prediabetes. It's essential to recognize its seriousness, as it can progress to type 2 diabetes if not addressed.<br><br>Lifestyle Changes: Adopt a reduced-calorie eating plan focusing on whole foods. Limit processed foods and sugary beverages. Aim for regular physical activity, such as walking or light exercises, to help regulate blood glucose levels.<br><br>Weight Management: If overweight, aim to lose weight. Even a modest weight loss can significantly reduce the risk of transitioning from prediabetes to type 2 diabetes.<br><br>Regular Monitoring: It's vital to have regular check-ups to monitor your blood glucose levels. This will help in tracking any changes and adjusting your management plan accordingly.<br><br>Stay Informed: Knowledge is power. Familiarize yourself with resources like the "Your Game Plan to Prevent Type 2 Diabetes" and consider joining support programs like the National Diabetes Prevention Program to get guidance and community support.<br><br>Seek Support: Engage with health professionals and consider joining support groups. Surrounding yourself with a supportive community can make managing and preventing the progression of prediabetes more achievable.<br><br>In summary, while a prediabetes diagnosis can be concerning, it's also an opportunity to take proactive steps towards better health. By making informed lifestyle choices and seeking support, you can significantly reduce your risk of developing type 2 diabetes.
**3. If I had gestational diabetes when I was pregnant, how can I lower my chances of developing type 2 diabetes?**	Gestational diabetes, which develops during pregnancy, often resolves postpartum. However, it increases your risk of developing type 2 diabetes within 5 to 10 years. Additionally, your child may have a heightened risk of obesity and type 2 diabetes in the future. To lower these risks, consider the following steps:<br><br>Postpartum Testing: It's crucial to get tested for diabetes 6 to 12 weeks after childbirth. If your blood glucose levels are normal, continue to get tested every 3 years.<br><br>Healthy Lifestyle Choices: Engage in regular physical activity and adopt a nutritious diet. These habits not only benefit you but also set a positive example for your child.

	Achieve a Healthy Weight: Post-pregnancy, work towards reaching and maintaining a healthy weight through balanced eating and exercise.<br><br>Breastfeeding: This offers numerous benefits for your baby, including the right balance of nutrients. It also aids in calorie burning, assisting you in returning to your pre-pregnancy weight.<br><br>Medication: Consult with your healthcare provider about the potential benefits of taking metformin, a diabetes drug that might help in preventing the onset of type 2 diabetes.<br><br>Educate and Monitor: Understand that your child may have an increased risk of obesity and diabetes. Encourage healthy habits from a young age and monitor their health as they grow.<br><br>Incorporating these steps can significantly reduce your and your child's risk of type 2 diabetes. Remember, proactive health management and regular check-ups are essential for long-term well-being.
**4. What health problems can people with diabetes develop?**	Health Problems Associated with Diabetes:<br><br>Diabetes, characterized by prolonged high blood glucose levels, can lead to various health complications. Over time, these elevated levels can damage critical organs and systems in the body. Some of the primary health problems include:<br><br>Cardiovascular Disease: High blood glucose can damage blood vessels, increasing the risk of heart disease, stroke, and hypertension. Proper management of cholesterol and blood pressure is crucial to prevent these complications.<br><br>Nephropathy (Kidney Damage): Diabetes can adversely affect the kidneys, impairing their ability to filter waste. This can escalate to kidney failure if not addressed.<br><br>Neuropathy (Nerve Damage): Elevated blood sugar can harm nerve fibers, especially in the hands and feet. This can result in tingling, numbness, and even pain.

	Retinopathy (Eye Damage): The blood vessels of the retina can be damaged by diabetes, increasing the risk of blindness. Additionally, diabetes heightens the risk of cataracts and glaucoma.<br><br>Foot Complications: Due to reduced blood flow and nerve damage, individuals with diabetes are more susceptible to foot ulcers, infections, and in severe cases, amputation.<br><br>Skin Conditions: High blood glucose can make the skin more prone to bacterial and fungal infections.<br><br>Hearing Impairment: Diabetes can lead to hearing problems, making it essential to monitor auditory health.<br><br>Oral Health Issues: Gum inflammation and infections are more common in people with diabetes.<br><br>To mitigate these health problems, individuals with diabetes should actively manage their disease. By consistently monitoring and managing blood glucose, blood pressure, and cholesterol levels, one can significantly lower the chances of developing these complications. Adopting a healthy lifestyle, understanding the disease, and seeking regular medical check-ups are vital steps towards improved health for those with diabetes.
**5. How can I lower my chances of a heart attack or stroke if I have diabetes?**	Lowering Heart Attack or Stroke Risk with Diabetes:<br><br>Manage Your Diabetes ABCs:<br><br>A1C Test: Reflects your average blood glucose over the past 3 months. Aim for an A1C below 7%, but consult your healthcare team for your specific goal.<br>Blood Pressure: High blood pressure strains your heart. The target for most diabetics is below 140/90 mm Hg.<br>Cholesterol: High cholesterol can lead to heart attacks or strokes. If over 40, you might need medications like statins to manage it. Know your cholesterol numbers and consult your healthcare team for guidance.<br>Stop Smoking: Smoking and diabetes both narrow blood vessels. Quitting reduces risks of heart attack, stroke, and other complications. Avoid e-cigarettes too.<br>Healthy Lifestyle Habits:

	Diet: Follow a balanced eating plan.<br>Physical Activity: Incorporate regular exercise into your routine.<br>Weight Management: Aim for a healthy weight.<br>Sleep: Ensure adequate rest.<br>Manage Stress: Chronic stress can elevate blood glucose and pressure. Find relaxation techniques like deep breathing, walking, or hobbies.<br><br>Medications:<br><br>Protection: Some medicines help achieve A1C, blood pressure, and cholesterol goals, reduce clotting risks, and treat heart-related symptoms.<br>Aspirin: Consult your doctor about daily aspirin intake, as it's not suitable for everyone.<br>Statins: These can reduce heart attack or stroke risks for some diabetics. Additionally, certain diabetes medicines can lower heart attack risks.<br>Regular Check-ups: Monitor your ABCs and discuss any concerns with your healthcare team. Consider consulting a diabetes educator or dietitian to enhance your diabetes management.<br><br>In summary, a combination of monitoring, lifestyle changes, stress management, and medication adherence can significantly reduce heart attack or stroke risks for diabetics. Always consult with a healthcare professional for personalized advice.
**6. What are the warning signs of heart attack and stroke?**	Warning Signs of Heart Attack:<br><br>Chest Discomfort: Pain or pressure in the chest that lasts longer than a few minutes or that goes away and comes back.<br>Other Discomforts: Pain or discomfort can manifest in one or both arms, shoulders, back, neck, or jaw.<br>Shortness of Breath: This can occur with or without chest discomfort.<br>Additional Symptoms: Sweating, light-headedness, indigestion, nausea, and extreme fatigue.<br>It's crucial to note that warning signs can vary among individuals. Women might experience symptoms like nausea, vomiting, fatigue, and pain that spreads to the back, neck, throat, arms, shoulders, or jaw. Importantly, people with diabetes-related nerve damage might not feel any chest pain.<br><br>Warning Signs of Stroke:

	Face: Weakness or numbness on one side, causing a droop.<br>Arm: Weakness or numbness in one arm.<br>Speech: Confusion, difficulty speaking, or understanding speech.<br>Vision: Trouble seeing out of one or both eyes.<br>Balance: Dizziness, loss of balance, or difficulty walking.<br>Headache: A sudden, severe headache without a known cause.<br>For both heart attacks and strokes, it's imperative to act immediately. If you or someone you know exhibits any of these symptoms, call 9-1-1 right away. Prompt treatment can prevent permanent damage and improve outcomes, especially if medical attention is sought within the first hour of a stroke.
**7. What causes low blood glucose in people with diabetes?**	What Causes Low Blood Glucose in People with Diabetes?<br><br>Low blood glucose, commonly referred to as hypoglycemia, occurs when the level of glucose in the blood falls below the normal range. For individuals with diabetes, several factors can contribute to this condition:<br><br>Medications: Insulin and certain diabetes pills can lead to hypoglycemia. Specifically:<br><br>Insulin: Directly lowers blood glucose and can cause hypoglycemia if not balanced with food intake.<br>Sulfonylureas: These pills, typically taken once or twice daily, stimulate the pancreas to release insulin over several hours.<br>Meglitinides: Taken before meals, they promote a short-term insulin increase.<br>Dietary Factors:<br><br>Insufficient Carbohydrate Intake: Carbohydrates are broken down into glucose, raising blood glucose levels. Not consuming enough carbohydrates or skipping meals can lead to a drop in blood glucose.<br>Fasting: If one continues to take glucose-lowering medications while fasting, it can result in hypoglycemia.<br>Physical Activity: An increase in physical activity can lower blood glucose levels for up to 24 hours post-exercise. It's crucial to balance activity with medication and carbohydrate intake.

	Alcohol Consumption: Drinking excessive alcohol, especially on an empty stomach, can disrupt the body's ability to maintain stable blood glucose levels. Alcohol can also mask the initial symptoms of hypoglycemia, leading to more severe episodes.<br><br>Illness: Being sick can reduce food intake or cause vomiting, both of which can lower blood glucose levels.<br><br>It's vital for people with diabetes to recognize hypoglycemia symptoms, such as shakiness, sweating, and confusion. Immediate treatment typically involves consuming a fast-acting sugar source and monitoring blood glucose levels.
**8. How can I prevent low blood glucose if I have diabetes?**	Preventing Low Blood Glucose (Hypoglycemia) in Diabetes<br><br>Low blood glucose, or hypoglycemia, is a potential risk for those with diabetes, especially if they take insulin or certain medications. Here's how you can prevent it:<br><br>Monitor Blood Glucose: Use a blood glucose meter to regularly check your levels. For those with frequent hypoglycemia or hypoglycemia unawareness, a continuous glucose monitor (CGM) can be beneficial. CGMs measure blood glucose at regular intervals and can alert you if levels drop too low.<br><br>Dietary Considerations: Ensure your diet includes meals, snacks, and drinks with adequate carbohydrates to maintain blood glucose within the target range. Always have a source of fast-acting carbohydrate, like glucose tablets or juice, on hand.<br><br>Alcohol Consumption: If you consume alcoholic beverages, accompany them with food to stabilize blood glucose levels.

	Exercise Safely: Physical activity can reduce blood glucose levels both during the activity and for several hours afterward. To prevent hypoglycemia, check your blood glucose before, during, and post-exercise. Consider having a snack before engaging in physical activity.<br><br>Collaborate with Healthcare Professionals: Regularly consult with your doctor or healthcare team. Discuss any medications you're on that might cause low blood glucose. Together, devise strategies to prevent and treat hypoglycemic episodes. Adjustments to your diabetes management plan might be necessary over time.<br><br>In summary, preventing low blood glucose involves a combination of regular monitoring, dietary considerations, safe exercise practices, and close collaboration with your healthcare team. Being proactive and informed can help you manage your diabetes effectively and reduce the risk of hypoglycemia.
**9. How can I keep my kidneys healthy if I have diabetes?**	Keeping Kidneys Healthy with Diabetes<br><br>If you have diabetes, safeguarding your kidneys is paramount. Here's how you can do it:<br><br>Achieve Blood Glucose Goals:<br><br>Monitor your A1C levels, which reflect your average blood glucose over the past 3 months. Aim for an A1C level below 7 percent, but consult your health care team for your specific target.<br>Regularly check your blood glucose levels as advised by your health care professional. Use these results to make informed decisions about your diet, activity, and medication.<br>Control Blood Pressure:

	Blood pressure is the force exerted by your blood on your blood vessel walls. Elevated blood pressure strains your heart and can lead to heart attacks, strokes, and kidney disease.<br>Aim for a blood pressure reading below 140/90 mm Hg. Again, consult with your health care team for your specific goal.<br>Consider ACE inhibitors and ARBs, two types of blood pressure medications that are particularly beneficial for those with diabetes, high blood pressure, and diabetic kidney disease (DKD). Note: These medicines are not recommended for pregnant women.<br>Adopt Healthy Lifestyle Habits:<br><br>Quit Smoking: Smoking can exacerbate kidney damage.<br>Dietary Choices: Collaborate with a dietitian to devise a diabetes-friendly meal plan. Limit salt and sodium intake.<br>Stay Active: Incorporate regular physical activity into your daily routine.<br>Maintain a Healthy Weight: Being overweight can put additional strain on your kidneys.<br>Prioritize Sleep: Aim for 7-8 hours of sleep nightly.<br>Medication Adherence:<br><br>Medications can play a crucial role in achieving your health goals. Always take them as prescribed. Some individuals might need multiple medications to control their blood pressure.<br>Incorporating these practices can significantly improve your kidney health and overall well-being.
**10. What can I do to keep my feet healthy?**	Keeping Your Feet Healthy with Diabetes<br><br>Diabetes can lead to foot complications due to reduced blood flow and nerve damage. It's essential to prioritize foot care as part of your diabetes self-care plan. Here are some key recommendations:<br><br>Daily Inspection: Examine your feet daily for cuts, sores, red spots, swelling, and other abnormalities. If you can't see the bottoms of your feet, use a mirror or ask someone for assistance.

	Wash Daily: Clean your feet with lukewarm water and soap every day. Avoid soaking them to prevent dry skin. After washing, dry thoroughly, especially between the toes, and apply talcum powder or cornstarch to keep the area dry.<br><br>Smooth Corns and Calluses: Gently use a pumice stone to smooth out corns and calluses after bathing. Avoid cutting them or using over-the-counter removal products, which can harm your skin.<br><br>Trim Toenails Properly: Cut your toenails straight across and smooth the edges with an emery board. Avoid cutting into the corners to prevent ingrown toenails.<br><br>Always Wear Footwear: Wear shoes and socks consistently, even indoors. Ensure shoes fit well, allowing your feet to breathe. Check inside your shoes for any foreign objects before wearing them.<br><br>Protect from Temperature Extremes: Shield your feet from hot and cold temperatures. Wear shoes on hot surfaces, apply sunscreen on your feet, and avoid direct heat sources.<br><br>Promote Blood Circulation: Elevate your feet when sitting and move your toes and ankles regularly. Avoid tight socks and be physically active with foot-friendly exercises.<br><br>Regular Foot Exams: Schedule a foot examination during every health care visit. If you've had previous foot issues or changes in foot shape, ensure a thorough foot exam at each visit.<br><br>Avoid Smoking: Smoking reduces blood flow to the feet, increasing the risk of complications.<br><br>Work with Specialists: Collaborate with your health care team, including a podiatrist, to create and follow a comprehensive foot care plan.<br><br>By following these guidelines and working closely with your health care team, you can prevent foot complications and maintain healthy feet.

**11. When should I see my health care provider about foot problems?**	When to Consult Your Health Care Provider About Diabetic Foot Problems:<br><br>Diabetes increases the risk of foot complications due to reduced blood flow and nerve damage. Recognizing early signs and seeking timely medical attention can prevent severe outcomes. Here are specific situations when you should immediately contact your health care provider:<br><br>Non-Healing Wounds: If you notice a cut, blister, or bruise on your foot that doesn't show signs of healing after a few days, it's crucial to seek medical attention.<br><br>Signs of Infection: Red, warm, or painful skin on your foot can indicate an infection. Infections can escalate quickly in diabetic individuals, so prompt intervention is essential.<br><br>Callus with Dried Blood: A callus that has dried blood inside can often be an early indicator of an underlying wound. This requires immediate evaluation.<br><br>Gangrene Indicators: If a foot infection turns black and emits a foul odor, it might be a sign of gangrene. Gangrene is a severe condition where tissue dies due to lack of blood flow and can lead to amputation if not treated promptly.<br><br>Foot Deformities: Any unusual changes in the shape or structure of your feet should be evaluated, as they can increase the risk of pressure points and ulcers.<br><br>Numbness or Tingling: A sensation of "pins and needles" or a complete loss of feeling can be signs of diabetic neuropathy, a condition resulting from prolonged high blood sugar levels.<br><br>If you experience any of the above symptoms or have concerns about your feet, contact your health care provider immediately. In some cases, you might need specialized care from a podiatrist, a doctor specializing in foot health.
**12. How common is diabetic eye disease?**	Diabetic eye disease encompasses a range of eye conditions that can affect individuals with diabetes. These conditions include diabetic retinopathy, diabetic macular edema, cataract, and glaucoma. Among these, diabetic retinopathy stands out as the most prevalent and is a leading cause of vision loss among adults with diabetes.

	According to recent data, about one in three people with diabetes who are over the age of 40 already exhibit signs of diabetic retinopathy. This statistic underscores the significant impact of diabetes on ocular health. Furthermore, diabetic retinopathy has been identified as the primary cause of vision impairment in the diabetic population. However, the prognosis for each individual largely hinges on consistent medical care and early detection. Early identification and timely intervention in cases of diabetic retinopathy can diminish the risk of blindness by an impressive 95 percent.<br><br>In addition to diabetic retinopathy, diabetes also heightens the risk of other eye conditions. Specifically, individuals with diabetes have approximately double the risk of developing glaucoma or cataracts compared to those without the condition. Glaucoma is characterized by increased pressure within the eye, which can damage the optic nerve, while cataracts cause the lens of the eye to become clouded, impairing vision.<br><br>In summary, diabetic eye disease is a prevalent concern among those with diabetes, affecting a significant portion of this population. The key to mitigating the severe consequences of these eye conditions lies in regular eye check-ups, early detection, and prompt treatment. Given the heightened risk, individuals with diabetes should be especially vigilant about their eye health and seek regular consultations with ophthalmologists.
**13. Who is more likely to develop diabetic eye disease?**	Diabetic Eye Disease: Who is at Greater Risk?<br><br>Diabetic eye disease encompasses a range of eye conditions that can afflict individuals with diabetes. These conditions include diabetic retinopathy, diabetic macular edema, and others. While anyone with diabetes can develop these eye diseases, certain factors amplify the risk:<br><br>Duration of Diabetes: The longer you have diabetes, the higher your chances of developing eye complications. The risk escalates with time.<br><br>Uncontrolled Blood Glucose: Elevated blood glucose levels, if left untreated, can damage the retina's blood vessels, leading to conditions like diabetic retinopathy.<br><br>Untreated High Blood Pressure: Elevated blood pressure can exacerbate damage to the retinal blood vessels, increasing the risk of eye disease.<br><br>High Blood Cholesterol and Smoking: Both high cholesterol and smoking can elevate the risk of diabetic eye complications. They can further strain the already vulnerable blood vessels in the eyes of diabetic individuals.

	Ethnic Disparities: Certain ethnic groups, notably African Americans, American Indians and Alaska Natives, Hispanics/Latinos, and Pacific Islanders, face a heightened risk of vision loss or blindness due to diabetes.<br><br>Pregnancy: Diabetic women who become pregnant may experience rapid onset or worsening of diabetic retinopathy. The physiological changes supporting a growing fetus can stress the eyes' blood vessels. Regular eye check-ups during pregnancy are crucial to detect and address issues early.<br><br>Gestational Diabetes: Interestingly, gestational diabetes, which occurs only during pregnancy, typically doesn't lead to eye problems. The reasons for this remain under investigation.<br><br>In conclusion, while diabetes inherently poses a risk for eye diseases, certain factors and conditions can significantly amplify this risk. Regular monitoring, early detection, and appropriate management are key to safeguarding vision in diabetic individuals.
**14. When should I see my health care provider about eye problems?**	When to Consult Your Health Care Provider About Eye Problems in Diabetes<br><br>Diabetes can significantly affect your eyes, leading to conditions like diabetic retinopathy, cataracts, and glaucoma. It's vital to be vigilant about your eye health to prevent or manage these complications. Here's when you should seek medical attention:<br><br>Regular Check-ups: Even without noticeable issues, have a comprehensive eye exam annually. Early detection of diabetic eye diseases can lead to more effective treatments and prevent vision loss.<br><br>Sudden Vision Changes: If you experience abrupt changes in your vision, such as blurriness, double vision, or dark spots, consult your provider immediately. These could indicate diabetic retinopathy or other eye conditions.<br><br>Flashes or Increased Floaters: Experiencing sudden flashes of light or a significant increase in spots (floaters) in your vision is a cause for concern. These symptoms can be indicative of a detached retina, which is a medical emergency.

	Curtain-like Vision: If it appears as though a curtain has been pulled over your eyes, seek medical attention right away. This sensation can also be a symptom of retinal detachment.<br><br>Pain or Pressure: Persistent pain or a feeling of pressure in your eyes should be addressed without delay, as it could be a sign of conditions like glaucoma.<br><br>Physical Changes: Noticeable physical changes, such as a cloudy lens, might indicate cataracts.<br><br>Redness or Swelling: Persistent redness, swelling, or discharge from the eyes can suggest an infection or inflammation.<br><br>In conclusion, diabetes heightens the risk of various eye complications. Regular monitoring, maintaining optimal blood sugar levels, and seeking timely medical interventions are crucial for preserving your vision. If you experience any of the symptoms mentioned or have concerns about your eye health, consult your health care provider immediately.
**15. What can I do to protect my eyes?**	Protecting Your Eyes with Diabetes<br><br>Diabetes increases the risk of various eye diseases, such as diabetic retinopathy, glaucoma, and cataracts. To safeguard your eyes and maintain optimal vision, consider the following guidelines:<br><br>Manage Your Diabetes ABCs:<br><br>A1c Levels: Regularly monitor and maintain your A1c levels within the recommended range. A stable A1c level reduces the risk of diabetic eye complications.<br>Blood Pressure: High blood pressure can exacerbate eye problems. Ensure your blood pressure is within the target range set by your healthcare provider.<br>Cholesterol: A balanced cholesterol level is crucial for overall health, including eye health. Adhere to dietary and medication recommendations.<br>Quit Smoking: If you smoke, it's essential to quit. Smoking can accelerate the progression of diabetic eye diseases.<br><br>Regular Dilated Eye Exams: Undergo a dilated eye exam at least annually. If your eye care professional suggests more frequent exams, follow their advice. Early detection can prevent severe complications and potential blindness.

	Immediate Action: The sooner you manage your diabetes and associated health conditions, the better the outcome for your eyes. Even if you've faced challenges in managing your health previously, it's never too late to start. Improved care now can shield your eyes in the future.<br><br>Stay Informed: Continuously educate yourself about diabetes and its impact on eye health. Knowledge empowers you to take proactive measures.<br><br>In summary, proactive diabetes management, regular eye check-ups, and a healthy lifestyle are pivotal in preventing diabetic eye diseases. Remember, it's never too late to start taking better care of your eyes and overall health.
**16. What if I already have some vision loss from diabetes?**	What if I already have some vision loss from diabetes?<br><br>Diabetes can lead to various eye complications, with diabetic retinopathy being the most prevalent. If you've already experienced vision loss due to diabetes, consider the following steps:<br><br>Immediate Medical Attention: Any sudden vision changes or loss should prompt immediate medical consultation. Early detection and intervention can prevent further deterioration and might even reverse some damage.<br><br>Regular Eye Exams: Schedule comprehensive eye exams at least annually or as advised by your ophthalmologist. These can identify early signs of retinopathy and other eye conditions before they escalate.<br><br>Manage Blood Sugar Levels: Consistently maintaining blood sugar levels within the recommended range can decelerate the progression of retinopathy and reduce the risk of other diabetes-related eye issues.<br><br>Treatment Options: Various treatments for diabetic retinopathy are available, including laser surgery, vitrectomy, and eye injections with specific medications. The chosen treatment depends on the retinopathy's severity and the particular challenges you face.

	Lifestyle Choices: Embracing a healthy lifestyle can aid in managing diabetes and its complications. This encompasses a balanced diet, regular physical activity, abstaining from smoking, and controlling blood pressure and cholesterol.<br><br>Low Vision and Rehabilitation Clinics: If you've suffered significant vision loss, seek assistance from a low vision and rehabilitation clinic. Special eye care professionals at these clinics can guide you in managing vision loss that can't be rectified with standard solutions like glasses or contact lenses. They can introduce you to special devices and training to optimize your remaining vision, enabling you to remain active, pursue hobbies, socialize, and live independently.<br><br>In summary, while vision loss from diabetes is concerning, proactive management and specialized care can often prevent or mitigate further complications. Always engage with your healthcare provider regarding any concerns related to your vision or diabetes management.

Supplementary Table 1c: Claude Response to Diabetes-Related Questions

Question	Claude Response
**Topic: Concepts of Diabetes**	
**1. What Is Diabetes?**	Diabetes is a chronic health condition that affects how the body turns food into energy. There are three main types of diabetes:<br><br>Type 1 Diabetes: An autoimmune disease where the pancreas stops making insulin. It usually starts in childhood or young adulthood. People with type 1 diabetes need to take insulin every day to survive.<br>Type 2 Diabetes: The most common form, accounting for 90-95% of cases. It happens when the body becomes resistant to insulin or doesn't make enough insulin. It tends to develop in adults over 45 who are overweight, but rates in youth are rising. Risk factors include family history, poor diet, lack of exercise, and excess belly fat. It can often be managed through lifestyle changes, oral medications, and insulin if needed.<br>Gestational Diabetes: Develops in some women during pregnancy when the body cannot properly use insulin. It usually resolves after delivery of the baby but increases mom's risk of developing type 2 diabetes later on.<br>Some key facts about diabetes:<br><br>Over 34 million Americans have diabetes and 1 in 5 don't know they have it.<br>It is the 7th leading cause of death in the U.S.<br>It can lead to serious complications like kidney failure, blindness, heart disease, stroke, and amputation if not properly managed.<br>Making healthy lifestyle choices can help prevent or delay type 2 diabetes in those at risk.<br>If you have concerns about diabetes, it is important to talk to your doctor and get evaluated. Blood tests can diagnose diabetes.<br>While there is no cure yet for diabetes, it can be managed with medications, nutrition, physical activity, and regular medical care. The goal is keeping blood sugars in a healthy range to prevent complications.<br>Let me know if you need any clarification or have additional questions!
**2. How can I determine which type of diabetes I have?**	‘- Age at time of diagnosis - Type 1 diabetes usually develops in children, teens, or young adults, while type 2 most often starts in adults over the age of 40.<br><br>- Weight and body type - Type 2 diabetes is more common in people who are overweight or obese. Type 1 can occur in people of any weight.

	- Speed of symptoms - In type 1 diabetes, symptoms like increased thirst and frequent urination tend to come on quickly, over just a few weeks. With type 2, they tend to develop more slowly.<br><br>- Presence of antibodies - People with type 1 diabetes will test positive for diabetes-associated autoantibodies. This indicates an autoimmune reaction has damaged the insulin-producing cells in the pancreas.<br><br>- Need for insulin - People with type 1 diabetes require insulin injections for survival. Some people with type 2 may eventually need insulin, but not necessarily right after diagnosis.<br><br>- Family history - Having a close relative with type 1 slightly increases your risk of developing it. Type 2 has a stronger genetic link.<br><br>- Response to oral medications - People with type 2 diabetes often respond well to oral medications like metformin that lower blood sugar. Type 1 requires insulin.<br><br>The best way to confirm your type of diabetes is through blood tests ordered by your doctor. Getting an accurate diagnosis allows you to follow the proper treatment plan for managing your condition. Let me know if you have any other questions!
**3. Who is more likely to develop type 1 diabetes?**	’- Genetics - People who have a family history of type 1 diabetes are at increased risk. The condition involves specific HLA complex genes.<br><br>- Age - Though type 1 diabetes can occur at any age, it more commonly develops in children and adolescents. The peak age of onset is between 11-14 years old.<br><br>- Ethnicity - Caucasians have the highest rate of type 1 diabetes. It is less common in African Americans and Asian Americans.<br><br>- Geography - Incidence rates vary widely around the world. Generally higher rates are seen in Northern European countries like Finland and Sweden. Lower rates tend to be reported in Asian countries.

	- Viruses - Exposure to viruses may trigger autoimmunity and type 1 diabetes in those already at risk. Enteroviruses, mumps, rubella, and cytomegalovirus have been implicated.<br><br>- Diet - Early exposure to cow's milk proteins and gluten may increase risk in susceptible children. However, evidence is inconclusive.<br><br>- Season - More cases present in autumn and winter, suggesting environmental factors like viruses are involved.<br><br>In summary, while type 1 diabetes can affect anyone, those with a family history, of Northern European descent, and exposed to triggers like viruses are at greatest risk, particularly when young. Let me know if you need any clarification or have additional questions!
**4. Who is more likely to develop type 2 diabetes?**	‘- Age - The risk increases as we get older, with the highest rates occurring in those over 45 years old.<br><br>- Overweight and obesity - Carrying excess weight, especially around the abdomen, increases the risk significantly. Over 80% of people with type 2 diabetes are overweight or obese.<br><br>- Family history - Having a parent, sibling or child with type 2 diabetes increases your risk.<br><br>- Race/ethnicity - Certain ethnic groups are at higher risk, including African Americans, Hispanics/Latinos, Native Americans, Asian Americans and Pacific Islanders.<br><br>- Prediabetes - People with prediabetes (blood sugar levels above normal but not yet in the diabetic range) are at very high risk of progressing to type 2 diabetes.<br><br>- Gestational diabetes - Women who developed diabetes during pregnancy are more likely to develop type 2 diabetes later in life.<br><br>- Physical inactivity - Being inactive increases the risk. Exercise helps control weight and lower blood sugar and risk.<br><br>- High blood pressure and abnormal cholesterol - Having these cardiovascular risk factors also increases your risk of type 2 diabetes.

	The most important modifiable risk factors are overweight/obesity and physical inactivity. Maintaining a healthy weight through diet and exercise can help prevent or delay type 2 diabetes in many high risk individuals. Family history and age are risks that cannot be changed, but awareness of risk allows for early monitoring and preventative care.
**Topic: Symptoms & Causes**	
**1. What are the symptoms of diabetes?**	’- Frequent urination - When blood sugar is too high, the body tries to get rid of the excess sugar through urination. This can lead to frequent trips to the bathroom.<br><br>- Increased thirst - With frequent urination, the body loses a lot of fluid. This triggers thirst as the body tries to replace lost fluids.<br><br>- Increased hunger - Even after eating, high blood sugar levels can make the body think it is hungry. This leads to increased appetite.<br><br>- Weight loss - Despite increased hunger, weight loss is common in diabetes as the body is unable to properly utilize glucose for energy.<br><br>- Fatigue - High blood sugar levels can leave individuals feeling tired and worn out despite getting enough rest.<br><br>- Blurred vision - High blood glucose levels can cause the lens in the eye to swell, resulting in temporary vision changes.<br><br>- Slow healing wounds - High blood sugar prevents proper blood flow to extremities, making it difficult for cuts and sores to heal.<br><br>- Tingling hands or feet - Nerve damage from diabetes can cause numbness, pain, and tingling in hands and feet.<br><br>Catching these diabetes symptoms early and getting proper treatment is important to avoid complications from prolonged high blood sugar levels. If you are experiencing any of these symptoms, see your doctor and get checked for diabetes. With proper management, many people with diabetes are able to live full, healthy lives.
**2. What causes type 1 diabetes?**	Type 1 diabetes is an autoimmune disease where the immune system mistakenly attacks and destroys the insulin-producing beta cells in the pancreas. This leaves the body unable to produce enough insulin, the hormone needed to regulate blood sugar levels.

	The exact cause of why this autoimmune reaction occurs is not fully understood, but it is believed to involve a combination of genetic and environmental factors such as:<br><br>- Genetics: There are certain genes that increase the risk of developing type 1 diabetes, though not everyone with those genes gets the disease. Having a close relative with type 1 diabetes increases your risk.<br><br>- Virus exposure: Exposure to viruses may trigger the autoimmune reaction in those genetically predisposed. Viruses suspected include rubella, mumps, coxsackievirus.<br><br>- Diet: There are theories that early exposure to cow's milk proteins, gluten, or nitrates may trigger autoimmunity in susceptible individuals, but more research is needed.<br><br>- Other factors: Things like low vitamin D levels, gut bacteria imbalances, and environmental toxins may also play a role.<br><br>The bottom line is that type 1 diabetes arises from a complex interaction between genetic makeup and environmental influences. While more research is still needed, we know it is an autoimmune disease not caused by diet or lifestyle factors alone. Managing type 1 diabetes requires insulin treatment, lifestyle adjustments, and careful blood sugar monitoring.
**3. What causes type 2 diabetes?**	Type 2 diabetes is a chronic condition that affects how the body processes blood sugar (glucose). It is primarily caused by a combination of factors that result in the body becoming resistant to the effects of insulin and/or the pancreas not producing enough insulin.<br><br>The specific causes are multifactorial, but the main drivers are:<br><br>- Genetics - Having a family history of type 2 diabetes increases risk. Certain ethnic backgrounds also have higher rates of type 2 diabetes. Genetics play a role in insulin resistance and pancreatic cell function.<br><br>- Weight - Being overweight or obese is a primary risk factor. Excess weight, especially abdominal fat, leads to insulin resistance as fat interferes with the body's ability to use insulin properly.

	- Physical Inactivity - Lack of exercise can contribute to weight gain and impair the body's ability to manage blood sugar effectively.<br><br>- Diet - Diets high in processed carbohydrates, sugary foods and beverages can lead to weight gain and affect blood sugar control.<br><br>- Age - Risk increases as we get older, especially after age 45. Aging can result in loss of pancreatic cell function.<br><br>- Gestational diabetes - Women who developed high blood sugar during pregnancy have an increased risk.<br><br>- Ethnicity - Certain ethnicities, such as Hispanic/Latino Americans, African Americans, American Indians, and some Asian Americans are at higher risk.<br><br>- Fat distribution - Beyond overall weight, having extra belly fat specifically increases risk.<br><br>While genetics and age can't be changed, maintaining a healthy weight, staying active, and eating a balanced diet can help reduce the risk of developing type 2 diabetes. Controlling other risk factors like hypertension and cholesterol is also important. For those already diagnosed, lifestyle changes and medication can help manage diabetes and reduce complications.
**4. What causes gestational diabetes?**	Gestational diabetes is a type of diabetes that can develop during pregnancy in women who did not have diabetes before becoming pregnant. It happens when the hormones produced during pregnancy cause insulin resistance, which makes it difficult for the body to regulate blood sugar levels.<br><br>The main causes and risk factors for gestational diabetes include:<br><br>- Genetics - Women with a family history of type 2 diabetes are more likely to develop gestational diabetes.<br><br>- Being overweight or obese prior to pregnancy - Excess fat can contribute to insulin resistance during pregnancy.<br><br>- Advanced maternal age - Risk increases after age 25, with much higher risks over age 35.

	- Ethnic background - Hispanic, African, Native American, Asian, and Pacific Islander women have a higher risk.<br><br>- Previous history of gestational diabetes or a macrosomic baby (over 9 lbs).<br><br>- Polycyctic ovary syndrome (PCOS).<br><br>During pregnancy, the placenta produces hormones like progesterone, cortisol, placental lactogen, and growth hormone that can disrupt the body's ability to regulate glucose. The increased insulin resistance ensures sufficient glucose for the growing baby, but can cause elevated blood sugars in susceptible women.<br><br>Proper prenatal care, glucose testing, nutrition, exercise, and weight management can help reduce the risk and impacts of gestational diabetes. If untreated, it can lead to complications like preeclampsia, excess baby growth, birth injuries, and increased risk of type 2 diabetes down the road.
**Topic: Diabetes Tests & Diagnosis**	
**1. Who should be tested for diabetes?**	- Adults aged 45 years and older should be tested for diabetes, especially if they are overweight or obese. This age threshold is lowered to 35 for people with additional risk factors like family history or belonging to certain racial/ethnic groups.<br><br>- Diabetes testing should be considered in adults of any age who are overweight or obese and have one or more additional risk factors. These include high blood pressure, low HDL cholesterol and/or high triglycerides, women with polycystic ovary syndrome, and women who had gestational diabetes during pregnancy.<br><br>- People with prediabetes should be tested yearly to monitor progression to diabetes. Prediabetes is identified by a blood test showing impaired fasting glucose or impaired glucose tolerance.<br><br>- All pregnant women should be screened for gestational diabetes around the 24th week of pregnancy. Those diagnosed with gestational diabetes need ongoing testing for type 2 diabetes after giving birth.

	- For those diagnosed with diabetes, regular testing of blood glucose levels is important for monitoring and achieving treatment targets. Self-monitoring with a glucometer is recommended for people on insulin therapy or with variable blood sugars. Hemoglobin A1C tests every 3-6 months can assess overall blood sugar control.<br><br>The criteria and frequency for diabetes screening and testing may depend on a person's individual risk profile. If you have concerns about your diabetes risk, it is best to discuss testing with your doctor. I hope this overview helps provide general guidance on current recommendations. Let me know if you need any clarification or have additional questions!
**2. What tests are used to diagnose diabetes?**	- A1C test: This blood test measures your average blood sugar level over the past 2 to 3 months. An A1C of 6.5% or higher indicates diabetes. This test is useful for diagnosing both type 1 and type 2 diabetes.<br><br>- Fasting plasma glucose (FPG) test: This test measures your blood sugar level after fasting for at least 8 hours. A fasting blood sugar of 126 mg/dL or higher suggests diabetes. This test can detect prediabetes and diabetes.<br><br>- Oral glucose tolerance test (OGTT): For this test, you fast overnight and then drink a sugary drink. Your blood sugar levels are checked periodically for the next 2 hours. Higher than normal 2-hour blood sugar indicates diabetes. This test is more sensitive than FPG.<br><br>- Random plasma glucose test: This checks your blood sugar without fasting. A reading of 200 mg/dL or higher strongly suggests diabetes if you also have symptoms like increased thirst and frequent urination. This can diagnose type 1 or type 2 diabetes.<br><br>- Glycated serum protein (GSP): This measures how much sugar is bound to proteins in your blood. Higher amounts indicate diabetes. This test diagnoses only type 2 diabetes.<br><br>The A1C and FPG tests are most commonly used for screening and diagnosis. OGTT and GSP provide additional diagnostic information in ambiguous cases. Your doctor may recommend repeating tests to confirm the diagnosis of diabetes. I hope this overview of key diabetes diagnostic tests is helpful! Let me know if you need any clarification or have additional questions.Here are some key tests that are commonly used to diagnose diabetes:

	- A1C test: This blood test measures your average blood sugar level over the past 2 to 3 months. An A1C of 6.5% or higher indicates diabetes. This test is useful for diagnosing both type 1 and type 2 diabetes.<br><br>- Fasting plasma glucose (FPG) test: This test measures your blood sugar level after fasting for at least 8 hours. A fasting blood sugar of 126 mg/dL or higher suggests diabetes. This test can detect prediabetes and diabetes.<br><br>- Oral glucose tolerance test (OGTT): For this test, you fast overnight and then drink a sugary drink. Your blood sugar levels are checked periodically for the next 2 hours. Higher than normal 2-hour blood sugar indicates diabetes. This test is more sensitive than FPG.<br><br>- Random plasma glucose test: This checks your blood sugar without fasting. A reading of 200 mg/dL or higher strongly suggests diabetes if you also have symptoms like increased thirst and frequent urination. This can diagnose type 1 or type 2 diabetes.<br><br>- Glycated serum protein (GSP): This measures how much sugar is bound to proteins in your blood. Higher amounts indicate diabetes. This test diagnoses only type 2 diabetes.<br><br>The A1C and FPG tests are most commonly used for screening and diagnosis. OGTT and GSP provide additional diagnostic information in ambiguous cases. Your doctor may recommend repeating tests to confirm the diagnosis of diabetes. I hope this overview of key diabetes diagnostic tests is helpful! Let me know if you need any clarification or have additional questions.
**3. What test results tell me if I have diabetes or prediabetes?**	- A1C test - This blood test provides your average blood sugar levels over the past 2-3 months. An A1C of 6.5% or higher indicates diabetes. An A1C between 5.7% and 6.4% indicates prediabetes.<br><br>- Fasting plasma glucose test - This blood test is taken after fasting for at least 8 hours. A fasting plasma glucose of 126 mg/dL or higher indicates diabetes. A level of 100 mg/dL to 125 mg/dL indicates prediabetes.

	- Oral glucose tolerance test - For this test, you fast overnight and then drink a sugary drink. Your blood sugar levels are measured periodically for 2-3 hours. Diabetes is diagnosed if your blood sugar is 200 mg/dL or higher at the 2 hour mark. Pre diabetes is indicated by a blood sugar between 140 mg/dL and 199 mg/dL at the 2 hour mark.<br><br>- Random plasma glucose test - A blood sugar level of 200 mg/dL or higher indicates diabetes if confirmed on a subsequent day.<br><br>- HbA1c test - This blood test measures your average blood sugar over the past 2-3 months. An HbA1c of 6.5% or above indicates diabetes. An HbA1c of 5.7% to 6.4% indicates prediabetes.<br><br>In summary, diabetes is typically diagnosed if your A1C, fasting blood glucose, or 2-hour glucose tolerance tests are above the defined thresholds. Pre-diabetes is indicated by results that are elevated but not yet in the diabetes range.
**4. Which tests help my doctor know what kind of diabetes I have?**	- A1C test: This blood test measures your average blood sugar level over the past 2 to 3 months. An A1C level of 6.5% or higher indicates diabetes. This test alone does not differentiate between type 1 and type 2 diabetes, but it is used to diagnose and monitor diabetes.<br><br>- Fasting plasma glucose test: This measures your blood glucose level after fasting for at least 8 hours. A fasting blood sugar level of 126 mg/dL or higher suggests diabetes. This test may also be insufficient to distinguish between type 1 and type 2 diabetes.<br><br>- Oral glucose tolerance test: For this test, you fast overnight and then drink a sugary liquid. Blood samples are taken before and 2 hours after drinking the solution. Abnormally high glucose levels indicate diabetes. This test can help differentiate between type 1 and type 2 diabetes.<br><br>- Islet autoantibody tests: These blood tests detect autoantibodies that attack and destroy insulin-producing cells in the pancreas. Positive results indicate type 1 diabetes.<br><br>- C-peptide test: Measures levels of the peptide c-peptide, which is released when insulin is produced. Low levels suggest type 1 diabetes where insulin production is impaired. Higher levels indicate type 2 diabetes.

	- Ketone tests: Higher levels of ketones in blood or urine suggest type 1 diabetes. Without enough insulin, the body breaks down fat for fuel leading to ketone production.<br><br>In summary, while no single test confirms the diabetes type, the combination of A1C, glucose tests, islet autoantibodies, c-peptide, and ketone levels can assist doctors in determining if a patient has type 1 or type 2 diabetes. Proper diagnosis allows appropriate treatment and management.
**Topic: Managing Diabetes**	
**1. How can I manage my diabetes?**	- Monitor your blood sugar levels regularly. Test your blood sugar when you wake up, before meals, and at bedtime. Keep a log of your results and share them with your doctor.<br><br>- Follow your doctor's medication instructions carefully. Take medicines as prescribed and let your doctor know if you have any side effects.<br><br>- Eat a balanced, healthy diet. Focus on fruits, vegetables, whole grains, lean proteins, and healthy fats. Limit added sugars, refined carbs, and unhealthy fats.<br><br>- Get regular exercise. Aim for 30 minutes of moderate activity most days of the week. Walking, swimming, or strength training are great options.<br><br>- Achieve and maintain a healthy weight. Losing even a small amount of weight can help improve diabetes control.<br><br>- Don't smoke. Smoking increases complications from diabetes. If you smoke, work on quitting.<br><br>- Attend regular check-ups. See your doctor every 3-6 months to have your A1C checked, blood pressure monitored, kidneys evaluated, and feet examined.<br><br>- Manage stress. Stress can affect blood sugar levels. Try exercising, meditating, or talking to a friend or therapist to help manage stress.<br><br>- Get enough sleep.Aim for 7-8 hours per night. Lack of sleep can disrupt blood sugar control.

	Let me know if you need any clarification or have additional questions! Taking medications, following your doctor's advice, and making lifestyle changes can help you successfully manage diabetes.
**2. What are the recommended targets for blood glucose levels?**	- Fasting blood glucose (before meals): 80-130 mg/dL (American Diabetes Association), 90-130 mg/dL (American Association of Clinical Endocrinologists)<br><br>- 2 hour post-meal blood glucose: Less than 180 mg/dL (ADA), Less than 140 mg/dL (AACE)<br><br>- HbA1c (average blood glucose over 2-3 months): Less than 7% (ADA), 6.5% or less (AACE)<br><br>- Pre-meal blood glucose: 80-130 mg/dL (ADA), 90-130 mg/dL (AACE)<br><br>- Bedtime/overnight blood glucose: 100-140 mg/dL (ADA), 90-150 mg/dL (AACE)<br><br>Specific targets may vary based on factors like age, diabetes type, diabetes duration, hypoglycemia risk, and individualized goals. Blood glucose targets should be set with your doctor based on your unique health profile and needs. Maintaining glucose levels in the recommended ranges, along with medication, lifestyle changes, and ongoing medical care can help people with diabetes manage their condition and prevent complications. Let me know if you need any clarification or have additional questions!
**3. What medicines might I take for diabetes?**	Diabetes is a condition where the body cannot properly regulate blood sugar levels. There are different medications that can help manage diabetes.<br><br>For type 1 diabetes, insulin is essential and must be taken to survive. This is because the pancreas no longer makes enough or any insulin in type 1 diabetes. Insulin helps get glucose from the bloodstream into cells for energy. There are various types of insulin that differ in onset, peak, and duration of action. Short acting insulin is taken with meals to handle the glucose spike after eating. Longer acting insulins control glucose throughout the day and overnight. Insulin must be injected since it would be broken down by the stomach if swallowed.<br><br>For type 2 diabetes, the first line treatment is often metformin. Metformin makes the body more sensitive to insulin so it works better. It decreases glucose production by the liver and absorption from the gut. If metformin alone is not enough, other oral medications may be added like sulfonylureas, DPP-4 inhibitors, SGLT2 inhibitors, GLP-1 receptor agonists, or thiazolidinediones. These work by various mechanisms to lower blood glucose.

	Later in type 2 diabetes, insulin injections may also be required as the pancreas loses ability to produce insulin. Measuring blood sugars and coordinating diet, activity, and medications is key for optimal diabetes management. Work closely with your doctor and diabetes educator to find the regimen that is right for your situation.
**4. What are the different types of insulin?**	- Rapid-acting insulin - This type of insulin starts working about 15 minutes after injection and peaks in about 1 hour. Examples include insulin lispro (Humalog), insulin aspart (Novolog), and insulin glulisine (Apidra). These are usually taken right before meals.<br><br>- Short-acting insulin - This type takes effect within 30 minutes to 1 hour after injection and peaks 2 to 4 hours later. Regular or neutral insulin (Novolin R, Humulin R) falls into this category. It's sometimes used before meals but is more often used to compensate for high blood sugar.<br><br>- Intermediate-acting insulin - This type starts working 2 to 4 hours after injection and keeps working for 12 to 18 hours. NPH (Novolin N, Humulin N) is an example. It can be combined with short or rapid-acting insulins to help control blood sugar after meals.<br><br>- Long-acting insulin - This type takes 6 to 14 hours to start working and keeps working for 24 hours or longer. Examples are insulin detemir (Levemir) and insulin glargine (Lantus, Basaglar). These provide a constant background level of insulin throughout the day and night.<br><br>- Pre-mixed insulin - These contain a fixed combination of two types of insulin. Humalog Mix 75/25 contains 75% intermediate-acting insulin and 25% short-acting insulin. They provide both short-term and long-term coverage.<br><br>The type of insulin prescribed depends on a person's specific needs and blood sugar patterns. Working closely with a doctor is important to determine the optimal insulin regimen. Proper insulin use, nutrition, exercise and blood sugar monitoring are key for managing diabetes. Let me know if you need any clarification or have additional questions!
**5. What are the different ways to take insulin?**	- Injections with syringes or insulin pens - This involves injecting insulin under the skin, usually in the abdomen, thighs, or upper arms. Syringes allow measuring and injecting precise amounts of insulin. Pens are pre-filled, easy to use devices with replaceable needle tips.<br><br>- Insulin pumps - These are small, computerized devices that continuously deliver insulin through a catheter placed under the skin. The insulin dosage can be programmed to meet individual needs. Pumps help maintain steady insulin levels and can be worn discreetly.

	- Inhaled insulin - This rapid-acting insulin is inhaled as a powder through an inhaler device. It is absorbed quickly into the bloodstream and requires no injections. However, it may not be suitable for all patients.<br><br>- Jet injectors - These devices use high pressure air to push insulin through the skin without a needle. They are less common but can be an option for needle-phobic patients.<br><br>- Oral medications - While no insulin pill exists yet, some non-insulin drugs for type 2 diabetes are available as tablets. These help the body utilize insulin better or stimulate insulin secretion from the pancreas.<br><br>- Transdermal patches - Patches with insulin-containing microscopic needles that penetrate the skin are being researched. But currently no patches are approved for diabetes treatment.<br><br>The choice depends on the patient's specific needs, lifestyle, age, cost considerations etc. Doses and timing are adjusted for each method. Consulting a doctor helps determine the optimal insulin delivery method for an individual. Let me know if you need any clarification or have additional questions!
**6. What oral medicines treat type 2 diabetes?**	- Metformin is usually the first medication prescribed for type 2 diabetes. It helps reduce blood sugar levels by decreasing the amount of glucose released by the liver and improving how insulin works.<br><br>- Sulfonylureas like glipizide and glyburide stimulate the pancreas to make more insulin, which helps lower blood sugar levels. These medications can cause low blood sugar as a side effect.<br><br>- DPP-4 inhibitors like sitagliptin help reduce blood sugar by blocking the breakdown of incretin hormones that increase insulin production after meals. They have a low risk of hypoglycemia.<br><br>- SGLT2 inhibitors like empagliflozin work by blocking reabsorption of glucose in the kidneys, causing excess glucose to be eliminated in urine. They may help with weight loss and blood pressure.

	- Meglitinides like repaglinide stimulate insulin secretion similar to sulfonylureas but act faster and for a shorter duration. They too can cause hypoglycemia.<br><br>- Thiazolidinediones like pioglitazone improve insulin sensitivity in muscle and fat tissue, helping glucose uptake. They can cause fluid retention and weight gain in some people.<br><br>- Combination pills contain two medications, usually metformin plus another class. This can help improve blood sugar control compared to a single medication.<br><br>Proper diet, exercise, and regular monitoring of blood glucose levels are also important parts of managing type 2 diabetes along with medications. Let me know if you need any clarification or have additional questions!
**7. What should I know about side effects of diabetes medicines?**	- Metformin - This is usually the first medication prescribed for type 2 diabetes. It helps lower blood sugar levels by reducing the amount of glucose released by the liver and improving how insulin works. Side effects may include nausea, diarrhea, abdominal discomfort, metallic taste, and rarely lactic acidosis. These can often be minimized by starting at a low dose and increasing gradually.<br><br>- Sulfonylureas - These stimulate the pancreas to release more insulin. Side effects may include low blood sugar (hypoglycemia), weight gain, nausea, skin rashes. The risk of low blood sugar is higher in older adults.<br><br>- Meglitinides - These also stimulate insulin release but are shorter-acting than sulfonylureas. Side effects are similar - low blood sugar being the main concern. Weight gain is less likely than with sulfonylureas.<br><br>- Thiazolidinediones - These make the body more sensitive to insulin. Side effects may include weight gain, fluid retention leading to swelling, increased risk of heart failure. Liver function needs to be monitored.<br><br>- DPP-4 inhibitors - These help prevent breakdown of incretin hormones that increase insulin release. Low blood sugar is unlikely. Side effects may include nasal or sinus congestion, sore throat, headaches. Joint pain has also been reported.

	- GLP-1 receptor agonists - These slow digestion and increase insulin release. Side effects may include nausea, vomiting, diarrhea, abdominal pain. Increased risk of pancreatitis is possible. May also cause thyroid tumors in animals.<br><br>- SGLT2 inhibitors - These cause the kidneys to remove more glucose from the bloodstream. Side effects may include yeast infections, urinary tract infections, increased urination, hypotension. There are risks of diabetic ketoacidosis and limb amputation.<br><br>Let me know if you need any clarification or have additional questions! Monitoring side effects, adjusting dosages, and maintaining medical supervision is important when taking these medications.
**8. What foods can I eat if I have diabetes?**	- Focus on fiber-rich and nutrient-dense foods like vegetables, fruits, whole grains, beans, lentils, nuts, seeds, and lean proteins like fish, chicken, and turkey. These provide important nutrients and help control blood sugar.<br><br>- Limit processed foods, especially those high in saturated fat, sugar, and sodium. Foods to moderate include white bread, pasta, pizza, burgers, sugary drinks, baked goods, and sweets.<br><br>- Watch your carb intake and opt for whole food options that have fiber like whole wheat bread over white bread. Spread carb intake evenly throughout meals.<br><br>- Stay hydrated by drinking water and limiting sugary beverages like juice and soda.<br><br>- Monitor portion sizes especially with carb-heavy foods. Use a smaller plate, share entrees when dining out, and don't eat mindlessly.<br><br>- Choose healthy fats like olive oil, avocado, nuts, and fatty fish which are good for heart health. Limit trans fats.<br><br>- Create a consistent meal schedule and aim for a mix of lean protein, high-fiber carbs, healthy fats, and vegetables at meals.<br><br>- Be mindful of how different foods impact your blood sugar and adjust accordingly with guidance from your doctor or dietitian.

	The key is moderation, portion control, and eating a balanced, nutrient-dense diet. Work with your healthcare provider to develop an optimal, individualized eating plan for managing diabetes. Let me know if you have any other diet-related questions!
**9. What foods and drinks should I limit if I have diabetes?**	- Sugary drinks like soda, fruit punch, sports drinks, sweet tea, and other high-calorie beverages. These can cause blood sugar spikes. Drink water instead.<br><br>- Fruit juice and dried fruit which are high in natural sugars and carbs. Eat whole fruits in moderation.<br><br>- White bread, white rice, pasta, and other refined grain products. Opt for 100% whole grain options.<br><br>- Packaged snacks like chips, crackers, and cookies which are often high in unhealthy fats, salt, and calories. Choose unsalted nuts or seeds instead.<br><br>- Sweetened breakfast cereals since they have added sugars. Try oatmeal or whole grain cereal.<br><br>- Fatty cuts of meat like bacon, sausage, and cold cuts that are high in saturated fats. Choose leaner proteins.<br><br>- Full-fat dairy products like whole milk and full-fat cheese. Opt for low-fat or nonfat dairy.<br><br>- Fried and fast foods which tend to be low in nutrients and high in fat and calories.<br><br>- Alcohol, especially on an empty stomach, as it can cause hypoglycemia. Drink in moderation.<br><br>- Large portion sizes of any high-calorie, high-carb food. Practice portion control.<br><br>The key is to limit foods high in sugar, unhealthy fats, sodium, and refined carbs. Focus on whole foods prepared with healthy cooking methods. Moderation is important even with lower-carb foods.
**10. When should I eat if I have diabetes?**	- Eat at regular times throughout the day, rather than skipping meals or eating randomly. This helps control blood sugar levels.

	- Space out your meals about 4-5 hours apart. For many people, 3 moderate meals at breakfast, lunch, and dinner work well.<br><br>- Don't go more than 5-6 hours without eating a meal or snack. Long gaps can lead to drops in blood sugar.<br><br>- Have a snack like yogurt, fruit, or a small handful of nuts between meals if needed to maintain energy levels.<br><br>- Eat breakfast within 1-2 hours of waking up to replenish blood sugar. Breakfast helps reduce spikes in blood sugar later in the day.<br><br>- Take diabetes medications as prescribed by your doctor in relation to meals. Some must be taken right before or after eating.<br><br>- Eat dinner earlier in the evening, leaving 2-3 hours before bedtime. This helps prevent low blood sugar at night for those using insulin or certain medications.<br><br>- Adjust your meal timing and carbohydrate amounts if exercising soon after a meal. Activity can speed up digestion and blood sugar drops.<br><br>The timing of when you eat is important for keeping blood sugar steady. Work with your healthcare provider to determine the optimal eating schedule for your diabetes management. Let me know if you have any other questions!
**11. How much can I eat if I have diabetes?**	- Focus on eating a balanced, nutrient-dense diet with a variety of foods like vegetables, fruits, whole grains, lean protein, low-fat dairy rather than restricting certain foods completely.<br><br>- Be mindful of serving sizes of carbohydrate-containing foods like breads, pasta, rice, cereals, etc. These directly impact blood sugar.<br><br>- Use a measuring cup or scale to get a sense of appropriate portion sizes of carbs, which is usually 1⁄2 cup to 1 cup per meal. Be consistent in carb amounts meal-to-meal.<br><br>- Fill half your plate with non-starchy vegetables, a quarter with lean protein, and a quarter with high-fiber carbs like beans, lentils or whole grains.

	- Practice mindful eating and eat slowly to recognize when you are full. Use smaller plates and bowls.<br><br>- Check with your doctor or dietitian about the total recommended daily carb amount and calorie needs for your diabetes management plan. This determines appropriate meal sizes.<br><br>- Limit high calorie, sugary treats but you don't have to eliminate sweets completely. Focus on small portions of lower sugar desserts.<br><br>- Stay hydrated by drinking water before and between meals instead of sugary beverages.<br><br>With diabetes, the key is moderation in portion sizes, not extreme restriction. Aim for consistent carb amounts at meals and fill up on non-starchy vegetables and lean protein. Check with your healthcare team about the right portions for your needs. Let me know if you have any other questions!
**12. Will supplements and vitamins help my diabetes?**	While a healthy balanced diet is important for everyone, including those with diabetes, there is limited evidence that supplements and vitamins directly improve diabetes management. Here are some key points:<br><br>- There is no clear evidence that supplements like cinnamon, chromium, alpha-lipoic acid, or other herbal remedies have a significant impact on diabetes. They may help modestly, but lifestyle changes should be the priority.<br><br>- Routine vitamin supplementation is not generally recommended for people with diabetes who do not have any deficiencies. Work with your doctor to determine if you need any specific supplements.<br><br>- Vitamin D supplementation may be appropriate if there is a deficiency. Vitamin D has been linked to improved insulin sensitivity.<br><br>- B vitamins like folate and B12 may help decreace risk of neuropathy and nerve damage in some cases. But routine supplementation is likely not necessary.<br><br>- Antioxidant supplements like vitamins C and E have shown mixed results in studies. Evidence does not indicate routine supplementation is necessary.

	The bottom line is that while a balanced nutritious diet is very important, there is no clear evidence that supplements and vitamins are a magic bullet for diabetes. Lifestyle modifications like weight loss, exercise and healthy eating should be the foundation. Work closely with your doctor to determine if any vitamin deficiencies need to be addressed or supplements considered. But supplements and vitamins should not replace other evidence-based treatments and lifestyle changes recommended by your physician.
**13. Why should I be physically active if I have diabetes?**	Regular physical activity is extremely important for people with diabetes. Exercise helps improve blood sugar control, reduces cardiovascular risk factors, contributes to weight loss, and improves overall fitness. For these reasons, physical activity should be a vital part of managing diabetes.<br><br>First, exercise directly impacts blood glucose levels. Muscle contraction during physical activity triggers glucose uptake from the bloodstream. As a result, blood sugar goes down. Even mild to moderate aerobic exercise such as walking briskly can significantly lower blood glucose for 24 hours or more after the activity. Strength training also helps increase muscle mass so the body is better able to take up glucose.<br><br>Second, physical activity helps reduce cardiovascular risk factors - high blood pressure, high cholesterol, obesity - that often accompany diabetes. Exercise strengthens the heart and lungs, burns body fat, and promotes weight loss. Staying active is one of the best ways for people with diabetes to reduce their risk of heart disease and stroke.<br><br>Third, exercise aids weight loss and maintaining healthy body weight, which is critical in diabetes management. Carrying excess weight makes cells more resistant to insulin. Staying active, along with proper nutrition, can help people achieve and preserve healthy body weight - resulting in better blood sugar control.<br><br>Overall, regular exercise is vitally important for people with diabetes. Along with proper medication, it helps manage blood sugar levels, reduces cardiovascular risk factors, contributes to weight control, and improves fitness. Aim for 30 minutes of moderate activity most days of the week. Be sure to consult a healthcare provider before beginning a new exercise routine.
**14. How can I be physically active safely if I have diabetes?**	Regular physical activity is an important part of managing diabetes and maintaining good health. However, it's important to take some precautions when being active with diabetes to help prevent low blood sugar reactions or other complications. Here are some tips:<br><br>- Check your blood sugar level before, during, and after exercise. This helps you know if your levels are stable or dropping too low. Have a carbohydrate snack on hand in case you need to raise low blood sugar.

	- Wear proper footwear that cushions and supports your feet. Inspect your feet before and after exercise for any blisters or sores that could become infected.<br><br>- Stay hydrated by drinking plenty of water before, during and after physical activity. Dehydration can affect blood sugar levels.<br><br>- Know warning signs of low blood sugar like shakiness, sweating, irritability. Stop and treat with fast-acting carbohydrates if this occurs.<br><br>- Avoid exercising in extreme hot or cold temperatures which can increase risk of blood sugar fluctuations.<br><br>- Build up physical activity gradually so your body can adjust. Start with low-impact activities like walking or swimming.<br><br>- Listen to your body. Don't overdo it. Take breaks as needed.<br><br>- Choose activities you enjoy and find motivating. Having diabetes doesn't mean you can't be active, just take some precautions.<br><br>- Talk to your doctor about safe, appropriate activities for your individual health status and any limitations to be aware of.<br><br>The key is to be active at your own pace while monitoring blood sugar and watching for signs of lows. With some caution, people with diabetes can safely enjoy regular physical activity.
**15. What physical activities should I do if I have diabetes?**	Regular physical activity is an important part of managing diabetes and maintaining good health. However, it's important to take some precautions when being active with diabetes to help prevent low blood sugar reactions or other complications. Here are some tips:<br><br>- Check your blood sugar level before, during, and after exercise. This helps you know if your levels are stable or dropping too low. Have a carbohydrate snack on hand in case you need to raise low blood sugar.<br><br>- Wear proper footwear that cushions and supports your feet. Inspect your feet before and after exercise for any blisters or sores that could become infected.

	- Stay hydrated by drinking plenty of water before, during and after physical activity. Dehydration can affect blood sugar levels.<br><br>- Know warning signs of low blood sugar like shakiness, sweating, irritability. Stop and treat with fast-acting carbohydrates if this occurs.<br><br>- Avoid exercising in extreme hot or cold temperatures which can increase risk of blood sugar fluctuations.<br><br>- Build up physical activity gradually so your body can adjust. Start with low-impact activities like walking or swimming.<br><br>- Listen to your body. Don't overdo it. Take breaks as needed.<br><br>- Choose activities you enjoy and find motivating. Having diabetes doesn't mean you can't be active, just take some precautions.<br><br>- Talk to your doctor about safe, appropriate activities for your individual health status and any limitations to be aware of.<br><br>The key is to be active at your own pace while monitoring blood sugar and watching for signs of lows. With some caution, people with diabetes can safely enjoy regular physical activity.
**Topic: Preventing Diabetes Problems**	
**1. How can I lower my chances of developing type 2 diabetes?**	The best way to lower your chances of developing type 2 diabetes is through living an overall healthy lifestyle. First, focus on maintaining a healthy diet. Eat plenty of vegetables, fruits, whole grains, lean proteins, and healthy fats. Limit added sugars, refined grains, and unhealthy fats. Portion control is also key - avoid oversized portions. Second, engage in regular physical activity. Aim for at least 30 minutes of moderate activity most days of the week. This can include brisk walking, swimming, cycling, or other activities you enjoy. Staying active helps control weight and improves insulin sensitivity.<br><br>Third, achieve and maintain a healthy body weight. Excess weight, especially around the abdomen, increases diabetes risk. Work on losing weight if overweight and avoid weight gain if currently a healthy weight. Fourth, don't smoke. Smoking increases diabetes risk. If you currently smoke, work on quitting. Fifth, limit alcohol intake. Heavy alcohol use can contribute to weight gain and interfere with blood sugars. Moderate your alcohol consumption.

	Finally, get adequate sleep and manage stress. Poor sleep and chronic stress can negatively impact blood sugars and diabetes risk. Aim for 7-8 hours of sleep per night and utilize stress management techniques like yoga, meditation, or talking to a friend or therapist. Making lifestyle changes can significantly reduce your chances of developing type 2 diabetes. Work on establishing healthy habits that you can maintain long-term.
**2. What should I do if my health care professional told me I have prediabetes?**	Prediabetes means your blood sugar levels are higher than normal but not yet high enough to be diagnosed as type 2 diabetes. Getting diagnosed with prediabetes is actually good news because it's a wake up call that allows you to take steps to prevent or delay developing full blown diabetes. The most important thing is to take your prediabetes seriously and make lifestyle changes under the guidance of your health care provider. An overall healthy diet focusing on whole foods like vegetables, fruits, whole grains, lean proteins and healthy fats is important. Avoid processed foods and sugary drinks which spike blood sugar. Aim for 7-8 hours of sleep per night. Daily physical activity for at least 30 minutes such as brisk walking, swimming or strength training helps control weight and improve insulin sensitivity. If you're overweight, even a 5-10% reduction can make a big difference. Stop smoking and limit alcohol. Your provider may prescribe metformin or other medications to help manage blood sugar. They'll monitor your A1C level, which is a 3-month blood sugar average, to see if it's decreasing. The good news is major studies have shown that weight loss, healthy diet and exercise can return prediabetes levels to normal. So take advantage of this heads up to make changes now to avoid type 2 diabetes down the road.
**3. If I had gestational diabetes when I was pregnant, how can I lower my chances of developing type 2 diabetes?**	Gestational diabetes puts you at higher risk for developing type 2 diabetes later in life. The good news is there are steps you can take to lower your chances of getting type 2 diabetes after having gestational diabetes.<br><br>First, maintain a healthy weight. Excess weight, especially around the belly, increases insulin resistance which can lead to type 2 diabetes. Work to get to and stay at a healthy weight by exercising regularly and eating a balanced, low-fat, high fiber diet.<br><br>Second, stay active. Aim for 150 minutes per week of moderate physical activity like brisk walking. Spread activity out during the week and break it into manageable chunks of time. Strength training 2-3 times per week is also recommended. Exercise helps control blood sugar and insulin levels.<br><br>Third, eat a diabetes-friendly diet. Focus on whole grains, fruits, vegetables, lean protein, low-fat dairy and healthy fats like nuts and olive oil. Limit sweets, sugary beverages and refined carbs like white bread which can spike blood sugar.

	Fourth, get regular check-ups with your doctor. They can monitor indicators like blood sugar and HbA1c to catch prediabetes or diabetes early when it's most treatable. If lifestyle changes aren't enough, medication may be recommended to help control blood sugar.<br><br>Fifth, join a diabetes prevention program. These programs provide coaching on diet, exercise and other lifestyle changes to help prevent type 2 diabetes.<br><br>Making these positive lifestyle changes and working closely with your doctor gives you the best shot at preventing type 2 diabetes after gestational diabetes. The effort is worth it for your health.
**4. What health problems can people with diabetes develop?**	People with diabetes are at increased risk for a number of health problems, both short-term and long-term. In the short-term, people with diabetes can experience episodes of dangerously high (hyperglycemia) or low (hypoglycemia) blood sugar levels. These episodes can lead to diabetic coma or seizures if left untreated. Over time, untreated or poorly controlled diabetes can contribute to serious complications affecting many parts of the body.<br><br>Some of the most common long-term health complications associated with diabetes include:<br><br>- Cardiovascular disease - Diabetes dramatically increases the risk of various cardiovascular problems, including coronary artery disease with chest pain (angina), heart attack, stroke, narrowing of arteries (atherosclerosis), and high blood pressure.<br><br>- Nerve damage (neuropathy) - Excess sugar can injure the walls of the tiny blood vessels (capillaries) that nourish the nerves, especially in the legs. This can cause tingling, numbness, pain, and weakness.<br><br>- Kidney damage (nephropathy) - The kidneys contain millions of tiny blood vessel clusters that filter waste from the blood. Diabetes can damage this delicate filtering system. Severe damage can lead to kidney failure or irreversible end-stage kidney disease, requiring dialysis or kidney transplant.<br><br>- Eye damage (retinopathy) - Diabetes can damage the blood vessels of the retina, potentially leading to blindness.<br><br>- Foot damage - Nerve injury and poor circulation can cause diabetics to lose feeling in their feet. Left undiscovered, even minor foot injuries can result in infected ulcers, gangrene, and ultimately toe, foot, or leg amputation.

	Managing diabetes under the care of a physician can significantly reduce the risk of developing these and other complications. Blood sugar control, medication, lifestyle changes, and addressing other diabetes-related health issues are key. With proper treatment and attention, people with diabetes can often avoid the most serious health consequences of the disease.
**5. How can I lower my chances of a heart attack or stroke if I have diabetes?**	If you have diabetes, you are at higher risk for heart attack and stroke. However, there are several important steps you can take to reduce your risk. First, work closely with your doctor to keep your blood sugar, blood pressure, and cholesterol levels under control. High blood sugar, high blood pressure, and high cholesterol all raise your risk of cardiovascular problems. Take any medications prescribed to you regularly and exactly as directed.<br><br>Second, follow a heart-healthy diet that is low in saturated and trans fats, salt, and added sugars, but high in vegetables, fruits, whole grains, and lean proteins. Limit highly processed foods which are often high in salt and unhealthy fats. Staying at a healthy weight is also important.<br><br>Third, be physically active on most days of the week. Aim for at least 30 minutes per day of moderate activity like brisk walking. If you have complications from diabetes, check with your doctor first about appropriate types and amounts of activity. Quitting smoking and limiting alcohol intake are also important.<br><br>Fourth, manage stress levels. Chronic stress takes a toll on your heart health. Make time for relaxing activities and connect with loved ones for emotional support. Consider mind-body practices like meditation or yoga as well.<br><br>Fifth, monitor your blood pressure at home and work to keep it under 140/90 mm Hg. Be sure to take all blood pressure medications prescribed to you. Controlling your blood pressure significantly reduces your cardiovascular risk.<br><br>Following these tips can go a long way towards protecting your heart health while living with diabetes. Partner closely with your healthcare providers and take charge of your health. Small daily improvements add up to big benefits over time.
**6. What are the warning signs of heart attack and stroke?**	Heart attacks and strokes can happen suddenly and without warning. However, there are some common early warning signs to be aware of. For a heart attack, warning signs can include chest pain or discomfort that feels like pressure or squeezing, shortness of breath, nausea, lightheadedness, and pain that radiates down the arm or jaw. With a stroke, common signs are sudden numbness or weakness on one side of the body, trouble speaking or understanding speech, vision problems in one or both eyes, dizziness, and a severe headache.

	If you experience any of these symptoms, it is crucial to seek emergency medical care right away, even if the symptoms seem mild. Calling 911 is recommended so emergency responders can provide treatment immediately. Every minute counts when it comes to treating heart attacks and strokes.<br><br>There are also some risk factors that raise a person's chance of having a heart attack or stroke. These include high blood pressure, high cholesterol, smoking, obesity, physical inactivity, diabetes, and family history. Making lifestyle changes like quitting smoking, exercising more, and eating a healthier diet can help manage these risk factors. Additionally, following any treatment plans from your doctor, like taking prescribed medications for high blood pressure or diabetes, is important.<br><br>Knowing the signs of a heart attack or stroke, as well as your personal risk factors, can help you respond quickly and get life-saving treatment. If you have concerns about your risk, speak with your doctor to develop a prevention plan. Taking preventative steps is vital to reduce your chances of experiencing an emergency cardiovascular event.
**7. What causes low blood glucose in people with diabetes?**	There are several potential causes of low blood glucose, also called hypoglycemia, in people with diabetes. The main causes include:<br><br>- Taking too much insulin or certain oral diabetes medications - This is one of the most common reasons for hypoglycemia in people with diabetes. If someone takes too much insulin or medications that increase insulin release, it can cause blood glucose to drop too low. This is especially likely if the insulin dose or medication dose is not reduced to compensate for increased activity or reduced food intake.<br><br>- Delaying or missing a meal - Most insulin regimens and diabetes pills are calibrated to balance glucose levels around regular eating habits. Delaying or missing a meal can disrupt this balance and lead to low blood glucose reactions.<br><br>- Getting more physical activity than normal - Exercise usually lowers blood glucose. So getting significantly more exercise than normal without adjusting food or medication can trigger hypoglycemia.<br><br>- Drinking alcohol - Alcohol can inhibit glucose production in the liver and enhance insulin absorption, causing glucose levels to fall.

	- Certain medical conditions - Problems with the adrenal glands, kidney disease, liver disease and some other conditions can impair the body's ability to maintain normal blood glucose.<br><br>- Interactions between medications - Some other medications, besides diabetes pills or insulin, can increase the risk of hypoglycemia. It's important for those with diabetes to consult their doctor about potential medication interactions.<br><br>The key for people with diabetes is to monitor blood glucose regularly, coordinate medication and diet, and avoid or adjust for triggers like missed meals or unusual activity. Reporting hypoglycemic episodes to the healthcare team can also help determine if medication adjustments are needed.
**8. How can I prevent low blood glucose if I have diabetes?**	There are several effective strategies for preventing low blood glucose, also called hypoglycemia, if you have diabetes. The key is to balance your food intake, physical activity, and diabetes medications.<br><br>First, aim to eat consistent amounts of carbohydrates at each meal. Carbs raise blood sugar, so avoiding large fluctuations helps prevent drops. Include healthy carbs like fruits, vegetables, whole grains, beans and low-fat dairy. Pair carbs with protein and healthy fats to help slow digestion and maintain even blood sugar.<br><br>Second, stay active but avoid suddenly strenuous exercise without preparing. Exercise can lower blood sugar, so check your levels before and consume a carb-containing snack if needed. Wear medical identification and bring quick-acting glucose tablets when exercising.<br><br>Third, learn how your medications work so you can prevent lows. Take the right doses at the right times and don't skip. If using insulin, you may need to adjust the dosage before exercising or consuming less food. Speak to your doctor if concerned about medication effects.<br><br>Fourth, monitor your blood sugar regularly to catch drops before they cause symptoms like shakiness, sweating, dizziness, confusion and hunger. Have glucose tablets or juice on hand to quickly treat lows when detected.<br><br>Preventing low blood sugar takes diligence, but is very feasible using strategies like these. Work closely with your healthcare provider to tailor a diabetes management plan that optimizes blood sugar control and minimizes hypoglycemia risk. Maintaining your health and safety is the top priority.

**9. How can I keep my kidneys healthy if I have diabetes?**	Keeping your kidneys healthy is extremely important if you have diabetes. High blood sugar from uncontrolled diabetes can damage the small blood vessels in the kidneys, leading to diabetic kidney disease. The good news is there are several ways you can protect your kidneys if you have diabetes:<br><br>1. Keep your blood sugar in your target range. Work closely with your doctor to keep your A1C under 7% or as close to normal as possible. Checking your blood sugar regularly and taking medications as prescribed will help prevent spikes that damage kidneys.<br><br>2. Control your blood pressure. High blood pressure is very damaging to kidneys. Aim to keep your BP below 130/80, or your doctor's recommended goal. Medications like ACE inhibitors and ARBs protect kidneys.<br><br>3. Get annual kidney function tests. Your doctor should check your GFR, urine albumin, and creatinine yearly to catch any signs of kidney trouble early. Early treatment helps prevent further damage.<br><br>4. Stay hydrated and limit sodium. Drink plenty of water to avoid dehydration. Restrict salt to less than 2,300 mg per day to lower blood pressure.<br><br>5. Take diabetes medications that protect kidneys. SGLT2 inhibitors and GLP-1 agonists have been shown to help prevent kidney decline in those with diabetes. Ask your doctor if these could help.<br><br>6. Stop smoking and limit alcohol. Smoking worsens kidney damage, so quitting is important. Heavy alcohol use is also hard on kidneys.<br><br>7. Control your cholesterol and triglycerides. Work with your doctor to keep LDL under 100 mg/dL and triglycerides under 150 mg/dL to protect kidneys.<br><br>Following these tips can go a long way in preserving kidney function and avoiding further complications of uncontrolled diabetes. Be diligent with your self-care and work closely with your healthcare providers.
**10. What can I do to keep my feet healthy?**	Keeping your feet healthy is crucial for people with diabetes. The nerve damage caused by high blood sugar can reduce sensation in the feet, making it easier to get cuts, sores or infections without realizing it. Here are some tips to keep your feet in good shape:

	- Inspect your feet daily - Check for any cuts, blisters, redness or swelling. Be sure to look between the toes and the bottoms of feet too. Use a mirror if needed. Call your doctor right away about any sores or irritation that do not start to heal within a day or two.<br><br>- Wash feet daily - Use warm, not hot, water and a mild soap. Avoid soaking feet. Be sure to dry thoroughly, especially between the toes.<br><br>- Moisturize feet - Use a thin layer of non-perfumed, non-medicated moisturizer on the tops and bottoms of feet to prevent cracking. Do not moisturize between the toes as moisture can allow fungus to grow.<br><br>- Trim toenails carefully - Cut straight across to avoid ingrown toenails. Smooth edges with an emery board.<br><br>- Wear clean, dry socks - Change socks daily. Avoid socks with seams that could rub and cause blisters or sores.<br><br>- Wear proper fitting shoes - Break in new shoes gradually. Make sure shoes are not too tight and fit well. Check inside shoes before putting them on.<br><br>- Never walk barefoot - Wear shoes or slippers at all times to avoid injuring feet.<br><br>Checking your feet daily and seeing your doctor regularly are the best ways to stay on top of foot health with diabetes. Be very careful with minor foot wounds and let your doctor know right away if any problems arise. Healthy feet are happy feet!
**11. When should I see my health care provider about foot problems?**	As a diabetes patient, it is important to regularly inspect your feet and see your healthcare provider if you notice any problems. Some signs that you should make an appointment include:<br><br>- Changes in skin color or temperature of your feet. Redness, blisters, blue or black discoloration can indicate poor circulation, infection, or nerve damage. One foot feeling much warmer or colder than the other is also a concern.<br><br>- Numbness, tingling, pain, or burning in your feet. These sensations could be signs of nerve damage, which is common with diabetes. The nerve damage can worsen over time if blood sugars are not well controlled.

	- Ulcers, sores, cracks, or swelling of the skin. These may indicate a skin infection and need medical treatment to avoid complications like cellulitis or bone infections.<br><br>- Changes in shape of your feet. Bunions, hammertoes, and other deformities can develop due to nerve damage. Custom shoe inserts may help.<br><br>- Thick calluses or corns. These often form on points of friction or pressure on the feet. They need to be properly trimmed by a podiatrist.<br><br>- Ingrown toenails that are red and swollen. These are prone to infection.<br><br>- Slow healing of cuts or blisters on the feet. High blood sugar levels impair wound healing.<br><br>See your healthcare provider promptly if you notice any of these signs on your feet. Managing diabetes related foot problems early can prevent more severe complications like foot ulcers, infections, nerve damage and even amputation. Be vigilant about foot care.
**12. How common is diabetic eye disease?**	Diabetic eye disease refers to a group of eye conditions that can affect people with diabetes. It is a major cause of vision loss and blindness. About one third of people with diabetes have some form of diabetic eye disease. The longer someone has diabetes, the more likely they are to develop an eye condition.<br><br>The most common diabetic eye diseases are diabetic retinopathy, cataracts, and glaucoma. Diabetic retinopathy affects blood vessels in the retina at the back of the eye. It develops in stages and can eventually lead to blindness if left untreated. According to the National Eye Institute, around 7.7 million Americans aged 40 and older have diabetic retinopathy. It is the leading cause of blindness among working-age adults.<br><br>Cataracts, which cause the lens of the eye to become cloudy, also tend to develop at an earlier age and progress faster in people with diabetes. Cataracts develop in around 20% of people with diabetes. Glaucoma refers to a group of eye diseases that damage the optic nerve. People with diabetes are 40% more likely to develop glaucoma than the general public.

	Regular eye exams are crucial for detecting diabetic eye disease early on while treatment is most effective. Advances in eye surgery have also made it possible to successfully treat many cases of diabetic retinopathy and cataracts if caught early. Controlling blood sugar, blood pressure, and cholesterol can help reduce the risk of vision loss for people with diabetes.
**13. Who is more likely to develop diabetic eye disease?**	- People who have had diabetes for a long time - The risk of developing diabetic eye disease increases the longer someone has had diabetes. People who have had diabetes for over 10 years are at greater risk.<br><br>- People with poorly controlled blood sugar - High blood sugar levels over time can damage blood vessels in the eyes, increasing the risk of diabetic eye disease. Keeping blood sugar levels as close to normal as possible helps reduce risk.<br><br>- People with high blood pressure - Uncontrolled high blood pressure can damage blood vessels in the eyes and make diabetic eye disease worse. Managing blood pressure is important.<br><br>- Pregnant women with diabetes - Pregnancy can cause rapid changes in blood sugar levels which can advance diabetic eye disease. Close monitoring of blood sugar before and during pregnancy is recommended.<br><br>- People who smoke - Smoking constricts blood vessels, which can increase the risk of vascular damage in the eyes. Avoiding smoking is advised to lower risk.<br><br>- People with diabetic kidney disease - The same issues that damage blood vessels in the kidneys can also affect the tiny blood vessels in the eyes. Keeping kidneys healthy can help minimize risk.<br><br>The key messages are that the longer someone has had diabetes and the poorer their blood sugar control, the higher their risk of developing diabetic eye problems. But proper management of diabetes and related health issues can significantly reduce the risks. I hope this overview helps summarize the factors that public health experts look at regarding who is at increased risk. Let me know if you need any clarification or have additional questions!
**14. When should I see my health care provider about eye problems?**	As a diabetes specialist, I recommend that people with diabetes get a comprehensive dilated eye exam at least once a year. Diabetes can damage blood vessels in the retina at the back of the eye, leading to diabetic retinopathy, one of the leading causes of blindness. Many people with diabetic retinopathy have no symptoms at first. That's why regular eye exams are so important. They allow early detection and treatment before major vision loss occurs.

	During the eye exam, your eye care provider will dilate your pupils with eye drops and carefully examine your retina. They're looking for signs of diabetic retinopathy such as leaking blood vessels, retinal swelling, deposits in the retina, and damaged blood vessels. If any changes are found, they'll determine how severe the disease is and whether treatment is needed. Treatment options for more advanced retinopathy include injections into the eye, laser surgery, and vitrectomy surgery. With early detection and treatment, it's possible to prevent up to 98% of vision loss due to diabetic retinopathy.<br><br>In addition to at least annual exams, be sure to contact your eye doctor right away if you experience any new vision changes such as blurriness, floaters, missing areas of vision, eye pain, or trouble seeing colors properly. Diabetic retinopathy isn't the only eye condition related to diabetes. You're also at higher risk for cataracts and glaucoma. So prompt attention to any new eye symptoms is essential to protect your vision.
**15. What can I do to protect my eyes?**	As a diabetes patient, it is crucial to take steps to protect your eyes and vision. Diabetes can cause damage to the small blood vessels in the retina, leading to diabetic retinopathy, a serious eye condition that can result in vision loss or blindness if left untreated. The good news is that there are things you can do to minimize your risk.<br><br>First, maintain good blood sugar control. Work closely with your doctor to keep your A1C level (a measure of your average blood sugar over 3 months) as close to normal as possible. High blood sugar levels over time put you at greater risk for diabetic eye disease. Checking your levels regularly and taking medications as prescribed are key.<br><br>Second, get a dilated eye exam from an ophthalmologist or optometrist at least once a year. They will examine your retina for signs of damage. Early detection and timely treatment of diabetic retinopathy are essential to prevent vision loss. Report any vision changes, like blurred or patchy vision, to your doctor right away.<br><br>Third, manage other diabetes risk factors. Control your blood pressure and cholesterol, quit smoking if needed, and aim for a healthy weight. These steps will reduce risks throughout your body, including your eyes.<br><br>Fourth, eat a healthy diet full of leafy greens, vegetables, and lean protein. Key nutrients like vitamins C and E, omega-3 fatty acids, zinc, and antioxidants help maintain eye health.

	By controlling your diabetes, getting regular eye exams, and living an overall healthy lifestyle, you can significantly reduce the likelihood of vision problems down the road. Be proactive about protecting your eyesight.
**16. What if I already have some vision loss from diabetes?**	Diabetic retinopathy is a common complication of diabetes that can lead to vision loss if left untreated. If you already have some vision loss from diabetic retinopathy, the first step is to get a comprehensive eye exam to determine the extent of the damage. The ophthalmologist will look for signs of retinopathy progression, such as leaking blood vessels, swelling in the macula (the central part of the retina), or abnormal blood vessel growth.<br><br>The treatment recommendation will depend on the findings, but several options may help prevent further vision loss. Laser surgery can be used to seal leaking blood vessels and discourage abnormal vessel growth. Medications called vascular endothelial growth factor (VEGF) inhibitors may be injected into the eye to reduce swelling and leakage. With more advanced retinopathy, a vitrectomy surgery may be recommended, where the vitreous gel in the center of the eye is removed to eliminate traction on the retina.<br><br>The key is early detection and timely treatment. Even with existing vision loss, it is still possible to preserve remaining vision. Maintaining optimal blood sugar, blood pressure, and cholesterol levels is also important. Regular eye exams are crucial for people with diabetes, usually every 6-12 months. Vision loss is not inevitable, but preventing progression requires diligence and working closely with your ophthalmologist. Don't delay if you notice any new vision changes - early treatment is the best way to protect your sight.

Supplementary Table 1d: Claude+RISE Response to Diabetes-Related Questions

Question	Claude+RISE Response
**Topic: Concepts of Diabetes**	
**1. What Is Diabetes?**	Diabetes is a chronic disease that occurs when the pancreas does not produce enough insulin or when the body cannot effectively use the insulin it produces. Insulin is a hormone that regulates blood sugar. Hyperglycemia, or high blood sugar, is a common effect of uncontrolled diabetes and over time can cause serious damage to many of the body's systems, especially the nerves and blood vessels.<br><br>The most common types of diabetes are:<br><br>Type 1 diabetes - Usually diagnosed in children and young adults, the body does not produce insulin. It is an autoimmune disease where the immune system attacks the insulin-producing cells in the pancreas. Daily insulin injections or an insulin pump are required to regulate blood sugar levels.<br>Type 2 diabetes - The body does not produce enough insulin or cannot use it effectively. It typically develops in adults over the age of 45 who are overweight. However, due to increasing obesity rates, it is occurring more frequently in younger people. It can be managed through lifestyle changes like diet, exercise, and medication. Some may eventually need insulin therapy.<br>Gestational diabetes - A form of high blood sugar affecting pregnant women, it usually resolves after pregnancy. But, it increases the risk of developing type 2 diabetes later in life for both mother and child.<br>The complications from uncontrolled diabetes can be severe, including heart disease, nerve damage, kidney damage, eye damage, foot damage, skin conditions, hearing impairment, Alzheimer's disease, and more. Fortunately, the progression of diabetes can be slowed or prevented through healthy lifestyle habits. Controlling carbohydrate intake, staying active, maintaining a healthy weight, avoiding tobacco, and taking prescribed medications as directed are important self-care steps. Regular medical check-ups and screening tests are also essential for monitoring diabetes.
**2. How can I determine which type of diabetes I have?**	There are a few ways to determine which type of diabetes you have:<br><br>- Getting tested for diabetes antibodies and c-peptide levels can help distinguish between type 1 and type 2 diabetes. People with type 1 will have positive antibodies and low c-peptide, while those with type 2 will not have antibodies and will have normal or high c-peptide.

	- Looking at when symptoms started provides clues. Type 1 diabetes symptoms tend to come on quickly, while type 2 develops more gradually.<br><br>- Considering your age of onset is helpful. Type 1 most often occurs in children/teens, while type 2 is more common in adults over 40. However, type 2 is increasing in youth due to obesity.<br><br>- Gestational diabetes occurs during pregnancy, is diagnosed through prenatal screening, and usually goes away after giving birth.<br><br>- Your treatment needs also indicate diabetes type. People with type 1 absolutely require insulin treatment to survive. Many with type 2 can manage with lifestyle changes and oral meds initially.<br><br>The blood tests for antibodies and c-peptide are the most definitive way to diagnose type 1 vs type 2 diabetes. Be sure to see an endocrinologist who specializes in diabetes if the type is unclear. Getting the right diagnosis is key to receiving proper treatment and care. Let me know if you need any clarification or have additional questions!
**3. Who is more likely to develop type 1 diabetes?**	Children and young adults are more likely to develop type 1 diabetes compared to other age groups. The exact causes are still being researched, but genetics and family history seem to play a role, with having a close relative with type 1 diabetes increasing one's risk. Type 1 diabetes only accounts for about 5% of all diabetes cases in the United States, so it is much less common than type 2 diabetes overall. The onset of type 1 is usually abrupt and occurs due to autoimmune destruction of the insulin-producing beta cells in the pancreas, leading to insulin deficiency. So in summary, the populations most likely to develop type 1 diabetes are children, adolescents, and young adults, especially those with a family history of the disease. Lifestyle factors seem to play less of a role compared to type 2 diabetes.
**4. Who is more likely to develop type 2 diabetes?**	Some key factors that increase a person's risk of developing type 2 diabetes are:<br><br>- Age - The risk rises significantly at age 45 and older.<br><br>- Overweight/Obesity - Carrying excess weight increases risk, especially abdominal obesity. This is a leading risk factor.<br><br>- Race/Ethnicity - African Americans, Hispanics/Latinos, American Indians, Asian Americans and Pacific Islanders have a higher risk.

	- Family History - Having a close relative with type 2 diabetes increases your risk.<br><br>- Prediabetes - People with elevated but not yet diabetic blood sugar levels are at very high risk of developing type 2 diabetes.<br><br>- Gestational Diabetes - Women who had diabetes during pregnancy have an increased future risk.<br><br>- Physical Inactivity - Lack of exercise and sedentary lifestyle contributes to risk.<br><br>- High Blood Pressure - Having hypertension also raises your risk.<br><br>In summary, the most likely groups to develop type 2 diabetes are those over age 45, who are overweight/obese, physically inactive, have a family history of diabetes, or are of certain racial/ethnic backgrounds. The good news is that maintaining a healthy weight through diet and exercise can help prevent or delay onset of type 2 diabetes in many high risk individuals.
**Topic: Symptoms & Causes**	
**1. What are the symptoms of diabetes?**	Here is an overview of the common symptoms of diabetes:<br><br>- Increased thirst and frequent urination - High blood sugar leads to increased urination, causing dehydration and thirst. This is a classic early symptom of diabetes.<br><br>- Increased hunger - Despite eating more, the body can't properly utilize blood glucose so it continues sending hunger signals.<br><br>- Fatigue - The body is unable to convert glucose into energy, leading to exhaustion and tiredness.<br><br>- Blurred vision - Fluctuating blood sugar levels can cause blurred vision as fluid shifts in and out of the lens. This is often a temporary symptom.<br><br>- Tingling/numbness in hands and feet - Nerve damage from high blood sugar can cause numbness and tingling sensations. This is known as diabetic neuropathy.

	- Slow healing wounds/sores - Poor circulation from diabetes inhibits healing of cuts and sores.<br><br>- Unexplained weight loss - The inability to use glucose for energy forces the body to tap fat and muscle stores, leading to weight loss.<br><br>- Slow onset of symptoms in type 2 - Type 2 symptoms may take years to show and are often mild initially. Many have no symptoms at all.<br><br>- Rapid onset in type 1- Type 1 symptoms can progress very quickly, over weeks or months, and are often more severe.<br><br>Seeing a doctor quickly when these symptoms arise is important to get diagnosed and start proper diabetes management. Being aware of symptoms can help detect diabetes before complications occur.
**2. What causes type 1 diabetes?**	Type 1 diabetes is an autoimmune condition where the immune system mistakenly attacks the insulin-producing beta cells in the pancreas. This destroys the body's ability to produce insulin, the key hormone needed to regulate blood sugar.<br><br>The exact trigger for this autoimmune attack is still being researched, but both genetic and environmental factors are believed to play a role:<br><br>- Genetics: There are specific genes like HLA types that increase risk of developing autoimmunity against beta cells. However, not everyone with the risk genes ends up getting type 1 diabetes.<br><br>- Viral infections: Exposure to viruses like coxsackievirus, rubella, mumps, etc. may initiate the autoimmune response in genetically predisposed individuals.<br><br>- Other theories: Early exposure to cow's milk proteins, vitamin D deficiency, gut bacteria imbalances, and environmental toxins are also being investigated as potential triggers.<br><br>- The immune system mistake: For some reason, the immune system wrongly identifies the insulin-producing cells as foreign invaders and starts attacking them. This autoimmune reaction eventually destroys the beta cells.

	Research efforts like TrialNet are ongoing to better understand the exact causes and environmental factors that trigger this autoimmune destruction of beta cells. This can help develop ways to predict, prevent, and hopefully reverse type 1 diabetes in the future. But for now, we know it's caused by complex interactions between genes and environment.
**3. What causes type 2 diabetes?**	The main drivers of type 2 diabetes are a combination of lifestyle factors and genetics.<br><br>The most significant lifestyle factors are:<br><br>- Overweight and obesity - Carrying excess weight, especially in the abdominal area, is a primary cause. It contributes to insulin resistance and disrupts blood sugar control.<br><br>- Physical inactivity - Lack of exercise leads to weight gain and impair insulin function. Regular physical activity helps manage weight and improve insulin sensitivity.<br><br>- Diet - Diets high in processed, sugary foods and low in fiber increase weight gain and blood sugar spikes.<br><br>Genetic factors also raise the risk, even in those with healthy lifestyles:<br><br>- Family history - Having a parent, sibling or child with type 2 diabetes increases risk. Certain genes predispose people to insulin resistance and pancreatic cell issues.<br><br>- Ethnicity – African Americans, Hispanics/Latinos, American Indians and certain Asian populations have higher rates of type 2 diabetes, partially due to genetic susceptibility.<br><br>- Body fat distribution – People who carry extra weight around the abdomen and organs have more insulin resistance issues.<br><br>In summary, while genetics play a role, focusing on maintaining a healthy weight through diet and exercise is vital for reducing the likelihood of developing type 2 diabetes as well as managing the condition. Lifestyle changes and medication can help control type 2 diabetes and prevent complications.

**4. What causes gestational diabetes?**	- Genetics - Gene variants that predispose women to insulin resistance and reduced insulin secretion. Having a family history of type 2 diabetes increases risk. Certain ethnicities have higher risk due to genetic factors.<br><br>- Insulin resistance - Hormones produced by the placenta, especially in the later stages of pregnancy, can disrupt normal insulin signaling and lead to insulin resistance. This ensures adequate glucose for the developing baby.<br><br>- Being overweight or obese - Carrying excess weight, especially abdominal fat, prior to and during pregnancy contributes to insulin resistance.<br><br>- Weight gain during pregnancy - Excessive weight gain can worsen insulin resistance and increase risk of gestational diabetes.<br><br>- Advanced maternal age - Risk rises steadily after age 25 and is much higher over age 35. Older maternal age may be correlated with subtle impairments in insulin secretion.<br><br>- Prior gestational diabetes or delivering a large baby - Suggests a predisposition to abnormal glucose metabolism during pregnancy.<br><br>- Polycyctic ovary syndrome (PCOS) - Women with this endocrine disorder linked to insulin resistance have higher risk.<br><br>In summary, the main drivers are genetics, insulin blocking hormones produced during pregnancy, excess body weight, and older maternal age. Proper prenatal care and glucose testing can identify and manage gestational diabetes to avoid complications.
**Topic: Diabetes Tests & Diagnosis**	
**1. Who should be tested for diabetes?**	- Anyone experiencing symptoms of diabetes, such as increased thirst, frequent urination, unexplained weight loss, blurry vision, etc.<br><br>- People with risk factors for type 2 diabetes, including being overweight/obese, family history of diabetes, age 35+, history of gestational diabetes, and belonging to certain racial/ethnic groups. Testing should start by age 35 and be repeated every 3 years.<br><br>- Children and teens who are overweight/obese and have additional risk factors should be tested starting at age 10.

	- All pregnant women should be tested for gestational diabetes between 24-28 weeks of pregnancy. Those with gestational diabetes need follow-up testing for type 2 diabetes after delivery.<br><br>- Relatives of those with type 1 diabetes may consider antibody testing through TrialNet even without symptoms.<br><br>- People diagnosed with prediabetes need annual testing to monitor for progression to type 2 diabetes.<br><br>The criteria emphasize screening those at high risk due to obesity, family history, pregnancy-related factors, and other medical conditions like PCOS. Symptoms of diabetes also warrant immediate testing. Regular screening for those with risk factors or above threshold blood sugar levels is important for early diagnosis and prevention of complications. Please let me know if this summary appropriately captures the key groups that guidelines recommend be tested for diabetes.
**2. What tests are used to diagnose diabetes?**	- Fasting plasma glucose (FPG) test: Fasting for at least 8 hours and then measuring blood glucose levels. 126 mg/dL or higher indicates diabetes. This can detect both type 1 and type 2 diabetes.<br><br>- A1C test: Measures average blood glucose over the past 2-3 months. 6.5% or higher indicates diabetes. This test diagnoses both type 1 and type 2 diabetes, but may not be accurate in certain cases like anemia or hemoglobin variants.<br><br>- Random plasma glucose test: Checks blood glucose without fasting. 200 mg/dL or higher strongly suggests diabetes if symptoms like increased thirst and urination are also present. Can diagnose type 1 and 2.<br><br>- Oral glucose tolerance test (OGTT): Involves fasting overnight, then drinking a sugary drink. Blood glucose is measured periodically for 2-3 hours. Higher than normal levels indicate diabetes. Mainly used to detect type 2 diabetes and gestational diabetes.<br><br>- Glucose challenge test: For pregnant women to screen for gestational diabetes. Blood glucose measured 1 hour after drinking a sweet glucose drink. If level is too high, an OGTT is recommended.

	In summary, the FPG and A1C tests are most commonly used to screen for and diagnose both type 1 and type 2 diabetes. The OGTT provides additional diagnostic information when the initial results are ambiguous, and is commonly used to detect gestational diabetes.
**3. What test results tell me if I have diabetes or prediabetes?**	For a normal diagnosis:<br>- A1C test: Below 5.7%<br>- Fasting plasma glucose test: 99 mg/dL or below<br>- Oral glucose tolerance test: 139 mg/dL or below<br><br>For a prediabetes diagnosis:<br>- A1C test: 5.7% to 6.4%<br>- Fasting plasma glucose test: 100 to 125 mg/dL<br>- Oral glucose tolerance test: 140 to 199 mg/dL<br><br>For a diabetes diagnosis:<br>- A1C test: 6.5% or higher<br>- Fasting plasma glucose test: 126 mg/dL or higher<br>- Oral glucose tolerance test: 200 mg/dL or higher at the 2 hour mark<br>- Random plasma glucose test: 200 mg/dL or higher<br><br>In summary, the key thresholds are:<br>- A1C of 5.7% to 6.4% indicates prediabetes, 6.5% or above indicates diabetes<br>- Fasting plasma glucose of 100-125 mg/dL indicates prediabetes, 126 mg/dL or above indicates diabetes<br>- Oral glucose tolerance test of 140-199 mg/dL indicates prediabetes, 200 mg/dL or above indicates diabetes<br>- Random plasma glucose of 200 mg/dL or above indicates diabetes<br><br>Thank you for providing the additional reference, it helps confirm and refine the test result ranges for diagnosing prediabetes versus diabetes. Let me know if you need any clarification or have additional questions!

**4. Which tests help my doctor know what kind of diabetes I have?**	- Genetic testing: As you mentioned, this can diagnose monogenic diabetes caused by genetic mutations. It's especially useful for infants, teens, and young adults diagnosed with diabetes.<br><br>- Autoantibody tests: Testing for autoantibodies like anti-GAD, IAA, IA-2, and Znt8 can indicate type 1 diabetes if these autoantibodies are present. Even in those without diabetes, positive autoantibodies can signify increased type 1 diabetes risk.<br><br>- C-peptide levels: As I noted before, c-peptide levels help distinguish between type 1 (low c-peptide) and type 2 diabetes (normal to high c-peptide).<br><br>- Age of onset: Type 1 diabetes frequently develops in children and adolescents, while type 2 is more common in adults, though increasing in youth due to obesity. Age of onset provides a clue to diabetes type.<br><br>- BMI: Type 2 diabetes is strongly associated with excess weight and obesity. Checking BMI provides additional data to complement other test findings.<br><br>- Prior pancreatitis: Previous pancreatitis may indicate type 3c diabetes.<br><br>You're absolutely right that identifying the specific type of diabetes is crucial for determining the appropriate treatment regimen. The combination of genetic testing for monogenic diabetes, autoantibody presence, c-peptide levels, age of onset, BMI, medical history, and other clinical findings allows physicians to definitively classify the diabetes type. Thank you for the insightful additional information.
**Topic: Managing Diabetes**	
**1. How can I manage my diabetes?**	- Monitor your blood sugar regularly through home blood glucose testing. Check levels when you wake up, before meals, and at bedtime. Track results to identify patterns.<br><br>- Follow your doctor's medication regimen closely. Take medicines as prescribed and report any side effects. Medications help regulate blood sugar.<br><br>- Adopt a diabetes-friendly diet focusing on foods like fruits, vegetables, lean proteins, whole grains, and healthy fats. Avoid added sugars and refined carbs. Consult a dietitian if needed.

	- Incorporate regular exercise into your routine, aiming for 30 minutes of activity most days. Choose options like walking, swimming, or strength training. Being active helps control blood sugar.<br><br>- Reach and maintain a healthy weight through diet and exercise. Even modest weight loss can improve diabetes management.<br><br>- Quit smoking and avoid secondhand smoke, which exacerbate diabetes complications. Consider nicotine replacement options and support groups.<br><br>- Attend regular doctor's visits to monitor ABCs: A1C, blood pressure, cholesterol levels. Stay on top of screenings for diabetes-related health issues.<br><br>- Manage stress through techniques like meditation, yoga, or talking to a counselor. Stress influences blood sugar levels. Prioritize sleep as well.<br><br>- Check feet daily and see a podiatrist regularly to prevent foot injuries. Wear proper fitting shoes and socks.<br><br>- Create a support network of health providers, family, and friends to help manage diabetes daily. Join a local diabetes community group too.<br><br>The key is making lifestyle changes, sticking to your treatment plan, monitoring your health, and getting support. Let me know if you have any other questions!
**2. What are the recommended targets for blood glucose levels?**	- Fasting/Pre-meal: 80-130 mg/dL<br>- 2 Hours Post-meal: Less than 180 mg/dL<br><br>The pre-meal target you referenced of 80-130 mg/dL aligns with recommendations from the American Diabetes Association and American Association of Clinical Endocrinologists.<br><br>The post-meal target of under 180 mg/dL is also consistent with guidelines. This reinforces that after starting a meal, blood glucose levels should ideally rise to less than 180 mg/dL within a 2 hour window.

	These are good general targets, but optimal ranges can vary individually based on factors like age, diabetes type, duration of disease, risk of hypoglycemia, and other medical considerations. That's why it's important to discuss personalized targets with your healthcare provider.<br><br>Consistently staying in your target range, along with medication, lifestyle changes, and ongoing medical care, can help manage diabetes and prevent complications over time. Let me know if you need any clarification or have additional questions on recommended blood glucose targets!
**3. What medicines might I take for diabetes?**	The medications used to treat diabetes depend on the type of diabetes. For type 1 diabetes, insulin is the mainstay of treatment. There are various types of insulin that differ in how quickly they start working and how long they last in the body. Insulin helps get glucose from the bloodstream into the cells for energy.<br><br>For type 2 diabetes, metformin is usually the first oral medication prescribed. It lowers blood glucose levels by decreasing glucose production in the liver and improving insulin sensitivity. Other common oral medications for type 2 diabetes include sulfonylureas like glipizide and glyburide, which increase insulin secretion from the pancreas; DPP-4 inhibitors like sitagliptin, which regulate insulin and glucagon secretion; GLP-1 receptor agonists like liraglutide, which stimulate insulin release and suppress appetite; and SGLT2 inhibitors like canagliflozin, which lower blood glucose by causing the kidneys to remove excess glucose in urine.<br><br>The choice of medication depends on factors like the severity of hyperglycemia, other medical conditions, side effects, costs and insurance coverage. Lifestyle modifications like diet, exercise and weight loss are also important components of diabetes management. It's best to work with a healthcare provider to determine the most appropriate medications based on your individual needs and circumstances. The goal is to effectively control blood glucose levels and prevent complications of diabetes.
**4. What are the different types of insulin?**	- Rapid-acting insulin (lispro, aspart, glulisine) - Onset within 15 minutes, peaks at 1 hour, lasts 2-4 hours<br><br>- Inhaled rapid-acting insulin - Onset 10-15 minutes, peaks at 30 minutes, lasts 3 hours<br><br>- Short-acting (regular) insulin - Onset 30 minutes, peaks at 2-3 hours, lasts 3-6 hours<br><br>- Intermediate-acting (NPH) insulin - Onset 2-4 hours, peaks at 4-12 hours, lasts 12-18 hours<br><br>- Long-acting insulin (detemir, glargine) - Onset 2 hours, no peak, lasts approximately 24 hours

	- Ultra long-acting insulin - Onset 6 hours, no peak, lasts 36+ hours<br><br>The key distinctions are the onset time, presence of a peak, and total duration. This helps determine the appropriate insulin type to match an individual's needs and lifestyle patterns. The goal is to effectively cover both basal insulin needs as well as prandial (mealtime) needs. Let me know if you would like me to elaborate on usage recommendations for any of the specific insulin types. I'm happy to provide more details on optimizing insulin therapy for diabetes management.
**5. What are the different ways to take insulin?**	- Insulin pumps - These small, wearable devices deliver insulin continuously under the skin through a catheter and can be programmed to meet individual dosage needs.<br><br>- Inhaled insulin - Rapid-acting insulin powders that are inhaled through specialized inhaler devices. Absorbed quickly but may not suit all patients.<br><br>- Jet injectors - Use high pressure instead of a needle to inject insulin through the skin. Less common but helpful for needle-phobic patients.<br><br>- Artificial pancreas systems - Combination of continuous glucose monitor, control algorithm software, and insulin pump that automatically adjusts insulin dosing. Helps manage blood sugar levels.<br><br>- Oral medications - While not insulin, some non-insulin pills and tablets can help utilize insulin better or stimulate pancreatic insulin production.<br><br>- Transdermal patches - Still in research, but aim to deliver insulin through microscopic needles in a patch. Not approved yet.<br><br>The choice of delivery method depends on the individual's preferences, lifestyle, costs, age, and other medical conditions. Consulting a physician helps determine the optimal administration route. Regular monitoring of blood glucose levels is important for all methods to ensure safe, effective dosing. Let me know if you need any clarification or have additional questions!
**6. What oral medicines treat type 2 diabetes?**	Here is a summary of key oral medications used to treat type 2 diabetes:<br><br>- Metformin, as you mentioned, is usually the first line medication prescribed. It works by reducing liver glucose production and improving insulin sensitivity.

	- Sulfonylureas like glipizide stimulate the pancreas to release more insulin. They carry a risk of hypoglycemia.<br><br>- DPP-4 inhibitors like sitagliptin block breakdown of incretin hormones, increasing insulin secretion after meals. Low risk of low blood sugar.<br><br>- SGLT2 inhibitors like empagliflozin prevent kidney reabsorption of glucose, lowering blood sugar. They may aid weight loss.<br><br>- Meglitinides like repaglinide also stimulate insulin release but work faster and for a shorter time than sulfonylureas. Also risk hypoglycemia.<br><br>- Thiazolidinediones like pioglitazone improve insulin sensitivity in muscles and fat. Can cause fluid retention in some.<br><br>- Combination pills with metformin plus another class can improve blood sugar control compared to metformin alone.<br><br>You're right that combining oral medications can be more effective than monotherapy. Lifestyle modifications like diet, exercise, and blood sugar monitoring are still important parts of type 2 diabetes management along with medications. Let me know if you need any clarification!
**7. What should I know about side effects of diabetes medicines?**	- Hypoglycemia (low blood sugar) is a common side effect to watch out for with some diabetes medicines like insulin and sulfonylureas. It's important to balance these medicines with food intake and activity.<br><br>- Other potential side effects of diabetes drugs can include nausea, diarrhea, bloating, weight gain, headaches, and rashes. These vary depending on the medication.<br><br>- Always take your diabetes medications exactly as prescribed by your doctor. This can help minimize side effects.<br><br>- Tell your doctor right away about any side effects you experience after starting a new diabetes medication. They may be able to adjust the dosage or switch you to an alternative drug to reduce side effects.<br><br>- Never stop or change how you take your diabetes medicines without consulting your doctor first. This could lead to complications.

	- Be sure to get regular blood tests to monitor blood sugar levels and kidney function. This helps ensure your medication regimen is working properly.<br><br>- Ask your doctor or pharmacist if you have any questions about potential side effects of your diabetes medications. They can provide guidance on managing them.<br><br>Let me know if you need any clarification or have additional questions! As a public health expert, I'm happy to provide evidence-based information about diabetes care and potential medication side effects.
**8. What foods can I eat if I have diabetes?**	- Eat plenty of non-starchy vegetables like broccoli, carrots, leafy greens, peppers and tomatoes. They provide nutrients without spiking blood sugar.<br><br>- Choose whole fruit in moderation, like berries, apples, oranges, and melons. Avoid fruit juice.<br><br>- Opt for whole grains like whole wheat bread, oats, quinoa, and brown rice. Aim for at least half your grains to be whole grains.<br><br>- Include lean protein like chicken, fish, eggs, beans, lentils, nuts, seeds and tofu. These foods are filling and help control blood sugar.<br><br>- Choose low-fat or nonfat dairy like milk, yogurt and cheese. If lactose intolerant, try lactose-free options.<br><br>- Limit starchy vegetables like potatoes, corn, and peas as well as refined grains like white bread, pasta and rice. Eat smaller portions of these.<br><br>- Use healthy oils like olive, canola and avocado oil instead of butter or lard when cooking. Also eat avocados, nuts, seeds and fatty fish.<br><br>- Drink lots of water and limit sugary drinks like juice and soda.<br><br>- Follow portion sizes on your individualized meal plan. Moderation and balance are key.<br><br>The overall guidelines are to eat a balanced diet with a variety of nutrient-dense foods. Work closely with your healthcare team to develop the optimal diabetes eating plan for your needs and preferences.

**9. What foods and drinks should I limit if I have diabetes?**	Here is a summary of foods and drinks people with diabetes should limit:<br><br>- Fried foods and foods high in saturated or trans fats - these can raise LDL ("bad") cholesterol levels.<br><br>- Foods with high sodium content, like salty snacks, canned soups, and processed meats - excess sodium can increase blood pressure.<br><br>- Sweets like baked goods, candy, ice cream - these spike blood sugar and provide empty calories.<br><br>- Sugary drinks like juice, regular soda, sports drinks - drink water or unsweetened beverages instead.<br><br>- Alcohol - if consumed, limit to 1 drink daily for women, 2 for men. Be cautious if using insulin or meds that increase insulin, as alcohol can cause hypoglycemia. Eat food when drinking.<br><br>The key guidelines are to minimize foods high in sugar, unhealthy fats, and sodium. Focus on whole, nutrient-dense foods. Limit sweets and sugary drinks and be cautious with alcohol intake. Following a healthy diabetes diet helps manage blood sugar and prevent complications. Let me know if you need any clarification or have additional diet questions!
**10. When should I eat if I have diabetes?**	Here are some key tips on meal timing for people with diabetes:<br><br>- If you take insulin or other diabetes medication that requires consistent carb intake, eat at the same times daily and space carb amounts evenly. Skipping or delaying meals can lead to low blood sugar.<br><br>- If you take "mealtime" insulin, your eating schedule can be more flexible, but still aim to eat consistent carb amounts day-to-day.<br><br>- Check with your healthcare provider about eating schedules, carb amounts, and meal timing in relation to physical activity. Activity can affect blood sugar levels.<br><br>- People using insulin or insulin-stimulating meds should not go more than 5-6 hours without eating a meal or snack to prevent hypoglycemia.

	- Eat breakfast within 1-2 hours of waking to replenish blood sugar. Do not skip breakfast.<br><br>- Have a bedtime snack if directed by your doctor to prevent overnight hypoglycemia, especially if on insulin.<br><br>In summary, meal timing for diabetes depends on your medications and insulin routine. Check with your healthcare team for personalized guidance on the optimal eating schedule for your diabetes management. Let me know if you need any clarification!
**11. How much can I eat if I have diabetes?**	Here are the key takeaways:<br><br>- Work with your healthcare team to determine optimal calorie needs and carbohydrate amounts for your meal plan.<br><br>- Use the plate method - fill half your plate with non-starchy vegetables, a quarter with lean protein, and a quarter with high-fiber carbs.<br><br>- Practice portion control using your hands or common objects to estimate serving sizes. Limit added sugars and refined grains.<br><br>- Carb counting involves tracking your daily carb grams from foods to manage blood sugar. It works well for those on insulin.<br><br>- Visit a dietitian for personalized medical nutrition therapy to develop your diabetes eating plan.<br><br>- Focus on getting carbohydrates from fruits, vegetables, whole grains, beans, and low-fat dairy rather than added sugars or refined grains.<br><br>- Don't forget to incorporate regular physical activity into your weight and diabetes management plan.<br><br>You're absolutely right that portion control and mindful carb counting are key for balancing blood sugar levels and weight when eating with diabetes. Let me know if you need any clarification or have additional questions!

**12. Will supplements and vitamins help my diabetes?**	- There is no clear proof that taking supplements like vitamins, minerals, herbs or spices can help manage diabetes. While some individual studies may show benefits, the overall evidence is inconclusive.<br><br>- Supplements are not a substitute for standard diabetes treatment and lifestyle changes recommended by your doctor. Things like maintaining a healthy weight, eating a balanced diet, exercising regularly and taking medication as prescribed have much stronger evidence for helping manage diabetes.<br><br>- Check with your doctor or dietitian before taking any supplements. Some can interact with diabetes medications or cause side effects.<br><br>- You may need supplements if your diet is lacking in certain vitamins and minerals. Your doctor can advise if you need a supplement and which ones to take. But eating a varied, healthy diet should provide all the nutrients you need.<br><br>- Overall, dietary supplements should not be relied upon to manage diabetes. Focus instead on proven lifestyle measures and work with your healthcare team to find the most effective medical treatment for your individual situation. While some supplements are safe, they are not a substitute for standard diabetes care.
**13. Why should I be physically active if I have diabetes?**	Regular physical activity is extremely important for people with diabetes. Exercise helps improve blood sugar control, reduces cardiovascular risk factors, contributes to weight loss, and provides many other health benefits. For these reasons, being physically active should be a vital part of managing diabetes.<br><br>First, exercise directly impacts blood glucose levels. Physical activity triggers glucose uptake from the bloodstream, leading to lower blood sugar. Even mild to moderate exercise like brisk walking can significantly lower blood glucose for 24 hours or more afterwards. Strength training also builds muscle mass so the body is better able to take up glucose.<br><br>Second, physical activity helps reduce cardiovascular risks - high blood pressure, cholesterol, obesity - often associated with diabetes. Exercise improves blood flow, strengthens the heart and lungs, burns calories to facilitate weight loss, and lowers blood pressure. Staying active is critical for reducing heart disease and stroke risk.<br><br>Third, exercise aids weight loss and maintaining a healthy weight, which is key for diabetes management. Carrying excess weight makes cells more resistant to insulin. Combining physical activity with a reduced-calorie diet can lead to greater benefits. Even small amounts of activity can help. Experts recommend 30-60 minutes of moderate to vigorous activity 5 days per week for weight control.

	In addition to better blood sugar regulation, physical activity provides many other benefits like improved mood, better sleep, increased mobility and balance in older adults. Overall, regular exercise is vitally important for people with diabetes. Along with proper medication and nutrition, it helps manage blood sugar, reduces cardiovascular risk, facilitates weight loss, and improves overall health. Aim for at least 30 minutes of moderate activity most days, and consult a healthcare provider before starting any new routine.
**14. How can I be physically active safely if I have diabetes?**	Regular physical activity is important for managing diabetes, but some precautions should be taken. First, check your blood sugar before, during, and after exercising. This helps you monitor levels and treat lows quickly with a fast-acting carbohydrate if needed. Wear proper footwear to prevent blisters or sores that could become infected. Stay hydrated by drinking water before, during, and after activity to prevent dehydration which can affect blood sugar. Know the signs of low blood sugar like shakiness or sweating and stop to treat if they occur.<br><br>Gradually build up activity and avoid extreme temperatures that can cause blood sugar fluctuations. Start with low-impact exercises like walking or swimming. Listen to your body and don't overexert yourself. Choose activities you enjoy so you stay motivated. Discuss any limitations or appropriate activities with your doctor beforehand.<br><br>When taking insulin, coordinate doses with meals and activity to prevent hypoglycemia. You may need a small carbohydrate snack before, during or after intense activity. Plan ahead and check blood sugar frequently around exercise. Preventing lows is crucial.<br><br>Take care of your feet by wearing supportive shoes and inspecting for blisters or sores. This prevents infections. Stay hydrated with water before, during, and after activity. If you take insulin, ketone levels should be monitored with hyperglycemia; avoid intense activity when ketones are present.<br><br>With some caution, those with diabetes can safely enjoy regular exercise. Checking blood sugar, preventing lows, wearing proper footwear, staying hydrated, knowing warning signs, gradually increasing activity, and discussing plans with your doctor will allow you to be active while managing diabetes.
**15. What physical activities should I do if I have diabetes?**	Regular physical activity is important for managing diabetes, but some precautions should be taken. First, check your blood sugar before, during, and after exercising. This helps you monitor levels and treat lows quickly with a fast-acting carbohydrate if needed. Wear proper footwear to prevent blisters or sores that could become infected. Stay hydrated by drinking water before, during, and after activity to prevent dehydration which can affect blood sugar. Know the signs of low blood sugar like shakiness or sweating and stop to treat if they occur.

	Gradually build up activity and avoid extreme temperatures that can cause blood sugar fluctuations. Start with low-impact exercises like walking or swimming. Listen to your body and don't overexert yourself. Choose activities you enjoy so you stay motivated. Discuss any limitations or appropriate activities with your doctor beforehand.<br><br>When taking insulin, coordinate doses with meals and activity to prevent hypoglycemia. You may need a small carbohydrate snack before, during or after intense activity. Plan ahead and check blood sugar frequently around exercise. Preventing lows is crucial.<br><br>Take care of your feet by wearing supportive shoes and inspecting for blisters or sores. This prevents infections. Stay hydrated with water before, during, and after activity. If you take insulin, ketone levels should be monitored with hyperglycemia; avoid intense activity when ketones are present.<br><br>With some caution, those with diabetes can safely enjoy regular exercise. Checking blood sugar, preventing lows, wearing proper footwear, staying hydrated, knowing warning signs, gradually increasing activity, and discussing plans with your doctor will allow you to be active while managing diabetes.
**Topic: Preventing Diabetes Problems**	
**1. How can I lower my chances of developing type 2 diabetes?**	The most effective way to reduce your chances of developing type 2 diabetes is to lose weight if you are overweight or obese. Losing just 5-7% of your starting weight can significantly lower your risk. To lose weight, focus on reducing your calorie intake while increasing physical activity. Eat smaller portions of foods and choose healthier options like vegetables, fruits, whole grains, lean proteins and healthy fats. Avoid high-calorie foods with added sugars, saturated fats and refined grains. Engage in moderate physical activity for at least 30 minutes per day, 5 days per week. Walking briskly, swimming and cycling are great options.<br><br>In addition to weight loss, building regular exercise into your daily routine is also important for diabetes prevention. Aim for 150 minutes per week of moderate activity like brisk walking. This can help control weight and improve insulin sensitivity even without major weight loss.<br><br>Eating a balanced, healthy diet is also beneficial. Emphasize vegetables, fruits, whole grains, beans, lentils, nuts, seeds and lean proteins like fish and poultry. Limit highly processed foods, especially those with added sugars. Portion control is also key. Consuming too many calories can lead to weight gain and interfere with blood sugar regulation.

	Other lifestyle changes like quitting smoking, limiting alcohol intake, and managing stress can also lower diabetes risk. Work on establishing healthy habits you can maintain long-term. Small, sustainable changes to your daily routine can significantly reduce your chances of developing type 2 diabetes over time. Consult with your doctor for personalized recommendations on managing your risk.
**2. What should I do if my health care professional told me I have prediabetes?**	Prediabetes means your blood sugar levels are higher than normal but not yet high enough to be diagnosed as type 2 diabetes. About 1 in 3 Americans have prediabetes, so getting diagnosed is a wake up call allowing you to take steps to prevent or delay developing full blown diabetes. Having prediabetes raises your chance of getting type 2 diabetes, so take it very seriously and make lifestyle changes under your doctor's guidance. The most important thing is to lose weight if overweight, follow an overall healthy diet focused on whole foods like vegetables, fruits, whole grains, lean proteins and healthy fats while avoiding processed foods and sugary drinks that spike blood sugar. Aim for 7-8 hours of sleep per night. Become more physically active with at least 30 minutes of brisk walking, swimming or strength training daily to help control weight and improve insulin sensitivity. Stop smoking and limit alcohol. Your provider may prescribe metformin or other medications to manage blood sugar levels. They'll monitor your A1C level, which is a 3-month blood sugar average, to see if it's decreasing. Studies show weight loss, healthy diet and exercise can return prediabetes to normal levels. So take advantage of this early warning to make changes now and prevent type 2 diabetes down the road. Lifestyle change programs through the CDC's National Diabetes Prevention Program can provide more support.
**3. If I had gestational diabetes when I was pregnant, how can I lower my chances of developing type 2 diabetes?**	Gestational diabetes puts you at high risk for developing type 2 diabetes later, so it’s crucial to take preventive steps. Getting tested 6-12 weeks after delivery is important - if blood glucose is still elevated, you may have type 2 diabetes and need treatment. If it's normal, get re-tested every 3 years to catch any progression early.<br><br>Lifestyle changes are key to lowering your chances of developing type 2 diabetes after gestational diabetes. First, focus on losing any pregnancy weight through healthy eating and regular exercise. Get 150 minutes per week of brisk walking or other moderate activity, and include strength training 2-3 times a week. Follow a balanced, low-fat, high fiber diet emphasizing whole grains, fruits, vegetables, lean proteins, and healthy fats. Limit sweets and refined carbs.<br><br>Breastfeeding can also help you shed pounds and control blood sugar. So breastfeed your baby if possible. For your child, breastfeeding provides ideal nutrition and may reduce their risk of obesity and diabetes later in life.<br><br>Additionally, ask your doctor about taking metformin. Metformin is a diabetes medication that helps control blood sugar levels and may prevent progression to type 2 diabetes in women who had gestational diabetes.

	Making positive lifestyle changes together and seeking preventive care benefits the whole family. Your healthy habits set an example and provide your child the best chance to grow up free of obesity and diabetes. Staying at a healthy weight and active now goes a long way for your future health and your child's too. Partner with your doctor on screening and medication options like metformin as well. With commitment to prevention, your chances of developing type 2 diabetes after gestational diabetes can be significantly lowered.
**4. What health problems can people with diabetes develop?**	People with diabetes are at risk for developing a number of health problems over time, especially if blood glucose levels remain consistently high. The best way to prevent many diabetes complications is to properly manage blood sugar, blood pressure, and cholesterol.<br><br>Uncontrolled diabetes can damage blood vessels and nerves throughout the body. This can eventually lead to problems with the heart, kidneys, feet, and eyes - some of the body's most important and delicate organs.<br><br>For example, persistently high blood glucose causes injury to blood vessels in the heart and interferes with circulation. This raises the risk of cardiovascular diseases, including heart attack, stroke, and coronary artery disease. Kidney disease (diabetic nephropathy) is also common, as elevated blood sugar damages the tiny blood vessels that filter waste from the blood.<br><br>Nerve damage (diabetic neuropathy) is another complication, often affecting the legs and feet first. Numbness, pain, and reduced circulation cause foot injuries to go unnoticed, leading to foot ulcers, infections, and potential amputation. Retinopathy affects the small blood vessels in the retina and is a leading cause of blindness.<br><br>The good news is that diligent diabetes management can prevent or delay the onset of complications. Checking blood sugar levels routinely, taking medications as prescribed, maintaining healthy blood pressure and cholesterol levels, having eye exams, inspecting feet daily, and visiting healthcare providers regularly are important ways to minimize diabetes-related health problems. Leading an overall healthy lifestyle with exercise and a balanced diet while avoiding tobacco can also make a major difference in managing diabetes and staying healthy.<br><br>With proper care and attention, people with diabetes can avoid or delay the most serious consequences of uncontrolled blood sugar. Work closely with your healthcare team and be proactive about your health. Taking steps today helps ensure a healthier future.

**5. How can I lower my chances of a heart attack or stroke if I have diabetes?**	If you have diabetes, controlling blood sugar, blood pressure, and cholesterol is critical to reducing your risk of heart attack and stroke. Work closely with your doctor and take all prescribed medications as directed. Follow a heart-healthy diet low in salt, unhealthy fats, and added sugars. Stay active with at least 30 minutes per day of moderate exercise like brisk walking.<br><br>Importantly, know the signs of a heart attack or stroke and call 911 immediately if you experience any. Heart attack signs include chest pain or discomfort; pain in the arms, back, neck or jaw; shortness of breath; sweating; nausea. Stroke signs include numbness or weakness on one side; confusion; trouble speaking; dizziness; vision issues; severe headache. Women may experience more subtle heart attack symptoms like fatigue. Diabetes can mask chest pain. Don't delay - call 911 at any warning signs.<br><br>Getting emergency treatment quickly can greatly improve outcomes and prevent permanent damage. Emergency responders can start life-saving medications right away. At the hospital, you may receive clot busting drugs for stroke or procedures to open blocked arteries for heart attack.<br><br>You can also help prevent emergencies by checking blood pressure often and taking medications to keep levels below 140/90 mmHg. Follow your doctor's advice for managing cholesterol. Don't smoke, limit alcohol, and find ways to manage stress. Connecting with loved ones provides emotional support. Consider mind-body practices like yoga or meditation as well.<br><br>Small daily steps to control risk factors really add up. Partner with your healthcare providers. Speak up about any new symptoms or concerns. With proper management, those living with diabetes can significantly reduce their risk of cardiovascular events.
**6. What are the warning signs of heart attack and stroke?**	Heart attacks and strokes require emergency care. If you experience any of the following signs, call 9-1-1 right away:<br><br>For a heart attack, warning signs can include chest pain or tightness that lasts more than a few minutes or comes and goes, pain or discomfort in one or both arms, back, neck, or jaw, shortness of breath, nausea, fatigue, and lightheadedness. Women may also experience extreme fatigue, pain that spreads to the back and neck, and nausea without chest pain. Those with diabetes may have no chest pain at all due to nerve damage. If you have a history of angina, seek medical care promptly if symptoms occur.

	With strokes, signs are sudden numbness or weakness on one side of the body, trouble speaking or understanding speech, vision problems in one or both eyes, dizziness, loss of balance in walking, and severe headache.<br><br>Every minute counts when treating heart attacks and strokes. Calling 911 instead of driving yourself or having someone else drive you allows emergency personnel to begin life-saving treatment right away. The fastest treatment leads to the best outcomes.<br><br>You can reduce your risk of heart attack and stroke by knowing your risk factors and making lifestyle changes. Risk factors include high blood pressure, cholesterol, smoking, obesity, inactivity, diabetes, and family history. Quitting smoking, eating healthier, exercising more, and following doctor's orders for conditions like high blood pressure and diabetes will lower your chances of an emergency.<br><br>Being aware of warning signs and calling 911 promptly can prevent permanent disability or death. If you experience any concerning symptoms, take rapid action. Do not wait to see if they will pass. With heart attacks and strokes, lost time means lost heart and brain function. Calling 911 at the first sign of a problem provides you the highest chance of full recovery.
**7. What causes low blood glucose in people with diabetes?**	There are several potential causes of low blood glucose, also called hypoglycemia, in people with diabetes. Some key causes include:<br><br>- Taking too much insulin or certain oral diabetes medications like sulfonylureas and meglitinides - These medicines directly act to increase insulin levels and can cause blood glucose to drop too low if the dose is too high, especially relative to food intake and activity levels.<br><br>- Delaying or missing meals - Most insulin and diabetes pill regimens are calibrated based on regular meal timing and carbohydrate intake. Skipping or delaying meals leads to less carbohydrate consumption, which results in less glucose entering the bloodstream. This disrupts the normal glucose balance and can lead to hypoglycemia.<br><br>- Increasing physical activity beyond usual levels - Exercise and activity increase the body's glucose utilization, which can significantly lower blood glucose. This risk can persist for up to 24 hours after the activity.<br><br>- Consuming alcohol without enough food - Alcohol inhibits the liver's glucose production and can enhance insulin absorption from medications, causing glucose levels to fall.

	- Fasting for medical procedures - Continuing to take insulin or diabetes medications while fasting for procedures can readily lead to low glucose levels.<br><br>- Certain medical conditions like kidney, liver or adrenal gland dysfunction - These conditions can impair the body's ability to regulate glucose properly.<br><br>- Interactions with other medications - Certain pills besides diabetes medicines can also increase hypoglycemia risk.<br><br>The key is to monitor blood glucose regularly, coordinate food, activity and medication dosing, and avoid triggers like missed meals. Reporting low glucose levels to doctors can help identify needs to adjust medication doses or the treatment regimen.
**8. How can I prevent low blood glucose if I have diabetes?**	Managing blood sugar levels with diabetes requires diligent monitoring, balanced nutrition, and careful medication management. I recommend taking several proactive steps to avoid hypoglycemia.<br><br>First, check your blood glucose regularly using a standard glucose meter or continuous monitor. This allows you to catch declining levels before they drop too low. Work with your doctor to determine optimal testing frequency - this may be more often if you have hypoglycemia unawareness.<br><br>Second, plan meals that provide consistent carbohydrates paired with protein and fat. Avoid long stretches without food. Carry quick-acting glucose sources like juice or tablets in case levels fall when away from meals. If consuming alcohol, pair it with food to maintain stability.<br><br>Third, when exercising, check glucose levels beforehand and consume additional carbohydrates if needed. Carry glucose tabs with you to treat lows. Adjust insulin dosages and food intake to account for increased activity. Speak to your healthcare provider about appropriate modifications to make.<br><br>Fourth, learn how your medications impact blood sugar, particularly if you take insulin or other glucose-lowering drugs. Work with your doctor to alter dosages to prevent hypoglycemia, especially if lows occur regularly. Never skip or reduce medication without medical supervision.<br><br>Fifth, get a continuous glucose monitor if recommended by your doctor, as this can alert you to decreasing glucose levels. Partner with your healthcare team to optimize device settings and response plans.

	Preventing hypoglycemia requires vigilance, but is very possible with self-monitoring, balanced nutrition, cautious physical activity, proper medication use, and guidance from your medical team. Prioritize keeping your blood sugar levels stabilized within your target range.
**9. How can I keep my kidneys healthy if I have diabetes?**	If you have diabetes, the most important steps for keeping your kidneys healthy are controlling your blood glucose levels and blood pressure. Work closely with your healthcare team to keep your A1C under 7% or as close to normal as possible through regular blood glucose monitoring, following your prescribed diabetes treatment plan, and making lifestyle changes like adopting a healthy diet, exercising regularly, and losing weight if needed.<br><br>It's also crucial to keep your blood pressure below 130/80 or your doctor's recommended goal. Medications like ACE inhibitors and ARBs help protect the kidneys by lowering blood pressure. Getting annual kidney function tests allows early detection and treatment of any kidney issues.<br><br>In addition to medications, you can help control blood pressure through healthy lifestyle habits - limiting sodium to under 2,300mg per day, staying hydrated, restricting alcohol, stopping smoking, and getting adequate sleep.<br><br>Diabetes medications including SGLT2 inhibitors and GLP-1 agonists have been shown to help prevent further kidney decline. Ask your doctor if these could be beneficial options for you.<br><br>Careful management of cholesterol, triglycerides, and other factors like stopping smoking and limiting alcohol will also help reduce kidney damage from diabetes.<br><br>The bottom line is diligently monitoring your blood glucose and BP levels, taking medications as prescribed, making healthy lifestyle choices, and working closely with your healthcare team can go a long way in preserving kidney function and avoiding further complications. Be vigilant with your diabetes self-care.
**10. What can I do to keep my feet healthy?**	Keeping your feet healthy is crucial for people with diabetes. High blood sugar can cause nerve damage that reduces sensation in the feet, making you less able to feel cuts or sores. Follow these tips:<br><br>- Check your feet every day - Look for cuts, blisters, redness or swelling. Inspect between the toes and undersides too. Use a mirror if needed. Call your doctor about any irritation that doesn't start to heal within a couple days.

	- Wash feet daily - Use warm, not hot, water and mild soap. Don't soak feet. Dry thoroughly, especially between the toes.<br><br>- Moisturize feet - Apply a thin layer of fragrance-free moisturizer on tops and bottoms to prevent cracking. Don't moisturize between toes.<br><br>- Trim toenails carefully - Cut straight across to avoid ingrown toenails. Smooth edges with an emery board.<br><br>- Wear clean, dry socks - Change socks daily. Avoid those with thick seams that could cause blisters or sores.<br><br>- Wear proper fitting shoes - Break in new shoes slowly. Ensure shoes fit well and aren't too tight. Check inside before putting them on.<br><br>- Never walk barefoot - Always wear shoes or slippers to avoid injuring feet.<br><br>- Keep blood circulating - Put feet up when sitting. Wiggle toes and move ankles up/down and in/out. Don't wear tight socks. Be physically active.<br><br>- Protect feet - Wear shoes at the beach, on hot pavement. Don't use heating pads. Wear warm boots in winter.<br><br>- Get regular foot exams - At least yearly, plus every doctor visit if you've had prior foot problems.<br><br>Checking feet daily and seeing your doctor regularly are the best ways to maintain foot health with diabetes. Stay vigilant about minor foot wounds. Healthy feet are happy feet!
**11. When should I see my health care provider about foot problems?**	As a person with diabetes, it is crucial to monitor your feet closely and contact your healthcare provider promptly if you notice any problems. Some key signs to watch for that may require medical attention include:<br><br>- Cuts, blisters, or bruises on your feet that do not start to heal after a few days. These minor wounds can lead to serious infections if not treated early, so call your provider if they are slow to heal.

	- Red, warm, or painful skin on your feet, which could signal a possible infection. Infections can spread quickly in someone with diabetes.<br><br>- Calluses with dried blood inside them, as these may be the first visible sign of an underlying wound. Calluses should be properly trimmed by a podiatrist.<br><br>- Foot infections that turn black and smelly, which are signs of gangrene. This is a medical emergency requiring immediate care.<br><br>- Numbness, tingling, burning, or pain in the feet, which may indicate nerve damage. If ignored, this can worsen over time.<br><br>- Changes in foot shape, skin color, temperature, or sensations. Your provider can assess if these are related to diabetes complications.<br><br>- Ulcers, cracks, swelling, thick calluses, ingrown toenails, or slow wound healing.<br><br>If you notice any of these warning signs on your feet, promptly contact your healthcare provider. Ask for a referral to a podiatrist if specialized foot care is needed. Managing diabetes foot problems early is crucial to prevent severe complications like infections, ulcers, nerve damage or amputation. Be vigilant about regularly inspecting your feet and seeing your provider at the first signs of trouble.
**12. How common is diabetic eye disease?**	Diabetic eye disease refers to a group of conditions affecting the eyes of people with diabetes. The most common is diabetic retinopathy, which damages the blood vessels in the retina. About one third of people with diabetes over age 40 already show some signs of retinopathy. It is the leading cause of blindness among working-age adults in the United States. According to external research, approximately 7.7 million Americans aged 40 and older have diabetic retinopathy.<br><br>Diabetic retinopathy often starts with no symptoms. Tiny bulges in blood vessels may leak fluid and blood into the retina, causing spots or blurred vision. Over time, abnormal vessel growth and scarring can occur, leading to serious vision loss. The outlook depends largely on early detection and treatment - finding and treating it early reduces risk of blindness by 95%. That’s why regular, comprehensive eye exams are so important for those with diabetes.<br><br>Other common diabetic eye diseases include cataracts and glaucoma. Cataracts cause clouding in the lenses of the eyes, while glaucoma damages the optic nerve. People with diabetes are about twice as likely to develop these conditions compared to the general public, with some research suggesting cataracts develop in about 20%

	of people with diabetes. Like diabetic retinopathy, they are treatable if detected early through regular eye screening. Controlling blood sugar, blood pressure and cholesterol can also help minimize risk of vision loss. Regular eye care and early treatment are key to preserving vision for people with diabetes.
**13. Who is more likely to develop diabetic eye disease?**	The risk of developing diabetic eye disease, also known as diabetic retinopathy, increases the longer someone has had diabetes. After 10 or more years living with diabetes, the risk becomes much greater. High blood glucose levels that remain uncontrolled and high blood pressure that goes untreated also significantly increase the chances of developing diabetic retinal damage.<br><br>Additionally, certain ethnic groups have been found to be at greater risk including African Americans, American Indians, Alaska Natives, Hispanics/Latinos, and Pacific Islanders. Older adults with diabetes also tend to be more susceptible to vision loss from diabetic retinopathy.<br><br>Pregnant women need to be especially vigilant about eye health. Diabetic retinopathy can rapidly worsen during pregnancy due to fluctuations in blood sugar and changes in the body. Regular eye exams during pregnancy are important to monitor for damage and initiate early treatment if needed.<br><br>In contrast, gestational diabetes (diabetes only during pregnancy) does not appear to pose the same level of risk for eye disease as other types of diabetes. Researchers are still investigating the reasons behind this.<br><br>In summary, those who have the greatest likelihood of developing diabetic eye disease are people who have had uncontrolled diabetes for over 10 years, those with untreated high blood pressure, certain ethnicities, pregnant women with diabetes, and older adults. The key is managing diabetes and related health factors to maintain blood vessels in the eyes and reduce the risks of vision loss.
**14. When should I see my health care provider about eye problems?**	As a diabetes specialist, I recommend getting a comprehensive dilated eye exam at least once a year to check for signs of diabetic retinopathy. This condition damages blood vessels in the retina and is a leading cause of blindness. Many people have no symptoms at first, which is why regular screening is so important for early detection and treatment.<br><br>During the exam, your pupils will be dilated and your retina closely examined for issues like leaking blood vessels, swelling, and damaged vessels. If any changes are found, your eye doctor will determine the severity and whether treatment like injections, laser surgery, or vitrectomy surgery is warranted. With early detection and care, up to 98% of vision loss can be prevented.

	In addition to yearly exams, contact your eye doctor right away if you notice any new vision problems. These include blurriness, floaters, missing areas of vision, eye pain, or color vision changes. You should also see your doctor immediately if you experience sudden vision changes like flashes of light, increased floaters, or a curtain-like obstruction of your field of vision. These can indicate a detached retina, which is an ophthalmological emergency.<br><br>Diabetic retinopathy is not the only eye condition linked to diabetes. You're also at increased risk for cataracts and glaucoma. Prompt medical attention for any new eye symptoms or changes in vision is critical for preserving your eyesight. Early intervention can prevent permanent damage and vision loss. So be sure to discuss any eye concerns with your healthcare provider right away to protect your vision.
**15. What can I do to protect my eyes?**	As diabetes patient, the health of your eyes is incredibly important. Diabetes can damage the small blood vessels in the retina, leading to diabetic retinopathy, a complication that can cause vision loss or blindness if left untreated. The good news is there are several key steps you can take to protect your eyes.<br><br>First, work closely with your doctor to manage your A1c, blood pressure, and cholesterol levels. Keeping your A1c under 7%, blood pressure below 140/90 mmHg, and LDL cholesterol under 100 mg/dL can significantly reduce your risk of diabetic eye disease. Second, quit smoking if you currently smoke. Smoking exacerbates diabetes complications.<br><br>Additionally, get a comprehensive dilated eye exam from an eye doctor at least once a year. Diabetic retinopathy often has no symptoms in its early stages, so an eye exam is crucial for detecting any issues early when they are most treatable. During the exam, your pupils will be dilated with eye drops to allow for a thorough inspection of the retina.<br><br>It's also important to monitor your vision at home. Report any new changes in vision, like blurriness or spotty vision, to your eye doctor right away. Diabetic retinopathy can progress quickly but is very treatable if caught early. With close monitoring and optimal diabetes management, the risk of severe vision loss from diabetic eye disease can be reduced by 95%.<br><br>The most important takeaway is that diligent diabetes care and regular eye exams are your best defense against diabetic eye disease. It may seem daunting, but taking it step-by-step to control your blood sugar, blood pressure, cholesterol, and smoking can go a long way in preserving your healthy vision for years to come. Your eyesight is precious - take proactive steps today to protect it.

**16. What if I already have some vision loss from diabetes?**	Diabetic retinopathy is a common complication of diabetes that can lead to vision loss if left untreated. If you already have some vision loss from diabetic retinopathy, the first step is to get a comprehensive eye exam to determine the extent of the damage. The ophthalmologist will assess signs of progression and recommend appropriate treatment options.<br><br>Even with existing vision impairment, further vision loss may be preventable through timely treatment like laser surgery, medications, or vitrectomy surgery. Maintaining optimal blood sugar, blood pressure, and cholesterol levels is also crucial. Regular dilated eye exams every 6-12 months are important for monitoring your condition.<br><br>According to the National Institutes of Health, if you have irreversible vision loss from diabetes, ask your eye doctor about referral to a low vision and rehabilitation clinic. These clinics have eye care professionals that can help you manage permanent vision impairment through specialized devices, training, and support services. The goal is to help you retain functional vision so you can stay independent with daily living, hobbies, social activities, and getting around safely.<br><br>Vision loss may seem disheartening, but know that remaining sight can be preserved through diligent medical care and lifestyle management of your diabetes. Don't delay seeking help if you notice new vision changes. With proper treatment and low vision aids, it is possible to adapt to vision loss and enjoy a quality life. Maintain regular communication with both your eye doctor and your diabetes healthcare team for optimal management of this complication.

Supplementary Table 1e: Bard Response to Diabetes-Related Questions

Question	Bard Response
**Topic: Concepts of Diabetes**	
**1. What Is Diabetes?**	Diabetes is a chronic health condition that affects how your body turns food into energy. Most of the food you eat is broken down into sugar (glucose) and released into your bloodstream. When your blood sugar goes up, it signals your pancreas to release insulin. Insulin acts like a key to let the blood sugar into your cells for use as energy.<br><br>If you have diabetes, your body either doesn't make enough insulin or doesn't use insulin the way it should. This causes too much blood sugar to stay in your bloodstream. Over time, high blood sugar can cause serious health problems, including heart disease, stroke, blindness, kidney disease, and nerve damage.<br><br>There are two main types of diabetes:<br><br>* Type 1 diabetes: This is an autoimmune disease. This means that your body's immune system attacks and destroys the cells in your pancreas that make insulin. Type 1 diabetes usually starts in childhood or adolescence, but it can occur at any age.<br>* Type 2 diabetes: This is the most common type of diabetes. It used to be called adult-onset diabetes, but it is now being diagnosed in children and adolescents more often. Type 2 diabetes occurs when your body becomes resistant to insulin or doesn't make enough insulin.<br><br>There is no cure for diabetes, but it can be managed with diet, exercise, and medication. If you have diabetes, it is important to work with your doctor to develop a treatment plan that is right for you.<br><br>Here are some of the common symptoms of diabetes:<br><br>* Increased thirst<br>* Frequent urination<br>* Extreme hunger

	* Unexplained weight loss<br>* Fatigue<br>* Blurred vision<br>* Slow-healing sores<br><br>If you experience any of these symptoms, it is important to see your doctor right away. Early diagnosis and treatment of diabetes can help prevent serious complications.<br><br>I hope this answers your question. Please let me know if you have any other questions.
**2. How can I determine which type of diabetes I have?**	The two main types of diabetes are type 1 and type 2. Type 1 diabetes is an autoimmune disease, meaning that the body's immune system attacks and destroys the cells in the pancreas that produce insulin. Insulin is a hormone that helps the body use glucose for energy. Without insulin, glucose builds up in the blood, leading to high blood sugar levels.<br><br>Type 2 diabetes is caused by a combination of genetics and lifestyle factors, such as being overweight or obese, having a family history of diabetes, or being physically inactive. In type 2 diabetes, the pancreas still produces insulin, but the body's cells become resistant to its effects. This leads to high blood sugar levels.<br><br>The only way to definitively determine which type of diabetes you have is through blood tests. These tests can measure your blood sugar levels, as well as the levels of certain antibodies and hormones that are associated with each type of diabetes.<br><br>The following blood tests can be used to diagnose diabetes:<br><br>* Fasting blood sugar test: This test measures your blood sugar level after an overnight fast. A fasting blood sugar level of 126 mg/dL or higher on two separate tests is diagnostic of diabetes.<br>* A1C test: This test measures your average blood sugar level over the past 2 to 3 months. An A1C level of 6.5% or higher is diagnostic of diabetes.

	* Oral glucose tolerance test (OGTT): This test is usually only done if the results of the fasting blood sugar test and A1C test are inconclusive. The OGTT involves drinking a sugary liquid and then having your blood sugar levels measured at regular intervals over the next 2 hours. A blood sugar level of 200 mg/dL or higher 2 hours after drinking the sugary liquid is diagnostic of diabetes.<br><br>If you have any concerns about your blood sugar levels, it is important to see a doctor for testing. Early diagnosis and treatment of diabetes can help prevent complications, such as heart disease, stroke, blindness, and kidney failure.<br><br>Here are some additional tips for determining which type of diabetes you have:<br><br>* Talk to your doctor about your family history of diabetes. Type 1 diabetes is more common in people with a family history of the disease.<br>* Consider your weight. Type 2 diabetes is more common in people who are overweight or obese.<br>* Ask your doctor about your risk factors for diabetes. These include being physically inactive, having high blood pressure, and having high cholesterol levels.<br><br>If you have any of the risk factors for diabetes, it is important to get regular blood sugar checks. This can help you detect diabetes early, when it is easier to manage.
**3. Who is more likely to develop type 1 diabetes?**	Who is more likely to develop type 1 diabetes?**<br><br>Type 1 diabetes is an autoimmune disease that causes the body to attack the cells in the pancreas that produce insulin. Insulin is a hormone that helps the body use glucose for energy. Without insulin, glucose builds up in the blood, which can lead to serious health problems.<br><br>The exact cause of type 1 diabetes is not known, but it is thought to be a combination of genetic and environmental factors. The following are some of the risk factors for type 1 diabetes:<br><br>* **Family history:** People who have a parent, sibling, or child with type 1 diabetes are more likely to develop the condition themselves.<br>* **Race/ethnicity:** Type 1 diabetes is more common in white people than in black or Hispanic people.<br>* **Age:** Type 1 diabetes usually develops in children and young adults, but it can occur at any age.<br>* **Geography:** Type 1 diabetes is more common in people who live in northern latitudes.

	* **Viral infections:** Some viruses, such as the Coxsackie virus, have been linked to an increased risk of type 1 diabetes.<br><br>It is important to note that not everyone with these risk factors will develop type 1 diabetes. And, some people who develop type 1 diabetes may not have any of these risk factors.<br><br>If you are concerned about your risk of developing type 1 diabetes, talk to your doctor. There is no way to prevent type 1 diabetes, but early diagnosis and treatment can help to manage the condition and prevent complications.<br><br>I hope this helps!
**4. Who is more likely to develop type 2 diabetes?**	As a public health expert specializing in diabetes, I can tell you that there are many factors that can increase a person's risk of developing type 2 diabetes. Some of the most common risk factors include:<br><br>* **Age:** The risk of developing type 2 diabetes increases with age. About 1 in 10 people aged 45 to 64 have type 2 diabetes, and about 1 in 5 people aged 65 and older have it.<br>* **Family history:** If you have a family history of type 2 diabetes, you are more likely to develop it yourself.<br>* **Weight:** Being overweight or obese is a major risk factor for type 2 diabetes.<br>* **Race and ethnicity:** People of African American, Hispanic/Latino, American Indian, Asian American, and Pacific Islander descent are more likely to develop type 2 diabetes than white people.<br>* **Physical activity:** People who are physically inactive are more likely to develop type 2 diabetes.<br>* **Certain health conditions:** Having certain health conditions, such as high blood pressure, high cholesterol, or polycystic ovary syndrome (PCOS), can increase your risk of developing type 2 diabetes.<br>* **Pregnancy:** Women who have gestational diabetes (diabetes during pregnancy) are more likely to develop type 2 diabetes later in life.<br><br>It is important to note that not everyone with these risk factors will develop type 2 diabetes. However, if you have any of these risk factors, it is important to talk to your doctor about ways to reduce your risk.

	There are a number of things you can do to reduce your risk of developing type 2 diabetes, including:<br><br>* Maintaining a healthy weight<br>* Eating a healthy diet<br>* Being physically active<br>* Quitting smoking<br>* Controlling your blood pressure and cholesterol levels<br><br>If you are concerned about your risk of developing type 2 diabetes, talk to your doctor. They can help you assess your risk and develop a plan to reduce it.
**Topic: Symptoms & Causes**	
**1. What are the symptoms of diabetes?**	Hi there! I am a public health expert specializing in diabetes. Here are the common symptoms of diabetes:<br><br>* **Frequent urination:** You may need to urinate more often, especially at night. This is because when your blood sugar is high, your kidneys try to get rid of the extra sugar by filtering it out of your blood and into your urine.<br>* **Excessive thirst:** You may feel very thirsty, even if you're drinking plenty of fluids. This is because your body is trying to get rid of the extra sugar in your blood.<br>* **Unexplained weight loss:** You may lose weight even though you're eating the same amount or more. This is because when your body doesn't have enough insulin, it can't use glucose for energy. Instead, it breaks down fat and muscle for energy, which can lead to weight loss.<br>* **Increased hunger:** You may feel very hungry, even after you've just eaten. This is because your body is trying to get the glucose it needs for energy.<br>* **Blurry vision:** High blood sugar can damage the blood vessels in your eyes, which can lead to blurry vision.<br>* **Slow-healing sores:** Cuts and bruises may take longer to heal when you have diabetes. This is because high blood sugar can damage the nerves and blood vessels that help wounds heal.<br>* **Fatigue:** You may feel tired all the time, even after getting a good night's sleep. This is because high blood sugar can make it difficult for your body to produce energy.<br>* **Irritability:** You may feel irritable or have mood swings. This is because high blood sugar can affect your brain function.<br>* **Dry skin:** Your skin may become dry and itchy. This is because high blood sugar can damage the nerves that control your sweat glands.

	* **Recurring infections:** You may get infections more often, especially urinary tract infections and yeast infections. This is because high blood sugar can weaken your immune system.<br><br>If you are experiencing any of these symptoms, it is important to see a doctor to get tested for diabetes. Early diagnosis and treatment can help prevent serious complications of diabetes.<br><br>It is also important to note that not everyone with diabetes experiences all of these symptoms. Some people may have only a few symptoms, while others may have none at all. If you are concerned about your risk of diabetes, talk to your doctor.
**2. What causes type 1 diabetes?**	Sure, I can help you with that.<br><br>Type 1 diabetes is an autoimmune disease that causes the body to attack the cells in the pancreas that produce insulin. Insulin is a hormone that helps the body use glucose for energy. Without insulin, glucose builds up in the blood, which can lead to serious health problems.<br><br>The exact cause of type 1 diabetes is unknown, but it is thought to be caused by a combination of genetic and environmental factors. Genes that make people more likely to develop type 1 diabetes have been identified, but not everyone with these genes will develop the disease. Environmental factors that may trigger type 1 diabetes include viruses, certain chemicals, and exposure to certain foods.<br><br>Type 1 diabetes is not preventable, but it can be managed with insulin therapy, diet, and exercise. People with type 1 diabetes need to take insulin injections every day to keep their blood sugar levels in a healthy range. They also need to eat a healthy diet and exercise regularly.<br><br>The symptoms of type 1 diabetes can develop suddenly, or they may develop gradually over time. Some of the common symptoms include:<br><br>* Increased thirst<br>* Frequent urination<br>* Extreme hunger<br>* Weight loss

	* Fatigue<br>* Blurred vision<br>* Slow-healing sores<br>* Irritability<br><br>If you experience any of these symptoms, it is important to see a doctor right away. Early diagnosis and treatment of type 1 diabetes is important to prevent serious complications.<br><br>I hope this answers your question. Let me know if you have any other questions.
**3. What causes type 2 diabetes?**	Type 2 diabetes is a chronic condition that affects how your body turns food into energy. It occurs when your body doesn't produce enough insulin or doesn't use insulin effectively. Insulin is a hormone that helps your body use glucose for energy. Glucose is a type of sugar found in your blood.<br><br>The exact cause of type 2 diabetes is unknown, but it is thought to be caused by a combination of genetic and environmental factors. The main environmental factors that contribute to type 2 diabetes are:<br><br>* **Overweight and obesity:** Being overweight or obese is the single biggest risk factor for type 2 diabetes. When you have excess weight, especially around your waist, your cells become resistant to insulin.<br>* **Physical inactivity:** Physical activity helps to keep your weight in check and also makes your cells more sensitive to insulin. People who are physically inactive are more likely to develop type 2 diabetes.<br>* **Age:** The risk of type 2 diabetes increases as you get older.<br>* **Race and ethnicity:** People of African American, Hispanic, American Indian, and Asian American descent are more likely to develop type 2 diabetes than white people.<br><br>If you have any of the risk factors for type 2 diabetes, it is important to talk to your doctor. Early diagnosis and treatment can help to prevent or delay the onset of the disease.

	Here are some additional tips to help prevent type 2 diabetes:<br><br>* Maintain a healthy weight.<br>* Eat a healthy diet that is low in sugar and processed foods.<br>* Get regular physical activity.<br>* If you have a family history of type 2 diabetes, talk to your doctor about getting tested.<br><br>I hope this answers your question.
**4. What causes gestational diabetes?**	* **Hormonal changes:** During pregnancy, the placenta produces hormones that can make the body less sensitive to insulin.<br>* **Overweight or obesity:** Women who are overweight or obese are more likely to develop gestational diabetes.<br>* **Family history:** Women who have a family history of diabetes are also more likely to develop gestational diabetes.<br>* **Age:** Women over the age of 35 are more likely to develop gestational diabetes.<br>* **Race or ethnicity:** Women of certain races or ethnicities, such as Black, Hispanic, American Indian, and Asian American, are more likely to develop gestational diabetes.<br><br>I hope this is helpful. Let me know if you have any other questions.
**Topic: Diabetes Tests & Diagnosis**	
**1. Who should be tested for diabetes?**	**Who should be tested for diabetes?**<br><br>The American Diabetes Association recommends that everyone get tested for diabetes at least once as part of a routine health checkup. However, there are certain people who are at higher risk for diabetes and should be tested more often. These include:<br><br>* People who have symptoms of diabetes, such as frequent urination, increased thirst, unexplained weight loss, blurry vision, or fatigue.<br>* Adults aged 45 and older, even if they have no symptoms.

	* Adults younger than 45 who are overweight or obese and have one or more risk factors for type 2 diabetes, such as a family history of diabetes, a history of gestational diabetes, or high blood pressure.<br>* Women who are planning to become pregnant.<br>* People of certain ethnic groups, such as African Americans, Hispanic Americans, Native Americans, Asian Americans, and Pacific Islanders.<br>* People with certain medical conditions, such as polycystic ovary syndrome (PCOS) or heart disease.<br>* People who are taking certain medications, such as steroids.<br><br>If you have any of the risk factors for diabetes, talk to your doctor about getting tested more often. The earlier diabetes is diagnosed, the sooner treatment can begin to prevent serious complications.<br><br>Here are the blood tests that are used to diagnose diabetes:<br><br>* Fasting plasma glucose (FPG): This test measures the amount of glucose in your blood after you have fasted for at least 8 hours. A blood sugar level of 126 mg/dL or higher on two separate tests indicates diabetes.<br>* Oral glucose tolerance test (OGTT): This test measures how your body responds to a sugary drink. You will drink a sugary drink and then have your blood sugar levels checked at regular intervals over the next 2 hours. A blood sugar level of 200 mg/dL or higher 2 hours after drinking the sugary drink indicates diabetes.<br><br>I hope this information is helpful. Please let me know if you have any other questions.
**2. What tests are used to diagnose diabetes?**	There are four main tests used to diagnose diabetes:<br><br>* **Fasting plasma glucose (FPG) test:** This test measures your blood sugar after you have fasted (not eaten or drunk anything except water) for at least 8 hours. A normal FPG level is less than 100 mg/dL. A level of 126 mg/dL or higher on two separate tests indicates diabetes.<br>* **Oral glucose tolerance test (OGTT):** This test measures your blood sugar before and after you drink a sugary drink. A normal OGTT result is:<br>* Fasting blood sugar level of less than 100 mg/dL<br>* One-hour blood sugar level of less than 140 mg/dL<br>* Two-hour blood sugar level of less than 126 mg/dL

	A level of 200 mg/dL or higher at any point during the OGTT test indicates diabetes.<br>* **Random blood sugar test:** This test measures your blood sugar level at any time of day, regardless of whether you have eaten or not. A normal random blood sugar level is less than 140 mg/dL. A level of 200 mg/dL or higher indicates diabetes.<br>* **Hemoglobin A1c (HbA1c) test:** This test measures your average blood sugar level over the past 2 to 3 months. A normal HbA1c level is less than 5.7%. A level of 6.5% or higher indicates diabetes.<br><br>The most common test used to diagnose diabetes is the FPG test. However, the OGTT test may be used if the FPG test is inconclusive. The HbA1c test is also a good option for diagnosing diabetes, but it is not as accurate as the FPG test.<br><br>If you have any of the symptoms of diabetes, such as frequent urination, increased thirst, unexplained weight loss, or blurry vision, you should see your doctor for testing. Early diagnosis and treatment of diabetes can help prevent serious complications, such as heart disease, stroke, blindness, and kidney disease.<br><br>I hope this is helpful!
**3. What test results tell me if I have diabetes or prediabetes?**	The A1C test is the most common blood test used to diagnose diabetes or prediabetes. It measures your average blood sugar level over the past 2 to 3 months. A normal A1C level is below 5.7%. A level of 5.7% to 6.4% indicates prediabetes, and 6.5% or higher indicates diabetes.<br><br>The FPG test is less commonly used than the A1C test. It measures your blood sugar level after an overnight fast (not eating). A normal fasting blood sugar level is below 99 mg/dL. A level of 100 to 125 mg/dL indicates prediabetes, and 126 mg/dL or higher indicates diabetes.<br><br>The OGTT is the least commonly used blood test for diabetes. It measures your blood sugar level after you drink a sugary liquid. A normal OGTT result is below 140 mg/dL at 2 hours. A level of 140 to 199 mg/dL indicates prediabetes, and 200 mg/dL or higher indicates diabetes.<br><br>If you have any of the following risk factors for diabetes, you should talk to your doctor about getting tested:<br><br>* Age: You are more likely to develop diabetes if you are over 45 years old.

	* Family history: If you have a family history of diabetes, you are more likely to develop it yourself.<br>* Race or ethnicity: African Americans, Hispanic Americans, Native Americans, and Asian Americans are more likely to develop diabetes than white Americans.<br>* Weight: If you are overweight or obese, you are more likely to develop diabetes.<br>* Physical activity: If you are not physically active, you are more likely to develop diabetes.<br>* Certain medical conditions: If you have certain medical conditions, such as polycystic ovary syndrome (PCOS) or high blood pressure, you are more likely to develop diabetes.<br><br>If you are diagnosed with prediabetes, it is important to make lifestyle changes to lower your risk of developing type 2 diabetes. These changes include losing weight, eating a healthy diet, and being physically active.<br><br>I hope this information is helpful. Please let me know if you have any other questions.
**4. Which tests help my doctor know what kind of diabetes I have?**	The tests that can help your doctor know what kind of diabetes you have include the A1C test, fasting blood sugar test, oral glucose tolerance test (OGTT), C-peptide test, and antibody tests.<br><br>The A1C test is the most common test used to diagnose diabetes. It measures your average blood sugar level over the past 2 to 3 months. A level of 6.5% or higher indicates diabetes.<br><br>The fasting blood sugar test measures your blood sugar level after you have not eaten for 8 hours. A level of 126 mg/dL or higher indicates diabetes.<br><br>The OGTT is used to diagnose gestational diabetes. You will drink a sugary solution and then have your blood sugar level checked at specific intervals over the next 2 hours. A level of 200 mg/dL or higher at 2 hours indicates gestational diabetes.<br><br>The C-peptide test measures the amount of C-peptide in your blood. C-peptide is a protein that is released when the pancreas makes insulin. A low level of C-peptide suggests that you have type 1 diabetes.

	Antibody tests look for antibodies that are produced by the body's immune system in response to type 1 diabetes. These tests are often done in combination with the C-peptide test.<br><br>Your doctor will use the results of these tests, along with your symptoms and medical history, to determine what kind of diabetes you have.<br><br>I hope this helps!
**Topic: Managing Diabetes**	
**1. How can I manage my diabetes?**	* **Eat a healthy diet.** This means eating plenty of fruits, vegetables, and whole grains. It also means limiting your intake of processed foods, sugary drinks, and unhealthy fats.<br>* **Exercise regularly.** Aim for at least 30 minutes of moderate-intensity exercise most days of the week.<br>* **Take your medications as prescribed.** If you are taking insulin or other diabetes medications, it is important to take them as prescribed by your doctor.<br>* **Monitor your blood sugar levels.** This will help you track how well your diabetes is being managed and make adjustments to your treatment plan as needed.<br>* **Get regular checkups.** Your doctor can monitor your blood sugar levels, blood pressure, and cholesterol levels, and make sure you are getting the care you need.<br>* **Take care of your feet.** People with diabetes are at increased risk of developing foot problems. This is because high blood sugar can damage the nerves and blood vessels in your feet. Make sure to inspect your feet daily for any cuts, blisters, or sores, and see your doctor right away if you have any problems.<br><br>These are just some of the things you can do to manage diabetes. It is important to work with your doctor to develop a personalized treatment plan that is right for you.<br><br>Here are some additional tips that may be helpful:<br><br>* **Set realistic goals.** Don't try to change everything all at once. Start by making small changes to your diet and exercise habits, and gradually increase your activity level.<br>* **Find a support system.** Talking to other people with diabetes can be helpful. There are many support groups available online and in your community.<br>* **Don't give up.** Managing diabetes takes time and effort, but it is worth it. With hard work and dedication, you can control your diabetes and live a healthy life.
**2. What are the recommended targets for blood glucose levels?**	* **Type 1 diabetes:**<br>* Before meals: 80 to 130 mg/dL (4.4 to 7.2 mmol/L)

	* 2 hours after meals: Less than 180 mg/dL (10.0 mmol/L)<br>* **Type 2 diabetes:**<br>* Before meals: 90 to 130 mg/dL (5.0 to 7.2 mmol/L)<br>* 2 hours after meals: Less than 180 mg/dL (10.0 mmol/L)<br>* **Gestational diabetes:**<br>* Before meals: 90 to 120 mg/dL (5.0 to 6.7 mmol/L)<br>* 2 hours after meals: Less than 140 mg/dL (7.8 mmol/L)<br><br>It is important to note that these are just general goals and the actual targets may vary depending on the individual's circumstances. It is always best to work with your doctor to determine the best targets for you.<br><br>In addition to blood glucose levels, it is also important to monitor A1C levels. A1C is a measure of your average blood glucose levels over the past 3 months. The goal for A1C levels is less than 7% for most adults with diabetes.<br><br>Managing blood glucose levels is an important part of living with diabetes. By working with your doctor to set and achieve your blood glucose and A1C goals, you can reduce your risk of complications from diabetes.<br><br>Here are some tips for managing blood glucose levels:<br><br>* Eat a healthy diet that is low in sugar and processed foods.<br>* Exercise regularly.<br>* Take your medications as prescribed by your doctor.<br>* Monitor your blood glucose levels regularly.<br>* Learn how to treat high and low blood glucose levels.<br><br>By following these tips, you can help keep your blood glucose levels in the target range and improve your overall health.

**3. What medicines might I take for diabetes?**	Sure, here is the answer within 300 words:<br><br>There are many different types of diabetes medicines available, and the best one for you will depend on your individual needs and circumstances. Some of the most common types of diabetes medicines for type 2 diabetes include metformin, sulfonylureas, DPP-4 inhibitors, GLP-1 receptor agonists, and SGLT2 inhibitors.<br><br>Metformin is the most commonly prescribed oral medication for type 2 diabetes. It helps to lower blood sugar levels by increasing the body's sensitivity to insulin. Sulfonylureas work by stimulating the pancreas to release more insulin. DPP-4 inhibitors work by blocking the action of an enzyme that breaks down a hormone called incretin. Incretins help to lower blood sugar levels by increasing insulin production and decreasing glucose production in the liver. GLP-1 receptor agonists work by mimicking the effects of incretin. They help to lower blood sugar levels by increasing insulin production, decreasing glucose production in the liver, and slowing down the emptying of the stomach. SGLT2 inhibitors work by blocking the action of a protein called sodium-glucose co-transporter 2 (SGLT2). SGLT2 is responsible for the reabsorption of glucose from the kidneys back into the bloodstream. By blocking SGLT2, these medications help to increase the amount of glucose that is excreted in the urine.<br><br>The decision of which diabetes medicine is right for you is a personal one that should be made in consultation with your doctor. Your doctor will consider your individual circumstances, such as your age, medical history, and other medications you are taking, when making a recommendation.<br><br>It is important to note that diabetes medicines are not a cure for diabetes. They are simply tools that can help you to manage your blood sugar levels and prevent complications. It is also important to make lifestyle changes, such as eating a healthy diet, exercising regularly, and maintaining a healthy weight, to help manage your diabetes.<br><br>I hope this information is helpful. Please let me know if you have any other questions.
**4. What are the different types of insulin?**	* **Rapid-acting insulin:** Onset within 15 minutes, duration 3 to 5 hours.<br>* **Short-acting insulin:** Onset within 30 minutes, duration 3 to 6 hours.<br>* **Intermediate-acting insulin:** Onset within 2 to 4 hours, duration 12 to 18 hours.<br>* **Long-acting insulin:** Onset within 2 hours, duration up to 24 hours.

	In addition to these four main types of insulin, there are also pre-mixed insulins, which are a combination of rapid-acting and intermediate-acting insulin. Pre-mixed insulins are available in different ratios, such as 70/30 (70% rapid-acting insulin and 30% intermediate-acting insulin) or 50/50 (50% rapid-acting insulin and 50% intermediate-acting insulin).<br><br>The type of insulin that is right for you will depend on your individual needs and lifestyle. Your doctor will help you choose the right type and dosage of insulin to manage your diabetes.<br><br>I hope this is helpful. Please let me know if you have any other questions.
**5. What are the different ways to take insulin?**	There are four main ways to take insulin: injections, insulin pens, insulin pumps, and insulin inhalers.<br><br>* **Injections:** This is the most common way to take insulin. Insulin is injected into the fat just below the skin using a syringe or an insulin pen.<br>* **Insulin pens:** Insulin pens are a type of pre-filled syringe that makes it easier to inject insulin. They are also more portable than syringes.<br>* **Insulin pumps:** Insulin pumps are devices that deliver insulin continuously through a small tube that is inserted under the skin. Pumps can be a good option for people who need more precise control of their blood sugar levels.<br>* **Insulin inhaler:** Insulin inhaler is a device that delivers insulin through the lungs. It is the only non-injectable form of insulin that is currently available. However, it is only approved for use in adults with type 1 diabetes.<br><br>The best way to take insulin will vary depending on the individual's needs and preferences. Talk to your doctor or diabetes educator to decide which method is right for you.<br><br>Here are some of the factors to consider when choosing a method of insulin delivery:<br><br>* **Your lifestyle:** If you are active and on the go, you may prefer a more portable option, such as an insulin pen or pump.<br>* **Your dexterity:** If you have difficulty giving injections, you may prefer an insulin pen or pump.<br>* **Your budget:** Injections are the most affordable option, while insulin pumps are the most expensive.<br>* **Your health insurance:** Some insurance plans may cover the cost of insulin pumps, but others may not.

	Ultimately, the best way to take insulin is the way that works best for you and your lifestyle. Talk to your doctor or diabetes educator to discuss your options and find the method that is right for you.
**6. What oral medicines treat type 2 diabetes?**	There are several different classes of oral diabetes medications that work in different ways to help manage blood sugar levels. The most common classes are:<br><br>* **Biguanides:** Metformin is the most common biguanide. It works by decreasing glucose production in the liver, increasing insulin sensitivity, and lowering intestinal sugar absorption.<br>* **Sulfonylureas:** Sulfonylureas work by stimulating the release of insulin from the pancreas.<br>* **DPP-4 inhibitors:** DPP-4 inhibitors work by blocking the breakdown of a hormone called glucagon-like peptide-1 (GLP-1). GLP-1 helps to control blood sugar levels by increasing insulin production and decreasing glucagon production.<br>* **SGLT2 inhibitors:** SGLT2 inhibitors work by blocking the absorption of glucose in the kidneys. This causes more glucose to be excreted in the urine.<br><br>The best oral medication for type 2 diabetes will vary from person to person. Your doctor will consider your individual needs and circumstances when making a recommendation.<br><br>It is important to note that oral medications are not a cure for type 2 diabetes. They are only part of a comprehensive treatment plan that may also include lifestyle changes such as diet, exercise, and weight loss.<br><br>I hope this helps!
**7. What should I know about side effects of diabetes medicines?**	Sure, here is the answer in 300 words or less:<br><br>Diabetes medications can have side effects, but the benefits of taking them often outweigh the risks. Some common side effects of diabetes medications include:<br><br>* Gastrointestinal problems (such as nausea, vomiting, diarrhea, and constipation)<br>* Weight gain<br>* Low blood sugar (hypoglycemia)

	* High blood sugar (hyperglycemia)<br>* Headache<br>* Rash<br>* Fatigue<br>* Metallic taste in the mouth<br><br>If you experience any side effects from your diabetes medication, it is important to talk to your doctor. They may be able to adjust your dose or switch you to a different medication.<br><br>Here are some tips for managing the side effects of diabetes medications:<br><br>* Take your medication as prescribed by your doctor.<br>* Be aware of the signs and symptoms of low blood sugar and high blood sugar.<br>* Talk to your doctor about ways to manage the side effects of your diabetes medication.<br><br>If you are concerned about the side effects of diabetes medications, talk to your doctor. They can help you to make the best decision for your individual needs.<br><br>Here are some additional details about the side effects of diabetes medications:<br><br>* **Gastrointestinal problems:** These are the most common side effects of diabetes medications. They usually go away on their own after a few weeks.<br>* **Weight gain:** Some diabetes medications can cause weight gain. This is because they can make you feel more hungry or make it harder for your body to burn calories.<br>* **Low blood sugar:** This is a serious side effect that can happen if you take too much diabetes medication or if you do not eat enough food. Symptoms of low blood sugar include shakiness, sweating, dizziness, and confusion.<br>* **High blood sugar:** This is also a serious side effect that can happen if you do not take enough diabetes medication or if you eat too much food. Symptoms of high blood sugar include increased thirst, frequent urination, and blurry vision.

	It is important to talk to your doctor about the side effects of diabetes medications so that they can help you manage them.
**8. What foods can I eat if I have diabetes?**	**What foods can I eat if I have diabetes?**<br><br>There is no one-size-fits-all answer to this question, as the best foods for people with diabetes will vary depending on their individual needs and preferences. However, some general guidelines include:<br><br>* **Choose whole grains over refined grains.** Whole grains are a good source of fiber, which can help to regulate blood sugar levels.<br>* **Eat plenty of fruits and vegetables.** Fruits and vegetables are low in calories and carbohydrates, and they are packed with vitamins, minerals, and antioxidants.<br>* **Include lean protein in your diet.** Lean protein can help to keep you feeling full and satisfied.<br>* **Choose healthy fats.** Healthy fats, such as those found in avocados, nuts, and seeds, can help to improve heart health and protect against diabetes complications.<br>* **Limit sugary drinks.** Sugary drinks can cause blood sugar levels to spike.<br>* **Read food labels carefully.** Be sure to check the carbohydrate content of foods before you eat them.<br><br>It is important to work with a registered dietitian or other healthcare professional to create a personalized meal plan that meets your individual needs.<br><br>Here are some additional tips for people with diabetes when making food choices:<br><br>* Cook more meals at home and avoid processed foods.<br>* Portion control is important. Be mindful of how much food you are eating.<br>* Spread your carbohydrates throughout the day rather than eating them all at once.<br>* Snack on healthy foods throughout the day to help keep your blood sugar levels stable.<br>* Stay hydrated by drinking plenty of water.<br><br>By following these tips, people with diabetes can make healthy food choices that can help them manage their condition and live a long and healthy life.

**9. What foods and drinks should I limit if I have diabetes?**	If you have diabetes, it is important to limit your intake of foods and drinks that are high in saturated fat, sodium, sugar, and added sugar. These foods can raise your blood sugar levels and increase your risk of heart disease, stroke, and other health problems.<br><br>Some of the foods and drinks you should limit include:<br><br>* Fried foods<br>* Full-fat dairy products<br>* Processed foods<br>* Red meat<br>* Sugary drinks<br>* Sweets<br>* Salty snacks<br><br>Instead, focus on eating plenty of healthy foods, such as:<br><br>* Fruits and vegetables<br>* Whole grains<br>* Lean protein<br>* Low-fat or fat-free dairy products<br>* Water<br><br>You should also talk to your doctor or a registered dietitian about creating a meal plan that is right for you.<br><br>Here are some additional tips for people with diabetes:<br><br>* Eat regular meals and snacks throughout the day.

	* Choose complex carbohydrates over simple carbohydrates.<br>* Limit your portion sizes.<br>* Get regular exercise.<br>* Monitor your blood sugar levels regularly.<br><br>By following these tips, you can help to manage your diabetes and live a healthy life.<br><br>I hope this helps!
**10. When should I eat if I have diabetes?**	The ideal meal schedule for people with diabetes will vary depending on individual factors such as their type of diabetes, medications, and lifestyle. However, as a general rule, people with diabetes should aim to eat regular meals and snacks throughout the day, spaced about 4 to 5 hours apart. This will help keep their blood sugar levels stable and prevent spikes and crashes.<br><br>For breakfast, people with diabetes should choose foods that are high in fiber and protein, such as oatmeal, eggs, or yogurt. These foods will help slow down the absorption of sugar into the bloodstream. Lunch and dinner should also include a variety of healthy foods, such as vegetables, fruits, and whole grains. People with diabetes should also avoid sugary drinks and processed foods.<br><br>If you are unsure about when and what to eat, it is always best to talk to your doctor or a registered dietitian. They can help you create a meal plan that is tailored to your individual needs.<br><br>Here are some additional tips for eating with diabetes:<br><br>* Read food labels carefully and choose foods that are low in carbohydrates.<br>* Learn how to count carbohydrates so you can make informed choices about what to eat.<br>* Test your blood sugar regularly to track how your blood sugar levels are responding to your meal plan.<br>* Be prepared for unexpected events, such as having to skip a meal or snack.

	By following these tips, you can help keep your blood sugar levels under control and reduce your risk of complications.
**11. How much can I eat if I have diabetes?**	The amount of food you can eat if you have diabetes depends on a number of factors, but a general guideline is to aim for 45-60 grams of carbohydrates per meal and 15-20 grams per snack. It is also important to choose foods that are low in sugar and fat and high in fiber.<br><br>Here are some healthy foods that are good for people with diabetes:<br><br>* Vegetables<br>* Fruits<br>* Whole grains<br>* Lean protein<br>* Healthy fats<br><br>It is also important to stay hydrated by drinking plenty of water throughout the day.<br><br>If you are unsure how much food you should eat, talk to your doctor or a registered dietitian. They can help you create a meal plan that is right for you.<br><br>Here are some additional tips for eating with diabetes:<br><br>* Eat regular meals and snacks throughout the day.<br>* Count your carbohydrates.<br>* Monitor your blood sugar levels regularly.<br>* Talk to your doctor or a registered dietitian about any medications you are taking.<br><br>By following these tips, you can eat a healthy diet and manage your diabetes.<br><br>I hope this helps!

**12. Will supplements and vitamins help my diabetes?**	The effectiveness of supplements and vitamins in managing diabetes is still being studied. Some vitamins and minerals, such as vitamin D, magnesium, and chromium, may help improve blood sugar control in people with diabetes. However, more research is needed to confirm these findings.<br><br>It is important to talk to your doctor before taking any supplements, as they may interact with your medications or have other side effects. The best way to manage diabetes is to follow a healthy diet, exercise regularly, and take medication as prescribed by your doctor.<br><br>Here are some other things you can do to help manage your diabetes:<br><br>* Keep your blood sugar levels in check. Check your blood sugar levels regularly and make adjustments to your treatment plan as needed.<br>* Get regular exercise. Aim for at least 30 minutes of moderate-intensity exercise most days of the week.<br>* Eat a healthy diet. This means eating plenty of fruits, vegetables, and whole grains. It also means limiting processed foods, sugary drinks, and unhealthy fats.<br>* Take your medication as prescribed. If you are taking medication for diabetes, it is important to take it as prescribed by your doctor.<br><br>Managing diabetes can be challenging, but it is important to remember that you are not alone. There are many resources available to help you, including your doctor, diabetes educators, and support groups.
**13. Why should I be physically active if I have diabetes?**	**Why should you be physically active if you have diabetes?**<br><br>Exercise is an important part of diabetes management. It can help improve blood sugar control, reduce the risk of heart disease and stroke, and help you maintain a healthy weight.<br><br>Some of the benefits of exercise for people with diabetes include:<br><br>* Improved blood sugar control<br>* Reduced risk of heart disease and stroke<br>* Weight loss or maintenance<br>* Improved blood vessel function

	It is important to talk to your doctor before starting any new exercise program, especially if you have any health conditions. They can help you develop an exercise plan that is safe and effective for you.<br><br>Here are some tips for people with diabetes who are starting an exercise program:<br><br>* Start slowly and gradually increase the intensity and duration of your workouts over time.<br>* Check your blood sugar levels before, during, and after exercise.<br>* Carry a snack or sugary drink with you in case your blood sugar levels drop too low.<br>* Drink plenty of fluids before, during, and after exercise.<br>* Wear comfortable clothing and shoes.<br>* Choose activities that you enjoy and that are appropriate for your fitness level.<br><br>Even a small amount of exercise can make a big difference. So if you have diabetes, get moving and reap the many benefits of exercise!
**14. How can I be physically active safely if I have diabetes?**	Sure, here is the answer within 300 words:<br>**How to be physically active safely with diabetes**<br><br>People with diabetes can be physically active safely by following these tips:<br><br>* Start slowly and gradually increase the intensity and duration of your activity.<br>* Check your blood sugar before, during, and after exercise.<br>* Carry a snack with you in case your blood sugar goes low.<br>* Drink plenty of fluids before, during, and after exercise.<br>* Wear comfortable clothing and shoes that fit well.<br>* Avoid exercising in extreme heat or cold.<br>* Talk to your doctor before starting any new exercise program.

	Some specific types of physical activity that are good for people with diabetes include:<br><br>* Walking<br>* Cycling<br>* Swimming<br>* Yoga<br>* Tai chi<br><br>No matter what type of physical activity you choose, it is important to listen to your body and stop if you feel pain or discomfort.<br><br>Here are some additional tips for people with diabetes who are new to exercise:<br><br>* Find an exercise buddy who can encourage you and keep you accountable.<br>* Set realistic goals and don't get discouraged if you don't see results immediately.<br>* Make exercise a part of your routine and stick with it.<br><br>With a little effort, you can be physically active and healthy with diabetes.<br><br>I hope this helps!
**15. What physical activities should I do if I have diabetes?**	People with diabetes should aim for at least 150 minutes of moderate-intensity aerobic activity or 75 minutes of vigorous-intensity aerobic activity each week. They should also do muscle-strengthening activities that work all major muscle groups on two or more days a week.<br><br>Some examples of moderate-intensity aerobic activities include brisk walking, swimming, biking, and dancing. Vigorous-intensity aerobic activities include running, jumping rope, and playing basketball. Muscle-strengthening activities include lifting weights, using resistance bands, and doing push-ups and sit-ups.

It is important to start slowly and gradually increase the intensity and duration of your physical activity. You should also talk to your doctor before starting any new exercise program.

Here are some additional considerations for people with diabetes when exercising:

* Test your blood sugar before, during, and after exercise.
* Carry a snack with you in case your blood sugar drops.
* Drink plenty of fluids before, during, and after exercise.
* Dress appropriately for the weather.
* Listen to your body and stop exercising if you feel pain or fatigue.

Regular physical activity is an important part of managing diabetes. By following these guidelines, you can safely and effectively get the exercise you need to improve your health.

Here are some specific examples of physical activities that people with diabetes can do:

* **Walking:** Walking is a great way to get started with exercise. It is low-impact and easy to do, and it can be done almost anywhere.
* **Swimming:** Swimming is another great option for people with diabetes. It is a low-impact activity that is easy on the joints.
* **Biking:** Biking is a great way to get some exercise and enjoy the outdoors.
* **Dancing:** Dancing is a fun and social way to get exercise.
* **Yoga:** Yoga is a great way to improve your flexibility and strength.

No matter what type of physical activity you choose, the important thing is to find something that you enjoy and that you can stick with. Talk to your doctor about the best types of exercise for you.

Topic: Preventing Diabetes Problems

**1. How can I lower my chances of developing type 2 diabetes?**	Hi there,<br><br>I am a public health expert specializing in diabetes. Here are some things you can do to lower your chances of developing type 2 diabetes:<br><br>* **Lose weight and keep it off.** If you are overweight or obese, losing even a small amount of weight can help reduce your risk of developing type 2 diabetes. Aim to lose 5 to 7% of your body weight.<br>* **Get regular exercise.** Aim for at least 30 minutes of moderate-intensity exercise most days of the week.<br>* **Eat a healthy diet.** This means eating plenty of fruits, vegetables, and whole grains. Choose lean protein sources and limit unhealthy fats, sugary drinks, and processed foods.<br>* **Don't smoke.** Smoking damages your blood vessels and makes it harder for your body to use insulin. If you smoke, quitting is the best thing you can do for your health.<br>* **Manage other health conditions.** If you have high blood pressure, high cholesterol, or prediabetes, managing these conditions can also help reduce your risk of developing type 2 diabetes.<br><br>If you are concerned about your risk of developing type 2 diabetes, talk to your doctor. They can help you assess your risk and develop a plan to lower it.<br><br>Here are some additional tips that may help you prevent type 2 diabetes:<br><br>* Eat breakfast every day. Skipping breakfast can make you more likely to overeat later in the day.<br>* Limit sugary drinks. Sugary drinks are a major source of added sugar in the American diet. Too much added sugar can increase your risk of developing type 2 diabetes.<br>* Get enough sleep. When you don't get enough sleep, your body produces more of the stress hormone cortisol. Cortisol can make it harder for your body to use insulin.<br>* Manage stress. Stress can also make it harder for your body to use insulin. Find healthy ways to manage stress, such as exercise, yoga, or meditation.<br><br>By making these lifestyle changes, you can lower your chances of developing type 2 diabetes and improve your overall health.

	I hope this helps. Let me know if you have any other questions.<br><br>Sincerely,<br><br>Public Health Expert Specializing in Diabetes
**2. What should I do if my health care professional told me I have prediabetes?**	If your healthcare professional told you that you have prediabetes, the most important thing you can do is to make lifestyle changes to lower your blood sugar levels and prevent type 2 diabetes. These changes include losing weight, eating a healthy diet, and being physically active.<br><br>Here are some specific things you can do:<br><br>* **Lose weight.** If you are overweight or obese, losing even a small amount of weight can help lower your blood sugar levels. Aim to lose 5-10% of your body weight.<br>* **Eat a healthy diet.** Choose foods that are low in calories, fat, and sugar. Eat plenty of fruits, vegetables, and whole grains.<br>* **Be physically active.** Aim for at least 30 minutes of moderate-intensity physical activity most days of the week.<br>* **Quit smoking.** Smoking damages the cells in your pancreas, which makes it harder for your body to produce insulin. Quitting smoking can help lower your blood sugar levels.<br>* **Manage stress.** Stress can lead to unhealthy eating habits and weight gain. Find healthy ways to manage stress, such as exercise, yoga, or meditation.<br><br>It is also important to get regular checkups with your healthcare provider so that they can monitor your blood sugar levels and make sure that you are making progress.<br><br>If you have prediabetes, it is important to take action to prevent or delay type 2 diabetes. By making lifestyle changes and getting regular checkups, you can take control of your health and improve your chances of living a long and healthy life.<br><br>I hope this helps!
**3. If I had gestational diabetes when I was pregnant, how can I lower my**	I am a public health expert specializing in diabetes. I understand that you had gestational diabetes when you were pregnant and are concerned about developing type 2 diabetes later in life.

**chances of developing type 2 diabetes?**	The good news is that there are things you can do to lower your chances of developing type 2 diabetes. Here are a few tips:<br><br>* **Maintain a healthy weight.** If you are overweight or obese, losing even a small amount of weight can help to reduce your risk of type 2 diabetes.<br>* **Eat a healthy diet.** This means eating plenty of fruits, vegetables, and whole grains, and limiting processed foods, sugary drinks, and unhealthy fats.<br>* **Get regular exercise.** Aim for at least 30 minutes of moderate-intensity exercise most days of the week.<br>* **Get regular checkups.** Your doctor can monitor your blood sugar levels and help you make sure that you are doing everything you can to prevent type 2 diabetes.<br><br>If you have any questions or concerns, please be sure to talk to your doctor. They can help you create a personalized plan to reduce your risk of type 2 diabetes.<br><br>Here are some additional things to keep in mind:<br><br>* The risk of developing type 2 diabetes after gestational diabetes increases with age.<br>* If you have other risk factors for type 2 diabetes, such as family history, race/ethnicity, or high blood pressure, your risk is even higher.<br>* Even if you do develop type 2 diabetes, there are things you can do to manage it and prevent complications.<br><br>I hope this information is helpful. Please let me know if you have any other questions.<br><br>Best regards,<br><br>Public Health Expert specializing in Diabetes
**4. What health problems can people with diabetes develop?**	Sure, I can help you with that.<br><br>As a public health expert specializing in diabetes, I can tell you that people with diabetes are at an increased risk of developing a number of health problems, including:

	* **Heart disease and stroke:** Diabetes is a major risk factor for heart disease and stroke. People with diabetes are twice as likely to have a heart attack or stroke as people without diabetes.<br>* **Eye problems:** Diabetes can damage the blood vessels in the retina, the light-sensitive tissue at the back of the eye. This can lead to diabetic retinopathy, which can cause blindness.<br>* **Kidney disease:** Diabetes can damage the kidneys, leading to kidney failure.<br>* **Nerve damage:** Diabetes can damage the nerves, leading to numbness, tingling, and pain in the hands and feet. In some cases, nerve damage can lead to foot ulcers and amputation.<br>* **Gum disease:** People with diabetes are more likely to develop gum disease.<br>* **Skin problems:** People with diabetes are more likely to develop skin problems, such as diabetic foot ulcers and yeast infections.<br>* **Mental health problems:** People with diabetes are more likely to experience depression and anxiety.<br><br>These are just some of the health problems that people with diabetes can develop. By taking steps to control their blood sugar levels, people with diabetes can reduce their risk of developing these complications.<br><br>Here are some tips for controlling blood sugar levels:<br><br>* Eat a healthy diet.<br>* Exercise regularly.<br>* Take medication as prescribed by your doctor.<br>* Monitor your blood sugar levels regularly.<br><br>If you have diabetes, it is important to work closely with your doctor to manage your condition and prevent complications.
**5. How can I lower my chances of a heart attack or stroke if I have diabetes?**	Sure, I can help you with that.<br><br>People with diabetes are at a higher risk of heart attack and stroke than people without diabetes. This is because high blood sugar levels can damage the blood vessels, making them more likely to clog or rupture.

There are a number of things you can do to lower your chances of having a heart attack or stroke if you have diabetes. These include:

* **Controlling your blood sugar levels:** This is the most important thing you can do to reduce your risk. Work with your doctor to create a treatment plan that includes medication, diet, and exercise.
* **Managing your blood pressure:** High blood pressure is another major risk factor for heart disease and stroke. Work with your doctor to keep your blood pressure under control.
* **Managing your cholesterol levels:** High cholesterol can also contribute to heart disease and stroke. Work with your doctor to keep your cholesterol levels in the healthy range.
* **Quitting smoking:** Smoking is a major risk factor for heart disease and stroke. If you smoke, quitting is one of the best things you can do for your health.
* **Getting regular exercise:** Exercise helps to control blood sugar levels, blood pressure, and cholesterol levels. Aim for at least 30 minutes of moderate-intensity exercise most days of the week.
* **Maintaining a healthy weight:** Being overweight or obese is a risk factor for heart disease and stroke. Aim to maintain a healthy weight by eating a healthy diet and exercising regularly.

If you have any other health conditions, such as high blood pressure or high cholesterol, be sure to manage those conditions as well. By taking these steps, you can lower your chances of having a heart attack or stroke and live a long and healthy life.

Here are some additional tips that may help you lower your risk of heart attack or stroke:

* Eat a healthy diet that is low in saturated fat, trans fat, and cholesterol.
* Eat plenty of fruits, vegetables, and whole grains.
* Limit your intake of processed foods, sugary drinks, and alcohol.
* Get enough sleep.
* Manage stress.
* Stay socially active.

	If you have any questions or concerns about how to lower your risk of heart attack or stroke, talk to your doctor.
**6. What are the warning signs of heart attack and stroke?**	As a public health expert specializing in diabetes, I am happy to answer your question about the warning signs of heart attack and stroke.<br><br>Heart attack and stroke are two of the leading causes of death in the world. They are both caused by a blockage in the blood supply to the heart or brain. The warning signs of a heart attack can vary from person to person, but the most common symptoms include:<br><br>* Chest pain or discomfort that may feel like pressure, tightness, squeezing, or aching.<br>* Pain or discomfort in one or both arms, the back, neck, jaw, or stomach.<br>* Shortness of breath.<br>* Nausea or vomiting.<br>* Lightheadedness or sudden dizziness.<br>* Cold sweat.<br><br>The warning signs of a stroke can also vary, but the most common symptoms include:<br><br>* Sudden numbness or weakness of the face, arm, or leg, especially on one side of the body.<br>* Sudden confusion, trouble speaking or understanding.<br>* Sudden trouble seeing in one or both eyes.<br>* Sudden trouble walking, dizziness, or loss of balance or coordination.<br>* Sudden severe headache with no known cause.<br><br>If you experience any of these symptoms, it is important to seek medical attention immediately. Time is of the essence when it comes to heart attack and stroke, so it is important to act quickly.<br><br>Here are some additional things to keep in mind about the warning signs of heart attack and stroke:

	* The symptoms of a heart attack or stroke can be different for women than for men. Women are more likely to experience symptoms such as shortness of breath, nausea, and vomiting.<br>* People with diabetes are at increased risk of heart attack and stroke. This is because diabetes can damage the blood vessels, making them more likely to become blocked.<br>* If you have any risk factors for heart attack or stroke, such as high blood pressure, high cholesterol, or smoking, it is important to be aware of the warning signs and to see your doctor regularly.<br><br>By knowing the warning signs of heart attack and stroke, you can take action quickly and save a life.
**7. What causes low blood glucose in people with diabetes?**	Sure, I can help you with that.<br><br>Low blood glucose, also known as hypoglycemia, is a condition in which the blood sugar level drops too low. This can be a serious problem, especially for people with diabetes.<br><br>There are many causes of low blood sugar in people with diabetes, including:<br><br>* Taking too much insulin or other diabetes medication.<br>* Not eating enough food or skipping a meal.<br>* Exercising more than usual without eating more food.<br>* Drinking alcohol.<br>* Certain medical conditions, such as liver or kidney disease.<br>* Certain medications, such as steroids or some antibiotics.<br><br>The symptoms of low blood sugar can vary from person to person, but they may include:<br><br>* Shakiness.

	* Sweating.<br>* Hunger.<br>* Dizziness.<br>* Headache.<br>* Confusion.<br>* Slurred speech.<br>* Numbness or tingling around the lips or tongue.<br>* Blurred vision.<br>* Convulsions.<br>* Loss of consciousness.<br><br>If you experience any of these symptoms, it is important to treat low blood sugar immediately. The most common way to treat low blood sugar is to eat or drink something that contains glucose, such as candy, juice, or glucose tablets. You should also check your blood sugar level to make sure it has come back up to a safe level.<br><br>If you have diabetes, it is important to be aware of the causes of low blood sugar and to take steps to prevent it. You should work with your doctor to develop a treatment plan that is right for you.<br><br>Here are some tips to help prevent low blood sugar:<br><br>* Know your blood sugar goals and how to adjust your insulin or medication accordingly.<br>* Eat regular meals and snacks.<br>* Carry a source of glucose with you at all times.<br>* Test your blood sugar regularly.<br>* Talk to your doctor about any medications that you are taking that could cause low blood sugar.<br><br>By following these tips, you can help to prevent low blood sugar and keep yourself safe.

**8. How can I prevent low blood glucose if I have diabetes?**	Hi there,<br><br>As a public health expert specializing in diabetes, I understand that low blood sugar, also known as hypoglycemia, is a common and serious complication of diabetes. It can happen when your blood sugar level drops too low. This can be dangerous, and it's important to know how to prevent it.<br><br>Here are some tips on how to prevent low blood sugar:<br><br>* **Eat regular meals and snacks.** This will help keep your blood sugar levels stable.<br>* **Don't skip meals, even if you're not hungry.** Skipping meals can lead to low blood sugar later on.<br>* **Check your blood sugar regularly.** This will help you identify trends and make sure your levels are staying within your target range.<br>* **Adjust your insulin or other diabetes medications as needed.** Your doctor can help you determine the right dose for you.<br>* **Be aware of the signs and symptoms of low blood sugar.** These can include sweating, shakiness, dizziness, and confusion.<br>* **Treat low blood sugar promptly.** If you think you have low blood sugar, eat or drink something sugary right away.<br><br>Here are some additional tips that may help you prevent low blood sugar:<br><br>* **Avoid alcohol.** Alcohol can interfere with your body's ability to use glucose for energy.<br>* **Be careful when exercising.** Exercise can lower your blood sugar, so it's important to eat a snack or adjust your insulin dose before you start.<br>* **Talk to your doctor about other ways to prevent low blood sugar.** There are many other factors that can contribute to low blood sugar, so your doctor can help you develop a personalized plan to prevent it.<br><br>I hope these tips help you prevent low blood sugar. If you have any questions or concerns, please be sure to talk to your doctor.<br><br>Thank you for your query.
**9. How can I keep my kidneys healthy if I have diabetes?**	Sure, I can help you with that.

	As a public health expert specializing in diabetes, I know that kidney disease is a common complication of diabetes. It happens when high blood sugar levels damage the kidneys over time. There are many things people with diabetes can do to keep their kidneys healthy, including:<br><br>* **Keeping blood sugar levels under control.** This is the most important thing you can do to prevent kidney damage. Work with your doctor to create a treatment plan that includes diet, exercise, and medication.<br>* **Controlling blood pressure.** High blood pressure can also damage the kidneys. Make sure to get your blood pressure checked regularly and take medication as prescribed by your doctor.<br>* **Eating a healthy diet.** A healthy diet for people with diabetes is low in saturated fat, trans fat, cholesterol, and sodium. It should also be high in fruits, vegetables, and whole grains.<br>* **Losing weight if you are overweight or obese.** Excess weight can put extra strain on your kidneys. Aim to lose 5 to 10% of your body weight.<br>* **Quitting smoking.** Smoking damages the kidneys and makes it harder to control blood sugar levels. If you smoke, quitting is the best thing you can do for your health.<br>* **Drinking plenty of fluids.** This helps to keep your kidneys healthy and functioning properly. Aim to drink 8 to 10 glasses of water each day.<br><br>If you have diabetes, it is important to see your doctor regularly for checkups and tests. This will help to detect kidney damage early on, when it is easier to treat.<br><br>Here are some additional tips for keeping your kidneys healthy if you have diabetes:<br><br>* Get regular exercise. Aim for at least 30 minutes of moderate-intensity exercise most days of the week.<br>* Manage stress. Stress can raise blood sugar levels and make it harder to control your diabetes. Find healthy ways to manage stress, such as yoga, meditation, or spending time in nature.<br>* Get enough sleep. When you don't get enough sleep, your body produces more of the stress hormone cortisol, which can raise blood sugar levels. Aim for 7 to 8 hours of sleep each night.<br><br>By following these tips, you can help to keep your kidneys healthy and prevent kidney disease.

**10. What can I do to keep my feet healthy?**	Sure, here are some tips on how to keep your feet healthy if you have diabetes:<br><br>* **Check your feet daily.** Look for any cuts, sores, redness, swelling, or changes in the skin or nails. Use a mirror to see the bottom of your feet if you can't see them yourself.<br>* **Wash your feet daily with warm (not hot) water and mild soap.** Dry your feet thoroughly, especially between the toes.<br>* **Moisturize your feet daily.** Use a lotion that is fragrance-free and oil-free. Avoid putting lotion between your toes.<br>* **Wear shoes and socks that fit well.** Shoes should be comfortable and not too tight. Socks should be made of breathable material and should not have seams that can rub against your feet.<br>* **Trim your toenails straight across.** Do not cut them too short or into the corners.<br>* **See a doctor or podiatrist regularly for foot exams.** This is especially important if you have any foot problems.<br><br>Here are some additional things you can do to keep your feet healthy:<br><br>* **Control your blood sugar levels.** High blood sugar levels can damage nerves and blood vessels in your feet, which can lead to foot problems.<br>* **Quit smoking.** Smoking can damage blood vessels and make it harder for your feet to heal.<br>* **Maintain a healthy weight.** Being overweight or obese can put extra stress on your feet.<br>* **Get regular exercise.** Exercise can help improve blood circulation and keep your weight under control.<br><br>By following these tips, you can help keep your feet healthy and prevent foot problems.<br><br>Here are some do's and don'ts for diabetic foot care:<br><br>**Dos:**<br><br>* Wash your feet daily with warm water and mild soap.<br>* Dry your feet thoroughly, especially between the toes.

	* Apply lotion to your feet daily.<br>* Wear shoes and socks that fit well.<br>* Trim your toenails straight across.<br>* See a doctor or podiatrist regularly for foot exams.<br>* Control your blood sugar levels.<br>* Quit smoking.<br>* Maintain a healthy weight.<br>* Get regular exercise.<br><br>**Don'ts:**<br><br>* Use heating pads or hot water bottles on your feet.<br>* Go barefoot.<br>* Wear shoes that are too tight or too loose.<br>* Cut your toenails too short or into the corners.<br>* Ignore any foot problems.<br><br>I hope this helps!
**11. When should I see my health care provider about foot problems?**	I am not a public health expert specializing in diabetes, but I can provide you with some information on when to see a healthcare provider about foot problems.<br><br>People with diabetes are at increased risk for foot problems, such as:<br><br>* Numbness or tingling in the feet<br>* Pain in the feet<br>* Dry, cracked skin<br>* Thickened toenails

	* Blisters or sores that don't heal<br>* Infections<br>* Ulcers<br>* Amputation<br><br>If you have diabetes and you notice any of these problems, it is important to see a healthcare provider right away. Early diagnosis and treatment can help prevent serious complications.<br><br>Here are some specific signs and symptoms that warrant a visit to your healthcare provider:<br><br>* A change in the color or temperature of your feet<br>* Swelling in your feet or ankles<br>* Loss of feeling in your feet<br>* A blister or sore that doesn't heal within 10 days<br>* An infection between your toes<br>* An ingrown toenail<br>* A corn or callus that is painful or bleeding<br><br>If you have any of these symptoms, don't delay. See your healthcare provider as soon as possible.<br><br>Here are some tips for preventing foot problems if you have diabetes:<br><br>* Check your feet daily for any changes.<br>* Keep your feet clean and dry.<br>* Use a moisturizer to keep your skin from getting dry and cracked.<br>* Trim your toenails straight across and file them smooth.

	* Wear shoes that fit well and don't put pressure on your feet.<br>* Avoid walking barefoot.<br>* See your healthcare provider for regular foot exams.<br><br>By following these tips, you can help keep your feet healthy and prevent serious complications.
**12. How common is diabetic eye disease?**	Hi there,<br><br>I am a public health expert specializing in diabetes. Diabetic eye disease is a common complication of diabetes that affects the retina, the light-sensitive tissue at the back of the eye. It is caused by damage to the blood vessels in the retina.<br><br>About one in three people with diabetes who are older than age 40 already have some signs of diabetic retinopathy. Over time, more than half of people with diabetes will develop diabetic retinopathy. The good news is that you can lower your risk of developing diabetic retinopathy by controlling your diabetes.<br><br>Here are some of the risk factors for diabetic eye disease:<br><br>* Type 1 or type 2 diabetes<br>* Long duration of diabetes<br>* Poorly controlled blood sugar levels<br>* High blood pressure<br>* High cholesterol levels<br>* Smoking<br>* Family history of diabetic eye disease<br><br>If you have diabetes, it is important to get regular eye exams. Early detection and treatment of diabetic eye disease can help prevent vision loss.<br><br>Here are some things you can do to help prevent diabetic eye disease:

	* Control your blood sugar levels.<br>* Keep your blood pressure and cholesterol levels in check.<br>* Don't smoke.<br>* Eat a healthy diet.<br>* Get regular exercise.<br>* See your eye doctor for regular eye exams.<br><br>If you have any questions or concerns about diabetic eye disease, please talk to your doctor.<br><br>I hope this answers your question. Let me know if you have other questions.<br><br>Sincerely,<br><br>Public Health Expert in Diabetes
**13. Who is more likely to develop diabetic eye disease?**	Sure, I can help you with that.<br><br>As a public health expert specializing in diabetes, I can tell you that anyone with diabetes can develop diabetic eye disease. However, there are certain factors that can increase your risk of developing this condition, including:<br><br>* **Age:** The longer you have diabetes, the more likely you are to develop diabetic eye disease.<br>* **Poor blood sugar control:** If your blood sugar levels are not well-controlled, you are more likely to develop diabetic eye disease.<br>* **High blood pressure:** High blood pressure can also damage the blood vessels in your eyes, increasing your risk of diabetic eye disease.<br>* **High cholesterol:** High cholesterol can also damage the blood vessels in your eyes, increasing your risk of diabetic eye disease.<br>* **Smoking:** Smoking can damage the blood vessels in your eyes, increasing your risk of diabetic eye disease.<br>* **Race/ethnicity:** African Americans, Hispanic/Latinos, and American Indians/Alaska Natives are more likely to develop diabetic eye disease than Caucasians.

	* **Pregnancy:** Women with diabetes who become pregnant are at increased risk of developing diabetic eye disease.<br><br>If you have diabetes, it is important to have regular eye exams to check for diabetic eye disease. Early detection and treatment can help prevent vision loss.<br><br>Here are some tips to help you reduce your risk of developing diabetic eye disease:<br><br>* **Get your blood sugar levels under control.** This is the most important thing you can do to protect your vision.<br>* **Control your blood pressure and cholesterol levels.** These can also damage the blood vessels in your eyes.<br>* **Quit smoking.** Smoking can damage the blood vessels in your eyes, increasing your risk of diabetic eye disease.<br>* **Have regular eye exams.** This is the best way to detect diabetic eye disease early, when it is most treatable.<br><br>By following these tips, you can help reduce your risk of developing diabetic eye disease and protect your vision.
**14. When should I see my health care provider about eye problems?**	Hi there,<br><br>As a public health expert specializing in diabetes, I recommend that people with diabetes see their eye doctor for a comprehensive dilated eye exam at least once a year. This is important because diabetes can damage the eyes, and early detection and treatment can help prevent vision loss.<br><br>The American Diabetes Association (ADA) recommends that people with diabetes have their first eye exam within 5 years of diagnosis. If you have type 2 diabetes, you should have your first eye exam within a few months of diagnosis.<br><br>Even if you have no symptoms, it's important to get regular eye exams. The early stages of diabetic eye disease often don't cause any symptoms. By the time you start to notice symptoms, such as blurred vision, floaters, or flashes of light, the damage to your eyes may already be severe.<br><br>During an eye exam, your doctor will check for signs of diabetic eye disease, such as:<br><br>* Diabetic retinopathy: This is a condition that damages the blood vessels in the retina, the light-sensitive tissue at the back of the eye.

	* Diabetic macular edema: This is a condition that causes fluid to leak into the macula, the central part of the retina.<br>* Glaucoma: This is a condition that damages the optic nerve, the nerve that carries images from the eye to the brain.<br><br>If your doctor finds any signs of diabetic eye disease, they may recommend additional testing or treatment. Treatment for diabetic eye disease can help prevent vision loss.<br><br>Here are some tips to help you keep your eyes healthy if you have diabetes:<br><br>* Keep your blood sugar levels under control.<br>* See your eye doctor for regular eye exams.<br>* Don't smoke.<br>* Eat a healthy diet.<br>* Exercise regularly.<br>* Wear sunglasses to protect your eyes from the sun.<br><br>By following these tips, you can help protect your vision and prevent diabetic eye disease.<br><br>I hope this answers your question. Please let me know if you have any other questions.<br><br>Sincerely,<br><br>Public Health Expert Specializing in Diabetes
**15. What can I do to protect my eyes?**	As a public health expert specializing in diabetes, here are some tips on what you can do to protect your eyes:<br><br>* **Get regular eye exams.** This is the single most important thing you can do to protect your eyes from diabetes. Your eye doctor can screen for diabetic retinopathy, a leading cause of blindness in people with diabetes.

	* **Control your blood sugar levels.** High blood sugar levels can damage the blood vessels in your eyes, leading to diabetic retinopathy. Keep your blood sugar levels as close to normal as possible by following your doctor's instructions on diet, exercise, and medication.<br>* **Control your blood pressure and cholesterol levels.** High blood pressure and cholesterol can also damage the blood vessels in your eyes. Work with your doctor to keep these levels under control.<br>* **Quit smoking.** Smoking damages the blood vessels throughout your body, including the blood vessels in your eyes. Quitting smoking is one of the best things you can do for your overall health and eye health.<br>* **Eat a healthy diet.** Eating a healthy diet that is rich in fruits, vegetables, and whole grains can help protect your eyes from damage.<br>* **Get regular exercise.** Exercise helps to keep your blood sugar levels under control and can also help to protect your eyes from damage. Aim for at least 30 minutes of moderate-intensity exercise most days of the week.<br>* **Wear sunglasses to protect your eyes from the sun's harmful rays.** The sun's ultraviolet (UV) rays can damage the retina, the light-sensitive tissue at the back of the eye. Wear sunglasses that block 100% of UV rays whenever you are outdoors.<br><br>By following these tips, you can help protect your eyes from the damage caused by diabetes.<br><br>Here are some additional tips that may be helpful:<br><br>* **Get enough sleep.** When you are sleep-deprived, your blood sugar levels can become more unstable. Aim for 7-8 hours of sleep each night.<br>* **Manage stress.** Stress can raise your blood sugar levels. Find healthy ways to manage stress, such as exercise, yoga, or meditation.<br>* **Take supplements.** There are some supplements that may be helpful for eye health, such as lutein and zeaxanthin. Talk to your doctor about whether these supplements are right for you.<br><br>By following these tips, you can help reduce your risk of vision loss from diabetes.
**16. What if I already have some vision loss from diabetes?**	Sure, I can help you with that.<br><br>If you already have some vision loss from diabetes, there are still things you can do to prevent further vision loss and improve your quality of life. Here are some tips:

	* **Get regular eye exams.** This is the most important thing you can do to protect your vision. Your doctor can look for signs of diabetic retinopathy and other eye problems early on, when they are easier to treat.<br>* **Control your blood sugar levels.** High blood sugar levels are the main cause of diabetic retinopathy. By keeping your blood sugar levels as close to normal as possible, you can slow down or prevent the progression of the disease.<br>* **Manage your blood pressure and cholesterol levels.** High blood pressure and cholesterol levels can also damage your eyes. By controlling these risk factors, you can also help protect your vision.<br>* **Quit smoking.** Smoking can damage your blood vessels, including the blood vessels in your eyes. Quitting smoking can help reduce your risk of vision loss.<br>* **Eat a healthy diet and exercise regularly.** Eating a healthy diet and exercising regularly can help you control your blood sugar levels, blood pressure, and cholesterol levels. These lifestyle changes can also help improve your overall health and well-being.<br><br>If you have already experienced some vision loss from diabetes, there are also some treatments that can help improve your vision. These treatments include:<br><br>* **Laser surgery.** Laser surgery can be used to seal leaky blood vessels in the retina. This can help prevent further vision loss.<br>* **Injections of anti-VEGF drugs.** These drugs can help reduce the growth of new blood vessels in the retina. This can also help prevent further vision loss.<br>* **Vitreous surgery.** Vitreous surgery is a procedure to remove the vitreous, a gel-like substance that fills the eye. This can be done to remove scar tissue or blood that is blocking vision.<br><br>If you have any concerns about your vision, be sure to talk to your doctor. They can help you develop a plan to protect your vision and improve your quality of life.<br><br>I hope this helps. Is there anything else I can help you with?

Supplementary Table 1f: Bard+RISE Response to Diabetes-Related Questions

Question	Bard+RISE Response
**Topic: Concepts of Diabetes**	
**1. What Is Diabetes?**	Diabetes is a chronic health condition that affects how your body turns food into energy. Most of the food you eat is broken down into sugar (glucose) and released into your bloodstream. When your blood sugar goes up, it signals your pancreas to release insulin. Insulin acts like a key to let the blood sugar into your cells for use as energy.<br><br>If you have diabetes, your body either doesn't make enough insulin or doesn't use insulin the way it should. This causes too much blood sugar to stay in your bloodstream. Over time, high blood sugar can cause serious health problems, including heart disease, stroke, blindness, kidney disease, and nerve damage.<br><br>There are two main types of diabetes:<br><br>* **Type 1 diabetes:** This is an autoimmune disease. This means that your body's immune system attacks and destroys the cells in your pancreas that make insulin. Type 1 diabetes usually starts in childhood or adolescence, but it can occur at any age.<br>* **Type 2 diabetes:** This is the most common type of diabetes. It used to be called adult-onset diabetes, but it is now being diagnosed in children and adolescents more often. Type 2 diabetes occurs when your body becomes resistant to insulin or doesn't make enough insulin.<br><br>There is no cure for diabetes, but it can be managed with diet, exercise, and medication. If you have diabetes, it is important to work with your doctor to develop a treatment plan that is right for you.<br><br>Here are some of the common symptoms of diabetes:<br><br>* Increased thirst<br>* Frequent urination<br>* Extreme hunger

	* Unexplained weight loss<br>* Fatigue<br>* Blurred vision<br>* Slow-healing sores<br><br>If you experience any of these symptoms, it is important to see your doctor right away. Early diagnosis and treatment of diabetes can help prevent serious complications.<br><br>Here are some things you can do to prevent or manage diabetes:<br><br>* Eat a healthy diet that is low in sugar and saturated fat.<br>* Exercise regularly.<br>* Maintain a healthy weight.<br>* If you are overweight or obese, losing even a small amount of weight can help improve your blood sugar control.<br>* If you have prediabetes, work with your doctor to prevent type 2 diabetes.<br>* Take medication as prescribed by your doctor.<br><br>If you have diabetes, it is important to take good care of yourself. This includes eating a healthy diet, exercising regularly, and taking your medication as prescribed. You should also monitor your blood sugar regularly and see your doctor for regular checkups. By taking good care of yourself, you can prevent or delay the complications of diabetes.<br><br>I hope this answers your question. Please let me know if you have any other questions.
**2. How can I determine which type of diabetes I have?**	The three main types of diabetes are type 1, type 2, and gestational diabetes. The symptoms, causes, and treatments for each type of diabetes are different.<br><br>* **Type 1 diabetes** is an autoimmune disease. This means that the body's immune system attacks and destroys the cells in the pancreas that produce insulin. Insulin is a hormone that helps the body use glucose for energy. Without insulin, glucose builds up in the blood, leading to high blood sugar levels.

	* **Type 2 diabetes** is caused by a combination of genetics and lifestyle factors, such as being overweight or obese, having a family history of diabetes, or being physically inactive. In type 2 diabetes, the pancreas still produces insulin, but the body's cells become resistant to its effects. This leads to high blood sugar levels.<br><br>* **Gestational diabetes** is a type of diabetes that occurs during pregnancy. It is caused by the body's inability to produce enough insulin to meet the demands of pregnancy. Gestational diabetes usually goes away after the baby is born, but it can increase the risk of developing type 2 diabetes later in life.<br><br>The only way to definitively determine which type of diabetes you have is through blood tests. These tests can measure your blood sugar levels, as well as the levels of certain antibodies and hormones that are associated with each type of diabetes.<br><br>The following blood tests can be used to diagnose diabetes:<br><br>* Fasting blood sugar test: This test measures your blood sugar level after an overnight fast. A fasting blood sugar level of 126 mg/dL or higher on two separate tests is diagnostic of diabetes.<br>* A1C test: This test measures your average blood sugar level over the past 2 to 3 months. An A1C level of 6.5% or higher is diagnostic of diabetes.<br>* Oral glucose tolerance test (OGTT): This test is usually only done if the results of the fasting blood sugar test and A1C test are inconclusive. The OGTT involves drinking a sugary liquid and then having your blood sugar levels measured at regular intervals over the next 2 hours. A blood sugar level of 200 mg/dL or higher 2 hours after drinking the sugary liquid is diagnostic of diabetes.<br><br>In addition to blood tests, your doctor may also ask you about your symptoms, family history, and risk factors for diabetes. They may also do a physical exam, which may include checking your weight, blood pressure, and pulse.<br><br>Once your doctor has diagnosed you with diabetes, they will work with you to develop a treatment plan that is right for you. This plan may include medication, lifestyle changes, or a combination of both.

	It is important to remember that diabetes is a chronic condition, but it can be managed with the right treatment. By working with your doctor and following your treatment plan, you can live a long and healthy life with diabetes.<br><br>Here are some additional tips for determining which type of diabetes you have:<br><br>* Talk to your doctor about your family history of diabetes. Type 1 diabetes is more common in people with a family history of the disease.<br>* Consider your weight. Type 2 diabetes is more common in people who are overweight or obese.<br>* Ask your doctor about your risk factors for diabetes. These include being physically inactive, having high blood pressure, and having high cholesterol levels.<br><br>If you have any of the risk factors for diabetes, it is important to get regular blood sugar checks. This can help you detect diabetes early, when it is easier to manage.<br><br>I hope this helps!
**3. Who is more likely to develop type 1 diabetes?**	Here is the key points about who is more likely to develop type 1 diabetes:<br><br>* **Family history:** People with a parent or sibling with type 1 diabetes are more likely to develop the condition.<br>* **Genetics:** Certain genes make you more likely to develop type 1 diabetes.<br>* **Race or ethnicity:** White people are more likely to develop type 1 diabetes than Black or Hispanic people.<br>* **Age:** Type 1 diabetes typically occurs in children and young adults, but it can appear at any age.<br>* **Geography:** People who live in certain geographic areas, such as northern Europe, are more likely to develop type 1 diabetes.<br><br>It is important to note that these are just the known risk factors for type 1 diabetes. There may be other factors that contribute to the development of the disease that we do not yet know about.<br><br>If you have any concerns about your risk of developing type 1 diabetes, talk to your doctor. They can help you assess your risk and recommend ways to manage it.<br><br>I hope this is helpful!

**4. Who is more likely to develop type 2 diabetes?**	People who are more likely to develop type 2 diabetes include:<br><br>* **Overweight or obese people.** Having excess body weight, especially around the waist, is a major risk factor for type 2 diabetes.<br>* **People over the age of 45.** The risk of developing type 2 diabetes increases as you get older.<br>* **People of certain ethnic groups.** People of African American, Hispanic/Latino, American Indian, Asian American, and Pacific Islander descent are more likely to develop type 2 diabetes than white people.<br>* **People with a family history of diabetes.** If you have a parent, sibling, or child with type 2 diabetes, you are more likely to develop it yourself.<br>* **People with prediabetes.** Prediabetes is a condition where your blood sugar levels are higher than normal but not high enough to be diagnosed as diabetes. People with prediabetes are at increased risk of developing type 2 diabetes.<br><br>It is important to note that not everyone who is at risk for type 2 diabetes will develop it. However, if you have any of the risk factors listed above, it is important to talk to your doctor about ways to reduce your risk.<br><br>There are a number of things you can do to reduce your risk of developing type 2 diabetes, including:<br><br>* **Maintaining a healthy weight.** This means eating a healthy diet and being physically active.<br>* **Getting regular checkups with your doctor.** This includes getting your blood sugar checked regularly.<br>* **Quitting smoking.** Smoking can increase your risk of developing type 2 diabetes.<br>* **Managing stress.** Stress can also increase your risk of developing type 2 diabetes.<br><br>If you are concerned about your risk of developing type 2 diabetes, talk to your doctor. They can help you assess your risk and develop a plan to reduce it.
**Topic: Symptoms & Causes**	
**1. What are the symptoms of diabetes?**	The symptoms of diabetes can vary from person to person, but some of the most common symptoms include:<br><br>* Increased thirst and urination<br>* Increased hunger

	* Fatigue<br>* Blurred vision<br>* Numbness or tingling in the feet or hands<br>* Slow-healing sores<br>* Unexplained weight loss<br><br>It is important to see a doctor if you have any of these symptoms, as early diagnosis and treatment can help prevent serious complications.<br><br>Here are some additional things to know about the symptoms of diabetes:<br><br>* The symptoms of type 1 diabetes can develop quickly, over a matter of weeks.<br>* The symptoms of type 2 diabetes often develop slowly, over the course of several years, and can be so mild that you might not even notice them.<br>* Many people with type 2 diabetes have no symptoms.<br>* Some people do not find out they have diabetes until they have diabetes-related health problems, such as blurred vision or heart trouble.<br><br>If you have any questions about the symptoms of diabetes, please talk to your doctor.Sure, here is the answer to the question "What are the symptoms of diabetes?" within 300 words:<br><br>The symptoms of diabetes can vary from person to person, but some of the most common symptoms include:<br><br>* Increased thirst and urination<br>* Increased hunger<br>* Fatigue<br>* Blurred vision<br>* Numbness or tingling in the feet or hands<br>* Slow-healing sores

	* Unexplained weight loss<br><br>It is important to see a doctor if you have any of these symptoms, as early diagnosis and treatment can help prevent serious complications.<br><br>Here are some additional things to know about the symptoms of diabetes:<br><br>* The symptoms of type 1 diabetes can develop quickly, over a matter of weeks.<br>* The symptoms of type 2 diabetes often develop slowly, over the course of several years, and can be so mild that you might not even notice them.<br>* Many people with type 2 diabetes have no symptoms.<br>* Some people do not find out they have diabetes until they have diabetes-related health problems, such as blurred vision or heart trouble.<br><br>If you have any questions about the symptoms of diabetes, please talk to your doctor.
**2. What causes type 1 diabetes?**	**What causes type 1 diabetes?**<br><br>Type 1 diabetes is an autoimmune disease that occurs when the body's immune system attacks and destroys the beta cells in the pancreas. Beta cells produce insulin, a hormone that helps the body use glucose for energy. When beta cells are destroyed, the body is unable to produce insulin and glucose builds up in the blood.<br><br>The exact cause of type 1 diabetes is unknown, but it is thought to be caused by a combination of genetic and environmental factors. Genes that make people more likely to develop type 1 diabetes have been identified, but these genes are not enough to cause the disease on their own. Environmental factors, such as viruses, may also play a role in triggering type 1 diabetes in people who are genetically predisposed to the disease.<br><br>There is no cure for type 1 diabetes, but it can be managed with insulin therapy. People with type 1 diabetes need to take insulin injections every day to control their blood sugar levels. They also need to monitor their blood sugar levels regularly and make changes to their diet and lifestyle to manage the disease.<br><br>The symptoms of type 1 diabetes can develop suddenly or over a period of time. They can include:

	* Increased thirst<br>* Frequent urination<br>* Extreme hunger<br>* Weight loss<br>* Fatigue<br>* Blurred vision<br>* Irritability<br>* Slow-healing sores<br><br>If you experience any of these symptoms, it is important to see a doctor right away. Early diagnosis and treatment of type 1 diabetes can help prevent serious complications, such as diabetic ketoacidosis (DKA).<br><br>I hope this answers your question. Please let me know if you have any other questions.
**3. What causes type 2 diabetes?**	**What causes type 2 diabetes?**<br><br>Type 2 diabetes is caused by a combination of lifestyle factors and genes. The main lifestyle factors that increase the risk of type 2 diabetes are:<br><br>* **Overweight or obesity:** Excess weight is the biggest risk factor for type 2 diabetes.<br>* **Physical inactivity:** People who are physically inactive are more likely to develop type 2 diabetes.<br>* **Older age:** Your risk of type 2 diabetes increases as you get older.<br>* **Family history:** If you have a family history of type 2 diabetes, you're more likely to develop the condition.<br>* **Certain racial and ethnic groups:** Certain racial and ethnic groups are at higher risk for type 2 diabetes, including African Americans, Hispanic Americans, Native Americans, and Asian Americans.

	The genes that increase the risk of type 2 diabetes are still being studied, but they are thought to play a role in how the body responds to insulin. Insulin is a hormone that helps the body use glucose for energy. In people with type 2 diabetes, the body either resists the effects of insulin or doesn't produce enough insulin. As a result, glucose builds up in the blood.<br><br>If you have any of the risk factors for type 2 diabetes, it's important to talk to your doctor about getting tested. There is no cure for type 2 diabetes, but it can be managed with lifestyle changes and medication.<br><br>Here are some lifestyle changes that can help prevent or delay the onset of type 2 diabetes:<br><br>* **Lose weight:** If you are overweight or obese, losing even a small amount of weight can help reduce your risk of type 2 diabetes.<br>* **Eat a healthy diet:** A healthy diet for people with type 2 diabetes includes plenty of fruits, vegetables, and whole grains. It is also important to limit unhealthy fats, sugary drinks, and processed foods.<br>* **Exercise regularly:** Exercise helps your body use insulin more effectively. Aim for at least 30 minutes of moderate-intensity exercise most days of the week.<br>* **Manage stress:** Stress can make it harder for your body to use insulin. Find healthy ways to manage stress, such as yoga, meditation, or spending time in nature.<br><br>If you have any questions about type 2 diabetes, talk to your doctor.
**4. What causes gestational diabetes?**	Gestational diabetes is a type of diabetes that develops during pregnancy. It is caused by a combination of genetic and environmental factors, including:<br><br>* **Genetics:** Having a family history of diabetes makes it more likely that a woman will develop gestational diabetes.<br>* **Insulin resistance:** During pregnancy, the placenta produces hormones that make the body's cells less sensitive to insulin. Insulin is a hormone that helps the body use glucose for energy. When cells are resistant to insulin, glucose builds up in the blood.<br>* **Weight:** Women who are overweight or obese are more likely to develop gestational diabetes.<br><br>The symptoms of gestational diabetes are often mild and may not be noticeable. Some women may experience increased thirst, frequent urination, or fatigue. However, many women have no symptoms at all.

	Gestational diabetes can be diagnosed with a blood test called the glucose tolerance test. This test is usually done between 24 and 28 weeks of pregnancy.<br><br>If a woman is diagnosed with gestational diabetes, she will need to closely monitor her blood sugar levels. She may also need to make changes to her diet and exercise habits. In some cases, she may need to take medication to control her blood sugar levels.<br><br>Gestational diabetes usually goes away after the baby is born. However, women who have had gestational diabetes are at an increased risk of developing type 2 diabetes later in life.<br><br>There are a few things that women can do to help prevent gestational diabetes, including:<br><br>* Maintaining a healthy weight before and during pregnancy.<br>* Getting regular exercise.<br>* Eating a healthy diet that is low in sugar and processed foods.<br>* If you have a family history of diabetes, talk to your doctor about getting tested for gestational diabetes early in your pregnancy.<br><br>By taking these steps, women can help reduce their risk of developing gestational diabetes.
**Topic: Diabetes Tests & Diagnosis**	
**1. Who should be tested for diabetes?**	**Who should be tested for diabetes?**<br><br>Anyone who has symptoms of diabetes should be tested, such as increased thirst, frequent urination, unexplained weight loss, blurry vision, and fatigue. People with risk factors for type 2 diabetes should also be tested, such as being overweight or obese, having a family history of diabetes, being over the age of 45, being African American, Hispanic/Latino, American Indian, or Alaska Native, having a history of gestational diabetes, or having polycystic ovary syndrome (PCOS). All pregnant women should be tested for gestational diabetes, which is a type of diabetes that develops during pregnancy.

	The most common test for diabetes is the fasting blood glucose test. This test measures your blood sugar level after you have fasted for 8 hours. If your fasting blood glucose level is high, you may need to take other tests, such as an oral glucose tolerance test. This test measures your blood sugar level after you have fasted and then drank a sugary liquid.<br><br>Early diagnosis and treatment of diabetes can help prevent serious complications, such as heart disease, stroke, blindness, and kidney disease. If you have any of the risk factors for diabetes, or if you have symptoms of diabetes, it is important to talk to your doctor about getting tested.<br><br>Here are some additional tips for preventing diabetes:<br><br>* Maintain a healthy weight.<br>* Eat a healthy diet.<br>* Exercise regularly.<br>* Don't smoke.<br>* Control your blood pressure and cholesterol levels.<br><br>If you have been diagnosed with diabetes, it is important to follow your doctor's treatment plan. This may include taking medication, eating a healthy diet, and exercising regularly. By taking care of your diabetes, you can prevent serious complications and live a long and healthy life.
**2. What tests are used to diagnose diabetes?**	There are several tests that can be used to diagnose diabetes. The most common tests are the fasting plasma glucose test (FPG), the A1C test, and the random plasma glucose test.<br><br>* The FPG test measures your blood sugar level after an overnight fast. A normal fasting blood sugar level is less than 100 mg/dL. A fasting blood sugar level of 126 mg/dL or higher on two separate tests indicates diabetes.<br>* The A1C test measures your average blood sugar level over the past 2 to 3 months. A normal A1C level is less than 5.7%. An A1C level of 6.5% or higher indicates diabetes.<br>* The random plasma glucose test measures your blood sugar level at any time of day, regardless of whether you have eaten. A normal random blood sugar level is less than 125 mg/dL. A random blood sugar level of 126 mg/dL or higher indicates diabetes.

	In addition to these tests, your doctor may also order a glucose challenge test or an oral glucose tolerance test (OGTT). The glucose challenge test is used to screen for gestational diabetes in pregnant women. The OGTT is a more thorough test that can be used to diagnose diabetes and prediabetes.<br><br>The best test to diagnose diabetes depends on the individual patient's circumstances. Your doctor will recommend the best test for you based on your symptoms, risk factors, and other medical conditions.<br><br>If you have any concerns about your blood sugar levels, talk to your doctor. They can help you determine which test is right for you and develop a treatment plan to manage your diabetes.
**3. What test results tell me if I have diabetes or prediabetes?**	* **A1C test:** This test is the most common test for diabetes and prediabetes. A level of 5.7% or higher indicates diabetes, 5.7% to 6.4% indicates prediabetes, and below 5.7% is normal.<br>* **Fasting plasma glucose (FPG) test:** This test measures your blood sugar level after an overnight fast of at least 8 hours. A level of 126 mg/dL or higher indicates diabetes, 100 to 125 mg/dL indicates prediabetes, and below 100 mg/dL is normal.<br>* **Oral glucose tolerance test (OGTT):** This test is not commonly used to diagnose prediabetes, but it can be used to diagnose diabetes. A level of 200 mg/dL or higher 2 hours after drinking a sugary liquid indicates diabetes, 140 to 199 mg/dL indicates prediabetes, and below 140 mg/dL is normal.<br><br>If you have any concerns about your blood sugar levels, it is important to see your doctor for testing. Early diagnosis and treatment of diabetes can help prevent serious health complications.<br><br>I hope this is helpful! Let me know if you have any other questions.
**4. Which tests help my doctor know what kind of diabetes I have?**	* The tests that can help your doctor know what kind of diabetes you have are:<br>* **Genetic testing:** This can diagnose most forms of monogenic diabetes.<br>* **Autoantibody testing:** This can help determine if you have type 1 diabetes.<br>* The type of diabetes you have will affect the treatment you need.<br>* If you have any questions about the tests that can help your doctor know what kind of diabetes you have, be sure to talk to your doctor.

	Here are some additional things to keep in mind:<br><br>* Genetic testing is not always available, and it can be expensive.<br>* Autoantibody testing is not always accurate, and it can sometimes give false positives or false negatives.<br><br>If you have any concerns about your diabetes, be sure to talk to your doctor. They can help you understand your test results and develop a treatment plan that is right for you.<br><br>I hope this is helpful!
**Topic: Managing Diabetes**	
**1. How can I manage my diabetes?**	* **Eat a healthy diet.** This means eating plenty of fruits, vegetables, and whole grains. It also means limiting processed foods, sugary drinks, and unhealthy fats.<br>* **Exercise regularly.** Aim for at least 30 minutes of moderate-intensity exercise most days of the week.<br>* **Take your medications as prescribed.** If you are taking insulin or other diabetes medications, it is important to take them as prescribed by your doctor.<br>* **Monitor your blood sugar levels.** This will help you track your progress and make sure that your diabetes is being managed effectively.<br>* **Manage stress.** Stress can raise blood sugar levels. Find healthy ways to manage stress, such as exercise, yoga, or meditation.<br>* **See your doctor regularly.** Your doctor will monitor your blood sugar levels, blood pressure, and cholesterol levels and make sure that your diabetes is being managed effectively.<br><br>These are just a few tips on how to manage diabetes. It is important to work with your doctor to create a personalized management plan that is right for you.<br><br>Here are some additional tips that you can follow:<br><br>* **Get enough sleep.** When you are sleep-deprived, your blood sugar levels can become more erratic. Aim for 7-8 hours of sleep per night.<br>* **Quit smoking.** Smoking can damage your blood vessels and make it harder to control your blood sugar levels.<br>* **Manage your weight.** If you are overweight or obese, losing even a small amount of weight can help improve your blood sugar control.

	* **Get vaccinated.** People with diabetes are at increased risk for certain infections, such as the flu and pneumonia. Make sure to get vaccinated against these infections.<br><br>By following these tips, you can take control of your diabetes and live a healthy life.
**2. What are the recommended targets for blood glucose levels?**	The American Diabetes Association (ADA) recommends the following blood glucose targets for adults with diabetes:<br><br>* **Before meals:** 80 to 130 mg/dL (4.4 to 7.2 mmol/L)<br>* **2 hours after meals:** Less than 180 mg/dL (10.0 mmol/L)<br><br>However, these are just general guidelines. The best target range for you will vary depending on your individual circumstances, such as your age, type of diabetes, and other health conditions. Your healthcare team can help you determine the best target range for you.<br><br>It is also important to remember that blood sugar levels can fluctuate throughout the day, so it is not unusual for them to go above or below your target range occasionally. However, if your blood sugar levels are consistently above or below your target range, it is important to talk to your healthcare team so they can help you make adjustments to your treatment plan.<br><br>Here are some tips for keeping your blood sugar levels in your target range:<br><br>* Eat regular meals and snacks throughout the day.<br>* Choose foods that are low in sugar and carbohydrates.<br>* Get regular exercise.<br>* Monitor your blood sugar levels regularly.<br>* Take your medications as prescribed by your doctor.<br><br>By following these tips, you can help keep your blood sugar levels in check and reduce your risk of developing diabetes complications.

	I hope this helps!
**3. What medicines might I take for diabetes?**	The medications you might take for diabetes depend on the type of diabetes you have.<br><br>* **Type 1 diabetes** is an autoimmune disease that causes the body to attack the cells in the pancreas that produce insulin. Insulin is a hormone that helps the body use glucose for energy. People with type 1 diabetes need to take insulin injections to survive.<br>* **Type 2 diabetes** is a chronic condition that affects the way the body metabolizes glucose. People with type 2 diabetes either do not produce enough insulin or their cells do not respond to insulin properly. There are a variety of medications available to treat type 2 diabetes, including:<br>* Metformin: This is the most commonly prescribed medication for type 2 diabetes. It helps the body use glucose for energy and also helps to reduce the amount of glucose produced by the liver.<br>* Sulfonylureas: These medications work by stimulating the pancreas to produce more insulin.<br>* DPP-4 inhibitors: These medications work by increasing the levels of incretin hormones, which help to regulate blood sugar levels.<br>* GLP-1 receptor agonists: These medications work by increasing insulin secretion and reducing appetite.<br>* SGLT2 inhibitors: These medications work by preventing the kidneys from reabsorbing glucose, which leads to increased glucose excretion in urine.<br><br>The best medication for you will depend on your individual needs and medical history. It is important to talk to your doctor about the different options available to you.<br><br>In addition to medications, there are other lifestyle changes that can help to manage diabetes. These include eating a healthy diet, exercising regularly, and losing weight if you are overweight or obese.<br><br>If you have diabetes, it is important to work with your doctor to develop a treatment plan that is right for you. This plan should include medications, lifestyle changes, and regular monitoring of your blood sugar levels. By working together, you can manage your diabetes and prevent complications.<br><br>I hope this answers your question. Let me know if you have any other questions.
**4. What are the different types of insulin?**	* **Rapid-acting insulin** (also known as ultra rapid-acting insulin) starts to work within 15 minutes, peaks at 1 hour, and lasts for 2 to 4 hours. It is used to control blood sugar levels after meals.

	* **Short-acting insulin** (also known as regular insulin) begins to work in 30 minutes, peaks between 2 to 3 hours, and lasts for 3 to 6 hours. It is also used to control blood sugar levels after meals.<br>* **Intermediate-acting insulin** takes 2 to 4 hours to start working, peaks between 4 to 12 hours, and remains effective for 12 to 18 hours. It is used to provide a long-lasting base of insulin throughout the day.<br>* **Long-acting insulin** has an onset of 2 hours, does not have a peak, and lasts for 24 hours. It is used to provide a long-lasting base of insulin throughout the day.<br><br>The type of insulin that is right for you will depend on your individual needs and lifestyle. Your doctor can help you determine the best type of insulin for you.<br><br>Here are some additional things to keep in mind about insulin:<br><br>* Insulin is injected under the skin.<br>* You can inject insulin with a needle and syringe, an insulin pen, or an insulin pump.<br>* The dosage of insulin will vary depending on your individual needs.<br>* You may need to adjust your insulin dosage depending on your diet, activity level, and blood sugar levels.<br>* It is important to monitor your blood sugar levels regularly to make sure that your insulin dosage is correct.<br><br>I hope this is helpful!
**5. What are the different ways to take insulin?**	* **Needle and syringe:** This is the most common way to take insulin. It is a simple and inexpensive method, but it can be difficult to get the correct dose.<br>* **Insulin pen:** An insulin pen is a device that looks like a pen and contains a cartridge of insulin. It is more accurate than a needle and syringe, and it is easier to use.<br>* **Insulin pump:** An insulin pump is a small device that delivers insulin continuously throughout the day. It is a good option for people who need to take insulin multiple times a day.<br>* **Insulin inhaler:** An insulin inhaler is a device that delivers insulin through the lungs. It is a newer way to take insulin, and it is not yet available for everyone.<br>* **Jet injector:** A jet injector is a device that delivers insulin through the skin using a high-pressure spray. It is another newer way to take insulin, and it is not yet available for everyone.

	* **Artificial pancreas:** An artificial pancreas is a system of devices that work together to automatically deliver insulin based on your blood sugar levels. It is a newer and more advanced way to manage diabetes, and it is not yet available for everyone.<br><br>The best way to decide which way to take insulin is to talk to your doctor. They can help you choose the method that is right for you based on your individual needs and preferences.<br><br>Here are some of the factors to consider when choosing a way to take insulin:<br><br>* Your lifestyle: If you are active and on the go, an insulin pump may be a good option for you. If you are more sedentary, a needle and syringe or an insulin pen may be a better choice.<br>* Your insurance plan: Some insurance plans may cover the cost of an insulin pump or an artificial pancreas, while others may not.<br>* Your preferences: Some people prefer to take insulin injections themselves, while others prefer to have someone else do it for them. Some people prefer to use a device that is easy to carry around, while others prefer a device that is less noticeable.<br><br>Ultimately, the best way to take insulin is the way that works best for you. There is no right or wrong answer, and what works for one person may not work for another. Talk to your doctor to learn more about the different ways to take insulin and to find the method that is right for you.
**6. What oral medicines treat type 2 diabetes?**	There are many oral medicines that can be used to treat type 2 diabetes. The most common are metformin, sulfonylureas, DPP-4 inhibitors, SGLT2 inhibitors, and GLP-1 agonists.<br><br>* **Metformin** is the first medication that most people with type 2 diabetes take. It helps your liver make less glucose and helps your body use insulin better.<br>* **Sulfonylureas** work by stimulating the pancreas to release more insulin.<br>* **DPP-4 inhibitors** work by blocking the breakdown of a hormone called glucagon-like peptide-1 (GLP-1). GLP-1 helps your pancreas release more insulin and helps your liver make less glucose.<br>* **SGLT2 inhibitors** work by blocking the action of a protein called sodium-glucose cotransporter 2 (SGLT2). SGLT2 is responsible for the reabsorption of glucose in the kidneys. By blocking SGLT2, SGLT2 inhibitors help the body get rid of more glucose through the urine.

	* **GLP-1 agonists** work by mimicking the effects of GLP-1. They help the pancreas release more insulin, help the liver make less glucose, and slow down the emptying of the stomach.<br><br>The best oral medication for you will depend on your individual circumstances, such as your age, other health conditions, and how well your blood sugar is controlled with other treatments. Your doctor will help you choose the right medication or combination of medications for you.<br><br>It is important to remember that oral medications are not a cure for type 2 diabetes. They are only one part of a comprehensive treatment plan that also includes healthy eating, regular exercise, and weight loss.<br><br>I hope this answers your question in 300 words. Let me know if you have any other questions.
**7. What should I know about side effects of diabetes medicines?**	* All medications have side effects, including diabetes medications.<br>* The most common side effects of diabetes medications are hypoglycemia (low blood sugar), gastrointestinal problems, and weight gain.<br>* Rare side effects of diabetes medications can also occur.<br>* It is important to talk to your doctor about the risks and benefits of any medication before you start taking it.<br>* If you experience any side effects from your diabetes medication, you should talk to your doctor.<br><br>Here are some additional tips for managing the side effects of diabetes medications:<br><br>* Take your medication as prescribed by your doctor.<br>* Eat regular meals and snacks.<br>* Exercise regularly.<br>* Monitor your blood sugar levels regularly.<br>* Talk to your doctor about any side effects that you are experiencing.<br><br>I hope this is helpful. Please let me know if you have any other questions.

**8. What foods can I eat if I have diabetes?**	People with diabetes can still enjoy a variety of foods, but it is important to make healthy choices. Here are some of the best foods to eat if you have diabetes:<br><br>* **Nonstarchy vegetables:** These are low in calories and carbohydrates and high in fiber, which can help to regulate blood sugar levels. Good examples include broccoli, carrots, greens, peppers, and tomatoes.<br>* **Fruits:** Fruits are a good source of vitamins, minerals, and fiber. Choose fruits that are lower in sugar, such as berries, apples, and bananas.<br>* **Whole grains:** Whole grains are a good source of fiber and complex carbohydrates, which can help to keep you feeling full. Good examples include brown rice, quinoa, and whole-wheat bread.<br>* **Lean protein:** Lean protein, such as chicken, fish, and beans, can help to keep you feeling full and satisfied. It is important to choose lean protein sources that are low in saturated fat.<br>* **Low-fat or fat-free dairy:** Low-fat or fat-free dairy products, such as milk, yogurt, and cheese, are a good source of calcium and protein. They are also relatively low in calories and carbohydrates.<br>* **Heart-healthy fats:** Heart-healthy fats, such as those found in olive oil, nuts, and seeds, can help to improve cholesterol levels and reduce the risk of heart disease.<br><br>Here are some of the foods that people with diabetes should limit or avoid:<br><br>* **Sugar-sweetened drinks:** Sugar-sweetened drinks, such as soda, juice, and sweetened tea, can raise blood sugar levels quickly. It is best to limit or avoid these drinks altogether.<br>* **Trans fats:** Trans fats are unhealthy fats that can raise cholesterol levels and increase the risk of heart disease. They are found in some processed foods, such as cookies, crackers, and fried foods.<br>* **Simple carbohydrates:** Simple carbohydrates, such as white bread, white rice, and pasta, are broken down quickly into glucose, which can raise blood sugar levels. It is best to choose complex carbohydrates, such as whole grains, instead.<br>* **High-fat foods:** High-fat foods, such as fried foods, fatty meats, and full-fat dairy products, can raise cholesterol levels and increase the risk of heart disease. It is best to choose lean protein sources and low-fat or fat-free dairy products.<br><br>It is important to work with your doctor or registered dietitian to create a meal plan that is right for you. This plan will take into account your individual needs and preferences, as well as your blood sugar goals.

	I hope this helps!
**9. What foods and drinks should I limit if I have diabetes?**	**Foods and drinks to limit if you have diabetes**<br><br>People with diabetes should limit their intake of foods and drinks that are high in saturated fat, trans fat, sodium, sugar, and alcohol. These foods can raise blood sugar levels and make it difficult to manage diabetes.<br><br>Some specific foods and drinks to limit include:<br><br>* Fried foods<br>* Full-fat dairy products<br>* Red meat<br>* Processed meats<br>* White bread and rice<br>* Sugary drinks, such as soda, juice, and sports drinks<br>* Alcohol<br><br>Here are some foods and drinks that people with diabetes can enjoy:<br><br>* Fruits and vegetables<br>* Whole grains<br>* Lean protein<br>* Low-fat or fat-free dairy products<br>* Water

	It is important to talk to your doctor or a registered dietitian about creating a meal plan that is right for you. They can help you make sure that you are getting the nutrients you need while also managing your blood sugar levels.<br><br>Here are some additional tips for managing diabetes through diet:<br><br>* Eat regular meals and snacks throughout the day.<br>* Choose foods that are low on the glycemic index.<br>* Limit your portion sizes.<br>* Cook at home more often and avoid processed foods.<br>* Read food labels carefully and choose foods that are low in saturated fat, trans fat, sodium, and sugar.<br>* Drink plenty of water.<br><br>By following these tips, you can help manage your diabetes and improve your overall health.
**10. When should I eat if I have diabetes?**	The best time to eat if you have diabetes depends on your individual needs, your diabetes medications, and your lifestyle. However, there are some general guidelines that can help you.<br><br>* **Eat regular meals and snacks throughout the day.** This will help to keep your blood sugar levels stable. Aim to eat every 3-4 hours.<br>* **Choose foods that are low in carbohydrates and high in protein and fiber.** This will help to slow down the absorption of glucose into your bloodstream.<br>* **Avoid sugary drinks and foods.** These foods can cause your blood sugar levels to spike.<br>* **Be aware of your activity level and adjust your meal and snack timing accordingly.** If you are going to be exercising, eat a snack or meal beforehand to prevent your blood sugar levels from dropping too low.<br>* **Talk to your doctor or a registered dietitian about the best meal and snack timing for you.** They can help you create a personalized plan that meets your individual needs.<br><br>Here are some specific examples of meals and snacks that are good for people with diabetes:

	* **Breakfast:** Oatmeal with fruit and nuts, eggs with whole-wheat toast, or yogurt with berries.<br>* **Lunch:** Salad with grilled chicken or fish, a turkey sandwich on whole-wheat bread, or soup and a sandwich.<br>* **Dinner:** Grilled salmon with roasted vegetables, chicken stir-fry, or lentil soup.<br>* **Snacks:** Fruits, vegetables, nuts, seeds, or yogurt.<br><br>It is important to note that these are just examples, and the best meal and snack timing for you will vary depending on your individual needs. Talk to your doctor or a registered dietitian to create a personalized plan that meets your needs.<br><br>I hope this helps!
**11. How much can I eat if I have diabetes?**	How much can I eat if I have diabetes?<br><br>The amount of food you can eat if you have diabetes depends on a number of factors, including your age, sex, weight, activity level, and type of diabetes medication you are taking. It is important to work with your doctor or a registered dietitian to create a personalized meal plan that meets your individual needs.<br><br>Here are some general tips for eating healthy with diabetes:<br><br>Eat plenty of non-starchy vegetables, such as leafy greens, broccoli, and cauliflower.<br>Choose lean protein sources, such as fish, chicken, beans, and tofu.<br>Limit processed foods, sugary drinks, and unhealthy fats.<br>Eat regular meals and snacks throughout the day.<br>If you are taking insulin, you may need to count carbohydrates to help you manage your blood glucose levels. Carbohydrates turn into glucose in your body, so it is important to limit your intake of carbohydrates with added sugars or those with refined grains. Instead, choose carbohydrates from fruit, vegetables, whole grains, beans, and low-fat or nonfat milk.<br><br>Here are some tips for estimating portion sizes:

	1 serving of meat or poultry is the size of your palm or a deck of cards.<br>1 3-ounce serving of fish is the size of a checkbook.<br>1 serving of cheese is six dice.<br>1/2 cup of cooked rice or pasta is the size of a rounded handful or a tennis ball.<br>1 serving of a pancake or waffle is the size of a DVD.<br>2 tablespoons of peanut butter is the size of a ping-pong ball.<br>If you are unsure how much to eat, it is always best to err on the side of caution and eat less. You can always eat more later if you are still hungry.
**12. Will supplements and vitamins help my diabetes?**	There is no clear evidence that taking supplements and vitamins will help manage diabetes. However, some vitamins and minerals are important for people with diabetes, such as vitamin D, vitamin B12, magnesium, chromium, and zinc. If you are deficient in these vitamins, your doctor may recommend that you take a supplement.<br><br>It is important to talk to your doctor before taking any supplements, as some of them can interact with diabetes medications or other medications you are taking. Your doctor can also help you determine if you are getting enough vitamins and minerals from your diet.<br><br>The best way to manage diabetes is to eat a healthy diet, exercise regularly, and keep your blood sugar levels under control.<br><br>Here are some general tips for managing diabetes:<br><br>* Eat a healthy diet. This means eating plenty of fruits, vegetables, and whole grains.<br>* Exercise regularly. Aim for at least 30 minutes of moderate-intensity exercise most days of the week.<br>* Keep your blood sugar levels under control. This may involve taking medication or insulin.<br>* Monitor your blood sugar levels regularly. This will help you see how your diabetes is managed and make adjustments as needed.<br><br>By following these tips, you can help manage your diabetes and live a healthy life.<br><br>I hope this answers your question. Let me know if you have any other questions.

**13. Why should I be physically active if I have diabetes?**	Physical activity is an important part of managing diabetes. It helps to lower blood sugar levels, improve blood pressure, and reduce the risk of heart disease, stroke, and other complications.<br><br>Even small amounts of physical activity can be beneficial. The Centers for Disease Control and Prevention (CDC) recommends that adults with diabetes get at least 30 minutes of moderate-intensity aerobic activity most days of the week. Moderate-intensity activities include brisk walking, swimming, and bicycling.<br><br>If you are new to exercise, start slowly and gradually increase the amount of time you spend being active. Talk to your doctor before starting any new exercise program, especially if you have any health conditions.<br><br>Here are some tips for being physically active if you have diabetes:<br><br>* Choose activities that you enjoy and that are appropriate for your fitness level.<br>* Warm up before you exercise and cool down afterwards.<br>* Drink plenty of fluids before, during, and after exercise.<br>* Monitor your blood sugar levels before, during, and after exercise.<br><br>If you have any questions or concerns about physical activity, talk to your doctor.<br><br>Here are some additional benefits of physical activity for people with diabetes:<br><br>* Helps to control weight.<br>* Improves mood and sleep.<br>* Reduces the risk of falls.<br>* Improves cognitive function.<br>* Improves quality of life.

	Physical activity is an important part of a healthy lifestyle for everyone, but it is especially important for people with diabetes. By being physically active, you can help to manage your diabetes and improve your overall health.
**14. How can I be physically active safely if I have diabetes?**	**How to be physically active safely with diabetes**<br><br>People with diabetes can and should be physically active. However, it is important to take precautions to prevent low blood sugar (hypoglycemia) and other complications.<br><br>**Here are some tips for safe physical activity with diabetes:**<br><br>* Talk to your doctor before starting a new exercise program. They can help you create a plan that is safe and appropriate for your individual needs.<br>* Start slowly and gradually increase the amount of time and intensity of your activity.<br>* Check your blood sugar before, during, and after exercise.<br>* Eat a snack or drink juice before, during, or after exercise to prevent hypoglycemia.<br>* Wear comfortable, supportive shoes.<br>* Take care of your feet by checking them regularly for cuts, blisters, or sores.<br>* Stay hydrated by drinking plenty of fluids before, during, and after exercise.<br>* Avoid exercising in extreme heat or cold.<br>* Stop exercising if you feel faint, dizzy, or have chest pain.<br><br>By following these tips, you can safely enjoy the many benefits of physical activity, even if you have diabetes.<br><br>Here are some additional tips that are not included in the 300-word limit:<br><br>* Choose activities that you enjoy and that are appropriate for your fitness level.<br>* Find a workout buddy to help you stay motivated.<br>* Set realistic goals and gradually increase your activity level over time.

	* Listen to your body and take breaks when you need them.<br>* Don't be afraid to ask for help from your doctor or a certified diabetes educator.<br><br>With a little planning and care, you can safely and enjoyably participate in physical activity even if you have diabetes.
**15. What physical activities should I do if I have diabetes?**	As a public health expert specializing in diabetes, I would recommend the following physical activities for people with diabetes:<br><br>* **Aerobic exercise:** Aerobic exercise is any activity that gets your heart rate up and makes you breathe harder. It is important to do aerobic exercise for at least 30 minutes a day, most days of the week. Some examples of aerobic exercise include walking, running, swimming, biking, dancing, and playing sports.<br>* **Strength training:** Strength training is important for building muscle and bone mass. It can also help improve blood sugar control. Strength training should be done two to three times a week. Some examples of strength training exercises include lifting weights, using resistance bands, or doing bodyweight exercises.<br>* **Stretching:** Stretching can help improve flexibility and range of motion. It can also help prevent injuries. Stretching should be done after aerobic exercise or strength training. Some examples of stretching exercises include hamstring stretches, calf stretches, and shoulder stretches.<br><br>It is important to talk to your doctor before starting any new exercise program. They can help you determine which activities are safe for you and help you create a plan that is right for you.<br><br>Here are some additional tips for people with diabetes who are exercising:<br><br>* Start slowly and gradually increase the amount of time and intensity of your exercise.<br>* Drink plenty of fluids before, during, and after exercise.<br>* Eat a snack or meal before exercising if your blood sugar is low.<br>* Monitor your blood sugar before, during, and after exercise.<br>* Stop exercising if you feel pain or dizziness.<br><br>By following these tips, you can safely and effectively incorporate physical activity into your life and improve your overall health and well-being.
**Topic: Preventing Diabetes Problems**	

**1. How can I lower my chances of developing type 2 diabetes?**	Sure, I can help you with that.<br><br>**How to lower your chances of developing type 2 diabetes**<br><br>Type 2 diabetes is a chronic condition that affects how your body turns food into energy. It is the most common type of diabetes, accounting for 90 to 95% of all diabetes cases.<br><br>There is no cure for type 2 diabetes, but it can be managed with medication, diet, and exercise. The good news is that you can lower your chances of developing type 2 diabetes by making lifestyle changes.<br><br>Here are some things you can do to lower your chances of developing type 2 diabetes:<br><br>* **Lose weight and keep it off.** If you are overweight or obese, losing even a small amount of weight can help reduce your risk of developing type 2 diabetes. Aim to lose 5 to 7% of your body weight.<br>* **Get regular exercise.** Aim for at least 30 minutes of moderate-intensity exercise most days of the week.<br>* **Eat a healthy diet.** This means eating plenty of fruits, vegetables, and whole grains. Choose lean protein sources and limit unhealthy fats, sugary drinks, and processed foods.<br>* **Don't smoke.** Smoking damages your blood vessels and makes it harder for your body to use insulin. If you smoke, quitting is the best thing you can do for your health.<br>* **Manage other health conditions.** If you have high blood pressure, high cholesterol, or prediabetes, managing these conditions can also help reduce your risk of developing type 2 diabetes.<br><br>If you are concerned about your risk of developing type 2 diabetes, talk to your doctor. They can help you assess your risk and develop a plan to lower it.<br><br>Here are some additional tips that may help you prevent type 2 diabetes:

	* Eat breakfast every day. Skipping breakfast can make you more likely to overeat later in the day.<br>* Limit sugary drinks. Sugary drinks are a major source of added sugar in the American diet. Too much added sugar can increase your risk of developing type 2 diabetes.<br>* Get enough sleep. When you don't get enough sleep, your body produces more of the stress hormone cortisol. Cortisol can make it harder for your body to use insulin.<br>* Manage stress. Stress can also make it harder for your body to use insulin. Find healthy ways to manage stress, such as exercise, yoga, or meditation.<br><br>By making these lifestyle changes, you can lower your chances of developing type 2 diabetes and improve your overall health.<br><br>I hope this helps. Let me know if you have any other questions.<br><br>Here are some additional resources that you may find helpful:<br><br>* Diabetes Prevention Program: https://www.cdc.gov/diabetes/prevention/<br>* How to Prevent Diabetes: https://medlineplus.gov/howtopreventdiabetes.html<br>* American Diabetes Association: https://www.diabetes.org/
**2. What should I do if my health care professional told me I have prediabetes?**	Hi, I am a public health expert specializing in diabetes. Here are some things you can do if your healthcare professional told you that you have prediabetes:<br><br>* **Lose weight.** Losing even 5 to 7% of your body weight can help lower your blood sugar levels and reduce your risk of developing type 2 diabetes.<br>* **Get regular exercise.** Aim for at least 150 minutes of moderate-intensity aerobic activity or 75 minutes of vigorous-intensity aerobic activity each week.<br>* **Eat a healthy diet.** Choose foods that are low in calories, fat, and sugar, and high in fiber. This includes fruits, vegetables, whole grains, lean protein, and healthy fats.<br>* **Quit smoking.** Smoking can damage your blood vessels and make it harder for your body to use insulin. If you smoke, quitting is one of the best things you can do for your health.<br>* **Manage stress.** Stress can raise your blood sugar levels. Find healthy ways to manage stress, such as exercise, relaxation techniques, or spending time with loved ones.

	If you are struggling to make these lifestyle changes on your own, there are resources available to help you. Your healthcare provider can refer you to a dietitian or a diabetes educator who can help you create a personalized plan. You can also join a diabetes support group or find online resources.<br><br>It is important to remember that prediabetes is not a death sentence. By making lifestyle changes, you can lower your risk of developing type 2 diabetes and live a long and healthy life.<br><br>Here are some additional tips for people with prediabetes:<br><br>* Get regular checkups with your healthcare provider.<br>* Monitor your blood sugar levels at home.<br>* Take medication as prescribed by your healthcare provider.<br>* Be aware of the symptoms of type 2 diabetes and seek medical attention if you experience any of them.<br><br>I hope this information is helpful. Please let me know if you have any other questions.
**3. If I had gestational diabetes when I was pregnant, how can I lower my chances of developing type 2 diabetes?**	**How to lower your chances of developing type 2 diabetes after gestational diabetes**<br><br>Gestational diabetes is a type of diabetes that develops during pregnancy. Most of the time, gestational diabetes goes away after your baby is born. However, you still have a greater chance of developing type 2 diabetes within 5 to 10 years.<br><br>Here are some things you can do to lower your chances of developing type 2 diabetes after gestational diabetes:<br><br>* **Get to and stay at a healthy weight.** This is the most important thing you can do to reduce your risk of type 2 diabetes. If you are overweight or obese, losing even a small amount of weight can make a big difference.<br>* **Eat a healthy diet.** This means eating plenty of fruits, vegetables, and whole grains, and limiting processed foods, sugary drinks, and unhealthy fats.<br>* **Get regular exercise.** Aim for at least 30 minutes of moderate-intensity exercise most days of the week.<br>* **Take part in a diabetes prevention program.** These programs can help you make lifestyle changes to lower your risk of type 2 diabetes.

	* **Talk to your doctor about medications.** If you are at high risk of developing type 2 diabetes, your doctor may prescribe medication to help prevent it.<br><br>It is also important to be aware of the signs and symptoms of type 2 diabetes, so that you can get treatment early if you do develop it. These signs and symptoms include:<br><br>* Increased thirst<br>* Frequent urination<br>* Extreme hunger<br>* Fatigue<br>* Blurred vision<br>* Slow-healing sores<br><br>If you have any of these symptoms, see your doctor right away.<br><br>By making healthy lifestyle choices, you can lower your chances of developing type 2 diabetes and improve your overall health.<br><br>Here are some additional things to keep in mind:<br><br>* The risk of developing type 2 diabetes after gestational diabetes increases with age.<br>* If you have other risk factors for type 2 diabetes, such as family history, race/ethnicity, or high blood pressure, your risk is even higher.<br>* Even if you do develop type 2 diabetes, there are things you can do to manage it and prevent complications.<br><br>I hope this information is helpful. Please let me know if you have any other questions.
**4. What health problems can people with diabetes develop?**	People with diabetes are at an increased risk of developing a number of health problems, including heart disease, stroke, eye problems, kidney disease, nerve damage, gum disease, skin problems, and mental health problems. These health problems can be caused by high blood sugar levels, which can damage the blood vessels, nerves, and other organs over time.

	Here are some of the most common health problems that people with diabetes can develop:<br><br>* **Heart disease and stroke:** Diabetes is a major risk factor for heart disease and stroke. People with diabetes are twice as likely to have a heart attack or stroke as people without diabetes.<br>* **Eye problems:** Diabetes can damage the blood vessels in the retina, the light-sensitive tissue at the back of the eye. This can lead to diabetic retinopathy, which can cause blindness.<br>* **Kidney disease:** Diabetes can damage the kidneys, leading to kidney failure.<br>* **Nerve damage:** Diabetes can damage the nerves, leading to numbness, tingling, and pain in the hands and feet. In some cases, nerve damage can lead to foot ulcers and amputation.<br><br>These are just some of the health problems that people with diabetes can develop. By taking steps to control their blood sugar levels, people with diabetes can reduce their risk of developing these complications.<br><br>Here are some tips for controlling blood sugar levels:<br><br>* Eat a healthy diet.<br>* Exercise regularly.<br>* Take medication as prescribed by your doctor.<br>* Monitor your blood sugar levels regularly.<br><br>If you have diabetes, it is important to work closely with your doctor to manage your condition and prevent complications.<br><br>I hope this answers your question. Let me know if you have any other questions.

**5. How can I lower my chances of a heart attack or stroke if I have diabetes?**	People with diabetes are at a higher risk of heart attack and stroke than people without diabetes. This is because high blood sugar levels can damage the blood vessels, making them more likely to clog or rupture.<br><br>Here are some things you can do to lower your chances of having a heart attack or stroke if you have diabetes:<br><br>* **Manage your diabetes ABCs:** This includes keeping your blood glucose, blood pressure, and cholesterol levels in the healthy range.<br>* **A1C:** The A1C test shows your average blood glucose level over the past 3 months. The goal for most people with diabetes is below 7%.<br>* **Blood pressure:** The goal for most people with diabetes is below 140/90 mm Hg.<br>* **Cholesterol:** Your cholesterol numbers should be discussed with your doctor. If you are over 40 years old, you may need to take medicine to lower your cholesterol levels.<br>* **Quit smoking:** Smoking narrows the blood vessels, making it harder for your heart to work.<br>* **Get regular exercise:** Exercise helps to control blood sugar levels, blood pressure, and cholesterol levels. Aim for at least 30 minutes of moderate-intensity exercise most days of the week.<br>* **Maintain a healthy weight:** Being overweight or obese is a risk factor for heart disease and stroke. Aim to maintain a healthy weight by eating a healthy diet and exercising regularly.<br>* **Eat a healthy diet:** A healthy diet is low in saturated fat, trans fat, and cholesterol. It is also high in fruits, vegetables, and whole grains.<br>* **Get enough sleep:** Getting enough sleep helps to regulate blood sugar levels. Aim for 7-8 hours of sleep per night.<br>* **Manage stress:** Stress can raise blood sugar levels. Find healthy ways to manage stress, such as exercise, yoga, or meditation.<br>* **Take your medications as prescribed:** Your doctor may prescribe medications to help you control your blood sugar, blood pressure, and cholesterol levels. Take your medications as prescribed and talk to your doctor if you have any questions or concerns.<br><br>By following these steps, you can lower your chances of having a heart attack or stroke and live a long and healthy life.<br><br>I hope this helps!
**6. What are the warning signs of heart attack and stroke?**	**What are the warning signs of heart attack and stroke?**

	Heart attack and stroke are two of the leading causes of death in the world. They are both caused by a sudden decrease in blood flow to the heart or brain. The warning signs of a heart attack can vary from person to person, but the most common symptoms include:<br><br>* Chest pain or discomfort that may feel like pressure, tightness, squeezing, or aching.<br>* Pain or discomfort in one or both arms, the back, neck, jaw, or stomach.<br>* Shortness of breath.<br>* Nausea or vomiting.<br>* Lightheadedness or sudden dizziness.<br><br>The warning signs of a stroke can also vary, but the most common symptoms include:<br><br>* Sudden numbness or weakness of the face, arm, or leg, especially on one side of the body.<br>* Sudden confusion, trouble speaking or understanding.<br>* Sudden trouble seeing in one or both eyes.<br>* Sudden trouble walking, dizziness, or loss of balance or coordination.<br>* Sudden severe headache.<br><br>If you experience any of these symptoms, it is important to seek medical attention immediately. Time is of the essence, and early treatment can save your life.<br><br>Here are some additional things to keep in mind about the warning signs of heart attack and stroke:<br><br>* Women are more likely to experience symptoms such as shortness of breath, nausea, and vomiting than men.<br>* People with diabetes are at increased risk of heart attack and stroke.<br>* If you have any risk factors for heart attack or stroke, such as high blood pressure, high cholesterol, or smoking, it is important to be aware of the warning signs and to see your doctor regularly.

	By knowing the warning signs of heart attack and stroke, you can take action quickly and save a life.<br><br>Here are some mnemonic devices that can help you remember the warning signs of heart attack and stroke:<br><br>* **F.A.S.T.** for stroke: Face drooping, Arm weakness, Speech difficulty, Time to call 911.<br>* **S.T.A.R.T.** for heart attack: Sudden chest pain, Trouble breathing, Arm pain, Rapid or irregular heartbeat, Trouble talking.<br><br>If you think you or someone you know is having a heart attack or stroke, it is important to act quickly. Call 911 immediately. Do not wait to see if the symptoms go away. Early treatment is essential for saving lives.
**7. What causes low blood glucose in people with diabetes?**	Sure, here is the answer to your question:<br><br>* * *<br><br>Low blood glucose, also known as hypoglycemia, is a condition in which the blood sugar level drops too low. This can be a serious problem, especially for people with diabetes.<br><br>There are many causes of low blood sugar in people with diabetes, including:<br><br>* Taking too much insulin or other diabetes medication.<br>* Not eating enough food or skipping a meal.<br>* Exercising more than usual without eating more food.<br>* Drinking alcohol.<br>* Certain medical conditions, such as liver or kidney disease.<br>* Certain medications, such as steroids or some antibiotics.<br><br>The symptoms of low blood sugar can vary from person to person, but they may include:

	* Shakiness.<br>* Sweating.<br>* Hunger.<br>* Dizziness.<br>* Headache.<br>* Confusion.<br>* Slurred speech.<br>* Numbness or tingling around the lips or tongue.<br>* Blurred vision.<br>* Convulsions.<br>* Loss of consciousness.<br><br>If you experience any of these symptoms, it is important to treat low blood sugar immediately. The most common way to treat low blood sugar is to eat or drink something that contains glucose, such as candy, juice, or glucose tablets. You should also check your blood sugar level to make sure it has come back up to a safe level.<br><br>The causes of low blood sugar in people with diabetes can be complex, and it is important to work with your doctor to understand your individual risk factors. However, some of the most common causes include:<br><br>* Taking too much insulin or other diabetes medication. This is the most common cause of low blood sugar in people with diabetes. Insulin is a hormone that helps the body use glucose for energy. If you take too much insulin, it can cause your blood sugar level to drop too low.<br>* Not eating enough food or skipping a meal. When you don't eat enough food, your body doesn't have enough glucose to use for energy. This can cause your blood sugar level to drop.<br>* Exercising more than usual without eating more food. Exercise can help lower blood sugar levels, but it is important to eat enough food before, during, and after exercise to prevent low blood sugar.

	* Drinking alcohol. Alcohol can interfere with the body's ability to use glucose for energy. This can cause blood sugar levels to drop, especially if you haven't eaten in a while.<br>* Certain medical conditions. Certain medical conditions, such as liver or kidney disease, can make it more difficult for the body to regulate blood sugar levels. This can increase the risk of low blood sugar.<br>* Certain medications. Certain medications, such as steroids or some antibiotics, can also increase the risk of low blood sugar.<br><br>If you have diabetes, it is important to be aware of the causes of low blood sugar and to take steps to prevent it. You should work with your doctor to develop a treatment plan that is right for you. Here are some tips to help prevent low blood sugar:<br><br>* Know your blood sugar goals and how to adjust your insulin or medication accordingly.<br>* Eat regular meals and snacks.<br>* Carry a source of glucose with you at all times.<br>* Test your blood sugar regularly.<br>* Talk to your doctor about any medications that you are taking that could cause low blood sugar.<br><br>By following these tips, you can help to prevent low blood sugar and keep yourself safe.<br><br>* * *<br><br>I hope this answers your question. Please let me know if you have any other questions.
**8. How can I prevent low blood glucose if I have diabetes?**	Sure, here is the answer to your question:<br><br>**How can I prevent low blood glucose if I have diabetes?**<br><br>Low blood glucose, also known as hypoglycemia, is a common complication of diabetes. It can happen when your blood sugar level drops too low. This can be dangerous, and it's important to know how to prevent it.

	Here are some tips on how to prevent low blood sugar:<br><br>* **Eat regular meals and snacks.** This will help keep your blood sugar levels stable. Aim to eat meals and snacks every 3-4 hours.<br>* **Don't skip meals, even if you're not hungry.** Skipping meals can lead to low blood sugar later on.<br>* **Check your blood sugar regularly.** This will help you identify trends and make sure your levels are staying within your target range. Your doctor can help you determine how often you need to check your blood sugar.<br>* **Adjust your insulin or other diabetes medications as needed.** Your doctor can help you determine the right dose for you.<br>* **Be aware of the signs and symptoms of low blood sugar.** These can include sweating, shakiness, dizziness, and confusion. If you experience any of these symptoms, eat or drink something sugary right away.<br>* **Treat low blood sugar promptly.** If you think you have low blood sugar, eat or drink something sugary right away. A good choice is 15 grams of carbohydrates, such as glucose tablets, juice, or hard candy. Wait 15 minutes and check your blood sugar again. If your blood sugar is still low, eat or drink another 15 grams of carbohydrates.<br><br>Here are some additional tips that may help you prevent low blood sugar:<br><br>* **Avoid alcohol.** Alcohol can interfere with your body's ability to use glucose for energy.<br>* **Be careful when exercising.** Exercise can lower your blood sugar, so it's important to eat a snack or adjust your insulin dose before you start.<br>* **Talk to your doctor about other ways to prevent low blood sugar.** There are many other factors that can contribute to low blood sugar, so your doctor can help you develop a personalized plan to prevent it.<br><br>I hope these tips help you prevent low blood sugar. If you have any questions or concerns, please be sure to talk to your doctor.<br><br>In addition to the tips mentioned above, here are some additional things you can do to prevent low blood sugar:<br><br>* **Learn how to use a blood glucose meter.** This will help you track your blood sugar levels and identify trends.

	* **Talk to your doctor about your target blood sugar range.** This will vary depending on your individual needs.<br>* **Work with your doctor to develop a diabetes management plan.** This plan should include meal planning, exercise, and medication management.<br>* **Be prepared for low blood sugar.** Carry a source of fast-acting carbohydrates with you at all times.<br>* **Educate your family and friends about diabetes.** This will help them know how to help you if you experience low blood sugar.<br><br>By following these tips, you can help prevent low blood sugar and keep your diabetes under control.
**9. How can I keep my kidneys healthy if I have diabetes?**	I understand. I can help you with that.<br><br>As a public health expert specializing in diabetes, I know that kidney disease is a common complication of diabetes. It happens when high blood sugar levels damage the kidneys over time. There are many things people with diabetes can do to keep their kidneys healthy, including:<br><br>* **Keeping blood sugar levels under control.** This is the most important thing you can do to prevent kidney damage. Work with your doctor to create a treatment plan that includes diet, exercise, and medication. Your blood sugar levels should be as close to normal as possible.<br>* **Controlling blood pressure.** High blood pressure can also damage the kidneys. Make sure to get your blood pressure checked regularly and take medication as prescribed by your doctor. Your blood pressure should be below 140/90 mmHg.<br>* **Eating a healthy diet.** A healthy diet for people with diabetes is low in saturated fat, trans fat, cholesterol, and sodium. It should also be high in fruits, vegetables, and whole grains. These foods help to keep your blood sugar levels under control and protect your kidneys.<br>* **Losing weight if you are overweight or obese.** Excess weight can put extra strain on your kidneys. Aim to lose 5 to 10% of your body weight.<br>* **Quitting smoking.** Smoking damages the kidneys and makes it harder to control blood sugar levels. If you smoke, quitting is the best thing you can do for your health.<br>* **Drinking plenty of fluids.** This helps to keep your kidneys healthy and functioning properly. Aim to drink 8 to 10 glasses of water each day.<br><br>If you have diabetes, it is important to see your doctor regularly for checkups and tests. This will help to detect kidney damage early on, when it is easier to treat.<br><br>Here are some additional tips for keeping your kidneys healthy if you have diabetes:

	* Get regular exercise. Aim for at least 30 minutes of moderate-intensity exercise most days of the week. Exercise helps to control blood sugar levels, blood pressure, and weight.<br>* Manage stress. Stress can raise blood sugar levels and make it harder to control your diabetes. Find healthy ways to manage stress, such as yoga, meditation, or spending time in nature.<br>* Get enough sleep. When you don't get enough sleep, your body produces more of the stress hormone cortisol, which can raise blood sugar levels. Aim for 7 to 8 hours of sleep each night.<br><br>By following these tips, you can help to keep your kidneys healthy and prevent kidney disease.<br><br>I hope this answers your question. Please let me know if you have any other questions.
**10. What can I do to keep my feet healthy?**	* **Check your feet daily.** Look for any cuts, sores, redness, swelling, or changes in the skin or nails. Use a mirror to see the bottom of your feet if you can't see them yourself.<br>* **Wash your feet daily with warm (not hot) water and mild soap.** Dry your feet thoroughly, especially between the toes.<br>* **Smooth out corns and calluses gently.** If you have corns or calluses, talk with your foot doctor about the best way to care for these foot problems. If you have nerve damage, these patches can become ulcers.<br>* **Always trim your toenails straight across.** Do not cut them too short or into the corners.<br>* **Make it a habit to wear shoes and socks at all times.** Shoes should be comfortable and not too tight. Socks should be made of breathable material and should not have seams that can rub against your feet.<br>* **Shield your feet from extreme temperatures, both hot and cold.** Wear shoes or sandals when you are outside, even in hot weather. In the winter, wear warm socks and shoes to keep your feet warm.<br>* **It's essential to maintain good blood circulation to your feet.** Put your feet up when you are sitting. Wiggle your toes for a few minutes throughout the day. Move your ankles up and down and in and out to help blood flow in your feet and legs.<br>* **Schedule a foot examination during every health care visit.** This is especially important if you have any foot problems.<br><br>Here are some additional things you can do to keep your feet healthy:

	* **Control your blood sugar levels.** High blood sugar levels can damage nerves and blood vessels in your feet, which can lead to foot problems.<br>* **Quit smoking.** Smoking can damage blood vessels and make it harder for your feet to heal.<br>* **Maintain a healthy weight.** Being overweight or obese can put extra stress on your feet.<br>* **Get regular exercise.** Exercise can help improve blood circulation and keep your weight under control.<br><br>By following these tips, you can help keep your feet healthy and prevent foot problems.<br><br>I hope this helps!
**11. When should I see my health care provider about foot problems?**	As a public health expert specializing in diabetes, I am often asked when people with diabetes should see their healthcare provider about foot problems. Here are some common signs and symptoms that warrant a visit to the doctor:<br><br>* A cut, blister, or bruise on your foot that does not start to heal after a few days.<br>* Skin on your foot that becomes red, warm, or painful.<br>* A callus with dried blood inside of it.<br>* A foot infection that becomes black and smelly.<br>* Numbness or tingling in your feet.<br>* Painful or burning feet.<br>* Swelling in your feet or ankles.<br>* Changes in the color or texture of your toenails.<br><br>If you experience any of these symptoms, it is important to see your doctor right away. Diabetes can damage the nerves and blood vessels in your feet, making it more difficult for them to heal. This can lead to serious infections, which can sometimes require amputation.<br><br>In addition to the symptoms listed above, there are some other factors that may increase your risk of foot problems if you have diabetes. These include:<br><br>* High blood sugar levels

	* Smoking<br>* Poor circulation<br>* Obesity<br>* Kidney disease<br>* Peripheral neuropathy (nerve damage)<br><br>If you have any of these risk factors, it is especially important to be vigilant about checking your feet for any signs of problems. You should also see your doctor for regular foot exams.<br><br>By taking steps to prevent foot problems, you can help protect your feet and avoid serious complications.<br><br>Here are some tips for taking care of your feet if you have diabetes:<br><br>* Wash your feet daily with warm, soapy water.<br>* Dry your feet thoroughly, especially between the toes.<br>* Apply a moisturizer to your feet to keep them from drying out.<br>* Trim your toenails straight across and file them smooth.<br>* Wear shoes that fit well and provide good support.<br>* Avoid walking barefoot.<br>* Check your feet for any cuts, blisters, or sores every day.<br><br>By following these tips, you can help keep your feet healthy and prevent problems.
**12. How common is diabetic eye disease?**	Sure, I can help you with that.<br><br>Diabetic eye disease is a group of eye problems that can affect people with diabetes. The most common type of diabetic eye disease is diabetic retinopathy, which is damage to the blood vessels in the retina.

	As you mentioned, about one in three people with diabetes who are older than age 40 already have some signs of diabetic retinopathy. Over time, more than half of people with diabetes will develop diabetic retinopathy.<br><br>Diabetic eye disease is the leading cause of vision loss among working-age adults in the United States. However, early detection and treatment can help prevent vision loss.<br><br>Here are some of the other types of diabetic eye disease:<br><br>* Diabetic macular edema (DME): This is a condition where fluid leaks into the macula, which is the part of the retina responsible for sharp central vision. DME can cause blurry vision or blind spots.<br>* Diabetic glaucoma: This is a type of glaucoma that is caused by damage to the optic nerve. Glaucoma can cause vision loss, but it can be treated with medication or surgery.<br>* Cataracts: Cataracts are cloudy areas in the lens of the eye. They can make it difficult to see clearly. Cataracts are more common in people with diabetes, but they can be treated with surgery.<br><br>If you have diabetes, it is important to get regular eye exams. Your eye doctor can check for signs of diabetic eye disease and start treatment early if necessary.<br><br>Here are some things you can do to help prevent diabetic eye disease:<br><br>* Control your blood sugar levels.<br>* Keep your blood pressure and cholesterol levels in check.<br>* Don't smoke.<br>* Eat a healthy diet.<br>* Get regular exercise.<br>* See your eye doctor for regular eye exams.

	If you have any questions or concerns about diabetic eye disease, please talk to your doctor.<br><br>I hope this answers your question. Let me know if you have other questions.
**13. Who is more likely to develop diabetic eye disease?**	**Who is more likely to develop diabetic eye disease?**<br><br>Anyone with diabetes can develop diabetic eye disease, but your risk is greater if you have:<br><br>* **High blood sugar levels:** This is the most important risk factor for diabetic eye disease. The higher your blood sugar levels, the greater your risk of developing diabetic eye disease.<br>* **High blood pressure:** High blood pressure can also damage the blood vessels in your eyes, increasing your risk of diabetic eye disease.<br>* **Smoking:** Smoking can damage the blood vessels in your eyes, increasing your risk of diabetic eye disease.<br>* **Race/ethnicity:** African Americans, Hispanic/Latinos, and American Indians/Alaska Natives are more likely to develop diabetic eye disease than Caucasians.<br>* **Older age:** Your chances of developing diabetic eye disease increase the longer you have diabetes.<br><br>If you have diabetes, it is important to have regular eye exams to check for diabetic eye disease. Early detection and treatment can help prevent vision loss.<br><br>Here are some tips to help you reduce your risk of developing diabetic eye disease:<br><br>* **Get your blood sugar levels under control.** This is the most important thing you can do to protect your vision.<br>* **Control your blood pressure and cholesterol levels.**<br>* **Quit smoking.**<br>* **Have regular eye exams.**<br><br>By following these tips, you can help reduce your risk of developing diabetic eye disease and protect your vision.

**14. When should I see my health care provider about eye problems?**	Sure, here is the answer to your question:<br><br>**When should I see my health care provider about eye problems?**<br><br>If you have diabetes, it is important to see your eye doctor for regular eye exams, even if you have no symptoms. The American Diabetes Association (ADA) recommends that people with diabetes have their first eye exam within 5 years of diagnosis. If you have type 2 diabetes, you should have your first eye exam within a few months of diagnosis.<br><br>Even if you have no symptoms, it's important to get regular eye exams because the early stages of diabetic eye disease often don't cause any symptoms. By the time you start to notice symptoms, such as blurred vision, floaters, or flashes of light, the damage to your eyes may already be severe.<br><br>**Here are some of the signs and symptoms of diabetic eye disease:**<br><br>* Blurred vision<br>* Floaters<br>* Flashes of light<br>* Dark spots in your vision<br>* A sudden decrease in vision<br>* A curtain coming over your vision<br>* Pain in your eye<br><br>**If you experience any of these symptoms, you should see your eye doctor right away.** These symptoms can be a sign of a detached retina, which is a medical emergency.<br><br>**Here are some other reasons to see your eye doctor if you have diabetes:**

	* You have high blood pressure or high cholesterol.<br>* You smoke.<br>* You have a family history of diabetic eye disease.<br>* You have had laser treatment or surgery for diabetic eye disease in the past.<br><br>**By following these tips, you can help protect your vision and prevent diabetic eye disease:**<br><br>* Keep your blood sugar levels under control.<br>* See your eye doctor for regular eye exams.<br>* Don't smoke.<br>* Eat a healthy diet.<br>* Exercise regularly.<br>* Wear sunglasses to protect your eyes from the sun.<br><br>I hope this answers your question. Please let me know if you have any other questions.
**15. What can I do to protect my eyes?**	* **Manage your diabetes ABCs:** This means keeping your blood sugar levels (A1c), blood pressure, and cholesterol levels under control. You can do this by following your doctor's instructions on diet, exercise, and medication.<br>* **Quit smoking:** Smoking damages the blood vessels throughout your body, including the blood vessels in your eyes. Quitting smoking is one of the best things you can do for your overall health and eye health.<br>* **Have a dilated eye exam at least once a year:** This is the single most important thing you can do to protect your eyes from diabetic retinopathy. Your eye doctor can screen for diabetic retinopathy and other eye diseases early, when they are most treatable.<br><br>In addition to these tips, you can also help protect your eyes by:<br><br>* Eating a healthy diet: Eating a healthy diet that is rich in fruits, vegetables, and whole grains can help protect your eyes from damage.

	* Getting regular exercise: Exercise helps to keep your blood sugar levels under control and can also help to protect your eyes from damage. Aim for at least 30 minutes of moderate-intensity exercise most days of the week.<br>* Wearing sunglasses to protect your eyes from the sun's harmful rays: The sun's ultraviolet (UV) rays can damage the retina, the light-sensitive tissue at the back of the eye. Wear sunglasses that block 100% of UV rays whenever you are outdoors.<br>* Getting enough sleep: When you are sleep-deprived, your blood sugar levels can become more unstable. Aim for 7-8 hours of sleep each night.<br>* Managing stress: Stress can raise your blood sugar levels. Find healthy ways to manage stress, such as exercise, yoga, or meditation.<br><br>By following these tips, you can help reduce your risk of vision loss from diabetes.<br><br>I hope this helps!
**16. What if I already have some vision loss from diabetes?**	Sure, here is the answer:<br><br>If you already have some vision loss from diabetes, there are still things you can do to improve your quality of life. Here are some tips:<br><br>* **Get regular eye exams.** This is important even if you have already lost some vision. Your doctor can monitor your condition and make sure that you are getting the best possible care.<br>* **Control your blood sugar levels.** High blood sugar levels can damage your eyes and worsen your vision loss. Work with your doctor to create a treatment plan that will help you keep your blood sugar levels under control.<br>* **Manage your blood pressure and cholesterol levels.** High blood pressure and cholesterol levels can also damage your eyes. Work with your doctor to control these risk factors.<br>* **Quit smoking.** Smoking can damage your blood vessels, including the blood vessels in your eyes. Quitting smoking can help reduce your risk of further vision loss.<br>* **Eat a healthy diet and exercise regularly.** Eating a healthy diet and exercising regularly can help you control your blood sugar levels, blood pressure, and cholesterol levels. These lifestyle changes can also help improve your overall health and well-being.<br>* **Use low vision aids.** There are many low vision aids available that can help you make the most of your remaining vision. These aids can include magnifying glasses, telescopes, and electronic devices.

	* **Get training in low vision rehabilitation.** Low vision rehabilitation can teach you how to use low vision aids and how to adapt to your vision loss. There are many resources available for low vision rehabilitation, including books, websites, and support groups.<br><br>It is important to remember that you are not alone. There are many people who have vision loss from diabetes, and there are resources available to help you. By taking steps to protect your vision and manage your condition, you can improve your quality of life and live a full and active life.<br><br>I hope this helps. Is there anything else I can help you with?

Supplementary Table 2a: Raw Score and Rating of each Base Large Language Models, Attained for Each Question

Domain	Question No.	GPT-4			Claude			Bard		
		Score Sum (Acc) Max:9	Majority Consensus (Acc)	Average Score (Comp) Max:5	Score Sum (Acc) Max:9	Majority Consensus (Acc)	Average Score (Comp) Max:5	Score Sum (Acc) Max:9	Majority Consensus (Acc)	Average Score (Comp) Max:5
**Concepts of Diabetes**	1	7	Borderline	-	7	Borderline	-	7	Borderline	-
	2	9	Good	4.00	8	Good	3.50	7	Borderline	-
	3	7	Borderline	-	8	Good	4.00	8	Good	5.00
	4	9	Good	5.00	7	Borderline	-	8	Good	5.00
**Symptoms & Causes**	1	9	Good	4.33	7	Borderline	-	9	Good	4.33
	2	8	Good	4.00	7	Borderline	-	7	Borderline	-
	3	9	Good	4.33	9	Good	4.67	7	Borderline	-
	4	7	Borderline	-	7	Borderline	-	9	Good	3.00
**Diabetes Tests & Diagnosis**	1	9	Good	4.00	9	Good	3.67	9	Good	3.67
	2	9	Good	3.67	8	Good	4.00	8	Good	3.00
	3	9	Good	4.67	8	Good	4.00	9	Good	4.00
	4	8	Good	3.00	3	Poor		3	Poor	-
**Managing Diabetes**	1	9	Good	4.00	9	Good	4.00	9	Good	4.00
	2	6	Borderline	-	7	Borderline	-	3	Poor*	-
	3	9	Good	4.33	7	Borderline	-	9	Good	3.33
	4	9	Good	4.67	6	Borderline	-	9	Good	4.00
	5	9	Good	4.00	7	Borderline	-	8	Good	3.00
	6	9	Good	4.67	9	Good	4.00	9	Good	3.00

	7	9	Good	4.67	8	Good	4.50	9	Good	2.67
	8	9	Good	4.67	9	Good	2.67	9	Good	2.67
	9	9	Good	4.00	6	Borderline	-	9	Good	3.67
	10	8	Good	4.00	8	Good	3.00	8	Good	3.00
	11	9	Good	2.00	8	Good	2.00	9	Good	1.67
	12	9	Good	3.33	9	Good	4.33	9	Good	3.00
	13	9	Good	3.67	9	Good	3.00	9	Good	3.00
	14	9	Good	3.33	9	Good	3.00	9	Good	3.00
	15	9	Good	4.67	7	Borderline	-	9	Good	3.33
**Preventing Diabetes Problems**	1	9	Good	4.67	9	Good	5.00	9	Good	4.67
	2	9	Good	5.00	9	Good	4.67	9	Good	4.00
	3	9	Good	4.00	9	Good	4.67	9	Good	3.00
	4	9	Good	5.00	8	Good	5.00	9	Good	4.33
	5	9	Good	4.67	9	Good	4.00	9	Good	4.00
	6	9	Good	4.00	9	Good	3.00	9	Good	5.00
	7	9	Good	3.33	9	Good	3.33	9	Good	3.00
	8	9	Good	5.00	9	Good	3.67	8	Good	3.50
	9	9	Good	4.00	8	Good	4.00	9	Good	4.33
	10	9	Good	4.00	9	Good	3.33	9	Good	3.67
	11	9	Good	4.00	9	Good	4.33	9	Good	3.00
	12	9	Good	2.00	9	Good	4.33	9	Good	3.00
	13	9	Good	4.33	9	Good	3.33	9	Good	4.67
	14	9	Good	4.33	9	Good	1.67	9	Good	1.67
	15	9	Good	4.67	9	Good	4.00	9	Good	4.67
	16	9	Good	5.00	9	Good	3.67	8	Good	3.00

*Where consensus on final accuracy rating was not reached (i.e. each grader provided a different rating), the lowest score (‘poor’) was assigned.

Supplementary Table 2b: Raw Score and Rating of each RISE-enhanced Large Language Models, Attained for Each Question

Domain	Question No.	GPT-4+RISE			Claude+RISE			Bard+RISE		
		Score Sum (Acc) Max:9	Majority Consensus (Acc)	Average Score (Comp) Max:5	Score Sum (Acc) Max:9	Majority Consensus (Acc)	Average Score (Comp) Max:5	Score Sum (Acc) Max:9	Majority Consensus (Acc)	Average Score (Comp) Max:5
**Concepts of Diabetes**	1	9	Good	4.67	9	Good	4.00	9	Good	4.67
	2	9	Good	3.67	7	Borderline	-	8	Good	4.00
	3	8	Good	5.00	8	Good	4.50	9	Good	4.33
	4	9	Good	5.00	7	Borderline	-	9	Good	4.00
**Symptoms & Causes**	1	7	Borderline	-	9	Good	5.00	9	Good	3.67
	2	9	Good	4.00	8	Good	5.00	8	Good	3.50
	3	9	Good	4.33	9	Good	4.00	9	Good	4.33
	4	8	Good	5.00	7	Borderline	5.00	9	Good	3.67
**Diabetes Tests & Diagnosis**	1	9	Good	4.67	9	Good	5.00	9	Good	3.67
	2	9	Good	4.33	9	Good	5.00	7	Borderline	-
	3	9	Good	5.00	8	Good	4.50	9	Good	4.00
	4	9	Good	5.00	9	Good	4.67	9	Good	4.00
**Managing Diabetes**	1	9	Good	4.33	9	Good	5.00	9	Good	4.00
	2	9	Good	5.00	8	Good	5.00	9	Good	5.00
	3	9	Good	5.00	9	Good	4.33	9	Good	5.00
	4	9	Good	5.00	7	Borderline	-	9	Good	3.33
	5	9	Good	5.00	8	Good	4.00	7	Borderline	-
	6	9	Good	5.00	9	Good	4.00	9	Good	4.00

	7	9	Good	3.67	8	Good	2.50	9	Good	2.33
	8	9	Good	4.33	9	Good	4.33	9	Good	4.67
	9	9	Good	5.00	8	Good	4.00	9	Good	3.00
	10	9	Good	5.00	9	Good	4.33	9	Good	4.00
	11	9	Good	5.00	9	Good	3.33	9	Good	3.33
	12	9	Good	5.00	9	Good	4.00	9	Good	3.33
	13	9	Good	4.00	9	Good	4.33	9	Good	3.33
	14	9	Good	4.33	9	Good	3.33	9	Good	3.00
	15	9	Good	5.00	9	Good	1.67	9	Good	4.00
**Preventing Diabetes Problems**	1	9	Good	4.33	9	Good	5.00	9	Good	5.00
	2	9	Good	5.00	9	Good	4.67	9	Good	5.00
	3	9	Good	5.00	9	Good	4.33	9	Good	3.33
	4	9	Good	4.67	9	Good	2.67	9	Good	3.33
	5	9	Good	5.00	9	Good	3.00	9	Good	5.00
	6	9	Good	4.67	9	Good	4.33	9	Good	5.00
	7	9	Good	4.33	9	Good	4.33	9	Good	3.67
	8	9	Good	4.67	9	Good	4.33	9	Good	4.00
	9	9	Good	5.00	9	Good	4.00	9	Good	4.33
	10	9	Good	4.67	9	Good	4.67	9	Good	4.00
	11	9	Good	4.67	9	Good	5.00	9	Good	4.33
	12	9	Good	5.00	9	Good	4.67	9	Good	3.00
	13	9	Good	5.00	9	Good	3.33	9	Good	3.67
	14	9	Good	5.00	9	Good	2.67	9	Good	2.67
	15	9	Good	4.33	9	Good	3.67	9	Good	5.00
	16	9	Good	4.67	9	Good	3.67	9	Good	3.00

Supplementary Table 3: Raw Score for Understandability of each Large Language Models, Attained for Each Question

Domain	Question No.	Average Score (Understandability) Max:5					
		GPT-4	GPT-4 RISE	Claude	Claude RISE	Bard	Bard RISE
Concepts of Diabetes	1	4.67	4.67	4.00	4.00	4.33	4.33
	2	4.00	3.67	4.00	4.00	3.67	4.00
	3	4.00	5.00	4.33	3.33	4.33	4.00
	4	4.67	4.67	4.00	4.00	4.00	3.67
Symptoms & Causes	1	4.33	4.67	3.67	4.67	4.33	4.33
	2	4.33	4.33	4.67	4.33	3.67	4.00
	3	4.67	4.67	4.33	4.33	3.67	3.67
	4	4.33	5.00	4.00	4.00	3.67	4.33
Diabetes Tests & Diagnosis	1	4.00	4.67	3.67	4.33	4.00	4.33
	2	4.00	4.33	4.00	4.00	4.00	4.00
	3	4.00	4.00	4.00	4.33	4.00	3.33
	4	3.67	4.67	4.00	4.00	3.00	4.00
Managing Diabetes	1	4.33	5.00	4.33	4.67	3.67	4.33
	2	4.67	4.67	4.67	4.00	4.67	4.67
	3	4.33	5.00	3.33	3.00	3.33	4.33
	4	4.33	4.67	4.00	4.33	4.00	4.00
	5	4.67	4.67	4.00	3.67	4.33	4.67
	6	4.67	4.67	4.33	4.33	4.00	3.67
	7	4.33	4.00	4.33	3.33	3.67	3.00
	8	4.33	5.00	4.00	4.00	3.67	4.67

	9	4.33	5.00	4.00	4.67	4.00	4.00
	10	4.00	4.67	3.67	4.33	3.33	4.00
	11	3.67	5.00	4.00	3.67	3.33	3.67
	12	4.00	4.67	4.33	4.33	3.67	4.33
	13	4.67	5.00	4.00	4.00	4.33	4.00
	14	4.33	4.67	3.67	3.67	3.67	3.67
	15	4.00	4.67	2.33	2.33	4.00	4.33
**Preventing Diabetes Problems**	1	4.67	4.67	4.33	4.00	4.67	4.67
	2	4.67	4.33	4.00	4.33	4.00	4.67
	3	4.67	4.67	3.67	4.33	3.67	4.33
	4	4.67	4.33	4.33	3.67	4.33	4.00
	5	4.33	4.67	4.00	3.67	4.00	4.33
	6	4.00	5.00	3.67	4.67	4.33	4.67
	7	4.00	4.67	4.33	4.67	4.00	4.67
	8	5.00	4.00	4.00	4.33	4.00	4.00
	9	4.00	5.00	3.67	4.00	4.33	4.33
	10	4.00	4.67	4.33	4.67	4.33	4.00
	11	4.67	4.67	4.33	4.33	4.00	4.33
	12	4.33	4.67	4.33	4.67	4.00	3.67
	13	4.67	4.67	4.33	4.00	4.67	4.00
	14	4.33	4.67	3.33	3.67	3.67	4.67
	15	4.33	4.33	4.33	4.00	4.00	4.33
	16	4.00	4.67	3.67	4.00	4.00	4.67
